# Mechanisms of *In Vivo* Ribosome Maintenance Change in Response to Nutrient Signals*[Fn FN2]

**DOI:** 10.1074/mcp.M116.063255

**Published:** 2016-12-08

**Authors:** Andrew D. Mathis, Bradley C. Naylor, Richard H. Carson, Eric Evans, Justin Harwell, Jared Knecht, Eric Hexem, Fredrick F. Peelor, Benjamin F. Miller, Karyn L. Hamilton, Mark K. Transtrum, Benjamin T. Bikman, John C. Price

**Affiliations:** From the ‡Department of Chemistry and Biochemistry,; ¶Department of Physics and Astronomy,; ‖Department of Physiology and Developmental Biology, Brigham Young University, Provo, Utah 84602;; §Department of Health and Exercise Science, Colorado State University, Fort Collins, Colorado 80523

## Abstract

Control of protein homeostasis is fundamental to the health and longevity of all organisms. Because the rate of protein synthesis by ribosomes is a central control point in this process, regulation, and maintenance of ribosome function could have amplified importance in the overall regulatory circuit. Indeed, ribosomal defects are commonly associated with loss of protein homeostasis, aging, and disease (1–4), whereas improved protein homeostasis, implying optimal ribosomal function, is associated with disease resistance and increased lifespan (5–7). To maintain a high-quality ribosome population within the cell, dysfunctional ribosomes are targeted for autophagic degradation. It is not known if complete degradation is the only mechanism for eukaryotic ribosome maintenance or if they might also be repaired by replacement of defective components. We used stable-isotope feeding and protein mass spectrometry to measure the kinetics of turnover of ribosomal RNA (rRNA) and 71 ribosomal proteins (r-proteins) in mice. The results indicate that exchange of individual proteins and whole ribosome degradation both contribute to ribosome maintenance *in vivo*. In general, peripheral r-proteins and those with more direct roles in peptide-bond formation are replaced multiple times during the lifespan of the assembled structure, presumably by exchange with a free cytoplasmic pool, whereas the majority of r-proteins are stably incorporated for the lifetime of the ribosome. Dietary signals impact the rates of both new ribosome assembly and component exchange. Signal-specific modulation of ribosomal repair and degradation could provide a mechanistic link in the frequently observed associations among diminished rates of protein synthesis, increased autophagy, and greater longevity (5, 6, 8, 9).

Cells in the body achieve protein homeostasis (proteostasis)^1^ by carefully balancing the synthesis and folding of each protein against the protein degradation and cellular proliferation rates. Controlled shifts in proteostasis occur during cell differentiation, and in response to stimuli (1, 4), while uncontrolled changes promote neurodegenerative disease (10–13), cancer (14–16), and aging (1, 5, 7, 17, 18). This suggests that each cell coordinately regulates the ribosome, proteasome, and other key structures of protein metabolism to achieve proteostasis. However, the regulatory mechanisms for coordination and how the proteome is remodeled to achieve a new proteostasis are poorly understood.

Diets restricting calories or amino acids protect against aging and the diseases of aging in model organisms (19, 20). Low calorie diets have been shown to reduce rates of protein synthesis and degradation for much of the observed proteome (5, 6, 17). Recent reports suggest that high protein synthesis demand is associated with reduced ribosomal accuracy (2) and efficiency (21). This suggests that maintaining ribosome quality is an intricate task that requires constant cellular effort.

Translation rate is a metric of ribosome quality (22). Ribosomes that stall during protein production are immediately tested by the ribosome quality control (RQC) complex (22). The RQC specifically tests the large subunit for activity (23) and presumably sequesters inactive ribosomes. Degradation of unnecessary or damaged ribosomes often occurs through directed autophagy (ribophagy) (24). Ribophagy is presumably one of the primary roles for autophagy; indeed, the earliest descriptions of autophagic vesicles show ribosomes in the interior (25). An important open question though is: Can dysfunctional ribosomes be repaired, or is ribophagy the only option?

Exchange of damaged ribosomal components could allow the cell to repair faulty ribosomes instead of degrading the entire structure. The formation of new ribosomes is extremely costly and has been estimated to account for 15% of the protein synthesis budget (26). In yeast, protein synthesis accounts for at least 90% of the energy usage (27). Damaged ribosomes in *Escherichia coli* have been shown to regain activity after exchange of r-proteins for undamaged copies (28). Although it has never been demonstrated in eukaryotes, exchange of damaged protein components could reduce the total energy expenditure to maintain active ribosomes.

Here, we show that exchange of r-proteins is occurring *in vivo*. Using metabolic labeling (Fig. 1*A*) and a kinetic model, we calculate exchange rates between assembled and free pools (Fig. 1*B*). Further, we show that r-protein exchange and ribophagy rates change with dietary signals. We compare mice fed an *ad libitum* (AL) *versus* a restricted diet (dietary restriction, DR) and observe that kinetically there are three groups of proteins in the assembled ribosome. One group is never exchanged and is degraded via ribophagy with the rRNA. The second group is exchanged multiple times with cytosolic copies and has members with either fast or slow cytosolic turnover. A third group of proteins alternates between the first two groups. We find that both ribophagy and r-protein exchange are modulated by dietary signaling. Our observations offer insight into the connection between reduced protein synthesis (5, 6, 9, 17), and increased autophagy (29–31) with increased health and longevity.

**Fig. 1.**
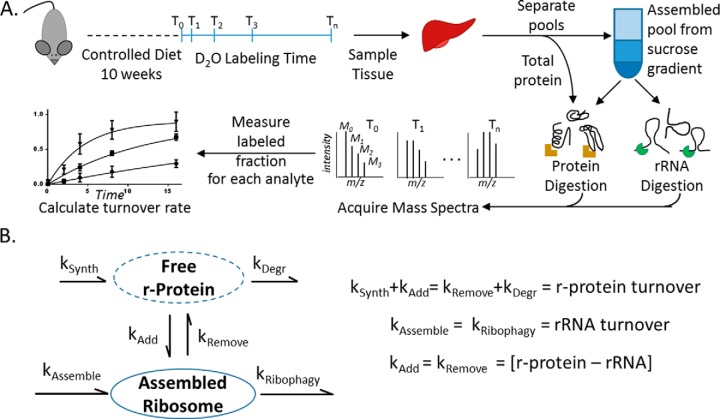
**Experimental Overview:** Workflow for heavy isotope labeling, analyte isolation, and measurement of turnover rates (*A*). Kinetic model for utilizing turnover measurements to describe ribosome maintenance and turnover (*B*). Under these experimental conditions opposing rates are equal maintaining homeostasis.

## MATERIALS AND METHODS

### 

#### 

##### Mouse Handling

Mice were housed, diet restricted, and metabolically labeled according to protocols approved by the Brigham Young University Institutional Animal Care and Use Committee. Ten-week-old male C57BL/6 mice were obtained from Charles River Laboratories. For the duration of the experiment, mice were housed in a specific-pathogen-free facility with 12 h light/dark cycles. All mice were fed an AL diet for 1 week with 3–4 mice per cage. AL consumption amounts were monitored during the first week. After 1 week, mice were separated and assigned to DR (*n* = 20) or AL (*n* = 19) fed diet on Harlan 8604 chow. The DR cohort then received a metered 65% daily ration for the rest of the study. The low calorie diet used in this study restricted every component of the diet equally, which classically is termed DR. Mouse weights were recorded each week. After 10 weeks of treatment, mice received an intraperitoneal sterile D_2_O injection (35 μl/g body weight) to immediately bring body water to 5% D_2_O as previously described (6). Drinking water was supplemented to 8% molar percentage excess D_2_O to maintain 5% body water throughout the experiment. Mice (*n* = 17) were sacrificed in duplicate (*n* = 2) at time points 0 days (no D_2_O injection), 0.4 days, 1 day, 2 days, 4 days, 8 days, 16 days, and triplicate at 32 days. Mice were immediately dissected, blood was extracted by cardiac puncture for percentage D_2_O analysis, and organs were either used fresh for mitochondrial respiration measurements or flash frozen on blocks of solid CO_2_. Tissues were stored at −80 °C.

##### Mitochondrial Respiration

Fresh liver tissue was quickly removed from exsanguinated mice and immediately placed in ice-cold mitochondrial respiration buffer 05 (MiR: 0.5 mm EGTA, 10 mm KH_2_PO_4_, 3 mm MgCl_2_-6 H_2_O, 60 mm K-lactobionate, 20 mm HEPES, 110 mm Sucrose, 1 mg/ml fatty acid free BSA, pH 7.1) and trimmed of connective tissue. Tissue was gently separated and homogenized under a surgical scope (Olympus, ST) to particles of around 1 mg. Homogenate was then transferred to a tube with chilled MiR05 and 50 μg/ml saponin and rocked at 4 °C for 30 min, then washed in MiR05 at 4 °C for at least 15 min before use. High-resolution O_2_ consumption was determined at 37 °C using the Oroboros O_2_K Oxygraph. Before addition of sample into respiration chambers, a baseline respiration rate was determined. After addition of sample, the chambers were hyperoxygenated to ∼350 nmol/ml. Following this, respiration was determined as indicated. Lastly, residual oxygen consumption was measured by adding antimycin A (2.5 μm) to block complex III action, effectively stopping any electron flow and providing a baseline respiration rate.

##### Isolating Assembled Ribosomes

Separation of free ribosomal proteins and assembled ribosomes was performed using a sucrose gradient as follows. Frozen liver, 62–215 mg, from time points 0, 1 day, 4 days, 8 days, and 16 days, was homogenized in polysome buffer (20 mm Tris/HCl, 150 mm NaCl, 5 mm MgCl_2_, 1 mm dithiothreitol, 1:100 dilution protease inhibitor mixture (Sigma), and 1% Triton X-100) using a bead homogenizer: 30 s, 4 m/s, repeated 1–3 times depending on need. Lysate was placed into a new Eppendorf tube and clarified by centrifugation at 20,000 *g* for 20 min at 4 °C. After clarification, sample was decanted then ∼300 μl were passed through a 2.2 ml sucrose cushion (1 m sucrose, 20 mm Tris/HCl, 150 mm NaCl, 5 mm MgCl_2_, and 1 mm dithiothreitol) for 12 h at ∼200,000 *g* (40,600 rpm) 4 °C using a Ti-55 rotor on the Optima l-100XP Ultracentrifuge (Beckman Coulter). After centrifugation, sucrose was decanted, and the ribosome pellet was suspended in 6 m guanidine/HCl, 100 mm Tris/HCl, pH 8.5.

##### Polysome Analysis

The polysome analysis was patterned on the method of Zhao *et al.* (32). Sucrose density gradients were prepared using five layers of buffered sucrose (50 mm Tris, 50 mm ammonium acetate, 12 mm MgCl_2_, pH 7.0, and either 7%, 21%, 33%, 47%, or 60% sucrose). 1 ml of each mixture was placed in a centrifuge tube from high density (60%) at the bottom to low density (7%) at the top. This step gradient was stored at 4 °C overnight to allow formation of a continuous gradient. The next morning, flash-frozen liver tissue was homogenized in lysis buffer (15 mm MgCl_2_, 200 mm KCl, 1% Triton x-100, 100 μg/ml cycloheximide, 2 mm DTT, 0.1% diethyl pyrocarbonate, pH 7.4) at 6 m/s for 60 s in a MP-Biomedicals FastPrep®-24. The homogenate was centrifuged to clarify (14,000 *g*, 5 min, 4 °C). Approximately 1.2 mg of total protein were loaded onto each gradient. The sample was then separated within the sucrose gradient using high speed centrifugation (99,526 *g*, 4 h, 4 °C) in a Beckman Coulter Optima™ l-100 XP. Polysomes were analyzed using an Isco UA-6 Absorbance Detector at 254 nm. Six mice from AL and six from DR were run in duplicate for assessment of polysome profiles.

##### Mass Spectrometry Sample Preparation

New DNA was measured as the isotope incorporation into the ribose moiety of the nucleoside bases, as previously described (33). New rRNA was also measured as the isotope incorporation into the ribose moiety of the nucleoside bases by adapting the GC/MS method to follow the heavier ribose instead of the deoxy-ribose. DNA was isolated from tissue homogenates using the DNAeasy kit. RNA was extracted from the assembled ribosomes using the Purlink RNA minikit (Life Technologies). rRNA was isolated by homogenizing liver tissue in 20 mm Tris (pH = 7.2), 0.2 m sucrose, 2 mm MgCl_2_, 150 mm KCl, 1 mm dithiothretol with protease inhibitor mixture (Sigma), and cycloheximide (Sigma). This homogenate was clarified by brief centrifugation (10 min at 14,000×G). Clarified homogenate was spun at 100,000×G through a 1 m sucrose cushion in the same buffer for 16 h. The supernatant was discarded, and the pellet containing assembled ribosomes were resuspended using 100 μl 6 m guanidine HCL, pH = 8. Isolated RNA/DNA was hydrolyzed overnight at 37 °C with nuclease S1 and potato acid phosphatase. Hydrolysates were reacted with pentafluorobenzyl hydroxylamine and acetic acid and then acetylated with acetic anhydride and 1-methylimidazole. Dichloromethane extracts were dried and resuspended in ethyl acetate to be analyzed by GC/MS.

Assembled pool protein samples were prepared for protein mass spectrometry using modified filter-aided sample preparation (34). Briefly, protein was denatured in 6 m guanidine/HCl 100 mm Tris/HCl (pH 8.5); cysteines were reduced using dithiothreitol and alkylated using iodoacetamide. Samples were placed on 500 μl 30 kD filters and washed 2–3 times on the filters using 6 m guanidine/HCl 100 mm Tris/HCl, pH 8.5. The guanidine solution was removed by two to three 25 mm ammonium bicarbonate washes. Proteins were resuspended in 25 mm ammonium bicarbonate and digested overnight using Pierce MS-Grade Trypsin in a 1:50 (w:w) ratio or minimum 0.1 μg or 0.5 μg of trypsin per sample. Trypsin digest was quenched using phenylmethane-sulfonylfluoride or centrifuging through above-mentioned filters to remove tryspin. Samples were spun through filters, placed in mass spec vials, speed vacuumed to dry, and then suspended at ∼1 μg/μl in 3% acetonitrile 0.1% formic acid.

Total pool samples were prepared from whole liver lysates protocols similar to ribosomal samples. Liver was homogenized in a 100 mm ammonium bicarbonate solution with the protease inhibitor mixture (Sigma), aiming for a final concentration of ∼10 mg/ml protein concentration. Approximately 500 μg of protein were lysed in 6 m guanidine/HCl 100 mm Tris/HCl and subject to similar filter-aided preparations and trypsin digestion as described above. After digestion, samples were spun through filters, speed vacuumed to dry, and resuspended in 10 mm LC-MS grade ammonium formate, pH 9.5. Samples were fractionated using high pH C18 high performance liquid chromatography (HPLC), which is orthogonal to low pH C18 chromatography (35). Fractionation was performed using the 1260 HPLC Infinity (Agilent) and the Gemini 50 × 2.00 mm C18 column with 3 μm beads and 110 Angstrom pore size. Peptides were eluted using a 10 mm ammonium formate, pH 9.5, H_2_O/acetonitrile gradient from 3% B to 60% B over 40 min flowing at 1 ml/min. Gradient A was 97% H_2_O, 3% acetonitrile, 10 mm ammonium formate, pH 9.5. Gradient B was 10% H_2_O, 90% acetonitrile, 10 mm ammonium formate, pH 9.5. 1 ml fractions were collected. 1 ml fractions were pooled into eight fractions by pooling every eighth fraction. For instance, fractions 1, 9, 17, 25, … would be pooled into one fraction. Pooled fractions were speed vacuumed to dryness then suspended in 200 μl of 80% acetonitrile (to extract peptides but leave some salts) and decanted into a mass spectrometry vial. Samples were again speed vacuumed to dry and then suspended in 40 μl of 3% acetonitrile 0.1% formic acid for LC-MS analysis.

##### LC-MS Proteomics Acquisition

As described previously, protein identification and kinetic acquisition were performed on the Agilent 6530 Q-ToF mass spectrometer coupled to capillary and nanoflow Agilent 1260 HPLC using the chipcube nanospray source (6, 36). Peptides were eluted from the Agilent C18 Polaris chip at 300 nl/min using an H_2_O-acetonitrile gradient acidified to pH 4 by use of Pierce LC-MS-grade formic acid. Buffer A was 3% acetonitrile, 0.1% formic acid. Buffer B was 97% acetonitrile, 0.1% formic acid. The elution gradient is as follows: 0 min, 100% A; 0.1 min, 95% A; 27 min, 40% A; followed by high percentage B column washing and low percentage B equilibration. The Agilent 6530 Q-ToF mass spectrometer was run in 2 Ghz high dynamic range mode. Protein identification runs were performed in MSMS mode using collision-induced dissociation with nitrogen gas. MS1 and MS2 data were collected at a maximum rate of 4 spectra/s with CID fragmentation on the top 10 most abundant precursors. Dynamic exclusion was set to 0.2 min. Kinetic acquisitions were performed in MS-only mode and collected at 1 spectra/s. MS only mode increases signal intensity, improves signal-to-noise, and gives more scan points per elution chromatogram, greatly enhancing isotopomer analysis accuracy. Raw data are available for download at the Chorus Project (ID# 1148).

##### Peptide Identification

Peptide identifications were made using SpectrumMill B.06 then overlaid onto kinetic acquisitions. SpectrumMill searches were performed against the Uniprot Mouse database (12–2015, with 16,802/51,418 entries searched) with MS1 tolerance ±20ppm and a MS2 tolerance ±50 ppm, cabomidomethylation as a static modification and pyroglutamic acid (n-term) and oxidation as dynamic modifications.

Searches were performed using trypsin as a digestion enzyme allowing two missed cleavages at lysine or arginine. A second search with no specific enzyme was performed against a restricted library of identified proteins. Following general recommendations from Agilent, peptides with a score greater than 7 and greater than 60% scored peptide intensity were used for further analysis. False discovery rate was calculated by the built-in algorithms of the Spectrum Mill software and was set at 1% for peptides and proteins. Identified peptides were exported and used to calculate mass isotopomer distributions and extract peptide isotope patterns from MS-only acquisitions (supplemental information).

##### Mass Isotopomer and Kinetic Analysis

MS-only isotopomer data were extracted based on peptide identification from MSMS acquisition using *m/z* (± 12 ppm) and retention time alignment (± 0.8 min). Data extraction and analysis were conducted using our DeuteRater (37) software tool based on previous publications (6, 36). Briefly, isotope peaks M0-M4 were normalized against the sum of the signal intensity then compared with theoretical calculations based on percentage D_2_O enrichment to determine fraction deuterium-enriched (new) peptide (as previously described (38)). Theoretical calculations were determined using the eMASS algorithm and based on the number of possible deuterium incorporation sites per amino acid (39). The theoretical changes in abundance of each isotope peak M0-M4 were compared against experimental changes at each time point in order to determine a time-dependent percentage of newly synthesized peptide reported for each isotope peak. Thus, for each peptide, there are up to five (M0-M3 for peptides below and M0-M4 for peptides above *m/z* = 2400) semi-independent measurements of the peptide turnover, as previously described (36, 38). We used the standard deviation between these measurements as a metric of the measurement precision for that peptide. If peptide precision was low (*i.e.* standard deviation exceeded 0.1) the data point was removed from downstream analysis (Fig. S2). Additional filters were also applied to remove peptides with total relevant intensity below 20,000 counts and a retention time deviation greater than 0.5 min.

The median percentage new was calculated at each point, and outliers (defined as greater than 1.4X the median absolute standard deviation) were removed from the calculation of the protein percentage new. All peptide measurements for an individual time point that passed these filters were weighted equally in the calculation of the fraction new protein at that time point. As described previously, the combined fraction new measurements were fit using a nonlinear least squares regression based on first-order kinetic rate equations (6). The proteins with high precision data at three or more time points were fit according to first-order rate kinetics. We required three or more labeled time points in order to increase the confidence of the rate constant (Fig. S2). For the regression fit, time point zero was set to 0% new and was given a standard deviation of 0.05 based on the accuracy during long-term performance of this instrument. The standard deviation and confidence interval from these fits were used to compare protein and rRNA in subsequent analysis. Coefficients of variance (standard deviation of the fit over the turnover rate) above 0.2 are considered high confidence fits.

##### rRNA Turnover Analysis

After running in scan mode, peaks were identified at *m/z* 212 and 433. From these, it was determined that the 212 had better peak shape and accurately predicted natural abundance in an unlabeled sample and was therefore used for subsequent analyses. Based on accurate mass and isotope distribution, we identified the fragment. Mass isotopomer calculations were performed on the fragment using EMass software based on three incorporated deuteriums. The monoisotopic M+1, and M+2 were used for percentage new RNA analysis and errors. Two mice were measured in triplicate at each time point. Rates and confidence intervals were solely based on standard deviations from least squared fits to first-order rate kinetics. Data were fit using a single-phase association curve in GraphPad Prism.

##### Quantitative Polymerase Chain Reaction

Quantitative polymerase chain reaction (qPCR) was performed using SYBR Green on an Applied Biosystems 7500 instrument. Reverse transcription was performed with the iScript cDNA synthesis kit (Bio Rad) and SYBR Green master mix (Bio-Rad). Primers: 18s rRNA forward (CTTAGAGGGACAAGTGGCG) reverse (ACGCTGAGCCAGTCAGTGTA); 16s mitochondrial rRNA forward (CGAGGGTCCAACTGTCTCTT) reverse (GGTCACCCCAACC GAAATTT); vRNA forward (GCTGAGCGGTTACTTTGACA) reverse (GTCTCGAACCAA ACACTCATG); TATA forward (ACAGCCTTCCACCTTATGCT) reverse (GATTGCTGTA CTGAGGCTGC). qPCR instrument parameters were as follows: Stage 1 (one cycle) 50 °C for 2 min; Stage 2 (one cycle) 95 °C for 15 s; Stage 3 (30–45 cycles depending on need), 95 °C for 15 s, 59 °C for 1 min. Melt curves to determine product purity and efficiency calculations were performed on all primer sets (supplemental data). qPCR primer efficiency was calculated using Real-Time PCR Miner 4.1.(37) Relative concentrations were calculated using method by Pfaffl, which corrects for differences in primer efficiency (38).

##### Calculation of Exchange Rate

Using the kinetic model (Fig. 1*B*), the rate of change for each protein in each pool can be described mathematically as

(Eq. 1) $$\text{Free}\;\text{pool}:\frac{d\left[P_{i\;free}\right]}{dt}=k_{synth}-k_{\deg}-k_{add}-k_{remove}=0$$

where *P_i_* is an individual protein concentration, *k_synth_* is the synthesis rate, *k_deg_* is the degradation rate, and *k_add_* is the rate of proteins adding to the assembled ribosome, while *k_remove_* is the rate of proteins leaving the assembled ribosome and reentering the free pool.

(Eq. 2) $$\text{Assembled}\;\text{pool}:\frac{d\left[P_{i\;assembled}\right]}{dt}=k_{assembled}-k_{ribophagy}-k_{add}-k_{remove}=0$$

where, in addition to the terms above, *k_assemble_* is the apparent rate of formation and export of the intact active ribosome from the nucleolus to the cytosol. *k_r ibophagy_* is the rate of whole ribosome degradation; this includes both protein and rRNA.

(Eq. 3) $$\text{Ribosome}\;\text{pool}:\frac{d\left[rRNA\right]}{dt}=k_{assembled}-k_{ribophagy}=0$$

(Eq. 4) $$\text{Total}\;\text{protein}\;\text{pool}:\frac{d\left[P_{i\;total}\right]}{dt}=\frac{\frac{d\left[P_{i\;free}\right]}{dt}+\frac{d\left[P_{i\;assembled}\right]}{dt}}{2}=0$$

(Eq. 5) $$\text{Exchange}\;\text{rate}:\frac{d\left[P_{i}\right]}{dt}=\left[\frac{d\left[P_{i\;assembled}\right]}{dt}-\frac{\left[rRNA\right]}{dt}\right]=k_{add}-k_{remove}=0$$

##### Experimental Design and Statistical Rationale

There were two biological replicates for each time point in each kinetic pool measurement (assembled/total) of each diet (AL *versus* DR). Each kinetic rate was determined by up to 10 biological replicates, so no technical replicates were included. A minimum of three time points were required to fit a rate constant because with three time points the rates are well constrained (Fig. S2). As described above, peptide measurements were included if they met the retention time and precision filters. Statistical analysis and graphing was performed using GraphPad Prism and the Numpy software package. GraphPad Prism was used to fit the DNA to a first-order kinetic (33) and rRNA to a second-order kinetic (40, 41) as previously described and to calculate 95% confidence intervals. An in-house Python tool termed DeuteRater (37) was used to calculate the fraction new peptide, fit the protein turnover rates to a single pool model using first-order rate kinetics, and calculate 95% confidence intervals. If rates were outside of the 95% confidence interval of rRNA or another protein they were considered significantly different.

## RESULTS

### 

#### 

##### Short-Term Dietary Restriction (DR) Elicits the Canonical Physiological Changes Associated with Lifespan Extension

**Fig. 2.**
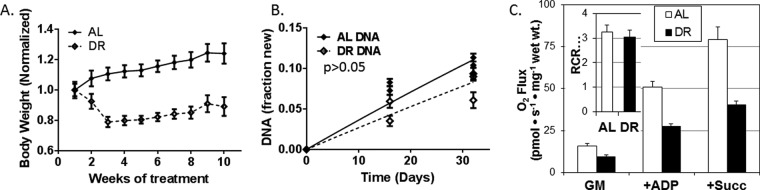
**Short-term DR elicits the classic physiological and biochemical adaptations associated with lifespan extension.** After an initial weight loss, DR mice continued to gain weight at a reduced rate (*A*). Liver cell proliferation was reduced (*p* < 0.01) from 0.04% per day to 0.03% per day (*B*). DR reduces mitochondrial respiration, samples were treated with: GM: glutamate (10 mm) + malate (2 mm); + ADP (2.5 mm adenosine diphosphate); +Succ: (succinate 10 mm). Respiratory control ratio (RCR; *inset*) was unchanged (C).

Restricted mice (*n* = 18) lost weight relative to (AL) controls (*n* = 18) for the first 2 weeks of the restricted diet, after which they gained weight at a rate similar to controls (Fig. 2*A*). In order to ensure that both the AL and the DR mice were at homeostasis, the dietary treatment was continued for 10 weeks prior to initiating the metabolic labeling experiment (Fig. 1). Using previously described methods (6), we found that the cell proliferation rate was reduced 25% (*p* < 0.05) in DR (Fig. 2*B*). Consistent with previous reports, we observed liver tissue respiration capacity was decreased by DR (42) for both complex 1 and complex 2 driven electron transport (Fig. 2*C*). Respiratory efficiency, which is the ratio of the ADP-dependent O_2_ usage (+ADP) *versus* nonspecific oxygen usage (glutamate+malate), was not changed (inset to Fig. 2*C*).

##### Ribosome Activity Is Reduced during DR

**Fig. 3.**
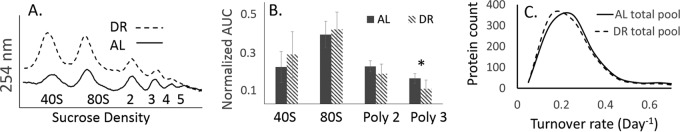
**Short-term DR reduces percentage of active ribosomes.** Representative data from polysome analysis in which a density gradient separates individual subunits from active ribosomes in both AL and DR tissue (*A*). Quantitation of the populations suggests that DR increases the free 40S state by significantly decreasing the number of active polysomes (Poly 3 in *B*). Comparison of turnover rates for 1050 matched proteins showed a statistically nonsignificant general reduction of protein turnover rates in DR (*C*).

Similar to published protocols (32), we used a sucrose gradient to separate out the various ribosome states within the liver tissue of AL and DR animals (Fig. 1). Polysome analysis (Fig. 3*A*) was conducted twice on six animals from each diet group, and the normalized area under the curve of each ribosomal species was quantified. The number of ribosomes actively transcribing mRNA (polysomes) was significantly lower (*p* < 0.05) in DR tissue (Fig. 3*B*). Interestingly, the total number of ribosomes, as measured by qPCR, was not significantly changed by DR (Fig. S1). These combined results suggest that during DR a lower percentage of available ribosomes are actively producing protein, similar to previous reports (5).

We also compared ribosome activity by measuring turnover (synthesis + degradation) rates for 1050 proteins observed in both AL and DR (Fig. 3*C*). Consistent with previous investigations of changes in protein turnover during low calorie diets, we observed that during DR the median protein turnover rate was 5% slower (Fig. 3*C*). This decrease is smaller than in previously reported studies (5, 6) and not statistically significant. We are currently investigating how specific components of the diet may have modified regulation of global protein homeostasis during lower calorie intake. Together, these results suggest that the overall ribosome pool has slightly reduced activity with more ribosomes in the cell as dissociated subunits in DR tissue.

##### Turnover Rate of the rRNA Backbone Is Slightly Faster in DR Tissue

**Fig. 4.**
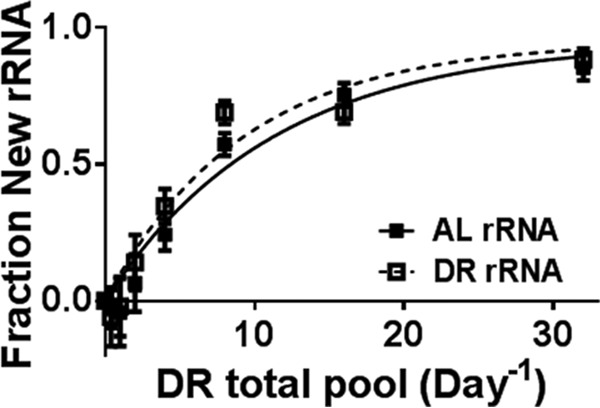
**rRNA turnover measures eukaryotic ribophagy.** Deuterium incorporation into the ribose of the rRNA bases is not significantly slower in AL (10.1 ± 1.2% Day^−1^) than the DR (11.1 ± 1.7% Day^−1^).

Assembled ribosomes were isolated from the liver tissue of two animals at each of the eight time points and separated into two samples for analysis of rRNA and r-protein turnover (Fig. 1*A*). New rRNA was measured as the isotope incorporation into the ribose moiety of the nucleoside bases, similar to the accepted method for measuring DNA synthesis (33). The fraction new rRNA increased exponentially with time (Fig. 4). Previously, rRNA modeling has shown that in rapidly dividing cultures incorporation of a precursor pool improved model accuracy (40, 41, 43). We compared both the single pool and the precursor model for fitting the rRNA data. Incorporating the precursor pool as described in the literature resulted in a precursor pool size of 13 and 6% for AL and DR tissue, respectively. We observed a nonsignificant increase in rRNA turnover in the DR tissue (11.1 ± 1.7% Day^−1^) relative to AL tissue (10.1 ± 1.2% Day^−1^).

We checked whether mitochondrial ribosomes are a potential source of contaminating rRNA (Fig. S1). Protein mass spectrometry of the isolated ribosomes confirmed that minor amounts of mitochondrial r-proteins were present. Mitochondrial r-proteins share little or no homology with the eukaryotic ribosomes and therefore cannot confound the measurement of protein kinetics. As measured by our assay, mitochondrial rRNA cannot be differentiated from eukaryotic rRNA. Therefore, in order to determine the effect of mitochondrial rRNA on measured ribophagy rates, we isolated ribosomes both with and without nonionic detergent. With detergent, we measured a relative fivefold higher concentration of eukaryotic ribosomal rRNA. Without detergent, the total ribosomal isolation was less efficient, but the amount of eukaryotic 18S to mitochondrial 16S rRNA as measured by qPCR increased 50% (Fig. S1). The large relative change in the mitochondrial content did not change our rates of rRNA turnover in these samples (Fig. S1), indicating that the absolute contamination from the mitochondrial ribosome rRNA is relatively minor and does not bias our results for cellular rRNA and ribophagy rates.

Simple stoichiometry of the rRNA within the cell supports the conclusion that mitochondrial contamination will not bias rRNA rates. Based on studies in yeast, there are ∼14 times more eukaryotic than mitochondrial ribosomes (44). The eukaryotic ribosome contains ∼3 times the number of rRNA bases (45). Therefore, the rRNA bases from eukaryotic ribosomes are about 42-fold more abundant than mitochondrial, which suggests that the rRNA turnover rate will dominated by the eukaryotic ribosomes. Our sample preparation also removed many of the mitochondria prior to ribosome isolation and should further bias the sample toward eukaryotic ribosomes.

##### DR Significantly Changes the Turnover Rates of Proteins in the Assembled Ribosome

**Fig. 5.**
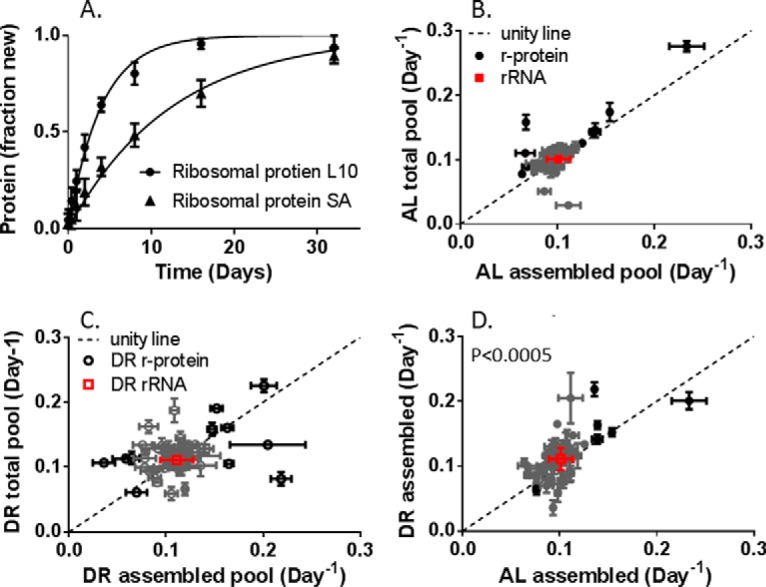
**Turnover rates of r-protein are equivalent between pools but not between dietary groups.** Individual ribosomal proteins turnover at different rates within the assembled ribosome (*A*) but have the same rate in both assembled and total pools (*B, C*). Approximately 80% of r-protein turnover rates (*gray circles*) are within the confidence interval of the rRNA (*red*). r-proteins with unusually fast or slow turnover (*black circles*) were observed in both AL (*B*) and DR (*C*) tissue. Comparison of assembled AL and DR (*D*) showed that some of the outlier proteins are the same in both conditions.

Similar to our previous studies, isotope incorporation into multiple tryptic peptides was measured for each protein in multiple samples along the time-course (6, 36, 46). We isolated assembled ribosomes for analysis of r-protein turnover (Fig. 1*A*). Turnover rates were significantly different between the individual r-proteins (Fig. 5*A*), we measured turnover rates for 71 of the 80 integral r-proteins, with turnover rates ranging from 4–25% per day (Supplemental Table). Interestingly, the median turnover rate for the entire group of r-proteins in the assembled ribosome is the same as the rRNA turnover rate (0.10 Day^−1^ in AL, 0.11 Day^−1^ in DR). Comparison of the individual protein turnover rates to the rRNA rate shows that many (∼80%) of the proteins turnover within two standard deviations of the rRNA rate (Figs. 5*B*–5*D*, gray symbols). This suggests that the rRNA and these proteins may all be synthesized and degraded together as a unit, as occurs during ribophagy. Also, the large number of individual protein measurements makes the DR-dependent increase in ribophagy statistically significant (Fig. 5*D*, *p* < 0.0005).

DR also increased the observed range of rates within the assembled ribosome, but the rRNA turnover confidence interval (defined as two standard deviations) was also wider, so the percentage of nonexchanging proteins stayed around 80%. The proteins at the 60S/40S interface (L10, L36A, L24, L34, and L19) were still among the fastest turnover proteins within the assembled ribosome and were replaced faster than the rRNA confidence interval (Fig. 3*D*). Although there were several proteins slower than the rRNA confidence interval in both AL and DR, these proteins were not the same in both groups (Fig. 5*D*). Importantly, each of these protein turnover rates was calculated from multiple measurements of multiple peptides.

##### Turnover for Most r-proteins Is Not Different between the Assembled and Total Pools

We were not confident that we could keep assembled and free pools separate during tissue homogenization. Therefore, we measured the total pool (free + assembled) and assembled pool turnover. The turnover rate within the total cellular lysate represents the average of the free and assembled protein pools (Equation 4). A deviation between assembled and total pool turnover rates could indicate that exchange is a rate-limiting process. Generally, we observed that there was no statistically significant change in turnover between assembled and total protein pools regardless of dietary intervention (Figs. 3*A* and 3*B*). Individual proteins had altered turnover in either AL or DR mice, but not in both. Experiments in *E. coli* suggest that greater than 95% of the r-proteins in the cell are bound in the ribosome structure (40). A mass-averaged turnover measurement would suggest that the total pool rate should reflect the assembled pool similar in these measurements.

##### Calculation of r-protein Exchange Rates

A kinetic model for ribosome biogenesis and maintenance was used to compare the turnover of the assembled and total r-proteins and to calculate the exchange between pools (Fig. 1*B*). We measured the relative concentration of the rRNA by qPCR between experimental cohorts and did not see a significant change. The unchanged rRNA concentration and the steady rate of weight increase (Fig. 2) after the 10 weeks of dietary acclimation reinforces the conclusion that these proteins are at a condition of homeostasis. Therefore, under these conditions, the total concentrations are constant, and the opposing rates are balanced (*i.e.* k*_add_* is equal to k*_remove_*).

Using the kinetic model, the rate of change for each protein in each pool can be described mathematically as shown in supplemental methods. We have directly measured the turnover of the rRNA, which represents the turnover of the ribosome as a unit (Equation 3). We also measured the protein turnover rate within the assembled ribosome (Equation 2, *k_assemble_* + *k_add_*, note that *k_assemble_* = *k_ribophagy_* and *k_add_* = *k_remove_* due to homestasis) and the total pool (Equation 4, *k_synth_* + *k_add_*, noting that *k_synth_* = *k_deg_* and *k_add_* = *k_remove_*). We therefore calculate the exchange rate (*k_add_ and k_remove_*) as the absolute value of the difference in turnover rates of the assembled pool and the rRNA (Equation 5: Fig. 1*B*).

**Fig. 6.**
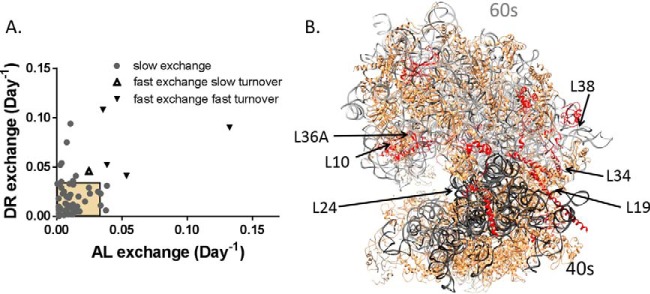
***In vivo* exchange of r-proteins.** Most r-proteins exchange slowly (*gray circles*) and are within the expected range for ribophagy (*yellow box* in *A*). Exchange rates for some r-proteins is outside of the expected ribophagy rate in one condition (*gray circles* outside in white field for AL or DR). Some exchange rates are rapid enough that these proteins are rarely degraded by ribophagy and instead are degraded in the free pool (*triangles*). Some members of this group have slow cytosolic turnover (*A*, *open triangles*), others have fast cytosolic turnover (*A*, *closed triangles*). (*B*) Slow exchange proteins (*tan*) cover the majority of the ribosome structure. Fast turnover/fast exchange r-proteins (*closed triangles* in Fig. 2*D*) are primarily at the interface between 60S (*gray rRNA*) and 40S (*black rRNA*) subunits (*red proteins*, model PDB: 4UG0).

This calculation suggests that the greater the difference between r-protein and rRNA, the faster the exchange rate. We therefore define all proteins more than two standard deviations from the rRNA rate (outside the confidence interval or CI, black symbols, Figs. 5*B*, and 5*C*) as fast exchange with the cytosolic pool (Fig. 6*A*). This separates the r-proteins into essentially three groups: 1-static; 2-rapid exchange-fast turnover, as well as rapid exchange-slow turnover proteins; and 3-proteins that switch between groups 1 and 2. Static proteins are integrated into the ribosome during initial assembly and are degraded with the assembled unit by ribophagy (Fig. 5*A*, gray circles within the square, ∼80% of the r-proteins are in this group). The fast exchange proteins (triangles in Fig. 5*A*) can be divided into two groups, fast exchange–fast turnover proteins (12%) and fast exchange–slow turnover proteins (8%).

In DR tissue, there were slight differences in the identity of the individual r-proteins but a similar percentage distribution of proteins. Proteins L3, L10, L38, L24, and S27-like were still fast exchange. This suggests that these proteins are rarely degraded by ribophagy and exchange is intrinsic to the operation of the ribosome. Proteins L19, and L34 are still at the fast end of the range but fail to exceed the confidence interval of the rRNA. This suggests that L19 and L34 are not exchanged as frequently during DR but that ribophagy plays a larger role in defining their lifetime.

Of the 14 fast exchange proteins observed in the AL ribosome, 12 were either at the interface between subunits or have significant surface exposed to the cytosol. Seven of these proteins were fast exchange in both DR and AL ribosomes. Rapid exchange proteins with fast turnover tend to be located at the interface between the 60S and 40S subunits and are involved with the structural motions of catalysis (47). For example, L24, L34, and L19 bridge the interface between the 60S and 40S ribosome (Fig. 6). They hold the subunits together and participate in the rotation between subunits during peptide bond synthesis (47). Other fast turnover proteins, L10 and L36, also have an active role in the formation of new peptide bonds and are close to the interface between the subunits (Fig. 6*B*). Protein L38 is also part of this group, but its function is less well understood and may play a role in mRNA substrate selection (48). Although there were a variety of rapid exchange proteins with slow turnover in either AL or DR, only L3 was rapid exchange under both conditions.

In AL ribosomes, L35a and slow turnover L7 were fast exchange but do not follow the structural trend of surface exposure. In the ribosome crystal structure (PDB: 4UGO), they both seem to be buried underneath large rRNA loops and slow exchange proteins. Interestingly, they are next to each other in the structure, suggesting that the rRNA may be displaced to allow exchange of these proteins simultaneously.

## Discussion

The ribosome is a multimegadalton complex of RNA and protein that synthesizes most proteins in the cell. High demand for protein synthesis reduces ribosome efficiency (21) and accuracy (2). In bacteria, damaged ribosomes can regain activity by replacement of damaged r-proteins in the assembled structure with undamaged cytosolic copies (28). Exchange of r-proteins may also be important to the functional integrity of the ribosome in eukaryotes (4), although this idea is controversial. Metabolic labeling affords a means to evaluate this hypothesis by testing directly for the replacement (turnover) of ribosomal components *in vivo*. We used metabolic deuterium incorporation rates to compare turnover of rRNA and the individual r-proteins in the assembled ribosome in mice (Fig. 1*A*). We also tested whether DR, which has previously been shown to modulate rates of ribosome biogenesis, assembly, and activity in cells (2, 49) and mice (5, 6), can impact r-protein exchange rates.

Components of the ribosome reside in two kinetically distinct pools (Fig. 1*B*), with different synthesis and degradation rates for the assembled complex and its individual constituents (40, 41). At homeostasis, the rates of opposing steps in the model (*e.g.* assembly and ribophagy) should be equal (Fig. 1*B*). Physiological and biochemical metrics of the DR effect verified that the mice used in these experiments were at homeostasis (Fig. 2) prior to metabolic labeling, similar to previous studies (5, 6). The model also assumes that free rRNA, without r-proteins, is degraded rapidly relative to the turnover of the assembled structure, as observed previously (50, 51). Under this assumption, turnover of rRNA reflects only turnover of the assembled ribosomes (Equation 3). Turnover of r-proteins in the assembled pool would depend on the kinetics of both assembly and exchange (Equation 2, *k_assemble_* + *k_add_*, note that *k_assemble_* = *k_ribophagy_* and *k_add_* = *k_remove_* due to homeostasis). We therefore calculated the exchange rate (*k_add_ and k_remove_*) as the absolute value of the difference in turnover rates of individual r-proteins (P*_i_*) in the assembled pool and the rRNA (Equation 5: Fig. 1*B*).

Assembled ribosomes isolated from the liver tissue of two animals at each of eight time points after introduction of the metabolic label were separated into two samples for analysis of rRNA and r-protein turnover (Fig. 1*A*). The amount of new rRNA increased exponentially with time (Fig. 4) and could be modeled by assumption of a single pool. There was a statistically insignificant increase in rRNA turnover increased in the DR tissue [(11.1 ± 1.7) % Day^−1^] relative to control with no dietary restriction (AL) tissue [(10.1 ± 1.2) % Day^−1^]. In DR, although fewer ribosomes were actively translating protein (Fig. 3), the total number was not different between AL and DR tissue (via qPCR, Fig. S1*B*). The results imply that, on average, ribosomes were less active and had a slightly shorter lifetime (6 days) in tissues of DR animals than in those from the AL animals (7 days). The measurements of protein turnover within the assembled ribosome and the observed proteome support these results.

Turnover rates of the individual ribosomal proteins (r-proteins) were resolved by monitoring incorporation of deuterium into multiple tryptic peptides for each protein along the labeling time-course (Fig. 5*A*), as previously described (6, 36, 46). Within the set of 71 (out of 80 total) integral r-proteins monitored, a range of 4–28% per day was observed (Supplemental Table). Most r-proteins turn over at rates that are similar to (within two standard deviations of) that of the rRNA (Figs. 5*B*-5*D* gray symbols), implying that this large group of r-proteins and the rRNA are replaced together as a unit (*i.e.* complete degradation of the complex). Indeed, the average rate for the r-proteins (10.2% in AL and 11.3% in DR) matched the ribophagy rate (10.1% in AL and 11.1% in DR) remarkably well. Comparison of resolved turnover rates of the individual r-protein components (Fig. 5*D*) makes it apparent that the small DR-dependent increase in rRNA turnover (Fig. 4) is significant (*p* < 0.0005). Overall, ∼80% of r-proteins, had individual turnover rates that match the ribophagy rate (within two standard deviations of the rRNA). This agrees with earlier studies of average rRNA and r-protein turnover (52).

The increased rate of ribophagy in DR tissues was surprising. In agreement with previous studies (5, 6, 53, 54), we observed that DR slows cell proliferation (Fig. 2*B*) and protein synthesis (Fig. 3). Interestingly, the cellular half-life (169 days in AL) is 25 times greater that the ribosome half-life in AL. In DR, the ribosome turnover is accelerated relative to cell proliferations, becoming 37 times greater. The two-dimensional comparison suggests that the rate of ribophagy, specifically, doubles relative to the rate of other processes like mitochondria-specific degradation or cell division. The up-regulation of ribophagy during DR may explain the previous observation that increased autophagy and lower protein synthesis rates work together to improve cellular fitness and whole organism lifespan (2, 5, 6, 9, 30, 31).

Although the turnover rate of r-proteins averaged over the entire set reflects the ribophagy rate (as previously reported), a small but intriguing set of r-proteins have significantly different turnover rates (Fig. 5*B*–5*D*, black symbols). Importantly, some of the proteins with unusually fast or slow turnover are the same in both AL and DR tissues (Fig. 2*D*). The parallel behavior of these proteins in both dietary cohorts suggests that the difference in their turnover rates might be intrinsic and functionally relevant. The difference in turnover may be due to the free pool r-protein turnover rate (Fig. 1*B*), which is independent of the ribophagy rate. The free pool turnover rate is difficult to measure directly since it is very low concentration for each r-protein (40) and homogenizing the tissue is likely to break assembled ribosomes, contaminating the free pool. However, as shown in Equation 5, the exchange rate of each protein can be calculated without direct measurement of the free pool. The proteins that are exchanged rapidly out of the assembled structure would have turnover rates defined by the free pool. Therefore, fast exchange between the assembled and free pool could explain outliers at both the fast and slow end of the turnover range (Fig. 5).

When we compared the calculated exchange rates (Fig. 6*A*) against the ribosome structure, we saw that the fast exchange and fast turnover r-proteins are predominately located at the interface between the 60S and 40S subunits (Fig. 6*B*). This region is known to undergo significant movement during the catalytic activity of the ribosome (47). There are at least two possible hypotheses to explain why ribosomal maintenance would include fast exchange of these proteins. First, proteins at this location may be more prone to damage and therefore exchange more rapidly. Second, damage of these proteins dramatically reduces formation of the 80S ribosome and ensures that there is a longer exchange period. Three of these proteins (L19, L24, and L34) are structurally important, acting like long fingers to secure the 40S to the 60S subunit (Fig. 6). The other members of this subgroup (L10, L36A, and L38) are also localized in the interface, either on the beak or directly across from it. Breaking the 80S down to the 40S and 60S could facilitate exchange of these proteins due to lost surface interactions and greater access to the cytosol.

Cytosolic RPL36a and L10 are critical to formation of the P-site during assembly and guide the structural rotation necessary to form each peptide bond (55). Dysfunction of these and other fast exchange proteins is associated with disease (4, 47); therefore, exchange may represent an important method to maintain ribosomal quality. Greater exchange of these proteins in AL tissue relative to the ribophagy rate may indicate that damage to these specific proteins occurs prior to ribophagy.

**Fig. 7.**
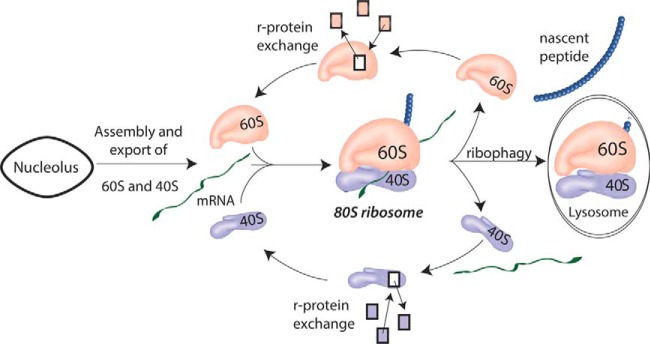
***In vivo* ribosome maintenance requires ribophagy and r-protein exchange.** Each day, ∼10% of the ribosomal pool is replaced via assembly of new ribosomes and ribophagy. During the lifetime of the assembled ribosomal structure, ribosome protein exchange occurs primarily when the ribosome disassociates to its individual subunits. This exchange may be a fast, low cost, method to repair and modify ribosomes. Cellular energetics and demand for protein synthesis may modulate the relative contribution of ribophagy *versus* exchange in response to stalled or damaged ribosomes.

One interpretation of these results is that ribophagy and r-protein exchange are both used to maintain the active pool of ribosomes (Fig. 7). Simplistically, the high synthetic demand and longer lifespan for individual ribosomes observed in AL tissue might result in accumulation of damaged ribosomes, as only a few selected proteins can be repaired. Slower cell proliferation in DR suggests that, because there is less dilution into new cells, protein degradation is up-regulated to match synthesis (6, 18). Lower synthetic demand with an accompanying increase in ribophagy, would allow for more extensive turnover of the assembled ribosome pool. Better maintenance of the ribosome might lead to higher quality nascent peptides and improved accuracy (2) and efficiency (21) relative to AL tissue. This provides an attractive interpretation for the frequently observed connection between lower rates of protein synthesis, increased autophagy, and improved protein homeostasis and longevity (5, 6, 8, 9).

It has been observed that the RQC complex is required to dissociate stalled 80S ribosomes (22). Our data suggest that ribosomes exchange components most rapidly when dissociated into subunits, potentially after the RQC has dissociated the complex. These results raise several interesting questions about mechanisms for maintenance. Is there cross talk between RQC activity and ribosome component turnover? What factors control ribosome component exchange? We assume a passive model, but could the RQC coordinate active exchange? Proteins L35a and L7 are fast exchange in AL tissue but are buried within the rRNA. These proteins may require outside assistance to facilitate exchange. Finally, does r-protein exchange improve the quality of nascent peptides? Initial results suggest that reducing global protein synthesis may improve protein quality (54). Further investigation is needed to confirm whether ribosomal maintenance is a mechanistic link explaining how lower protein synthesis burdens are connected to improved protein homeostasis and lifespan.

In conclusion, this work uses a generally applicable strategy for investigating cellular maintenance of the proteome, including multiprotein and ribonuclear structures. Here, we show that exchange of protein components and degradation of the entire ribosome are important maintenance strategies. Our results suggest a mechanism wherein dissociation of 60S and 40S subunits promotes *in vivo* r-protein exchange. We find that dietary signals can change both ribophagy and r-protein exchange rates. Future work testing biological models that modulate the RQC activity and/or mechanistic target of rapamycin (mTOR) signaling will test whether this is mechanism is generally applicable. Also, linking changes in longevity to the rates of protein synthesis and autophagy as we have done, may help identify mechanisms of aging.

## Supplementary Material

Supplemental Data

## Footnotes

^1^ The abbreviations used are:

Proteostasis: protein homeostasis
rRNA: ribosomal RNA
r-proteins: ribosomal proteins
DR: dietary restriction
AL: *Ad Libitium* or unrestricted diet
RQC: ribosome quality control
ribophagy: directed autophagy of ribosomes
M0: mono isotopic mass spectral peak
M1: mass spectral peak with 1 extra neutron
M2: mass spectral peak with 2 extra neutrons
RCR: respiratory control ratio
mTOR: mechanistic target of rapamycin.
