# Supplementary material for: Mechanisms of In Vivo Ribosome Maintenance Change in Response to Nutrient Signals
Source: Mol Cell Proteomics. 2016 Dec 8;16(2):243–54. doi: 10.1074/mcp.M116.063255 (PMC5294211; doi:10.1074/mcp.M116.063255)
Supplement: Supplemental Data [file supp_16_2_243__index.html]

Mechanisms of in vivo ribosome maintenance change in response to nutrient signals — Mechanisms of In Vivo Ribosome Maintenance Change in Response to Nutrient Signals — In Vivo Ribosome Maintenance — Supplemental Data 

# Mechanisms of *In Vivo* Ribosome Maintenance Change in Response to Nutrient Signals

## Supplemental Data

- DR assembled graphs (.pdf, 917 KB) - This file contains the fitting curves used to calculate the turnover rates for each of the ribosomal proteins with sufficient high quality data in this pool
- DR total graphs (.pdf, 1.0 MB) - This file contains the fitting curves used to calculate the turnover rates for each of the ribosomal proteins with sufficient high quality data in this pool
- AL assembled graphs (.pdf, 1.0 MB) - This file contains the fitting curves used to calculate the turnover rates for each of the ribosomal proteins with sufficient high quality data in this pool
- AL total graphs (.pdf, 827 KB) - This file contains the fitting curves used to calculate the turnover rates for each of the ribosomal proteins with sufficient high quality data in this pool
- Table identifying homologus accession numbers (.xlsx, 33 KB) - Sequence analysis of the homologus protein annotations
- AL assembled peptide level data (.xlsx, 2.8 MB) - all of the extracted isotope information for the unfiltered peptides
- AL total peptide level data (.xlsx, 2.0 MB) - all of the extracted isotope information for the unfiltered peptides
- DR assembled peptides (.xlsx, 1.9 MB) - all of the extracted isotope information for the unfiltered peptides
- DR total peptides (.xlsx, 2.8 MB) - all of the extracted isotope information for the unfiltered peptides
- Supplemental Table of protein kinetics (.xlsx, 75 KB) - Summary of the protein level information from each ribosomal protien in each of the kinetic pools, as well as the MSMS scores for the peptides used in calculating these rates.
- Supplemental Information Summary (.pdf, 348 KB) - Supplemental figures and legends for the tables and graphs as referenced in the main body of the text
