# Supplementary material for: Mechanisms of In Vivo Ribosome Maintenance Change in Response to Nutrient Signals
Source: Mol Cell Proteomics. 2016 Dec 8;16(2):243–54. doi: 10.1074/mcp.M116.063255 (PMC5294211; doi:10.1074/mcp.M116.063255)
Supplement: Supplemental Data [file 10.1074_M116.063255_mcp.M116.063255-1.pdf]

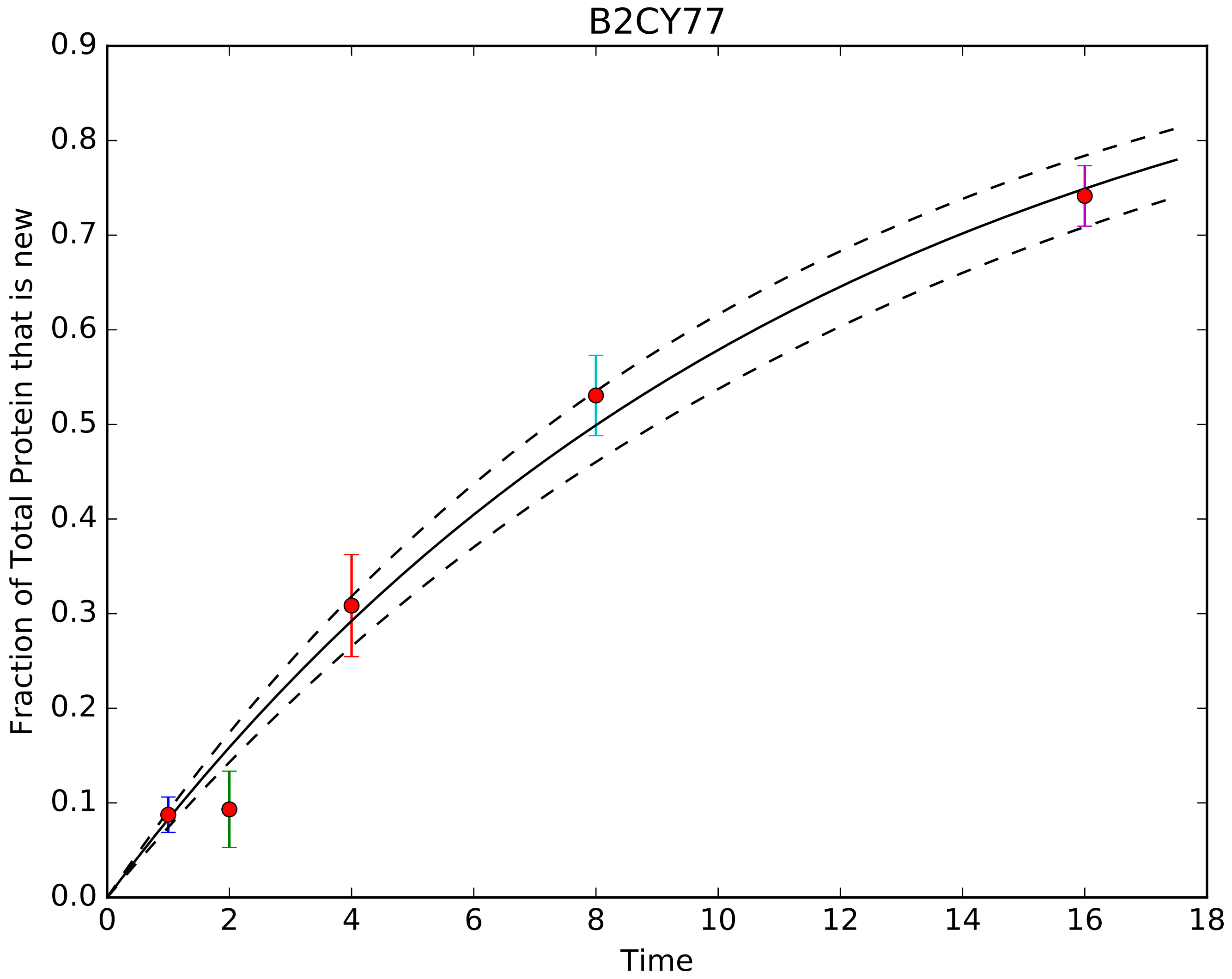

B2CY77
Fraction of Total Protein that is new
Time
0.0
0.1
0.2
0.3
0.4
0.5
0.6
0.7
0.8
0.9
0
2
4
6
8
10
12
14
16
18

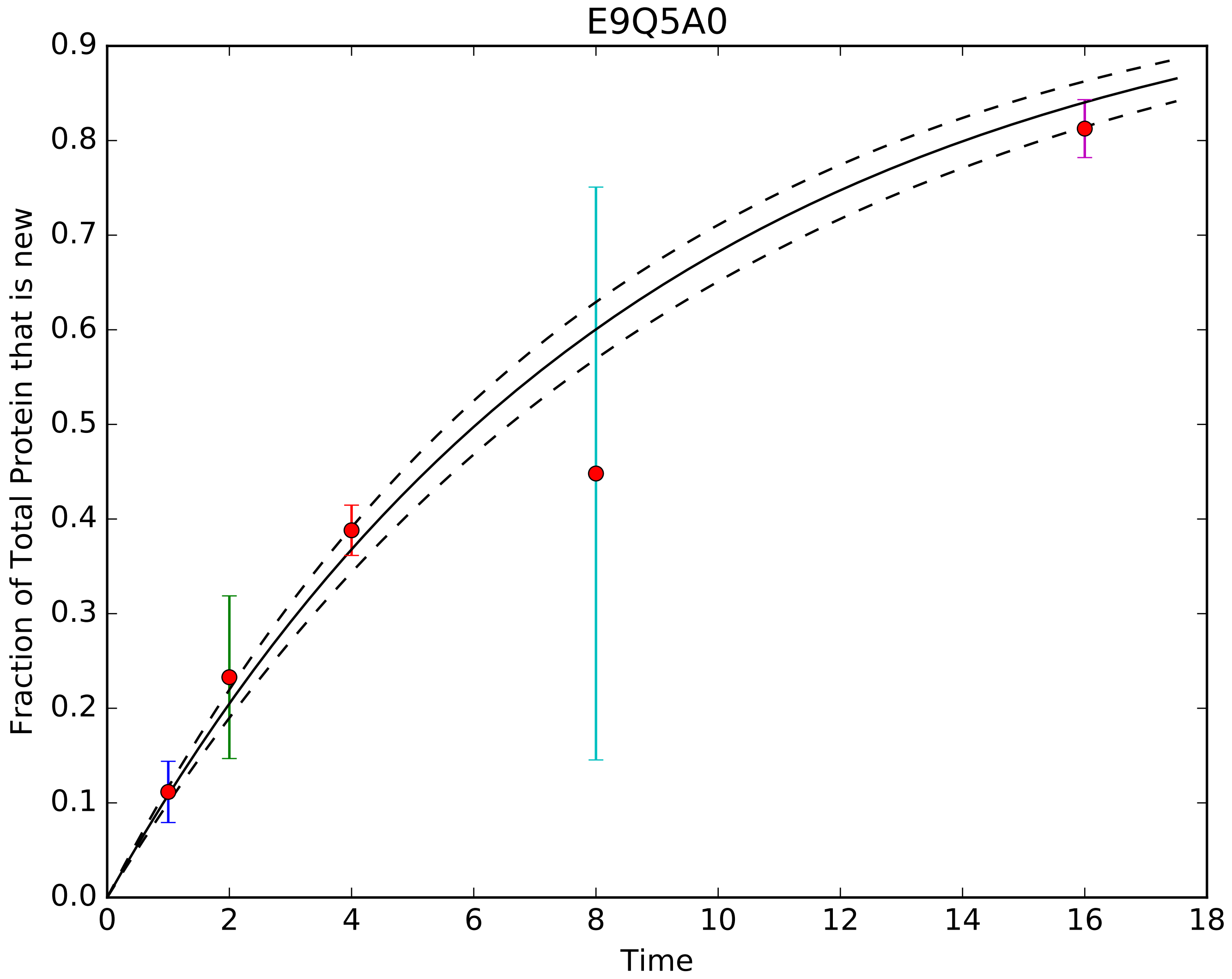
E9Q5A0
Fraction of Total Protein that is new
Time
0.0
0.1
0.2
0.3
0.4
0.5
0.6
0.7
0.8
0.9
0
2
4
6
8
10
12
14
16
18

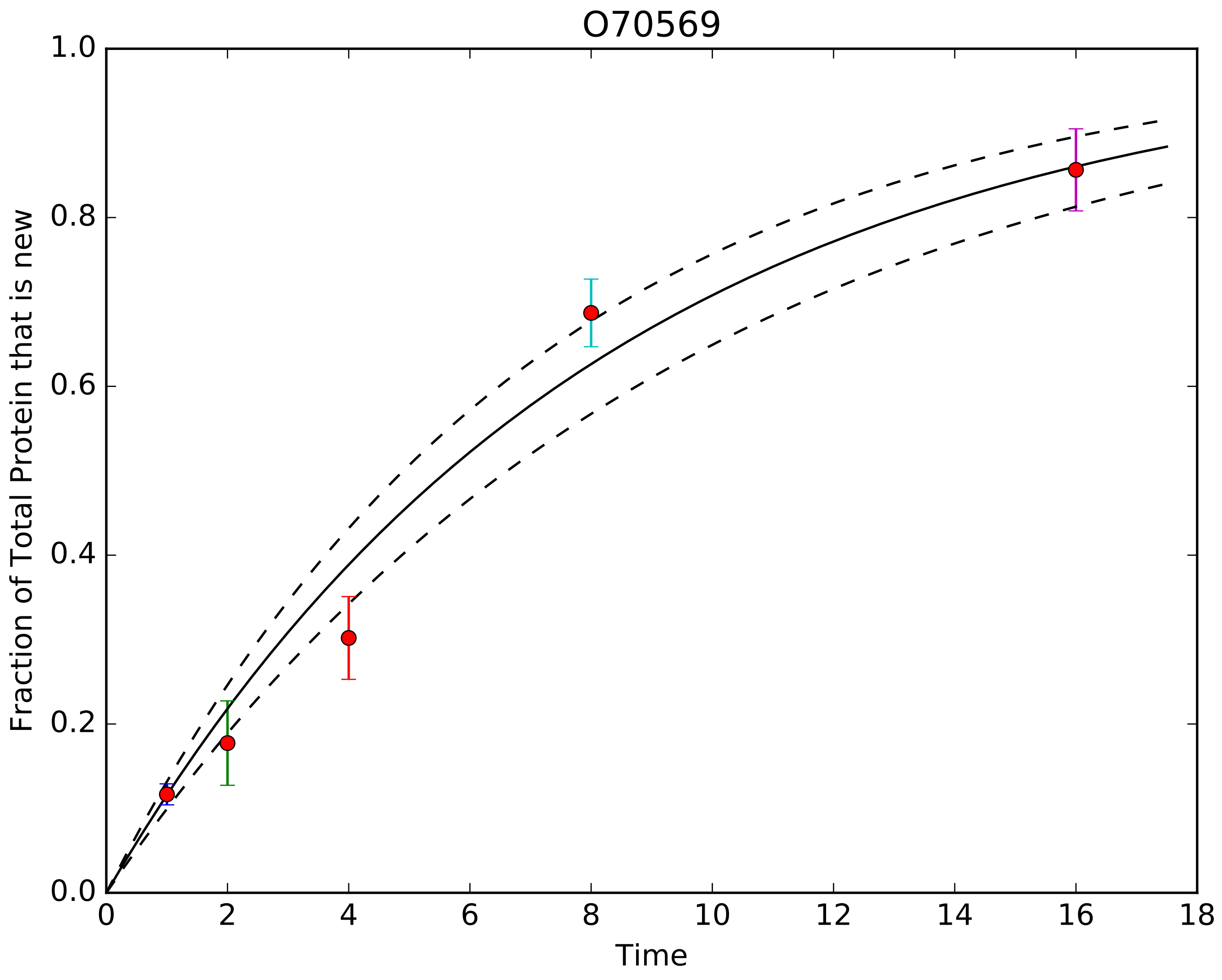

O70569
Fraction of Total Protein that is new
Time
0.0
0.2
0.4
0.6
0.8
1.0
0
2
4
6
8
10
12
14
16
18

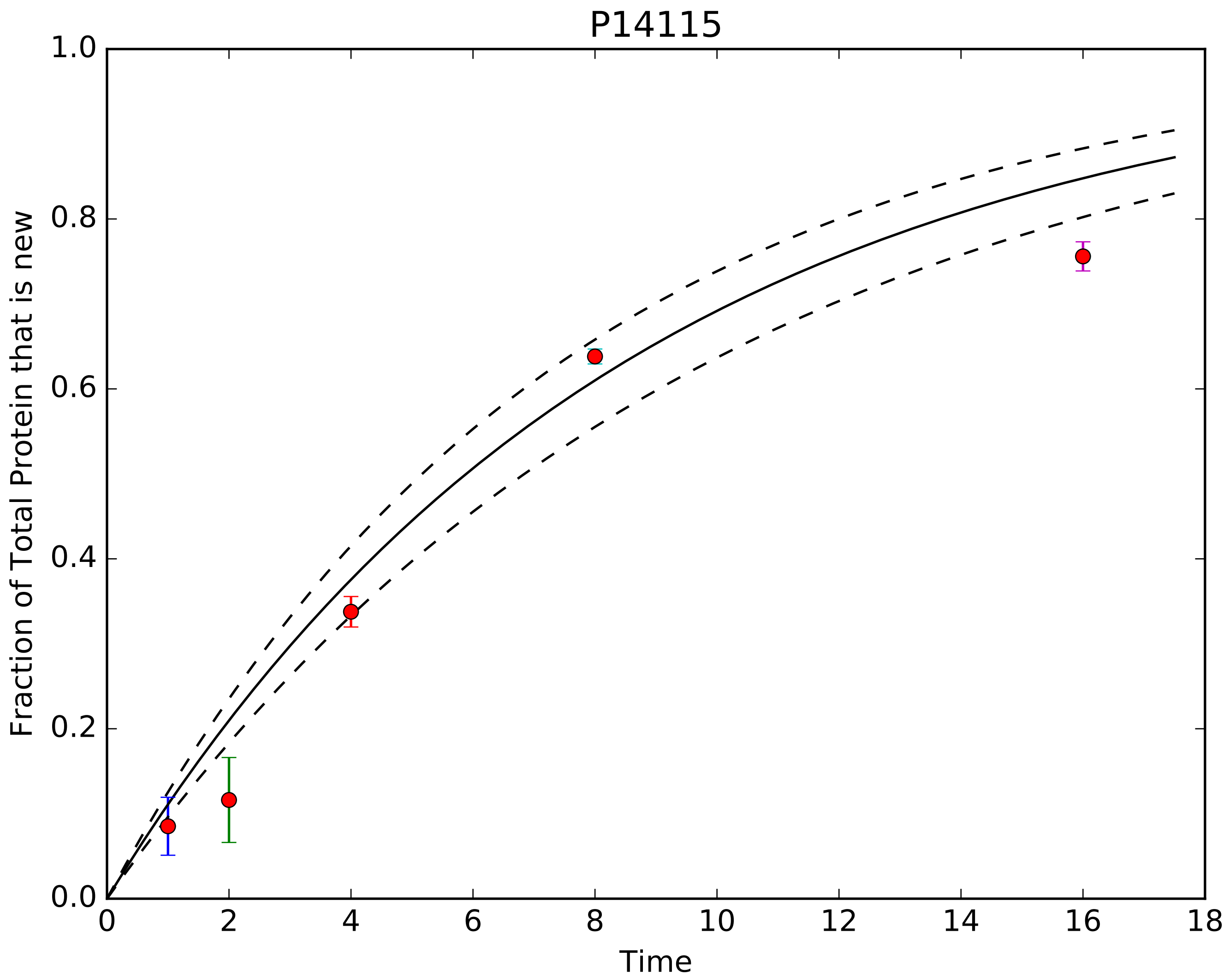

P14115
Fraction of Total Protein that is new
Time
0.0
0.2
0.4
0.6
0.8
1.0
0
2
4
6
8
10
12
14
16
18

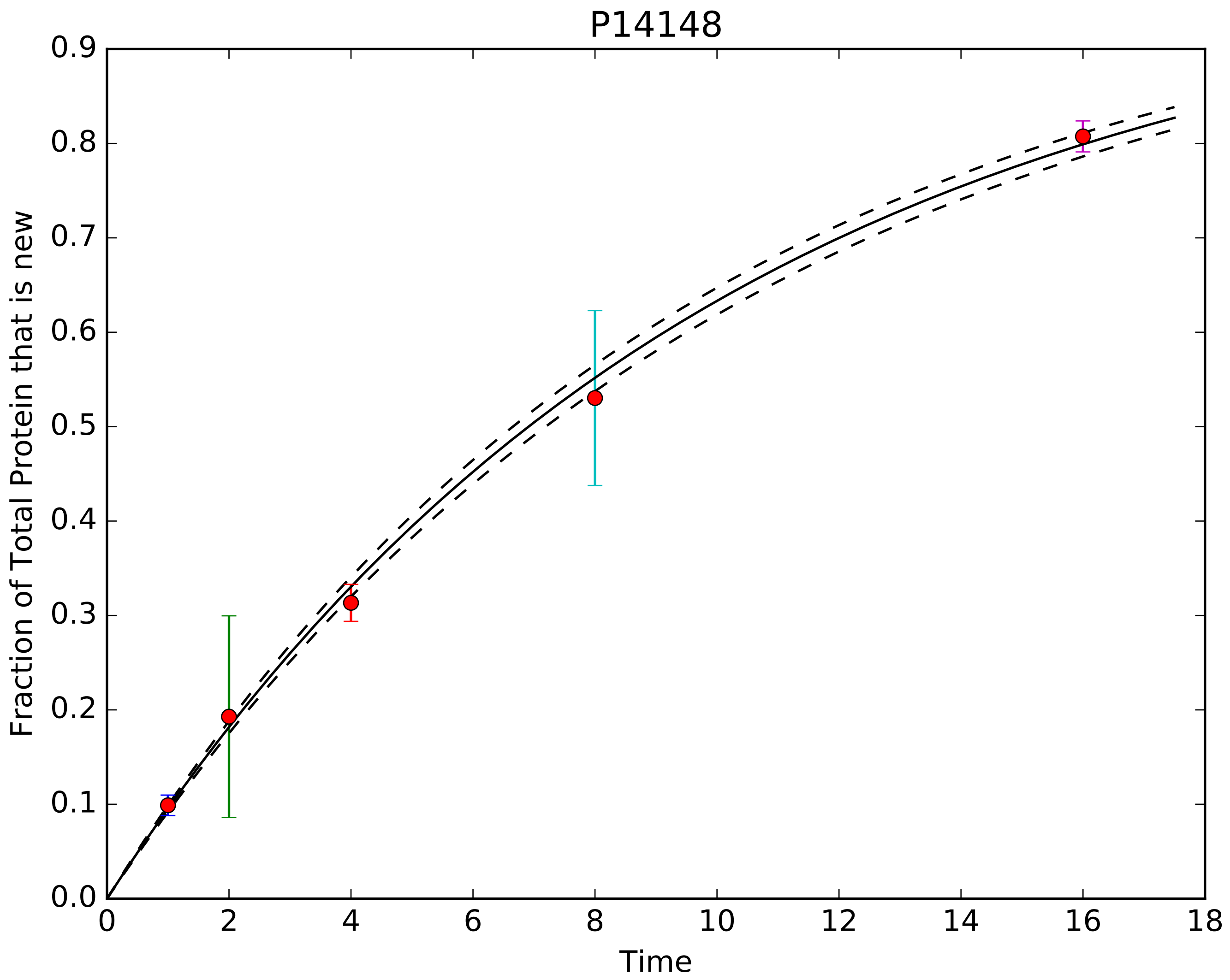

P14148
Fraction of Total Protein that is new
Time
0.0
0.1
0.2
0.3
0.4
0.5
0.6
0.7
0.8
0.9
0
2
4
6
8
10
12
14
16
18

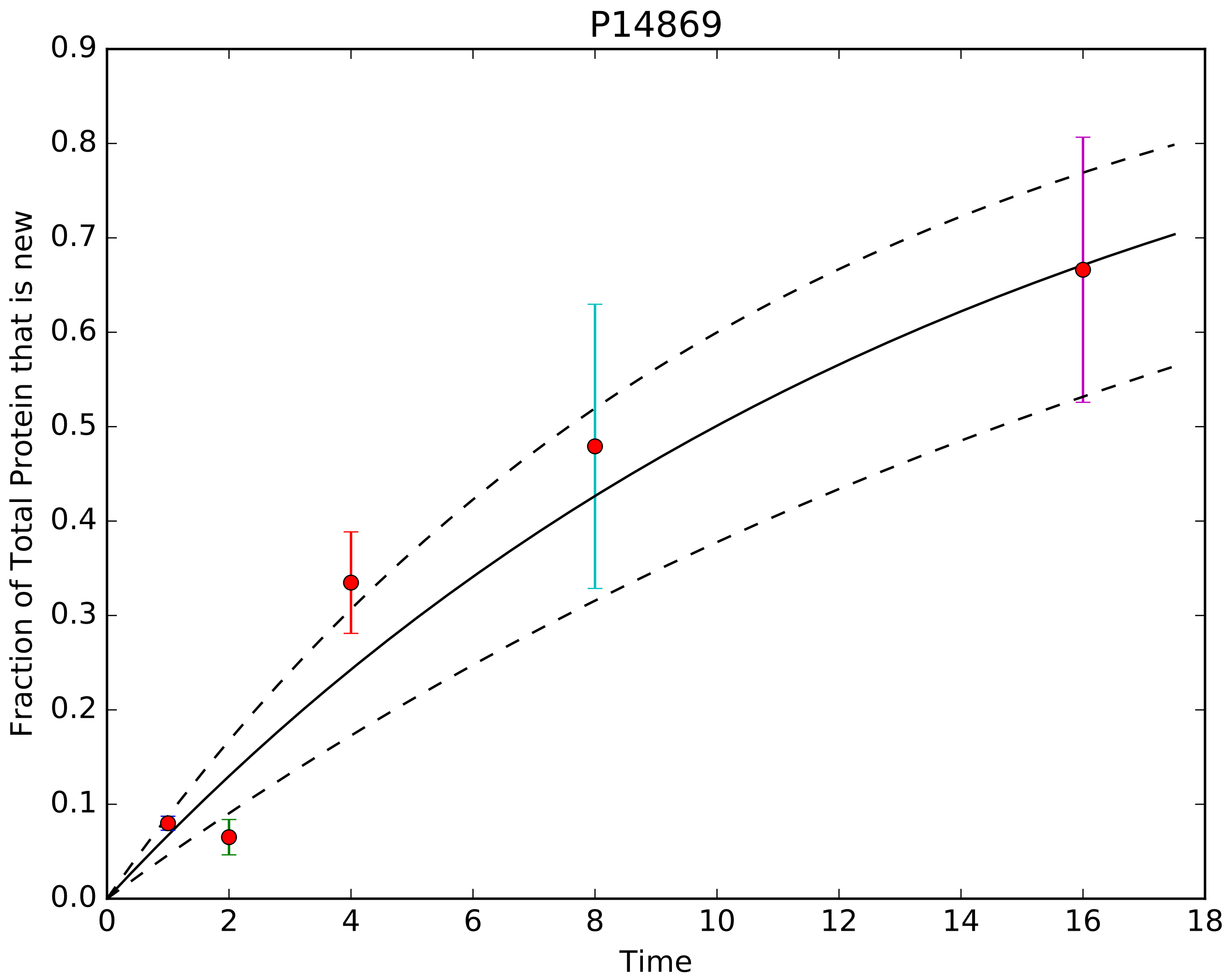
P14869
Fraction of Total Protein that is new
Time
0.0
0.1
0.2
0.3
0.4
0.5
0.6
0.7
0.8
0.9
0
2
4
6
8
10
12
14
16
18

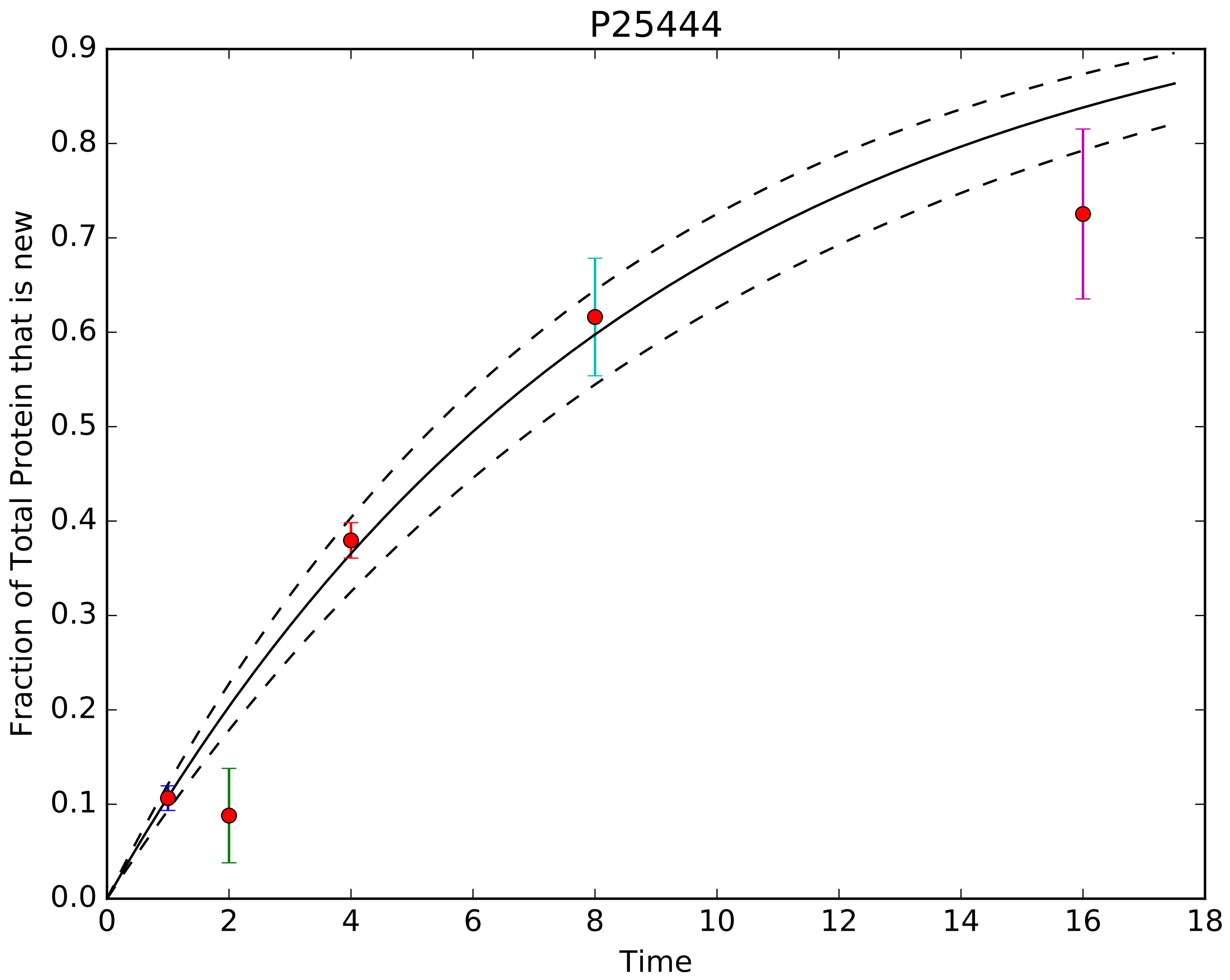
P25444
Fraction of Total Protein that is new
Time
0.0
0.1
0.2
0.3
0.4
0.5
0.6
0.7
0.8
0.9
0
2
4
6
8
10
12
14
16
18

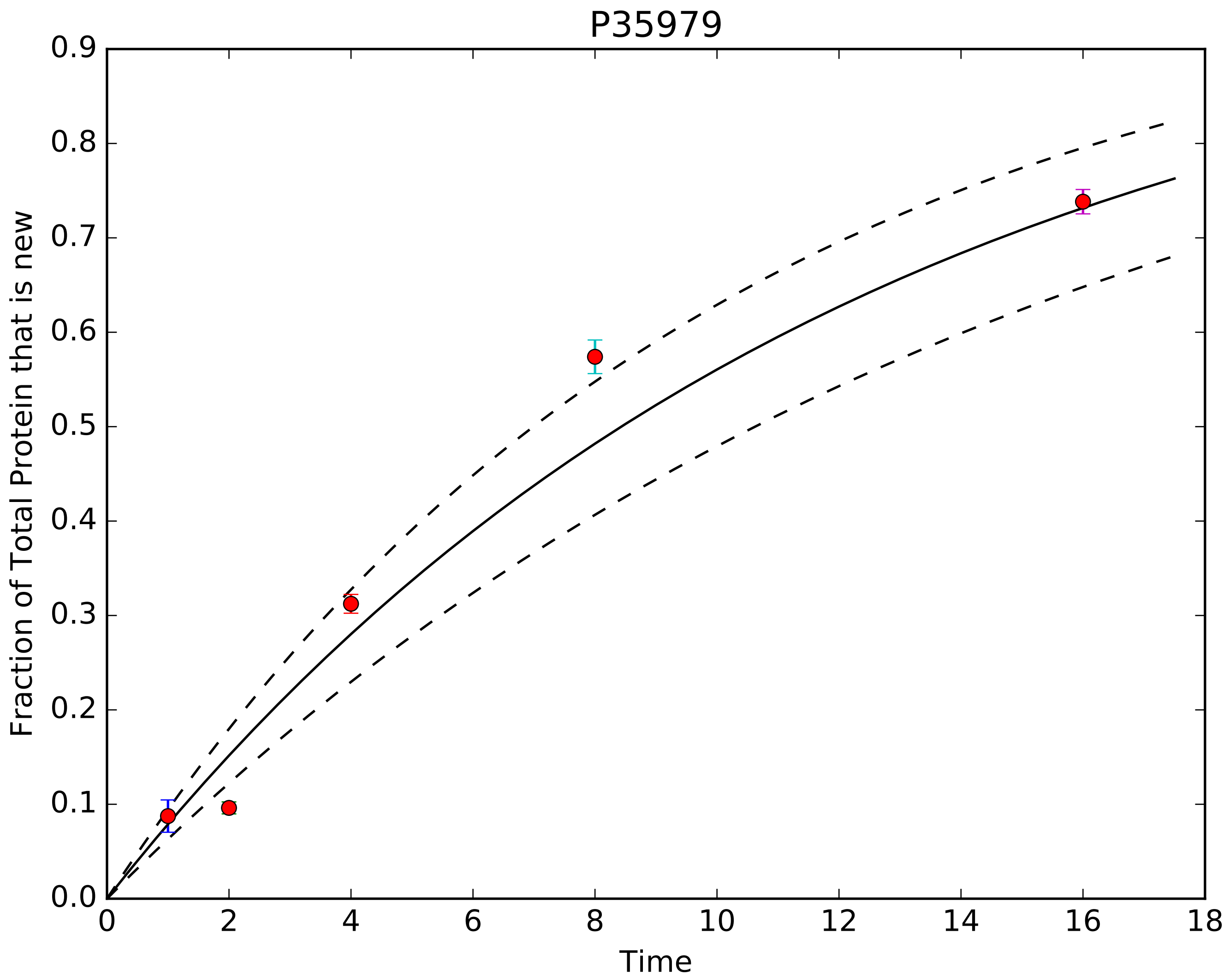
P35979
Fraction of Total Protein that is new
Time
0.0
0.1
0.2
0.3
0.4
0.5
0.6
0.7
0.8
0.9
0
2
4
6
8
10
12
14
16
18

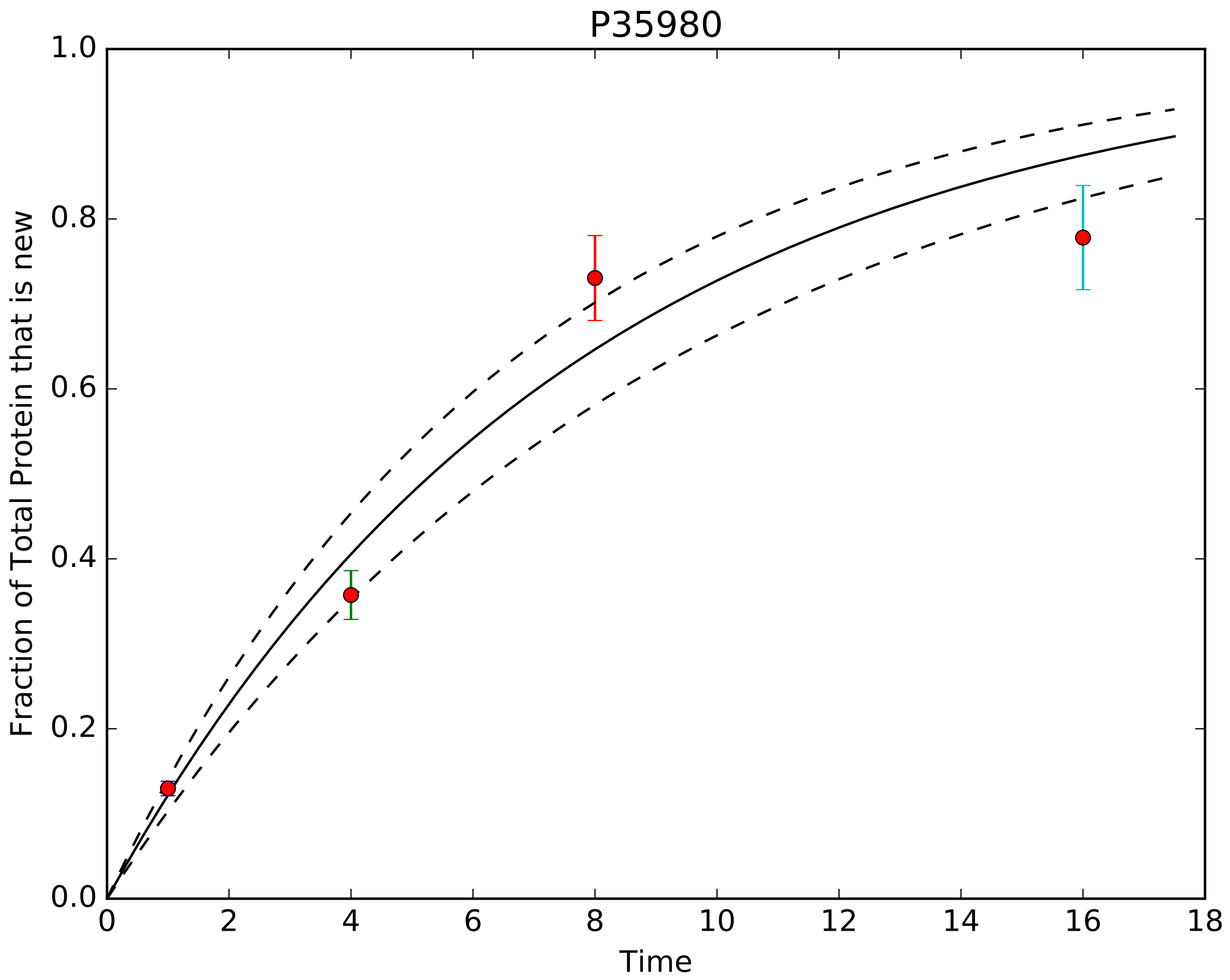
P35980
Fraction of Total Protein that is new
Time
0.0
0.2
0.4
0.6
0.8
1.0
0
2
4
6
8
10
12
14
16
18

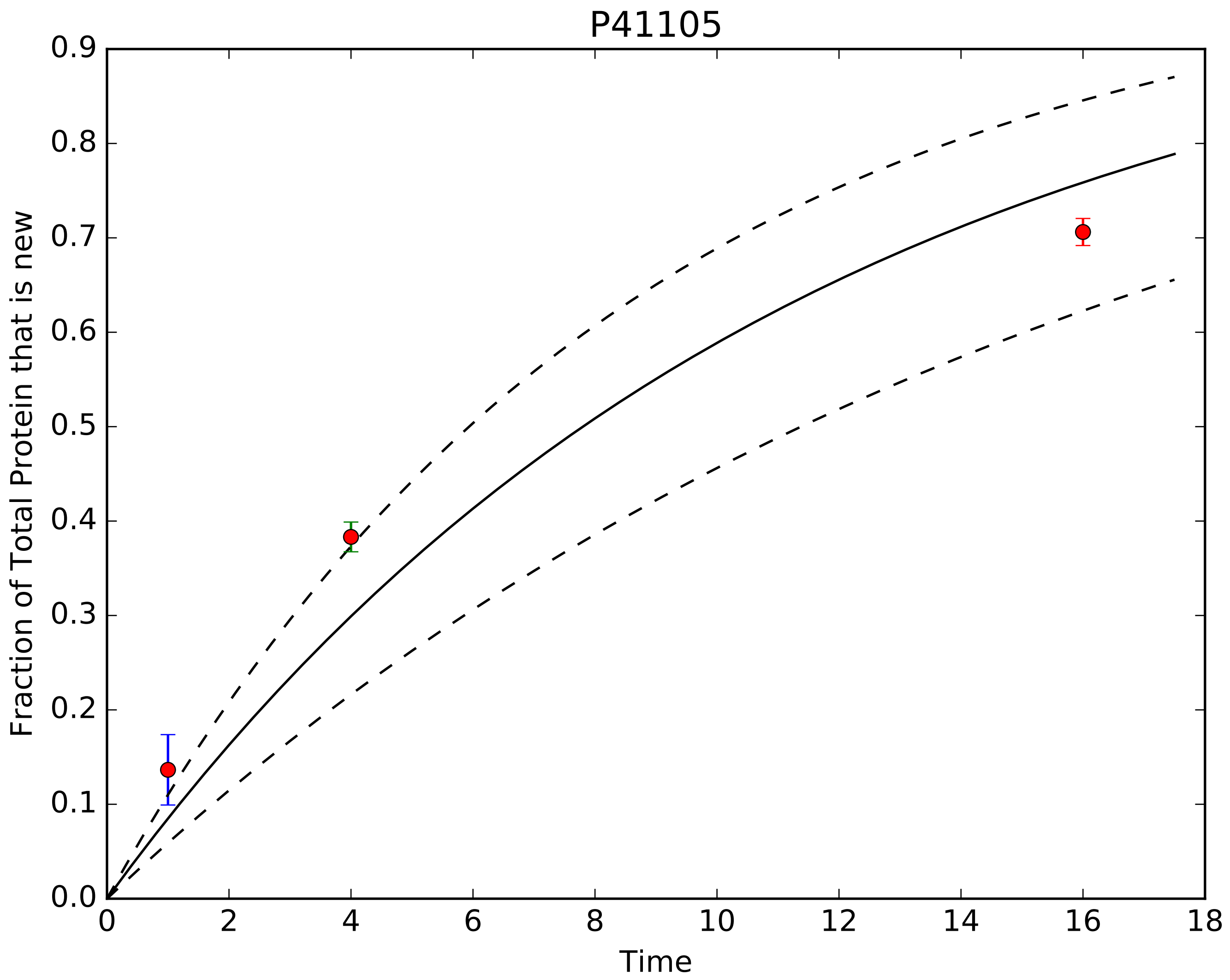
P41105
Fraction of Total Protein that is new
Time
0.0
0.1
0.2
0.3
0.4
0.5
0.6
0.7
0.8
0.9
0
2
4
6
8
10
12
14
16
18

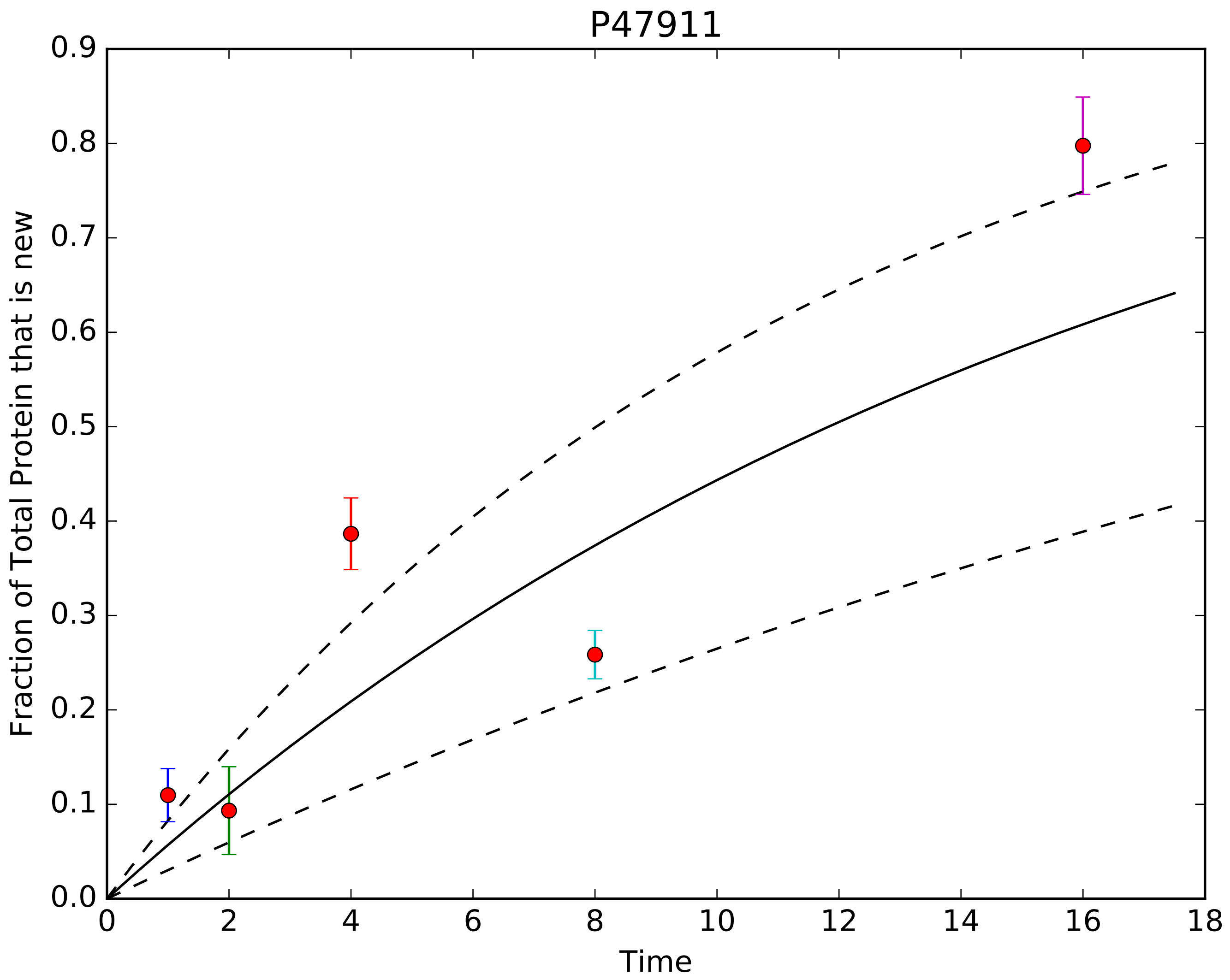
P47911
Fraction of Total Protein that is new
Time
0.0
0.1
0.2
0.3
0.4
0.5
0.6
0.7
0.8
0.9
0
2
4
6
8
10
12
14
16
18

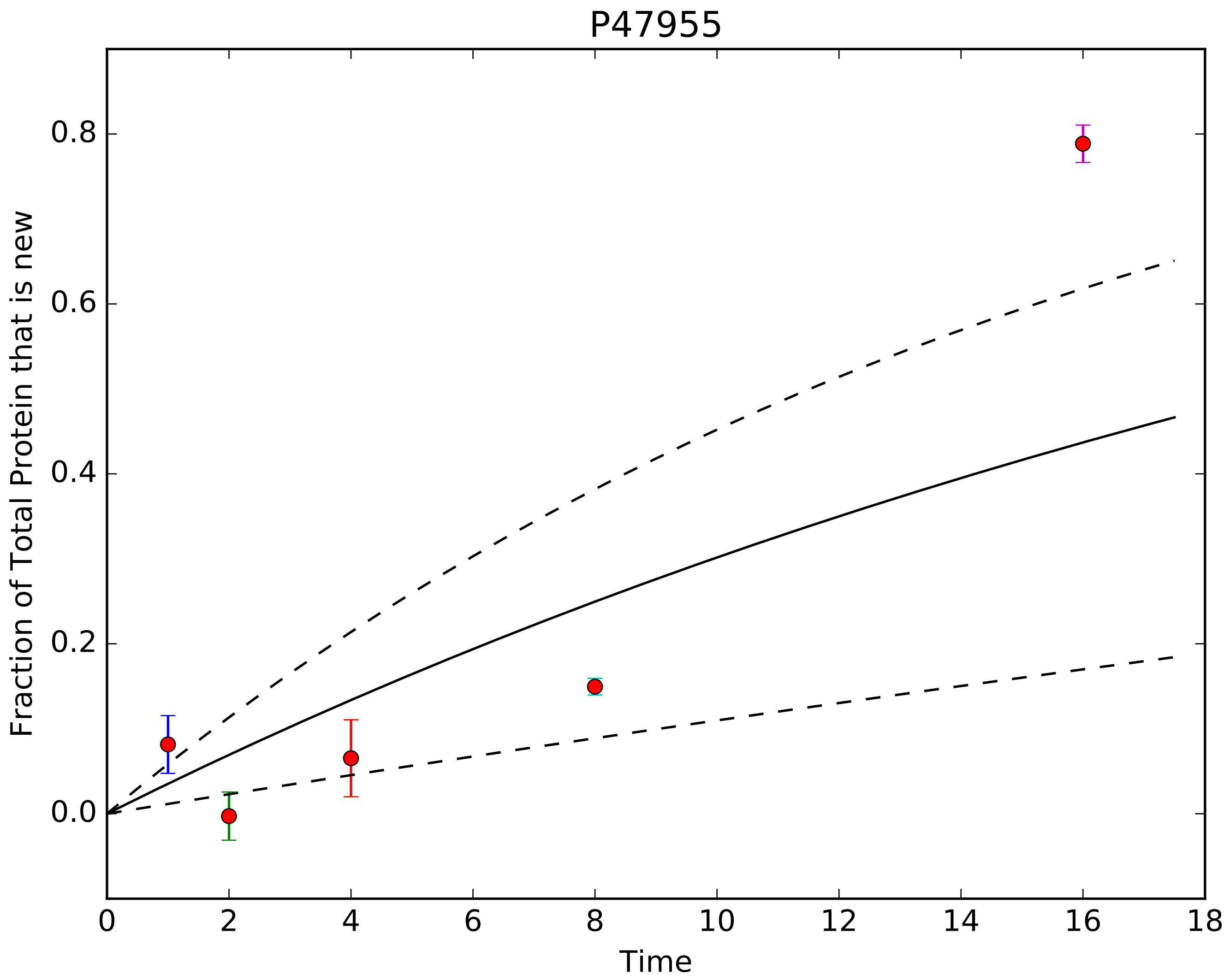
P47955
Fraction of Total Protein that is new
Time
0.0
0.2
0.4
0.6
0.8
0
2
4
6
8
10
12
14
16
18

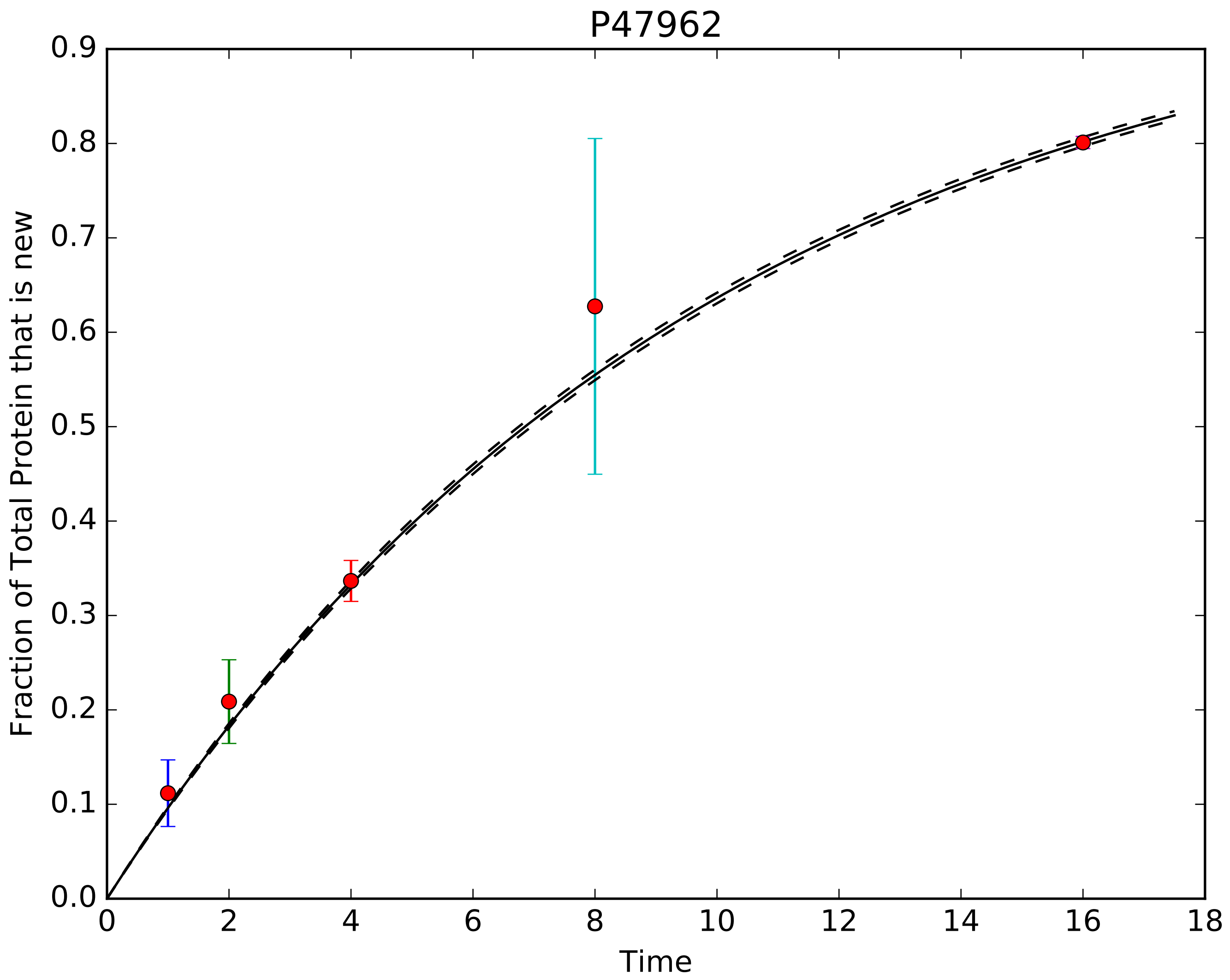
P47962
Fraction of Total Protein that is new
Time
0.0
0.1
0.2
0.3
0.4
0.5
0.6
0.7
0.8
0.9
0
2
4
6
8
10
12
14
16
18

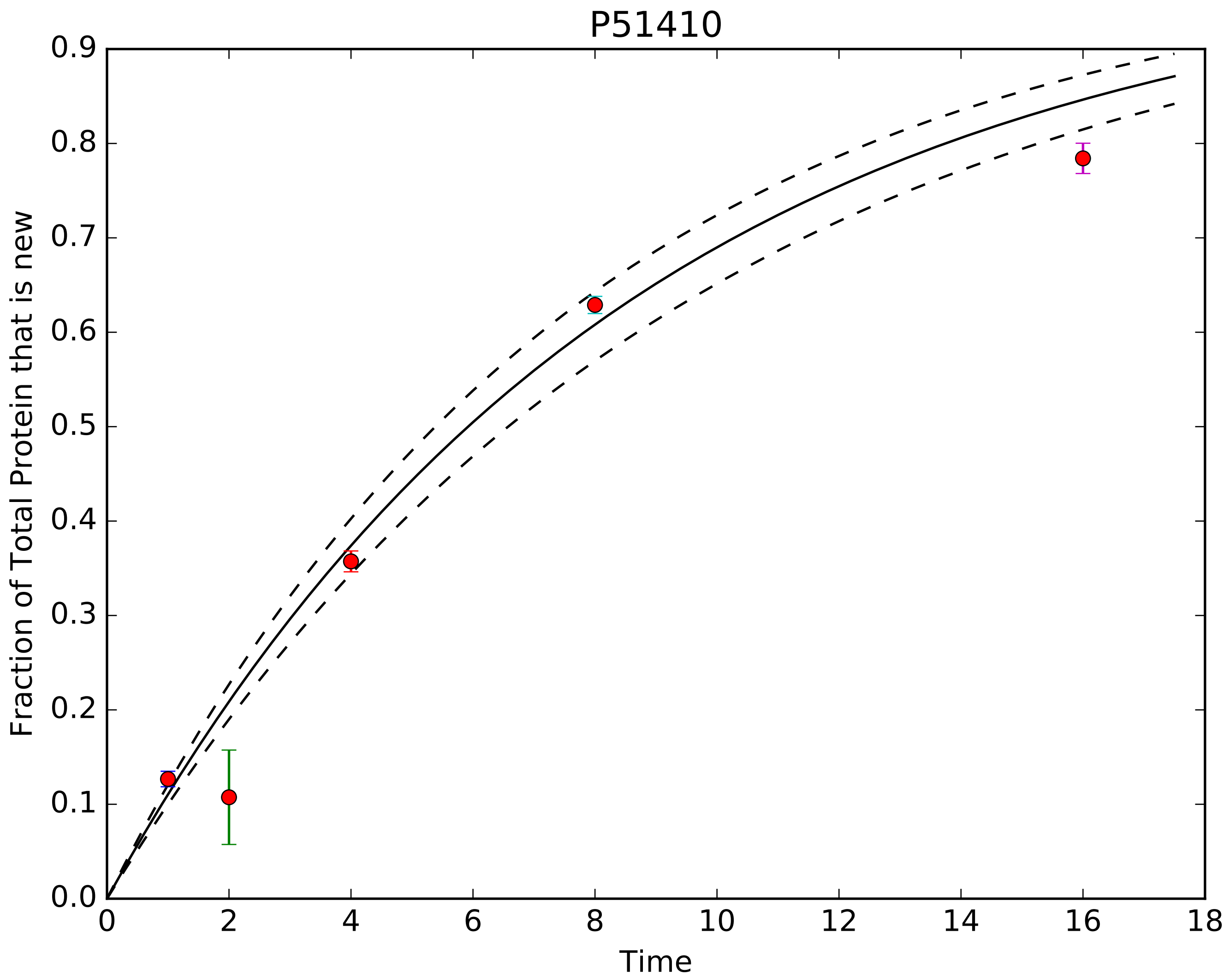
P51410
Fraction of Total Protein that is new
Time
0.0
0.1
0.2
0.3
0.4
0.5
0.6
0.7
0.8
0.9
0
2
4
6
8
10
12
14
16
18

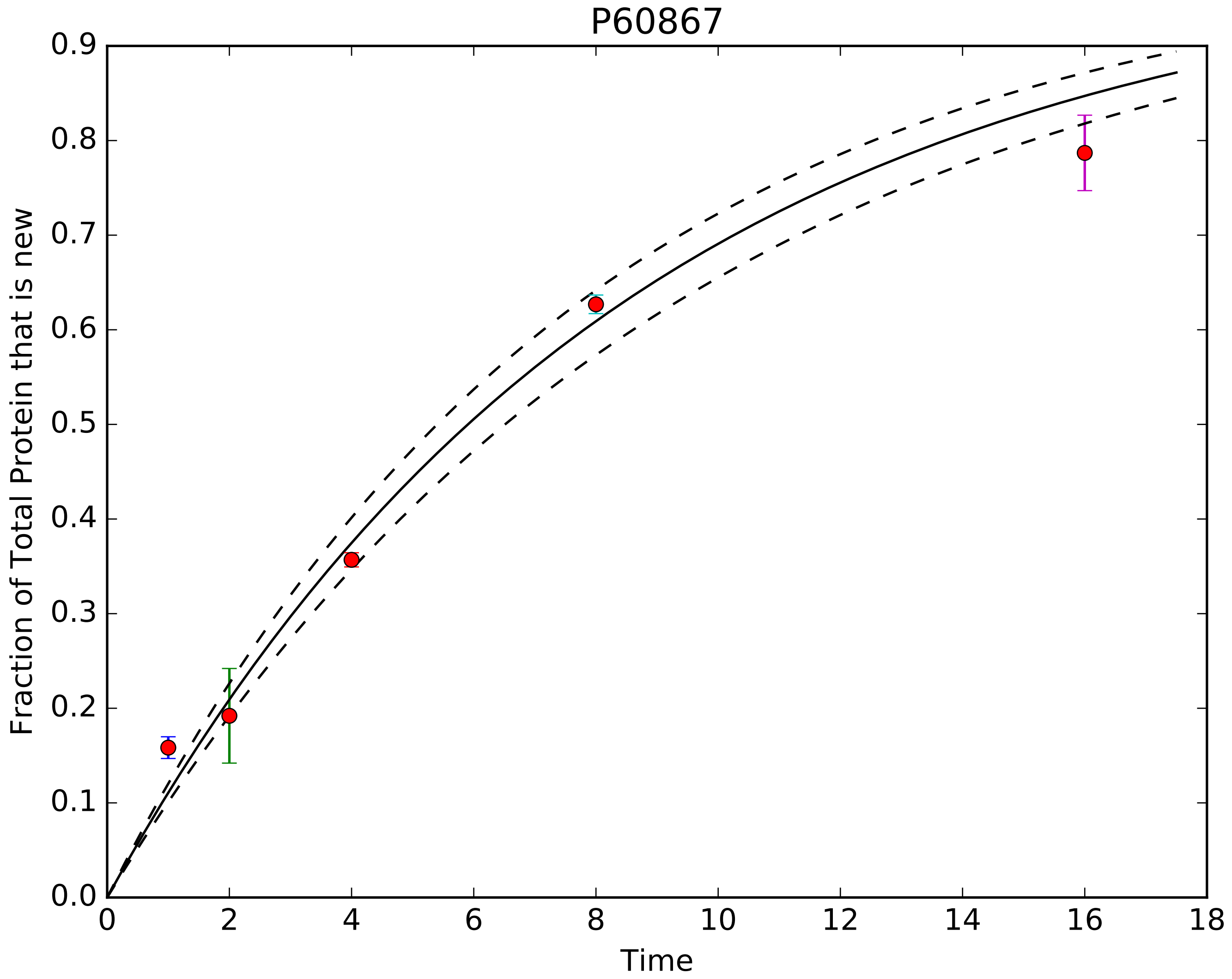
P60867
Fraction of Total Protein that is new
Time
0.0
0.1
0.2
0.3
0.4
0.5
0.6
0.7
0.8
0.9
0
2
4
6
8
10
12
14
16
18

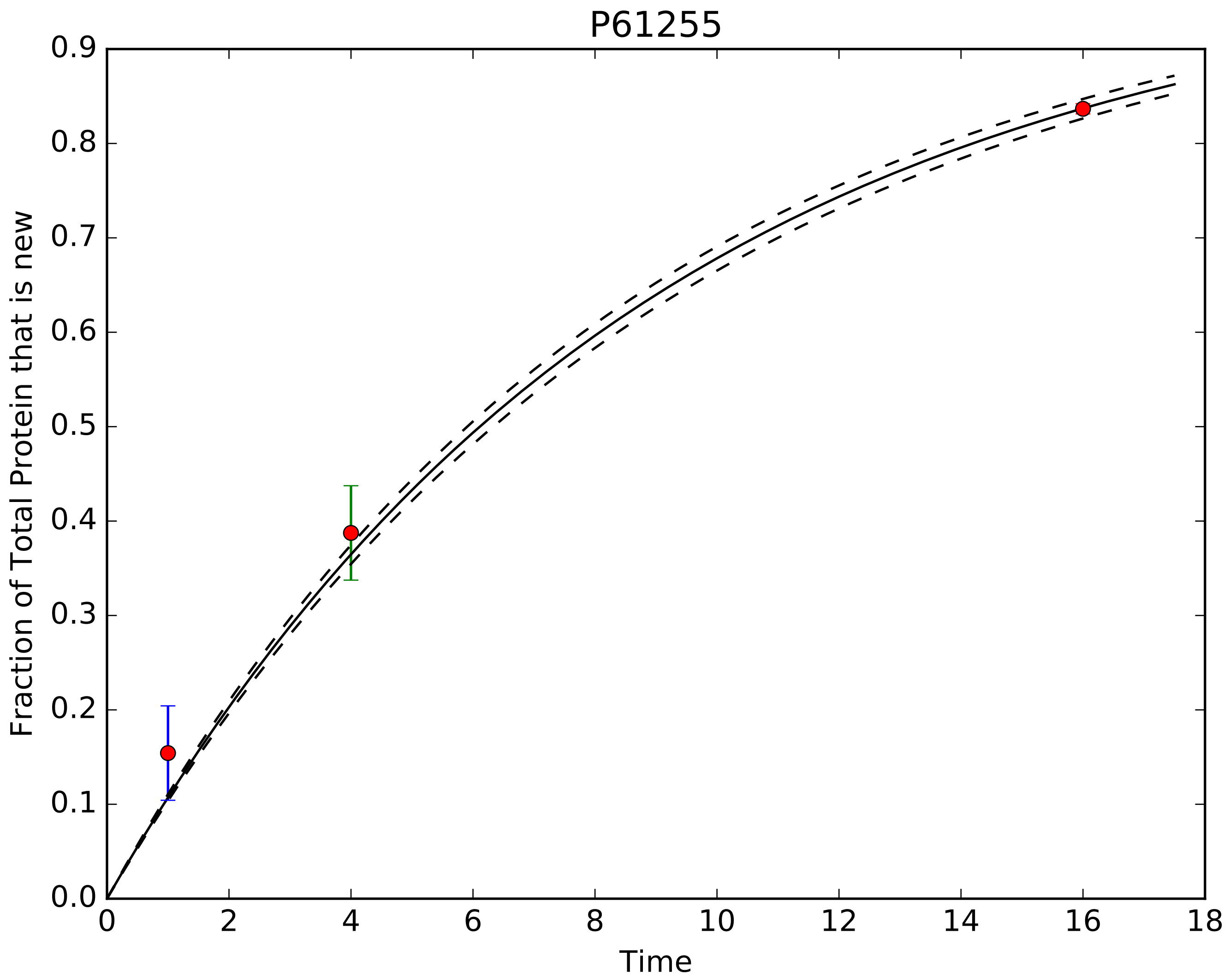

P61255
Fraction of Total Protein that is new
Time
0.0
0.1
0.2
0.3
0.4
0.5
0.6
0.7
0.8
0.9
0
2
4
6
8
10
12
14
16
18

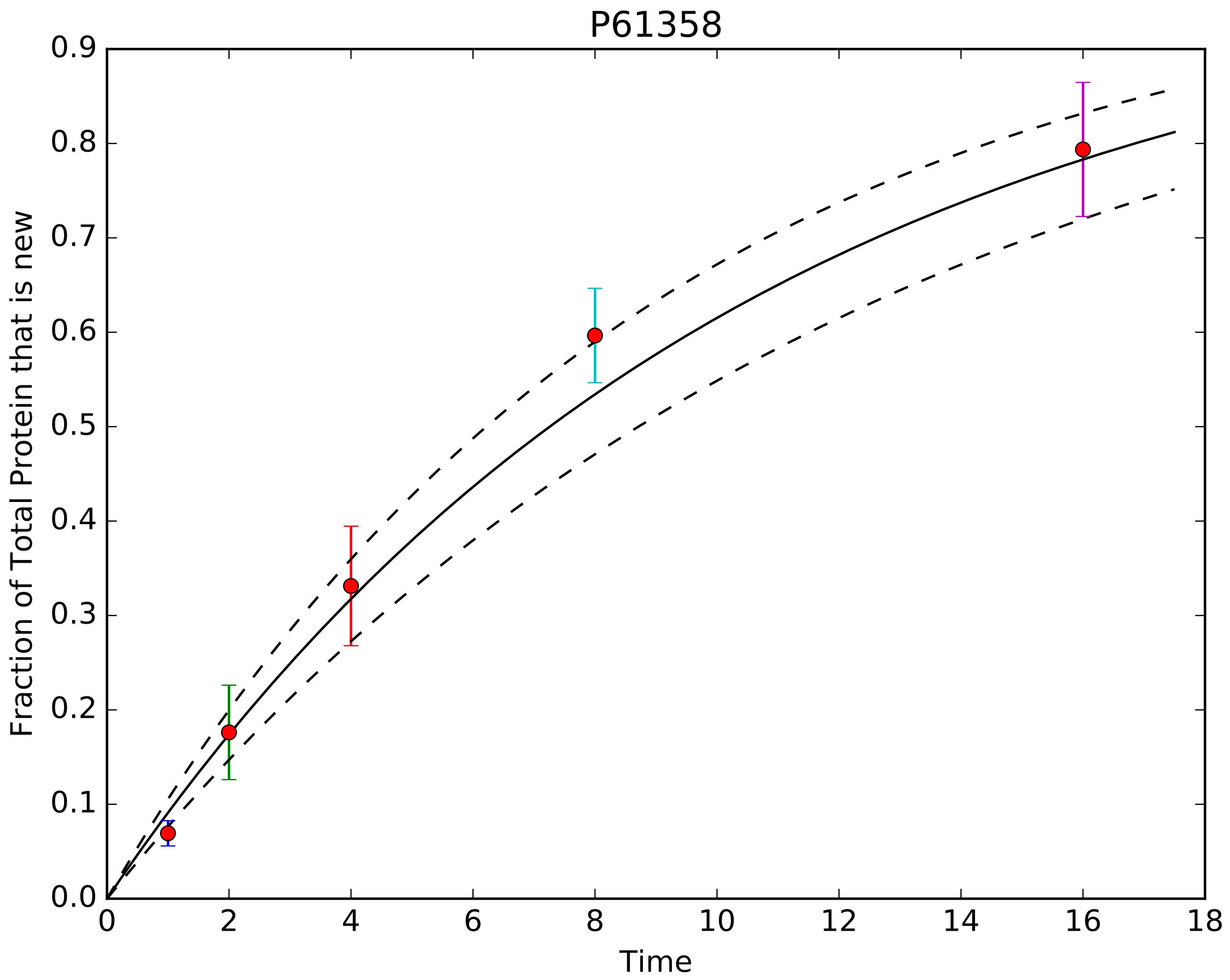
P61358
Fraction of Total Protein that is new
Time
0.0
0.1
0.2
0.3
0.4
0.5
0.6
0.7
0.8
0.9
0
2
4
6
8
10
12
14
16
18

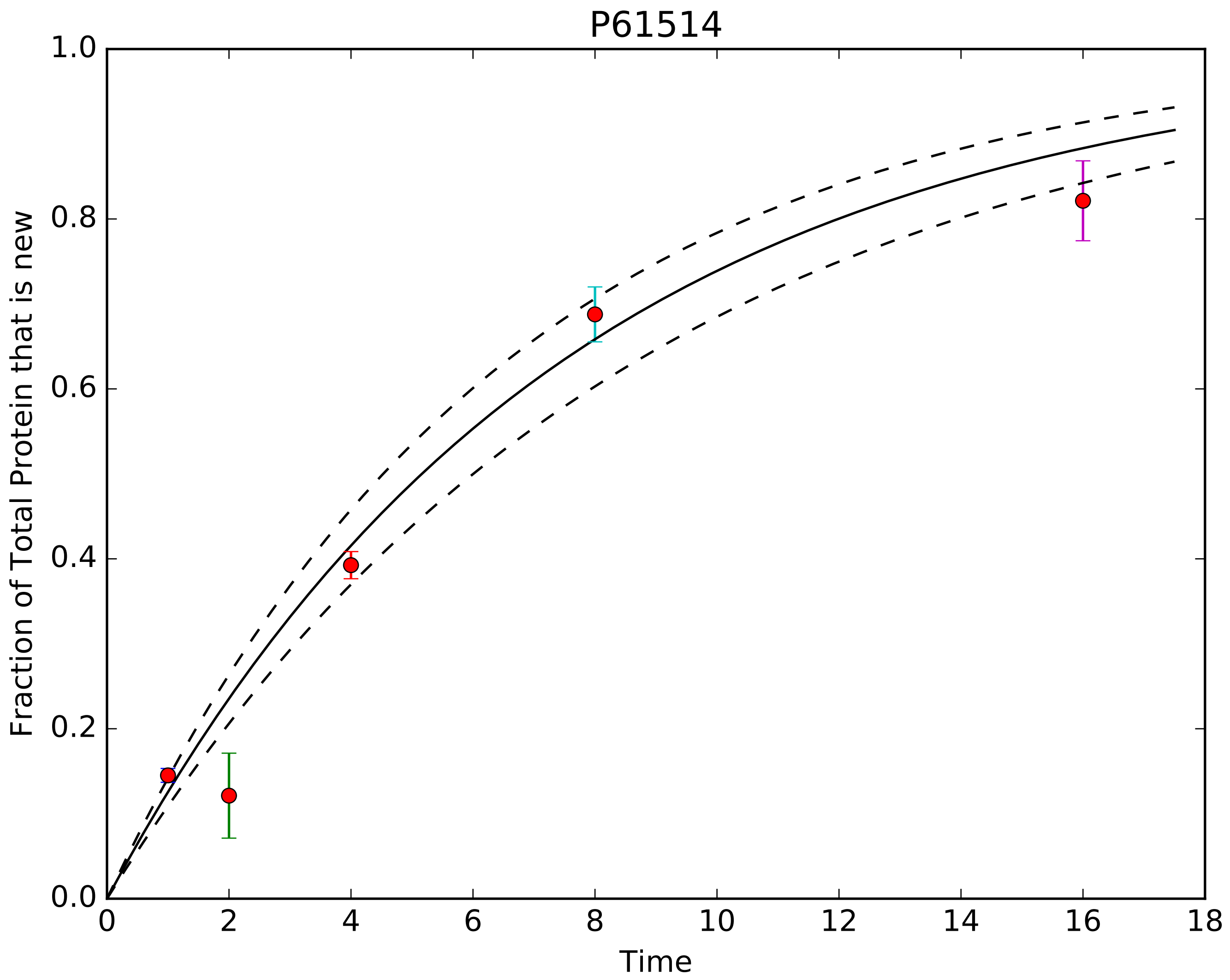
P61514
Fraction of Total Protein that is new
Time
0.0
0.2
0.4
0.6
0.8
1.0
0
2
4
6
8
10
12
14
16
18

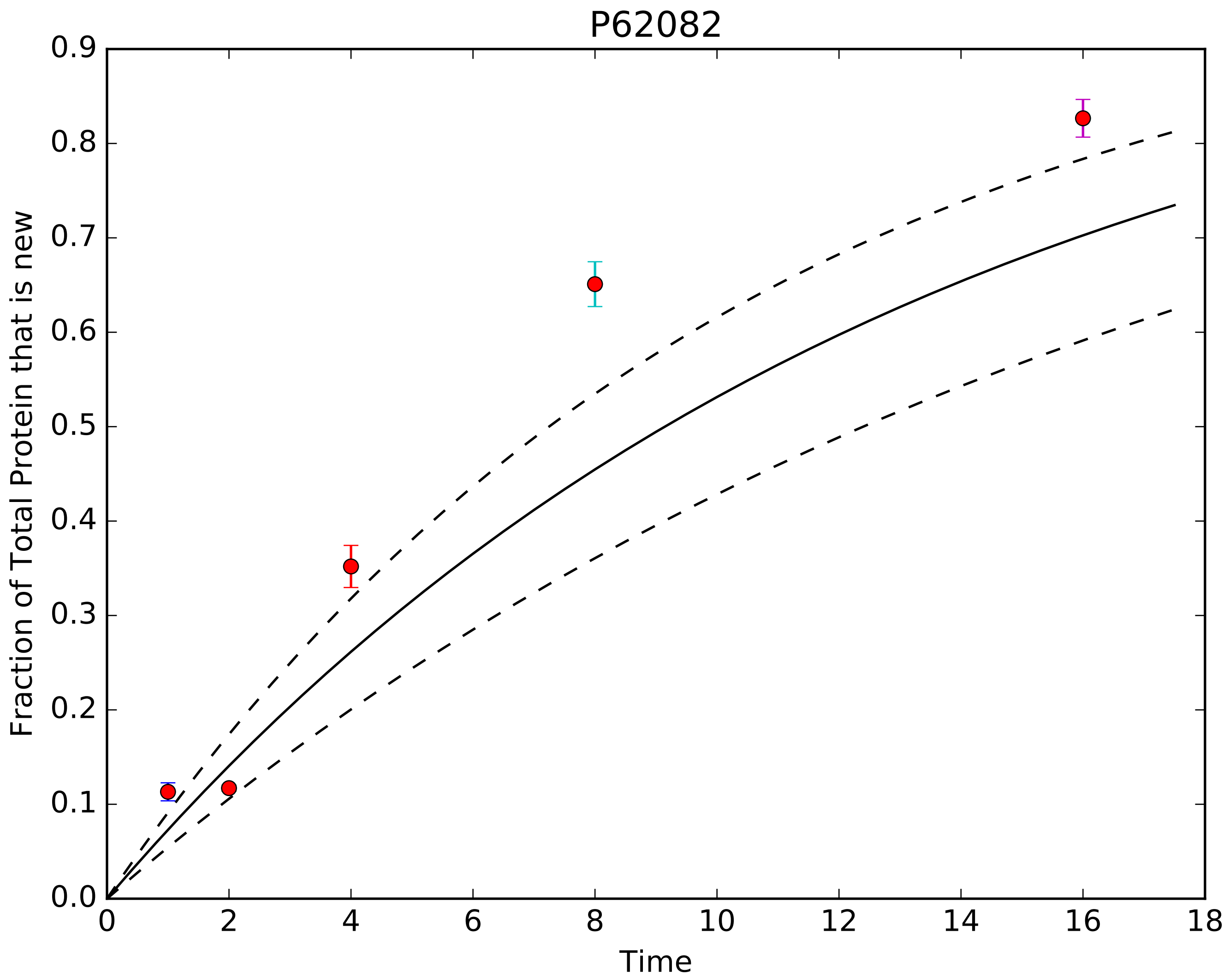
P62082
Fraction of Total Protein that is new
Time
0.0
0.1
0.2
0.3
0.4
0.5
0.6
0.7
0.8
0.9
0
2
4
6
8
10
12
14
16
18

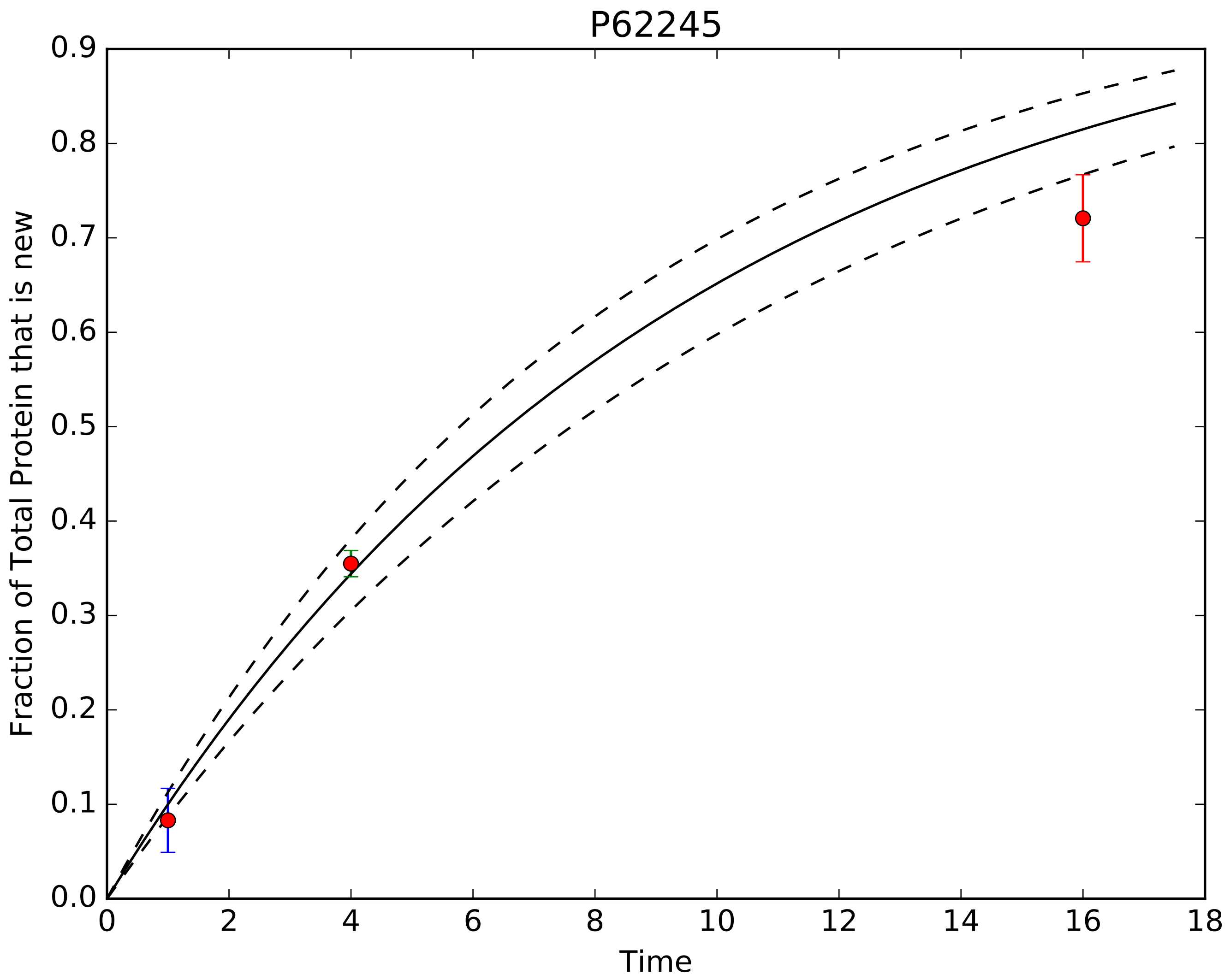
P62245
Fraction of Total Protein that is new
Time
0.0
0.1
0.2
0.3
0.4
0.5
0.6
0.7
0.8
0.9
0
2
4
6
8
10
12
14
16
18

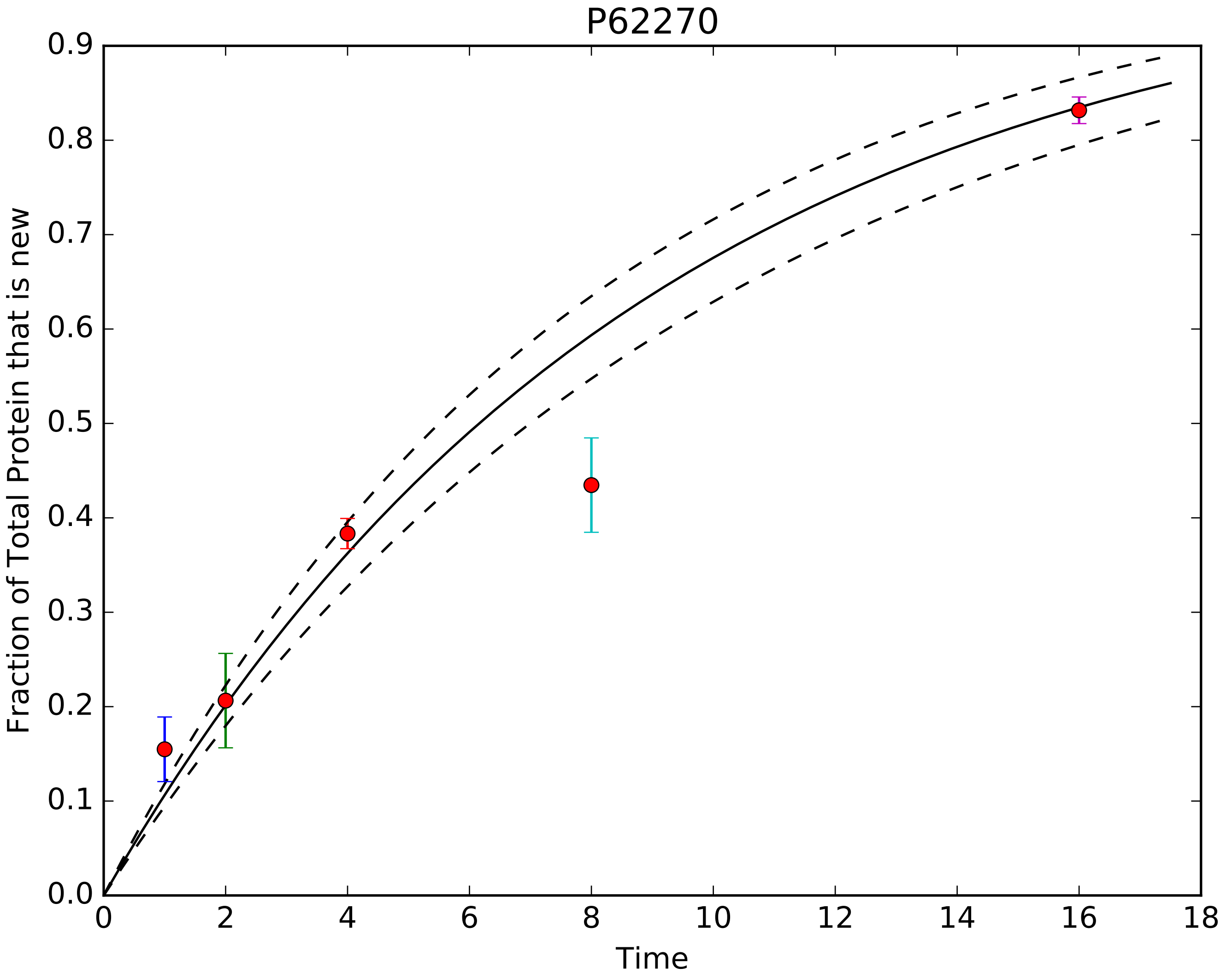
P62270
Fraction of Total Protein that is new
Time
0.0
0.1
0.2
0.3
0.4
0.5
0.6
0.7
0.8
0.9
0
2
4
6
8
10
12
14
16
18

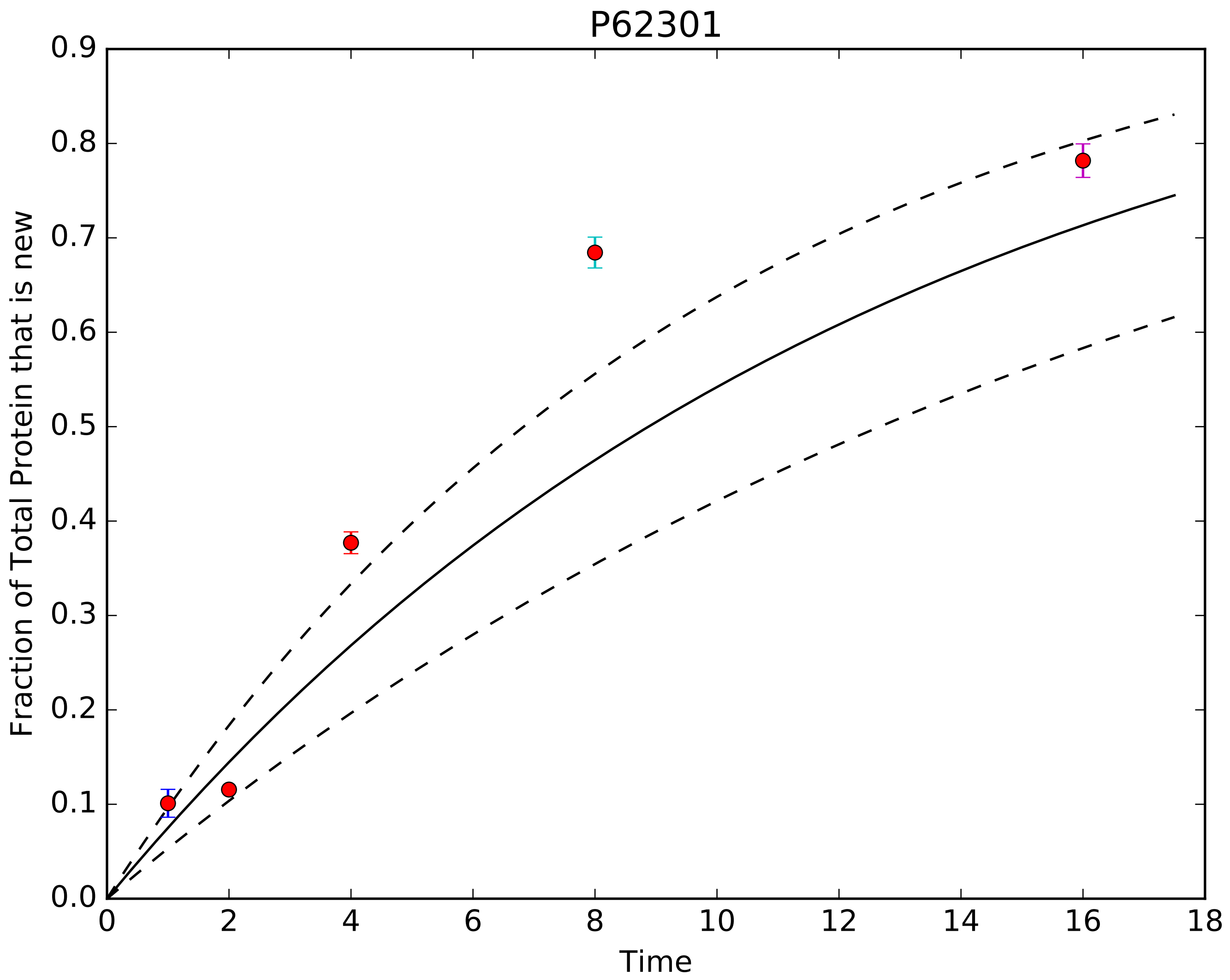
P62301
Fraction of Total Protein that is new
Time
0.0
0.1
0.2
0.3
0.4
0.5
0.6
0.7
0.8
0.9
0
2
4
6
8
10
12
14
16
18

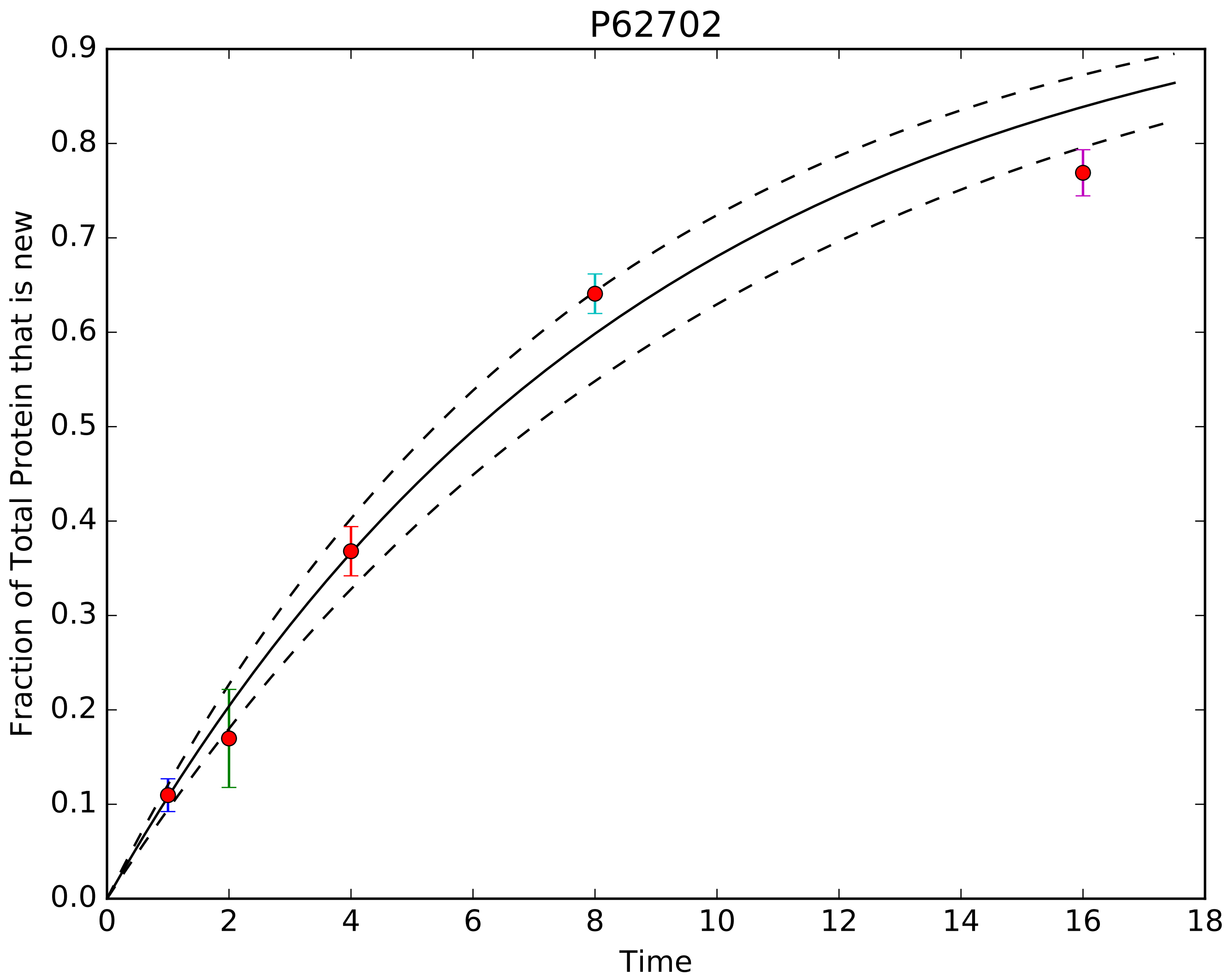
P62702
Fraction of Total Protein that is new
Time
0.0
0.1
0.2
0.3
0.4
0.5
0.6
0.7
0.8
0.9
0
2
4
6
8
10
12
14
16
18

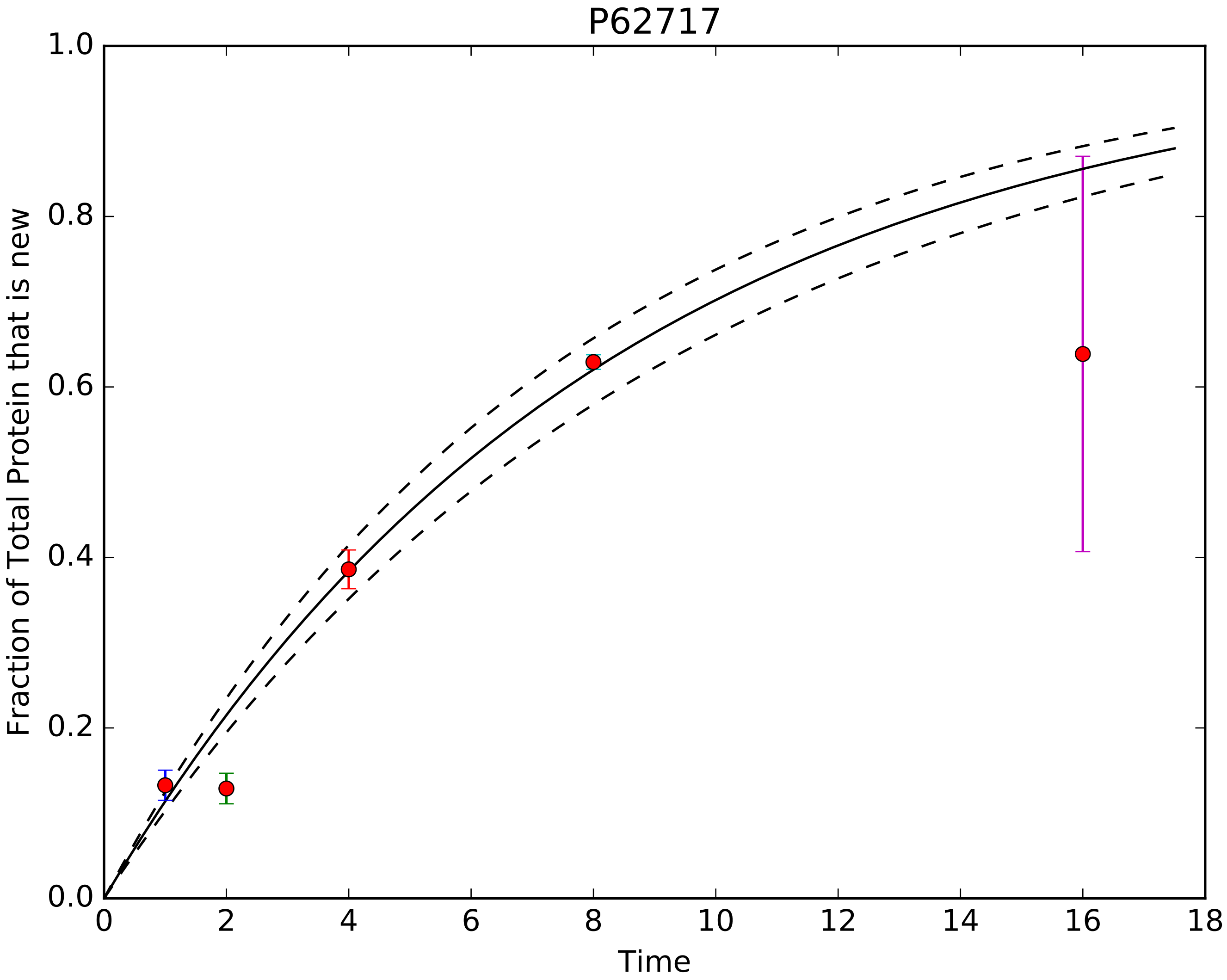
P62717
Fraction of Total Protein that is new
Time
0.0
0.2
0.4
0.6
0.8
1.0
0
2
4
6
8
10
12
14
16
18

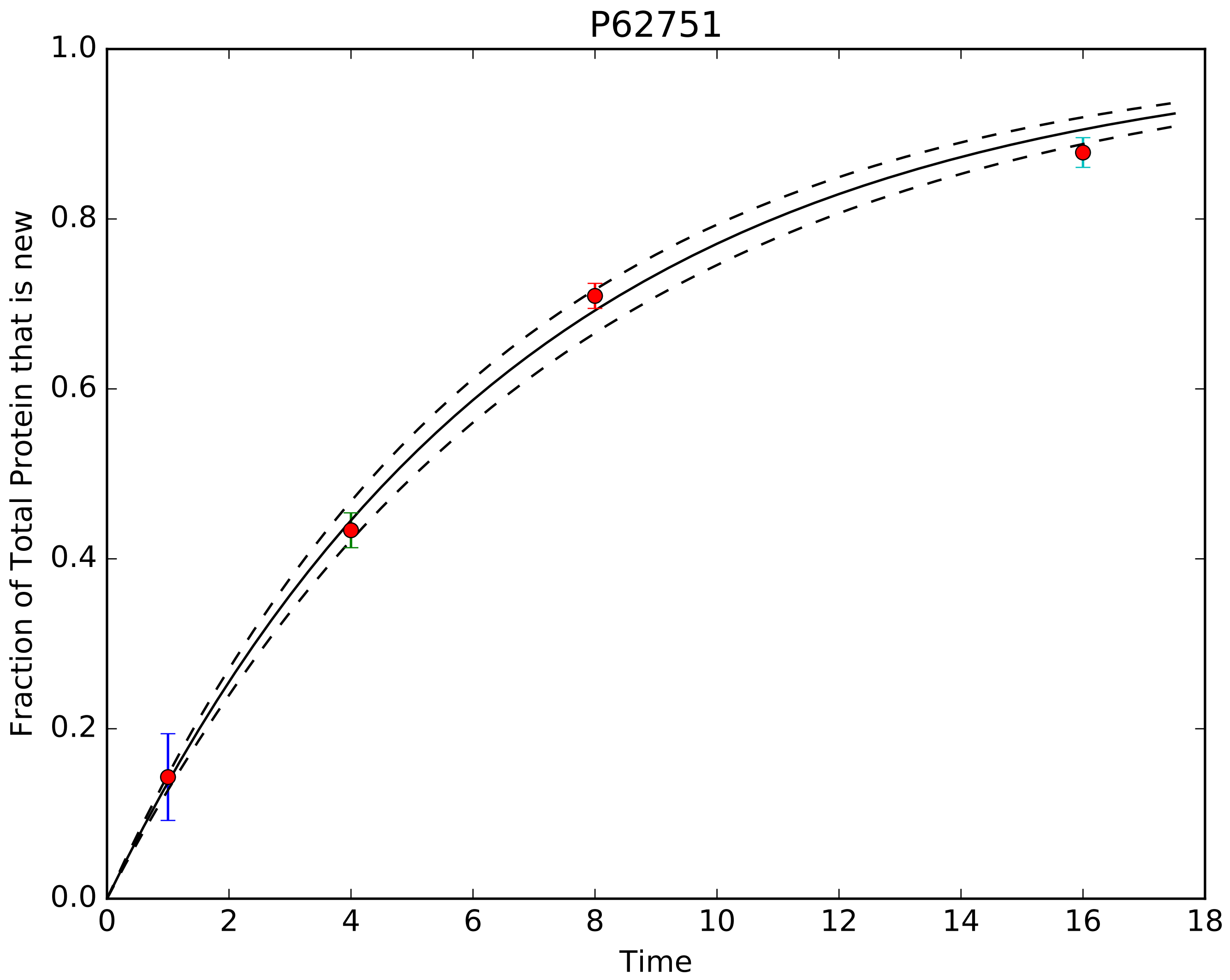

P62751
Fraction of Total Protein that is new
Time
0.0
0.2
0.4
0.6
0.8
1.0
0
2
4
6
8
10
12
14
16
18

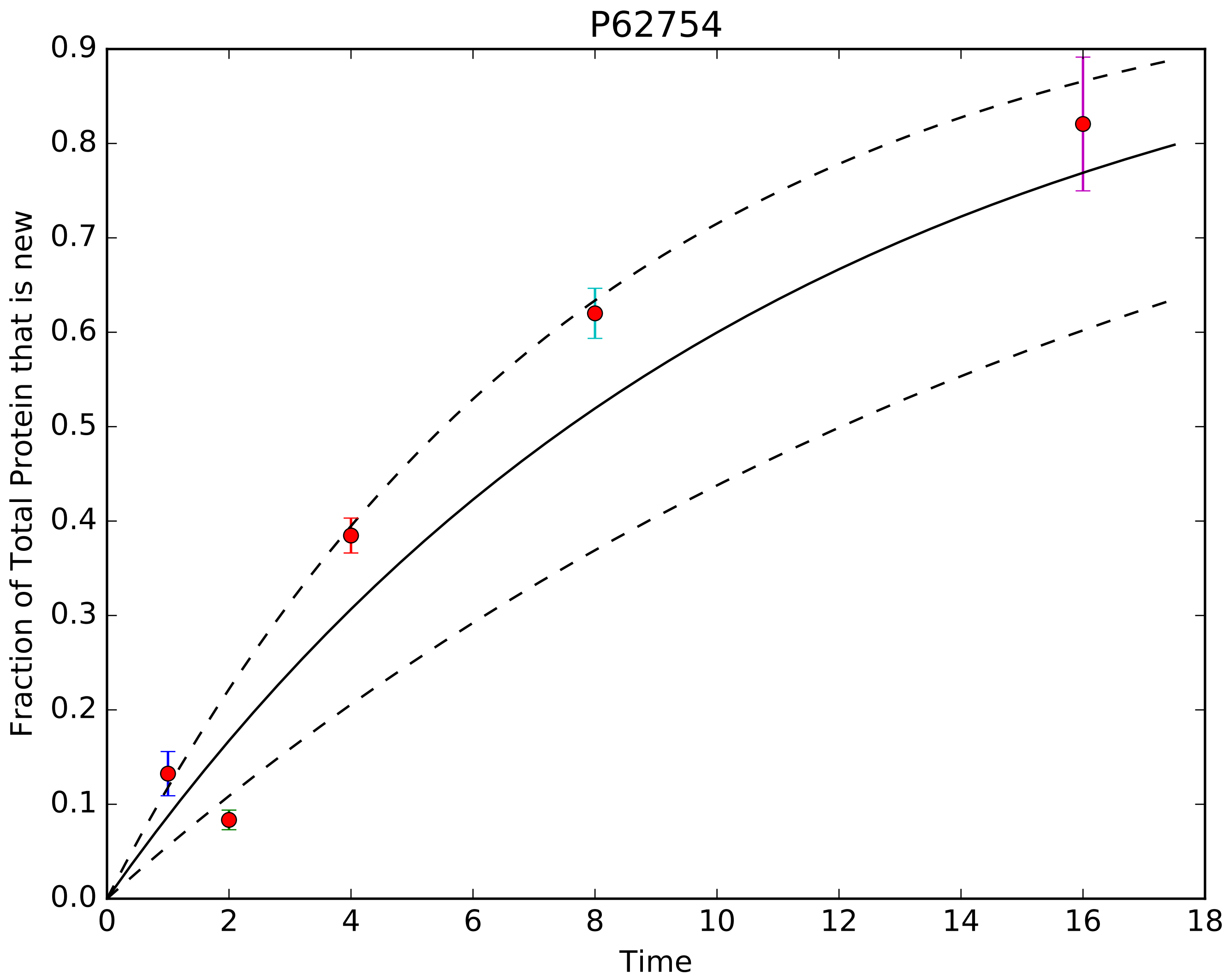

P62754
Fraction of Total Protein that is new
Time
0.0
0.1
0.2
0.3
0.4
0.5
0.6
0.7
0.8
0.9
0
2
4
6
8
10
12
14
16
18

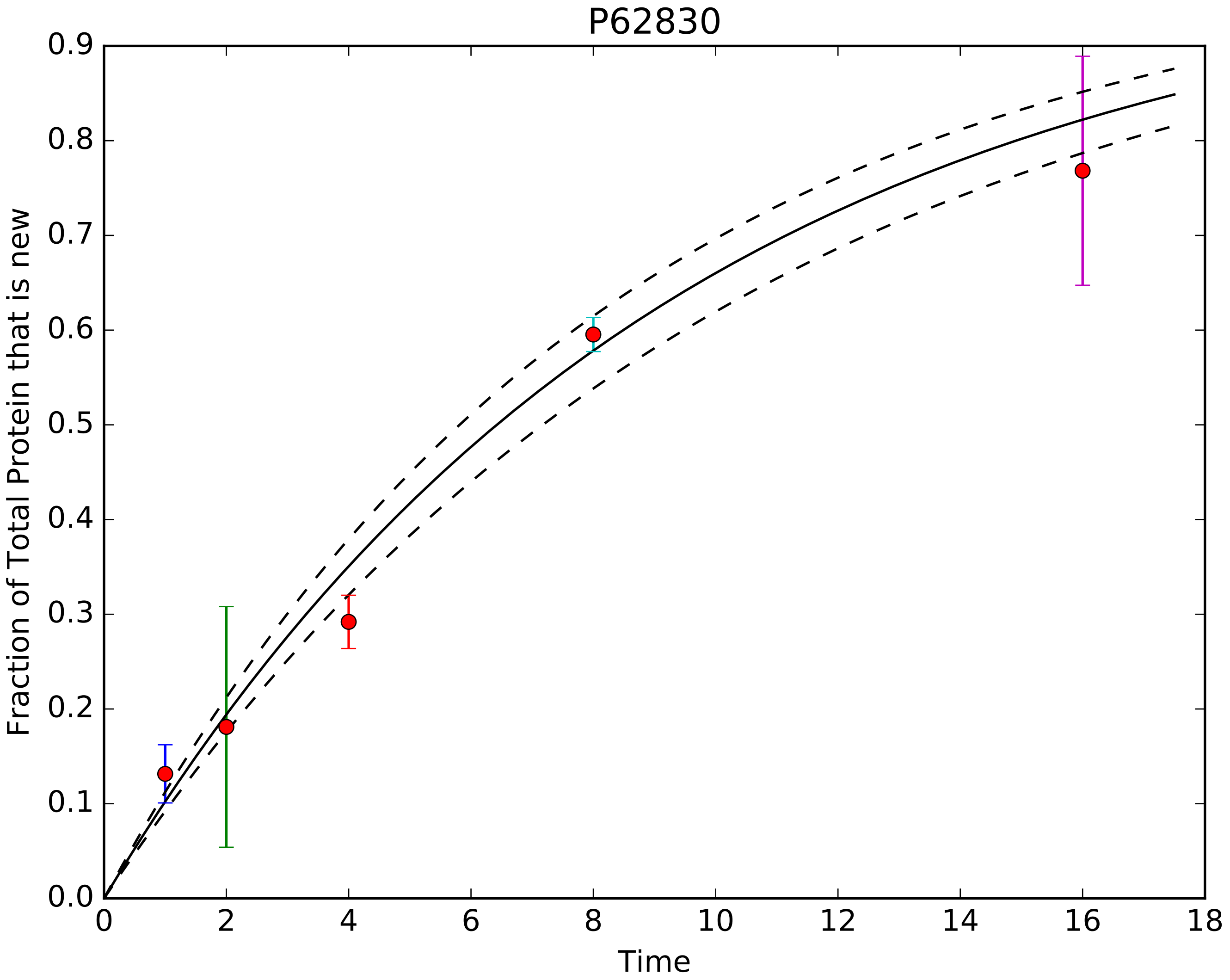

P62830
Fraction of Total Protein that is new
Time
0.0
0.1
0.2
0.3
0.4
0.5
0.6
0.7
0.8
0.9
0
2
4
6
8
10
12
14
16
18

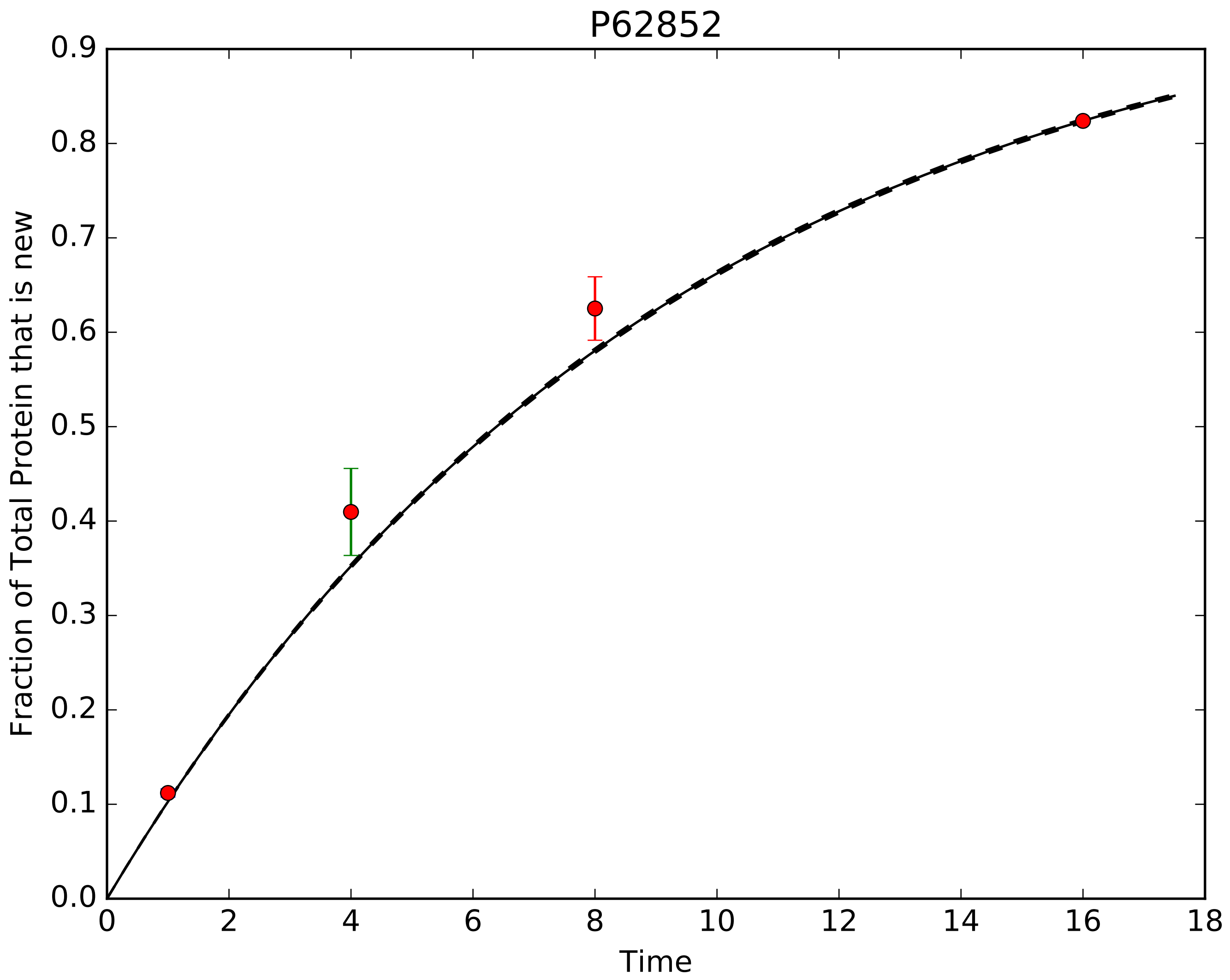
P62852
Fraction of Total Protein that is new
Time
0.0
0.1
0.2
0.3
0.4
0.5
0.6
0.7
0.8
0.9
0
2
4
6
8
10
12
14
16
18

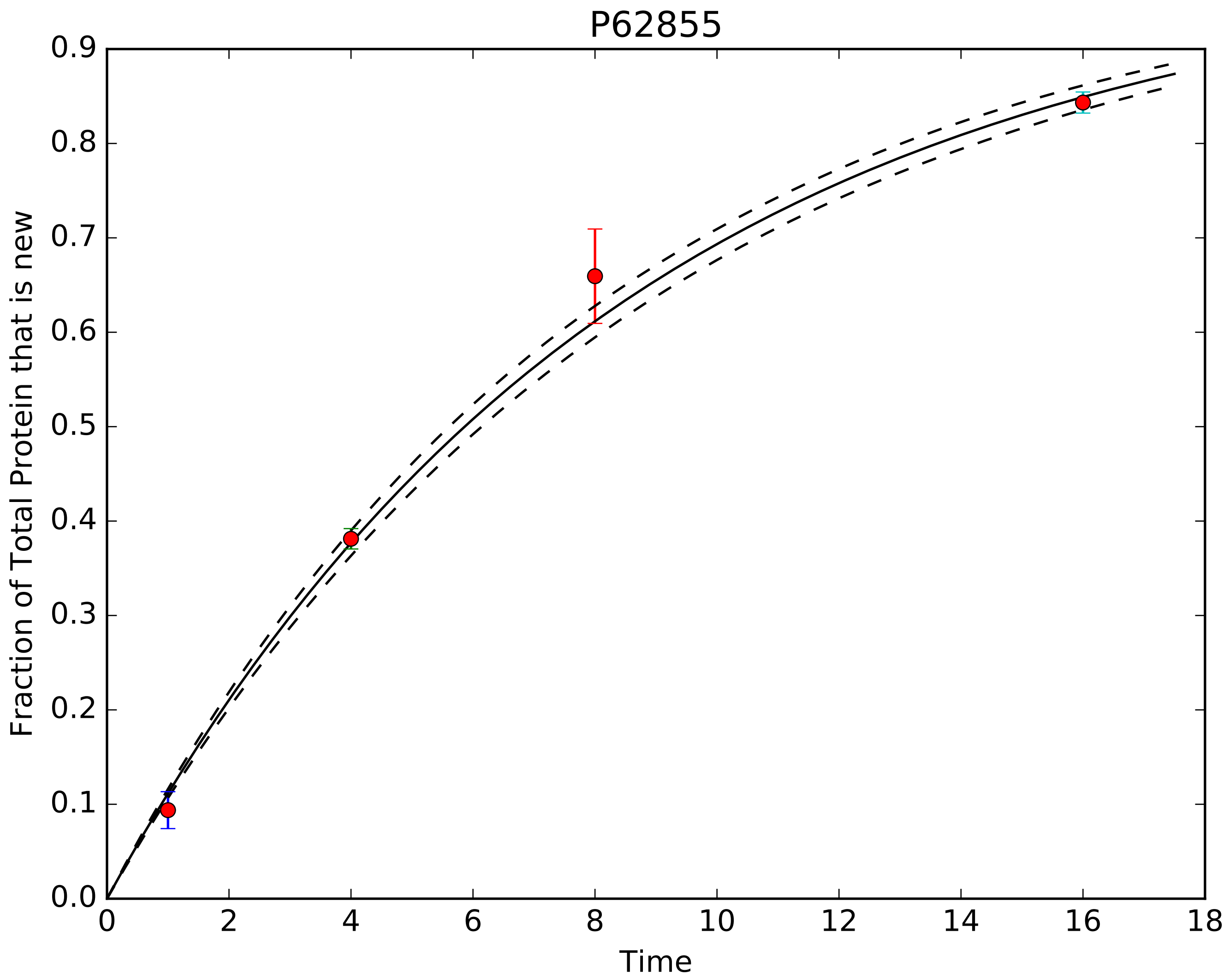

P62855
Fraction of Total Protein that is new
Time
0.0
0.1
0.2
0.3
0.4
0.5
0.6
0.7
0.8
0.9
0
2
4
6
8
10
12
14
16
18

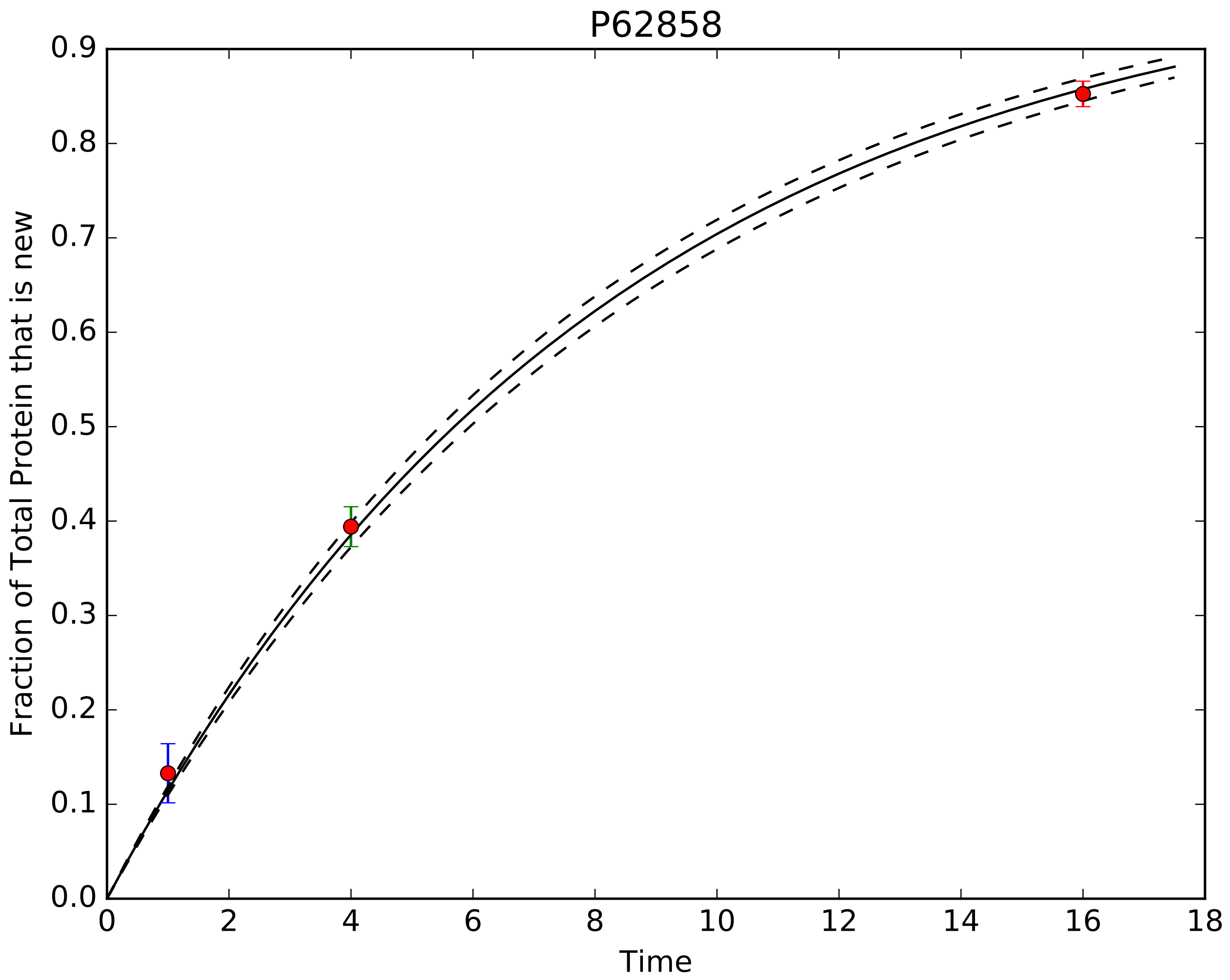
P62858
Fraction of Total Protein that is new
Time
0.0
0.1
0.2
0.3
0.4
0.5
0.6
0.7
0.8
0.9
0
2
4
6
8
10
12
14
16
18

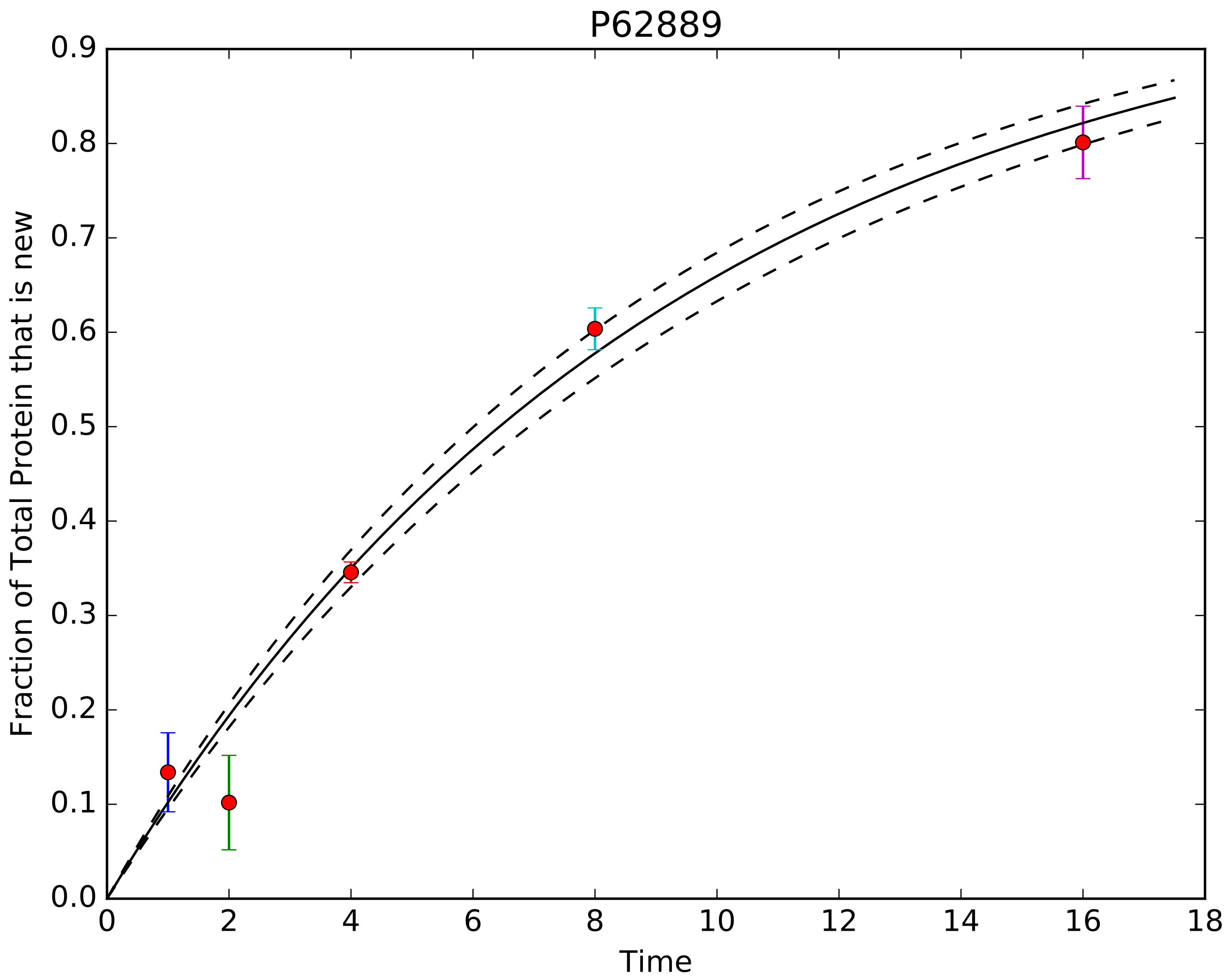

P62889
Fraction of Total Protein that is new
Time
0.0
0.1
0.2
0.3
0.4
0.5
0.6
0.7
0.8
0.9
0
2
4
6
8
10
12
14
16
18

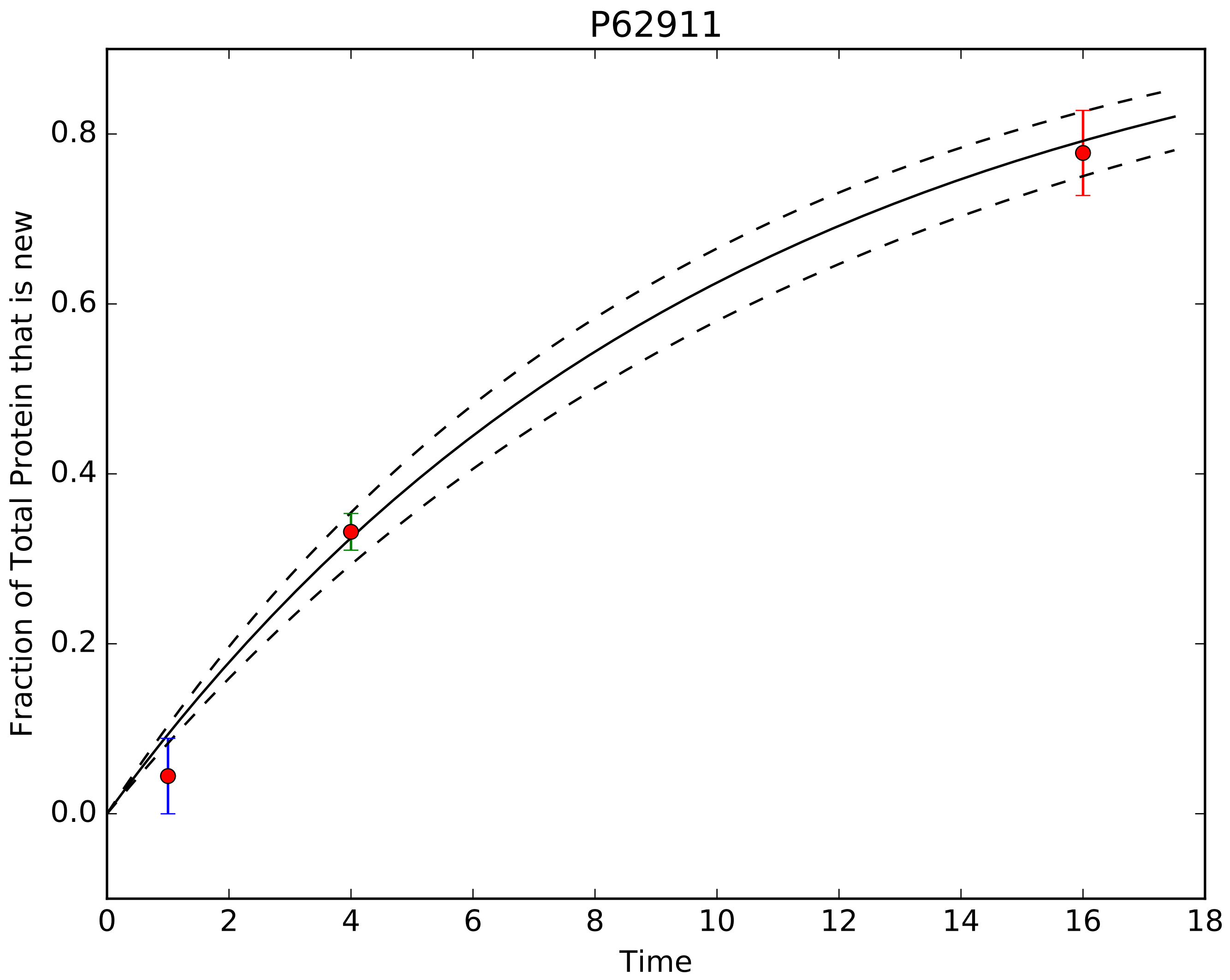
P62911
Fraction of Total Protein that is new
Time
0.0
0.2
0.4
0.6
0.8
0
2
4
6
8
10
12
14
16
18

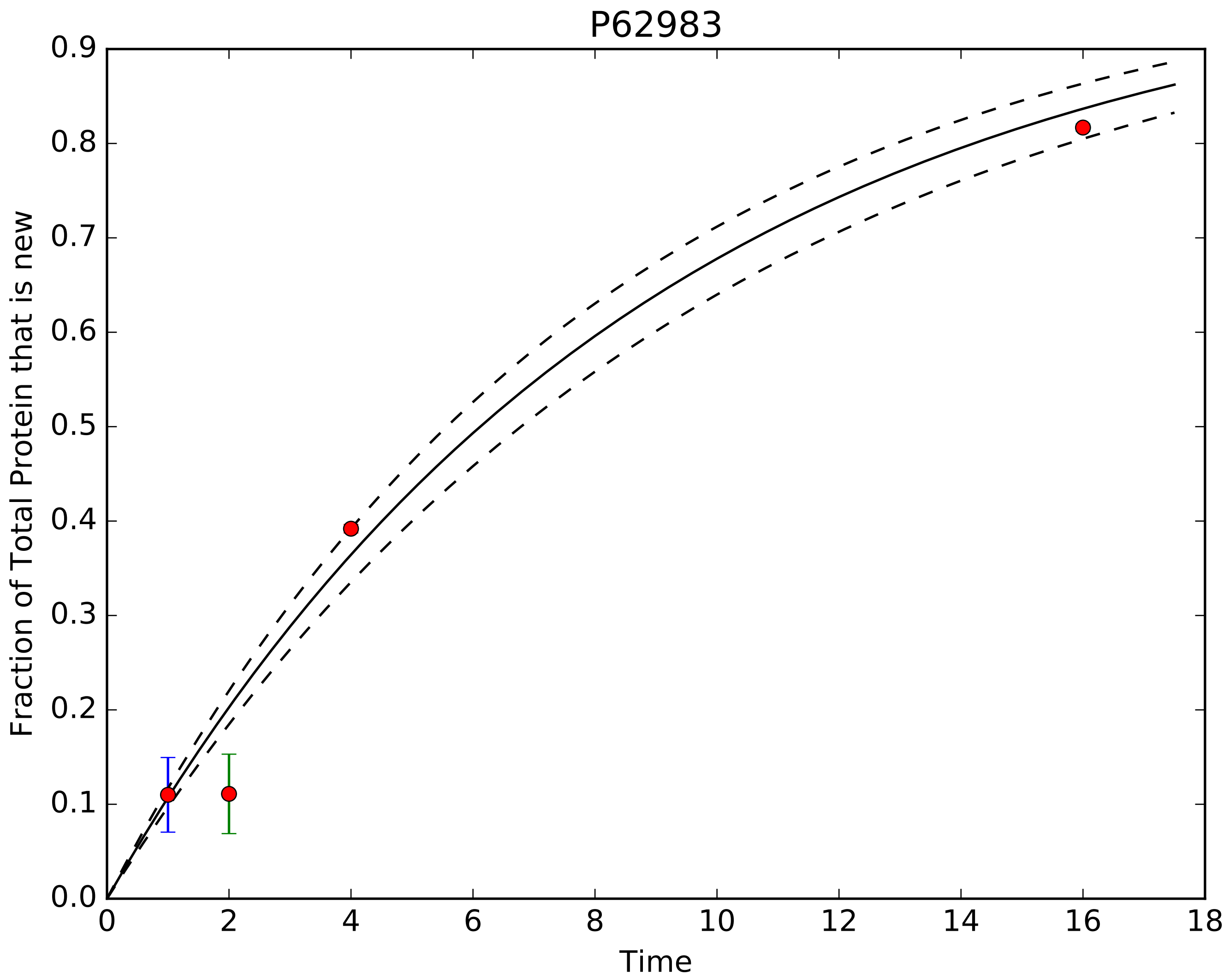
P62983
Fraction of Total Protein that is new
Time
0.0
0.1
0.2
0.3
0.4
0.5
0.6
0.7
0.8
0.9
0
2
4
6
8
10
12
14
16
18

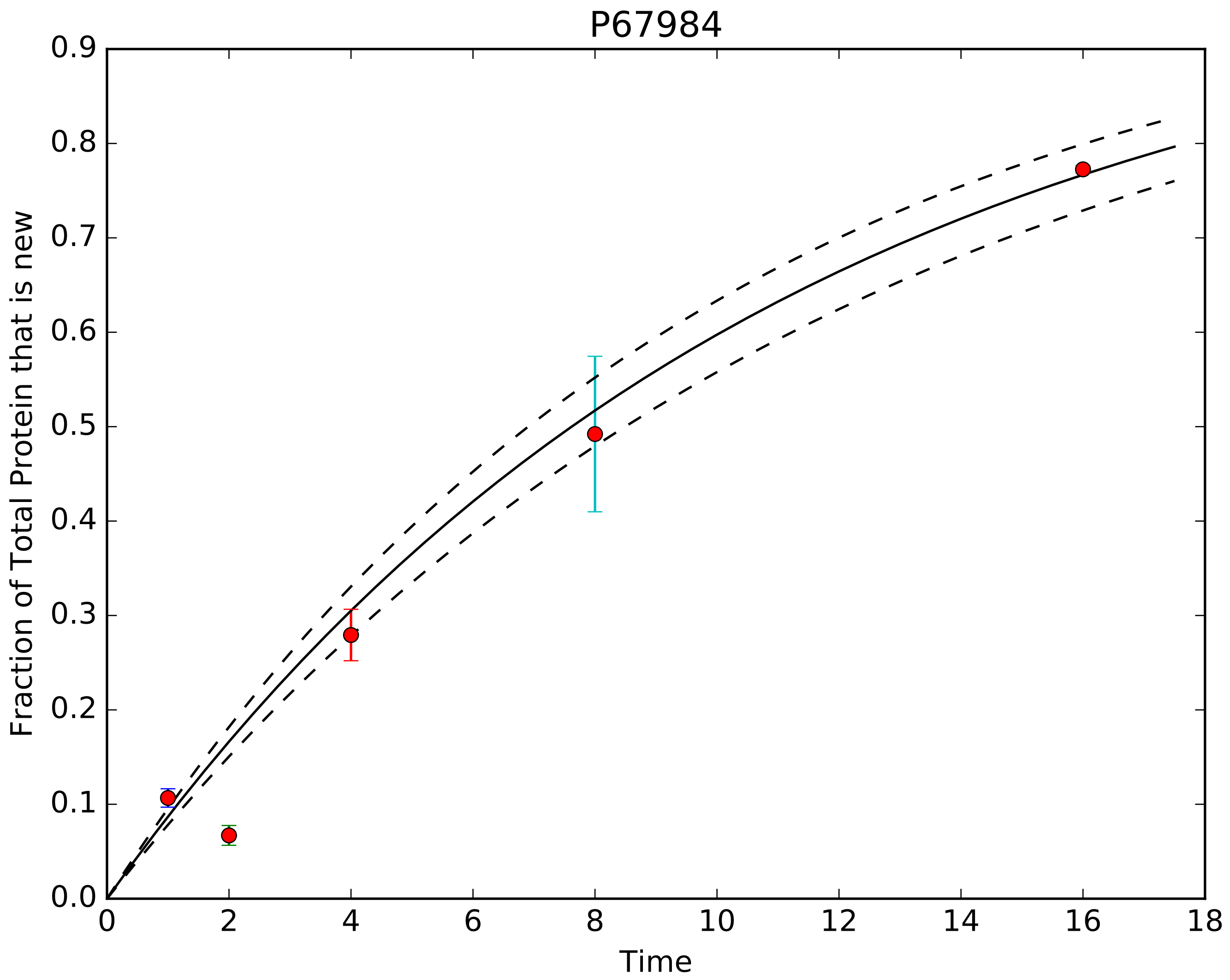
P67984
Fraction of Total Protein that is new
Time
0.0
0.1
0.2
0.3
0.4
0.5
0.6
0.7
0.8
0.9
0
2
4
6
8
10
12
14
16
18

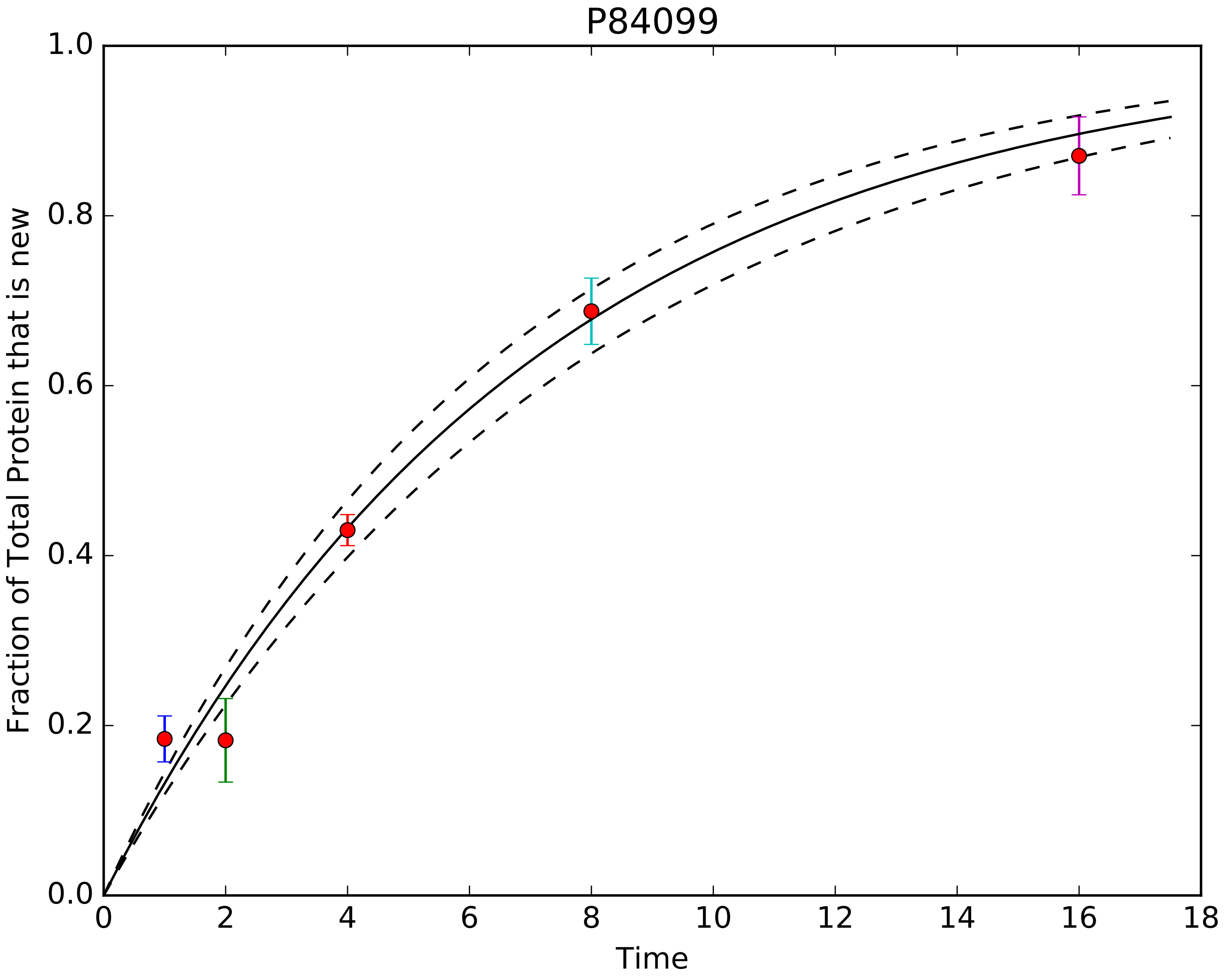

P84099
Fraction of Total Protein that is new
Time
0.0
0.2
0.4
0.6
0.8
1.0
0
2
4
6
8
10
12
14
16
18

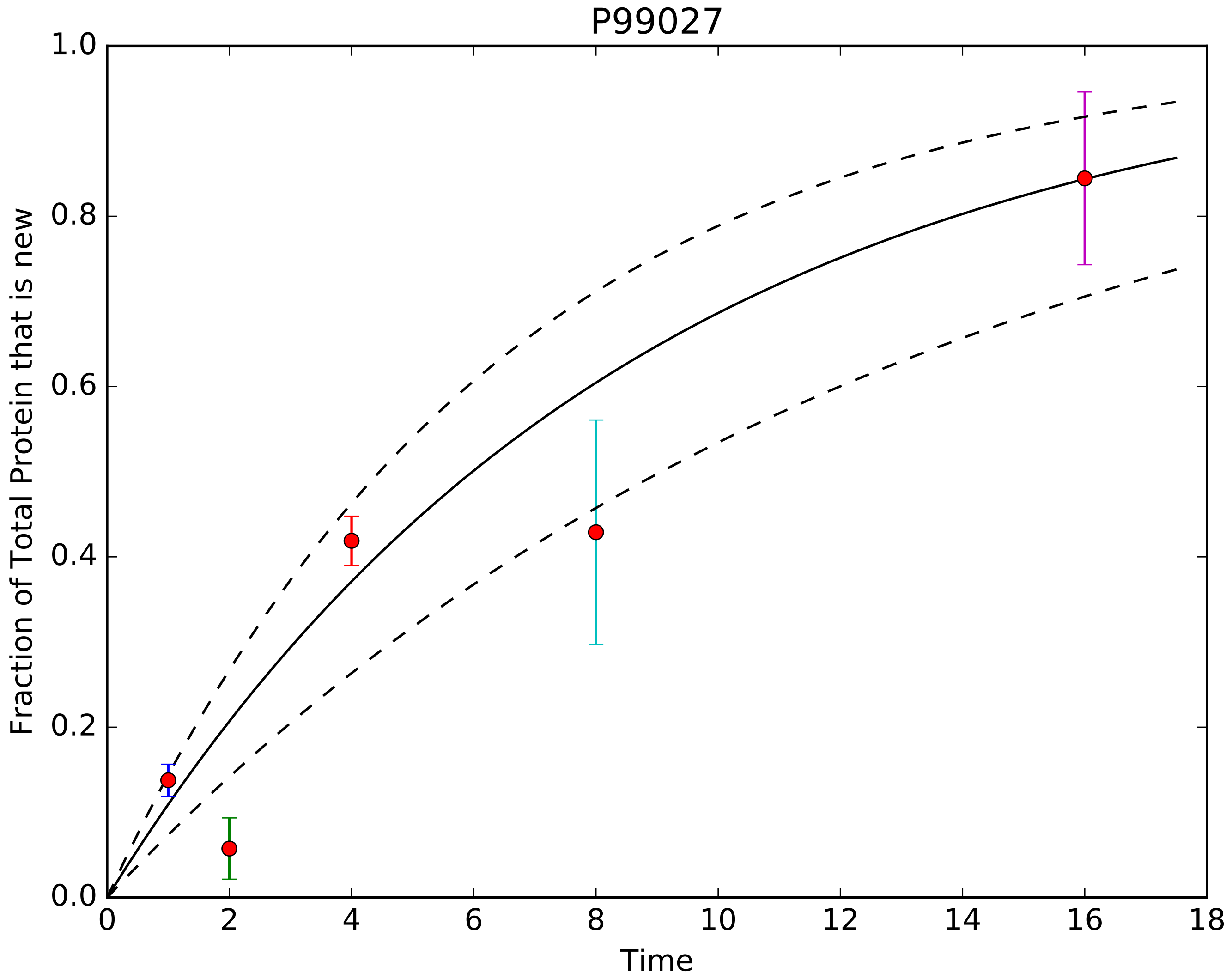

P99027
Fraction of Total Protein that is new
Time
0.0
0.2
0.4
0.6
0.8
1.0
0
2
4
6
8
10
12
14
16
18

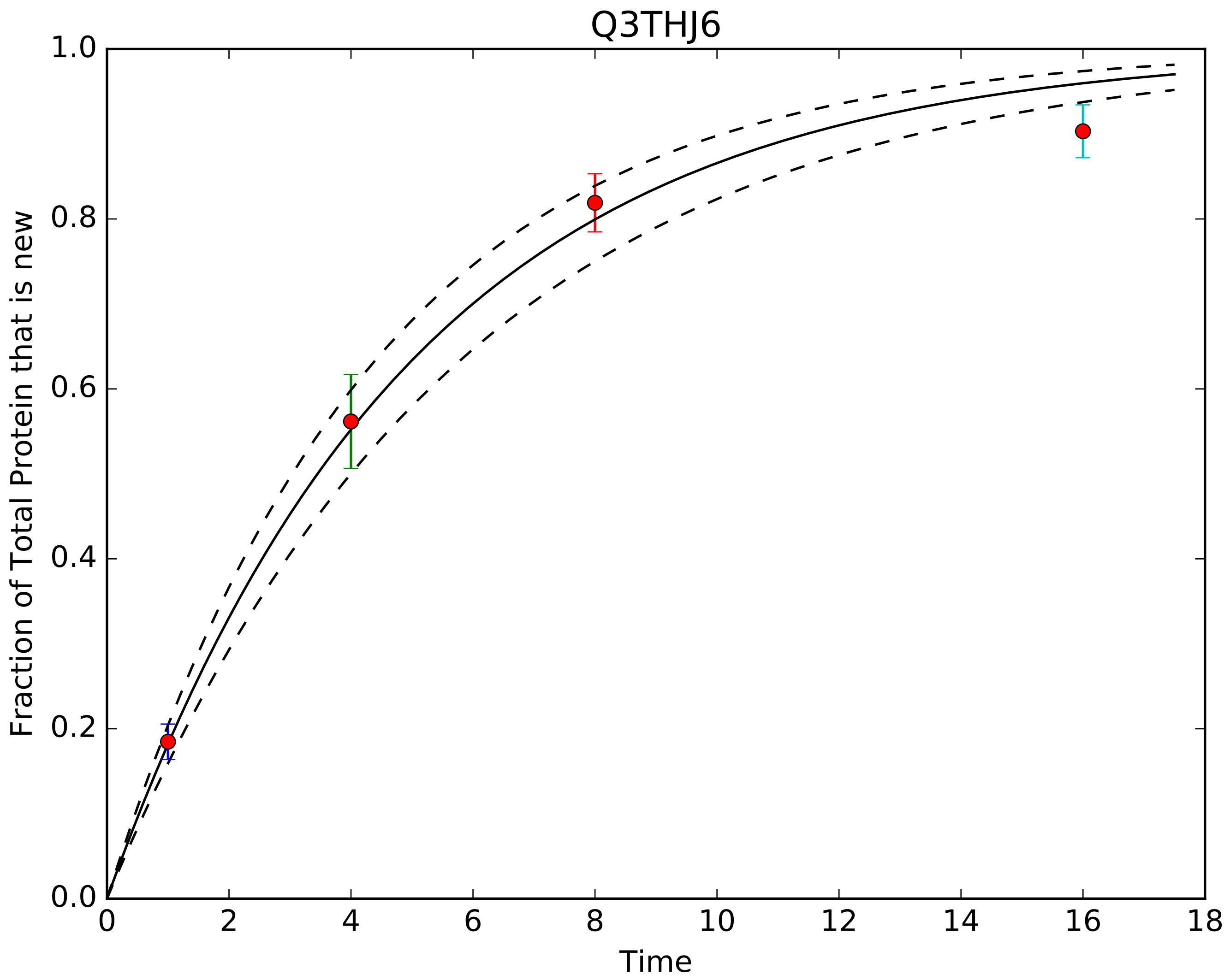
Q3THJ6
Fraction of Total Protein that is new
Time
0.0
0.2
0.4
0.6
0.8
1.0
0
2
4
6
8
10
12
14
16
18

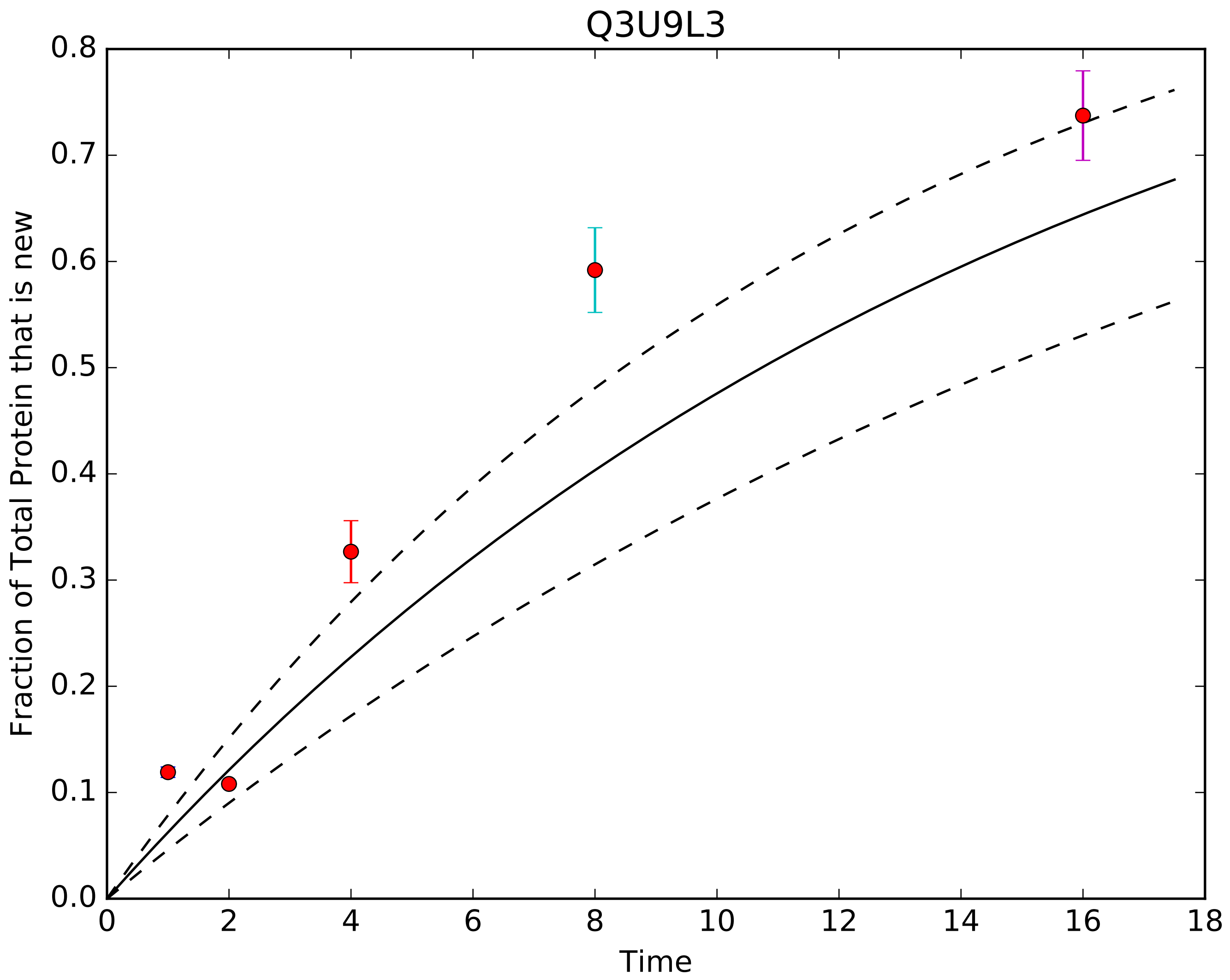
Q3U9L3
Fraction of Total Protein that is new
Time
0.0
0.1
0.2
0.3
0.4
0.5
0.6
0.7
0.8
0
2
4
6
8
10
12
14
16
18

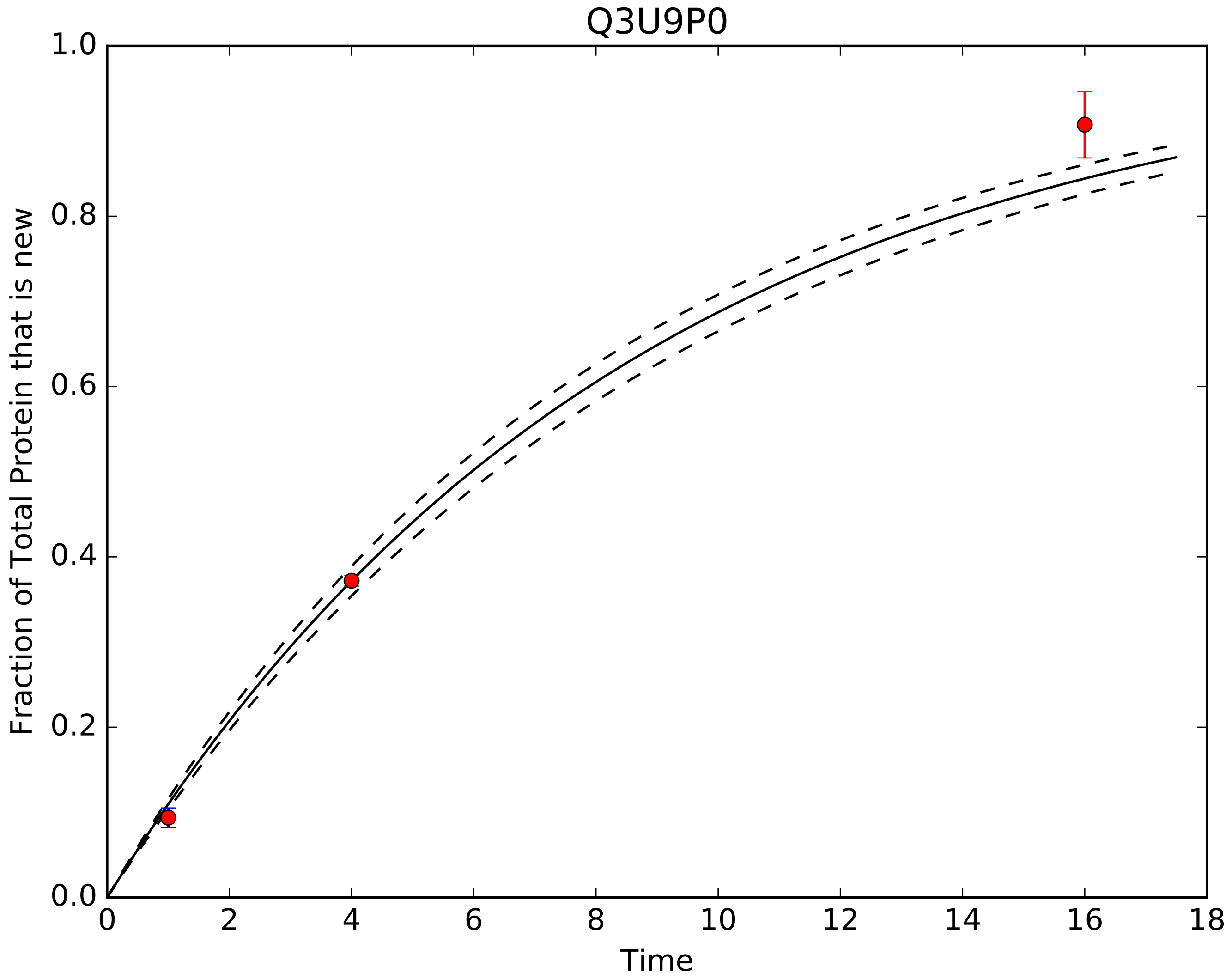
Q3U9P0
Fraction of Total Protein that is new
Time
0.0
0.2
0.4
0.6
0.8
1.0
0
2
4
6
8
10
12
14
16
18

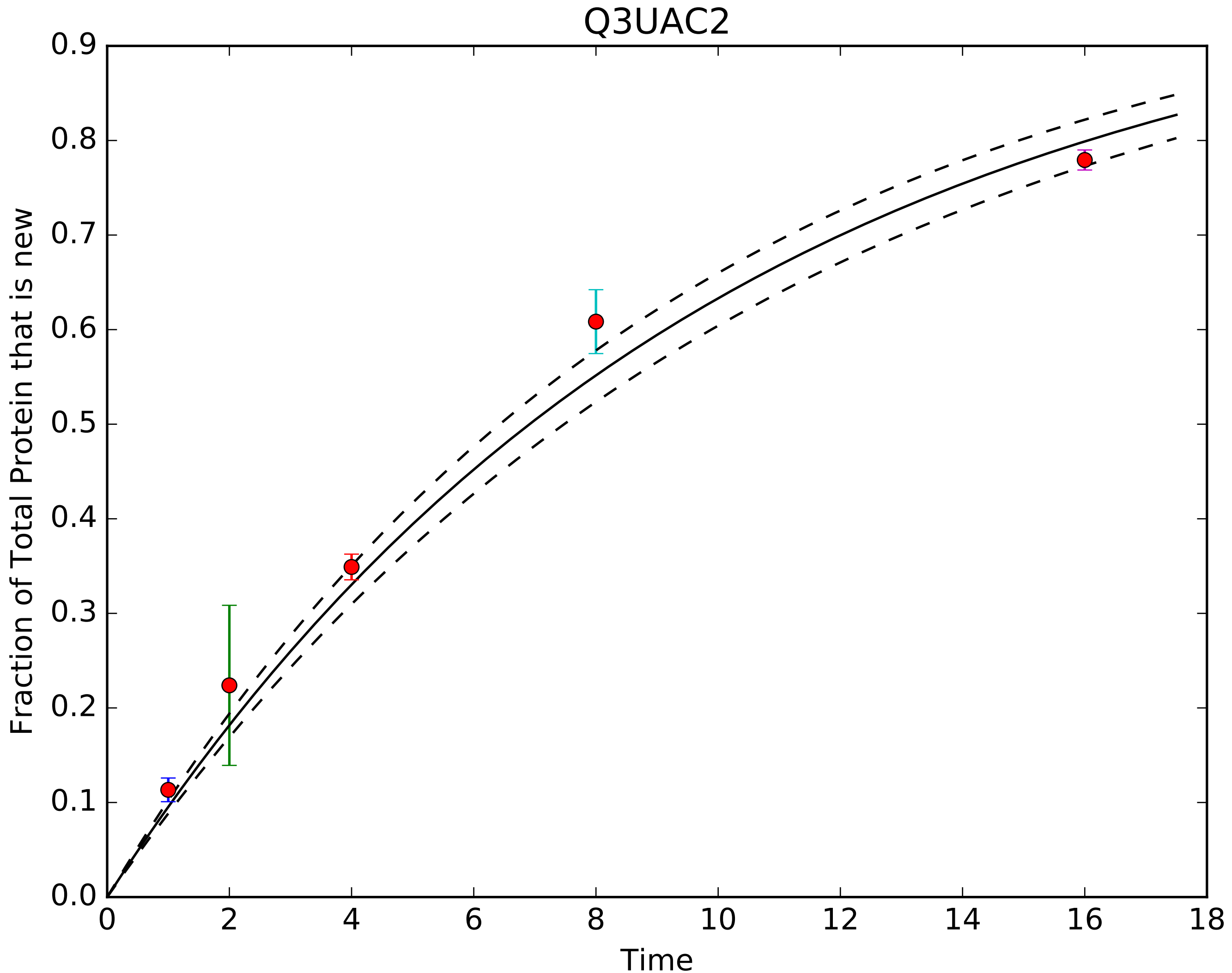

Q3UAC2
Fraction of Total Protein that is new
Time
0.0
0.1
0.2
0.3
0.4
0.5
0.6
0.7
0.8
0.9
0
2
4
6
8
10
12
14
16
18

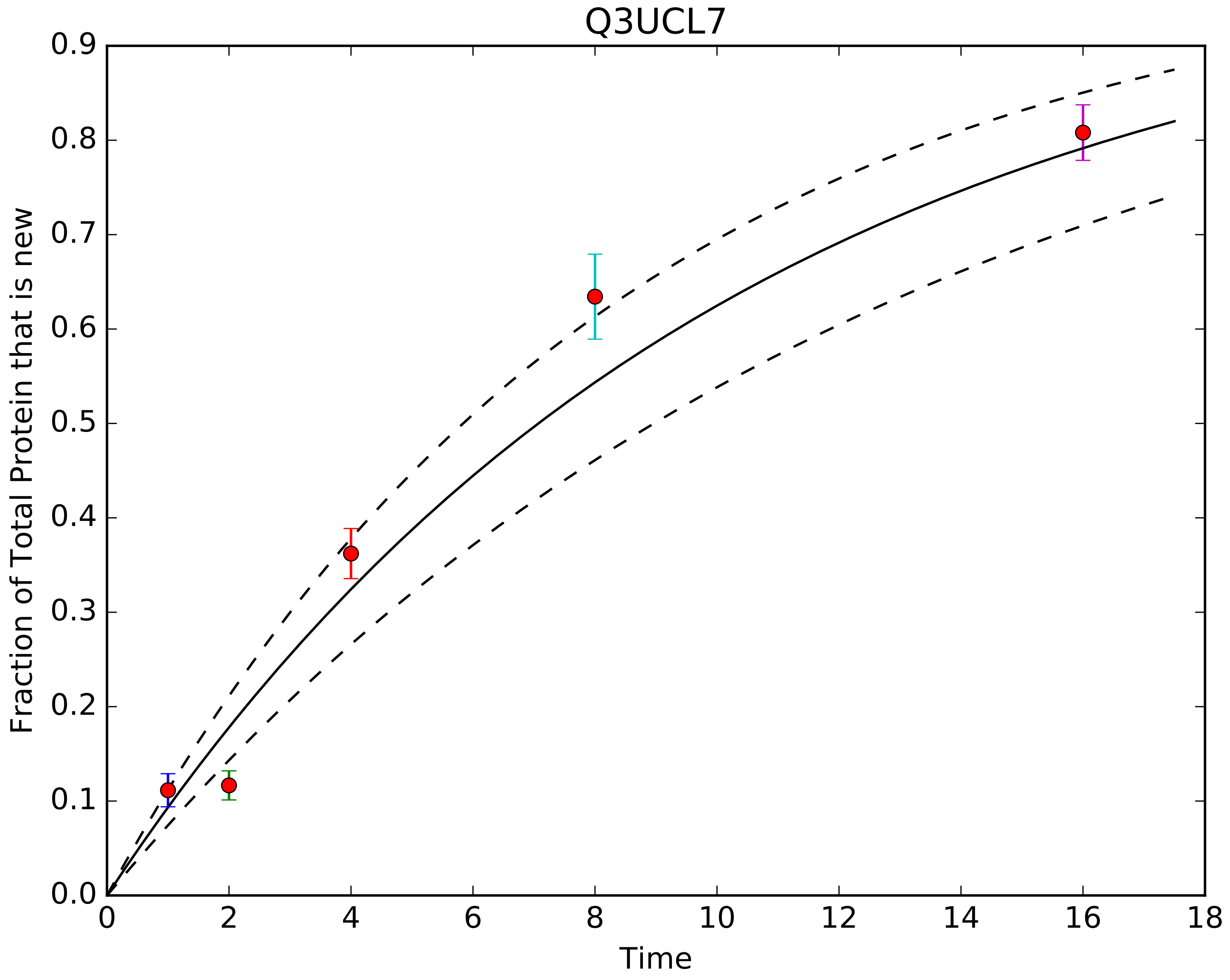
Q3UCL7
Fraction of Total Protein that is new
Time
0.0
0.1
0.2
0.3
0.4
0.5
0.6
0.7
0.8
0.9
0
2
4
6
8
10
12
14
16
18

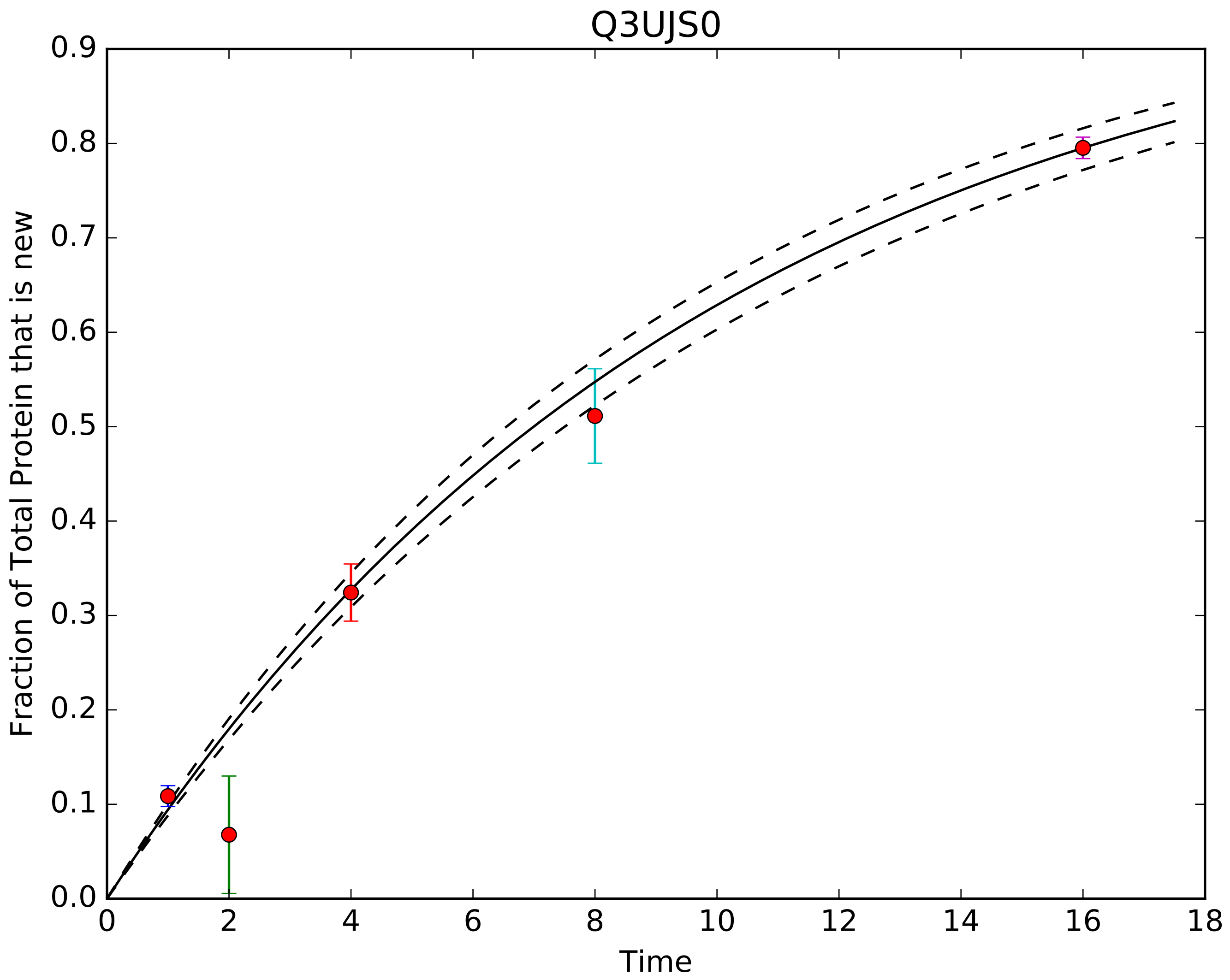
Q3UJS0
Fraction of Total Protein that is new
Time
0.0
0.1
0.2
0.3
0.4
0.5
0.6
0.7
0.8
0.9
0
2
4
6
8
10
12
14
16
18

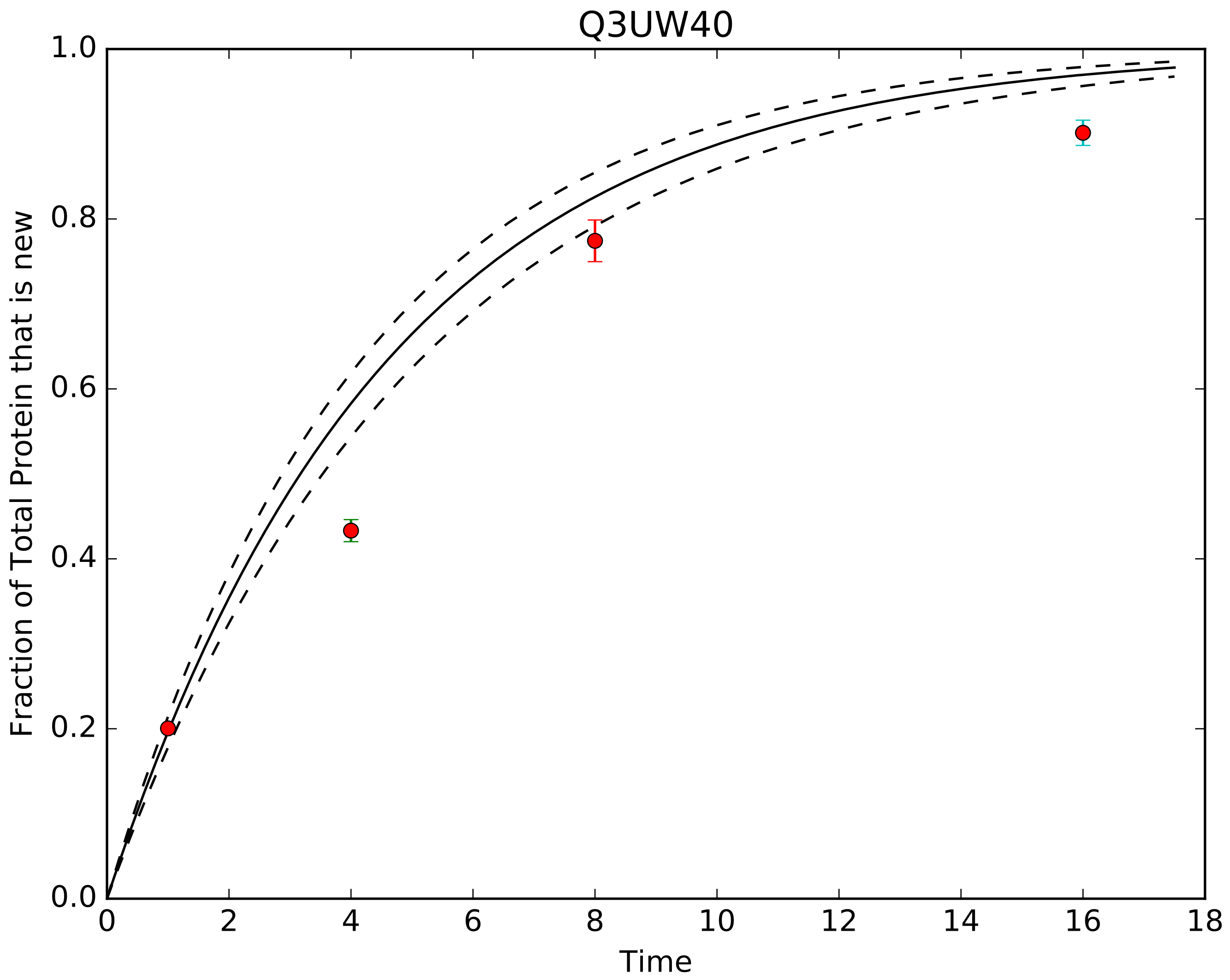
Q3UW40
Fraction of Total Protein that is new
Time
0.0
0.2
0.4
0.6
0.8
1.0
0
2
4
6
8
10
12
14
16
18

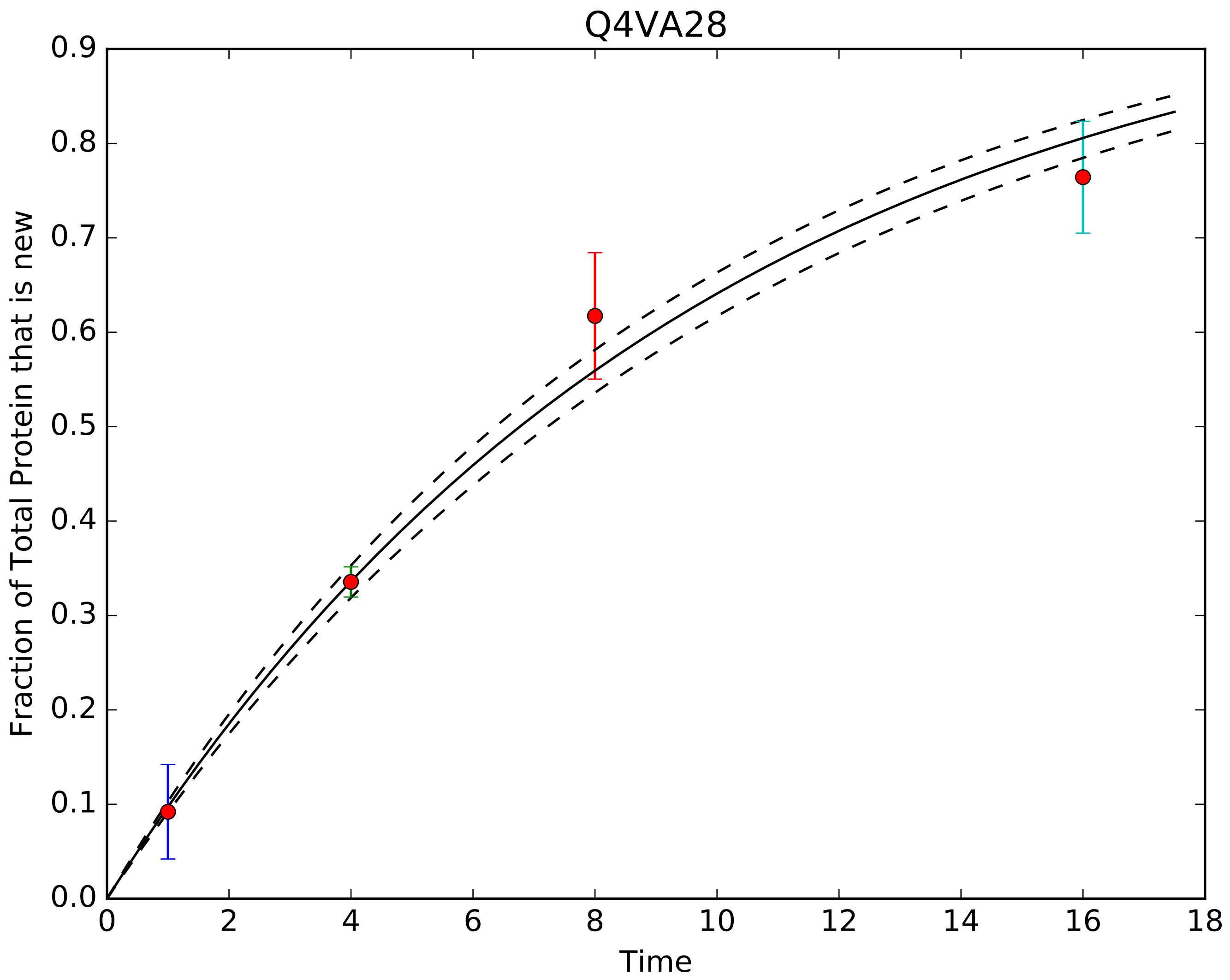

Q4VA28
Fraction of Total Protein that is new
Time
0.0
0.1
0.2
0.3
0.4
0.5
0.6
0.7
0.8
0.9
0
2
4
6
8
10
12
14
16
18

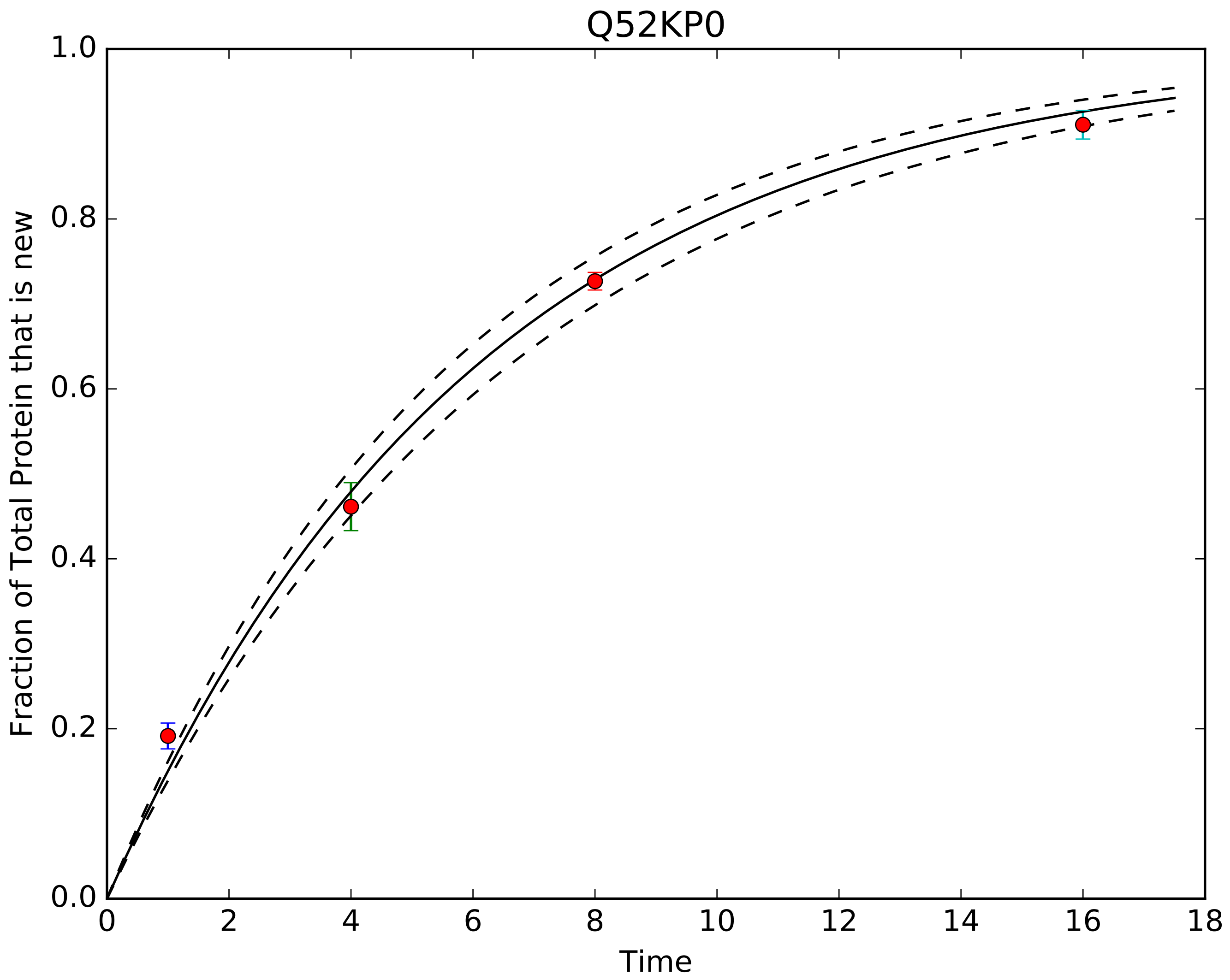

Q52KP0
1.0
0.8
0.6
0.4
0.2
0.0
Fraction of Total Protein that is new
0
2
4
6
8
10
12
14
16
18
Time

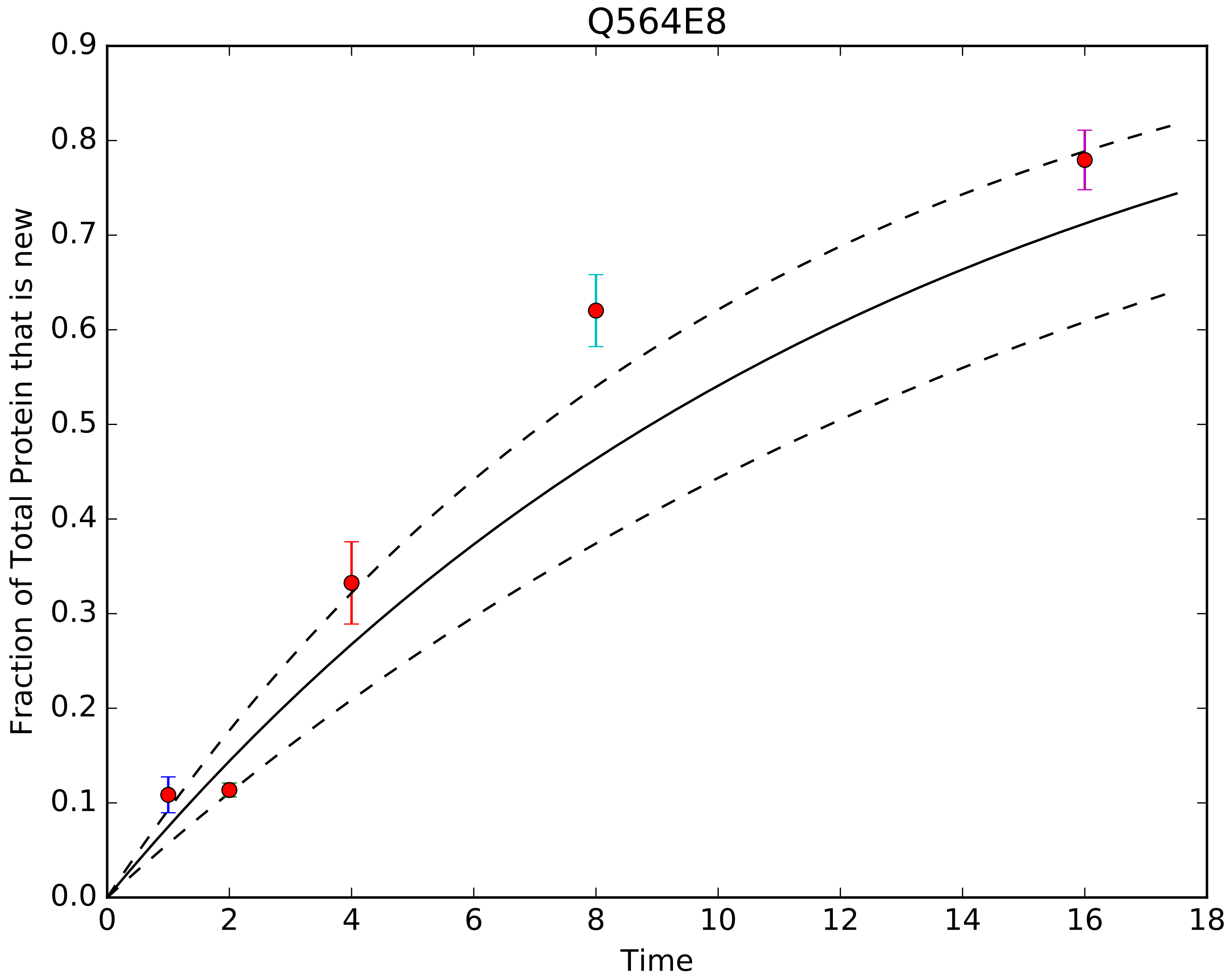

Q564E8
Fraction of Total Protein that is new
Time
0.0
0.1
0.2
0.3
0.4
0.5
0.6
0.7
0.8
0.9
0
2
4
6
8
10
12
14
16
18

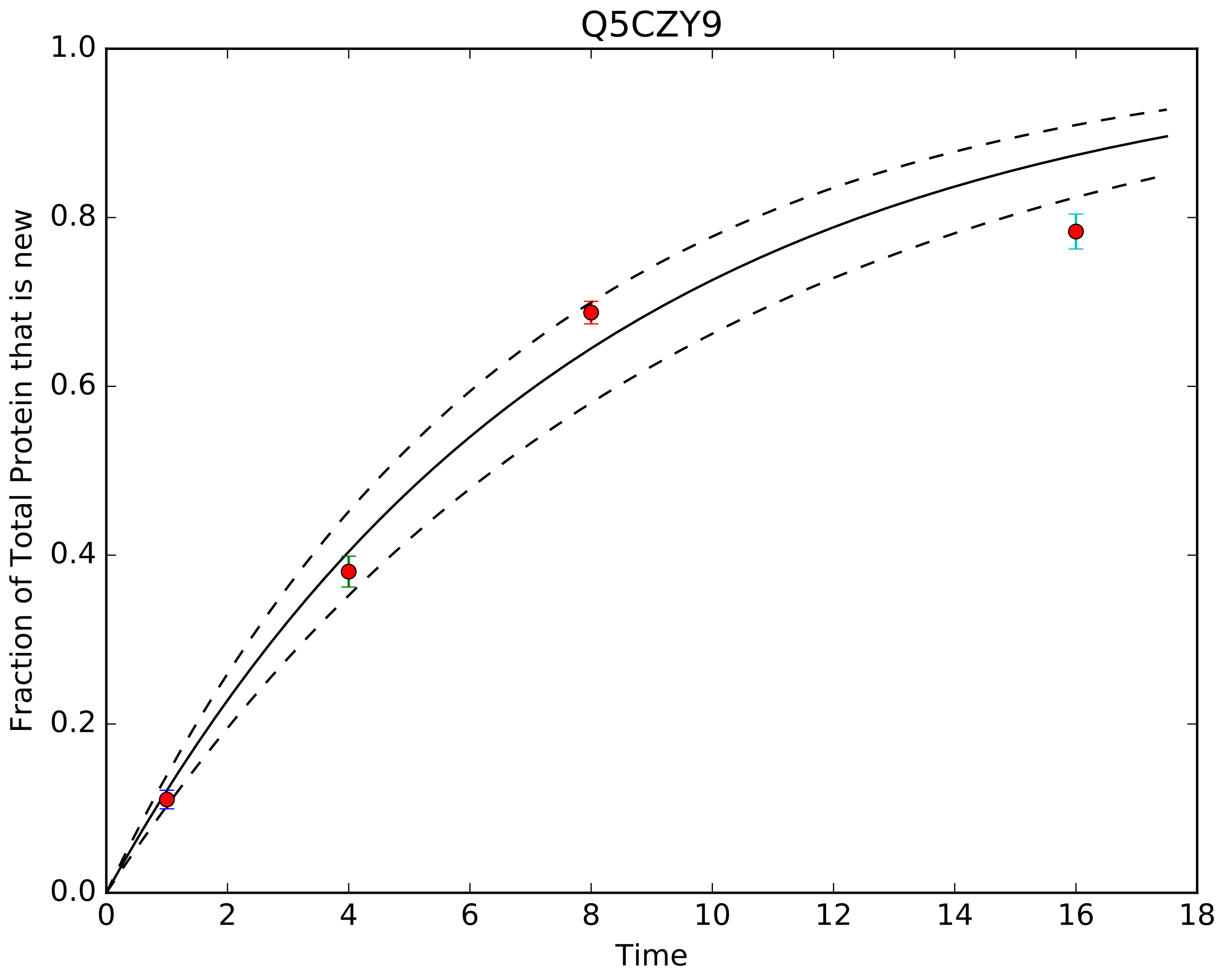

Q5CZY9
1.0
0.8
0.6
0.4
0.2
0.0
0
2
4
6
8
10
12
14
16
18
Time
Fraction of Total Protein that is new

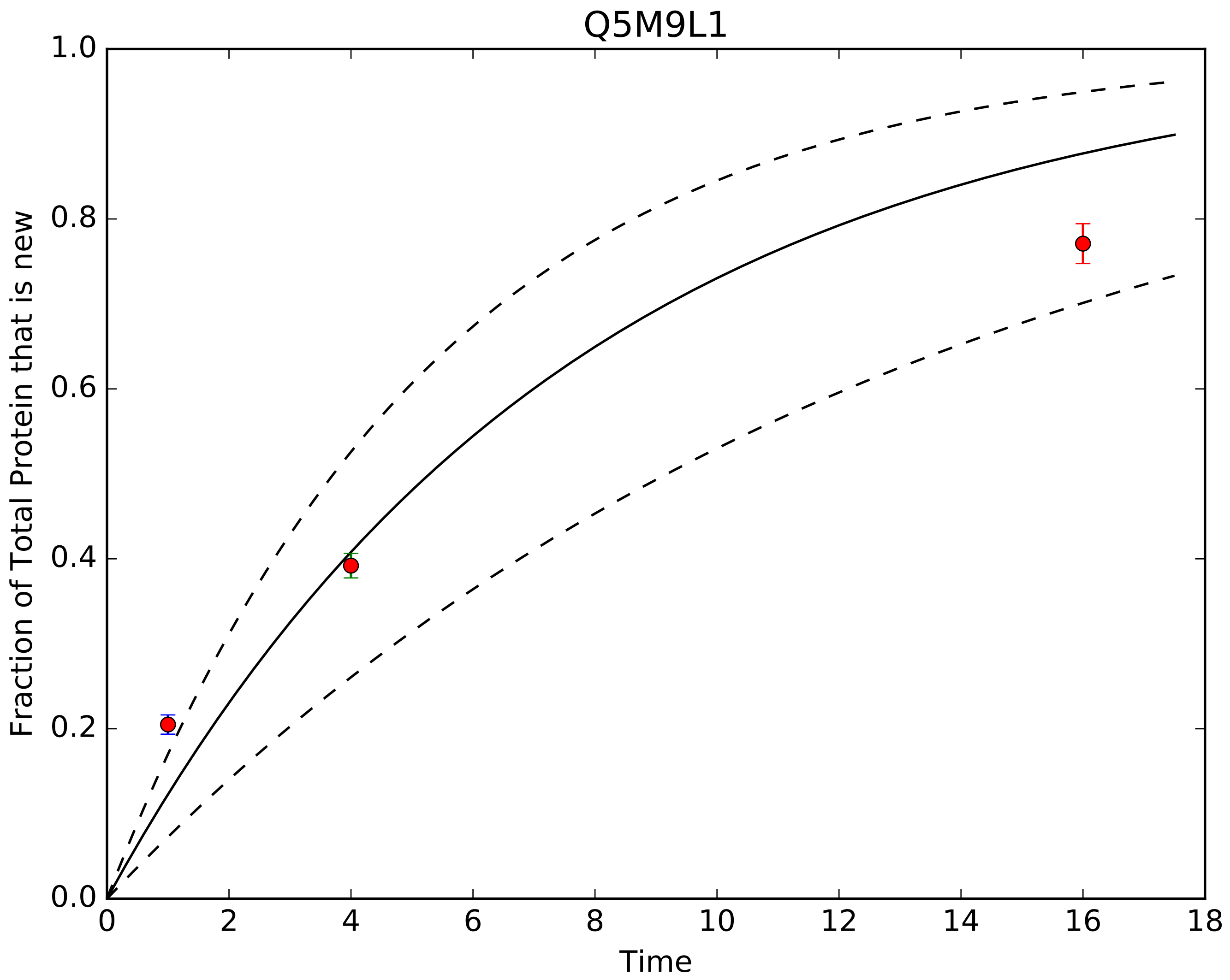
Q5M9L1
Fraction of Total Protein that is new
Time
0.0
0.2
0.4
0.6
0.8
1.0
0
2
4
6
8
10
12
14
16
18

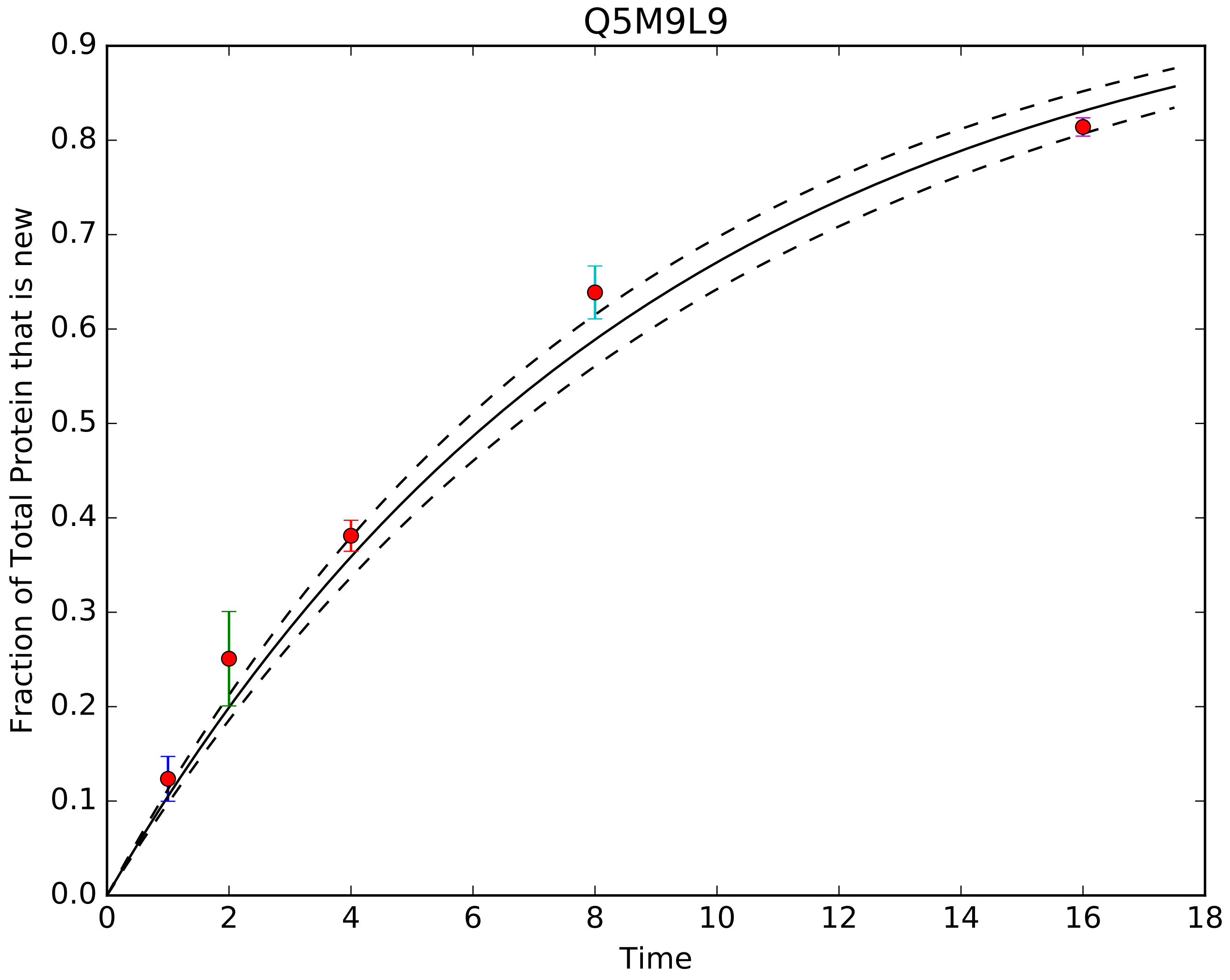

Q5M9L9
Fraction of Total Protein that is new
Time
0.0
0.1
0.2
0.3
0.4
0.5
0.6
0.7
0.8
0.9
0
2
4
6
8
10
12
14
16
18

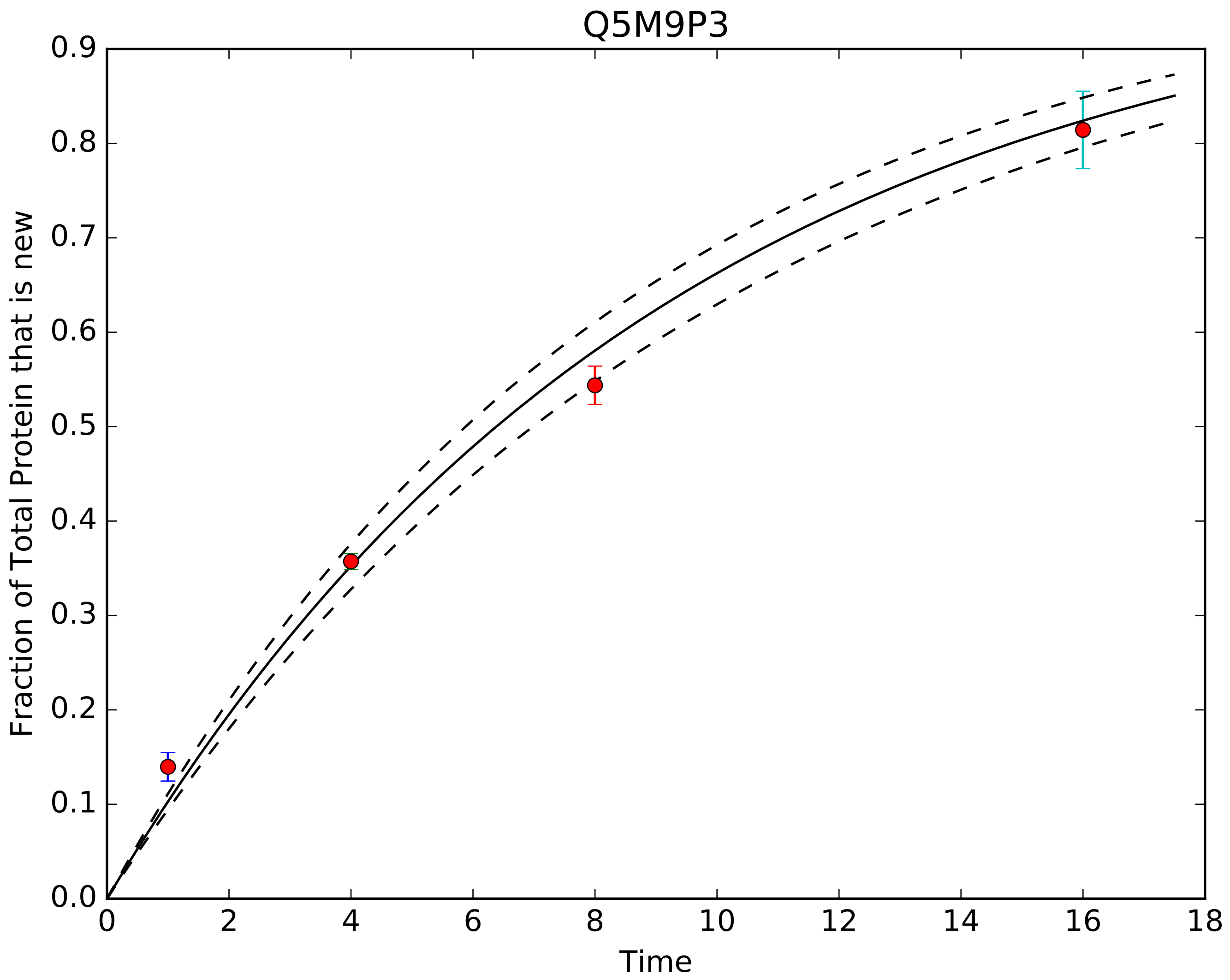
Q5M9P3
Fraction of Total Protein that is new
Time
0.0
0.1
0.2
0.3
0.4
0.5
0.6
0.7
0.8
0.9
0
2
4
6
8
10
12
14
16
18

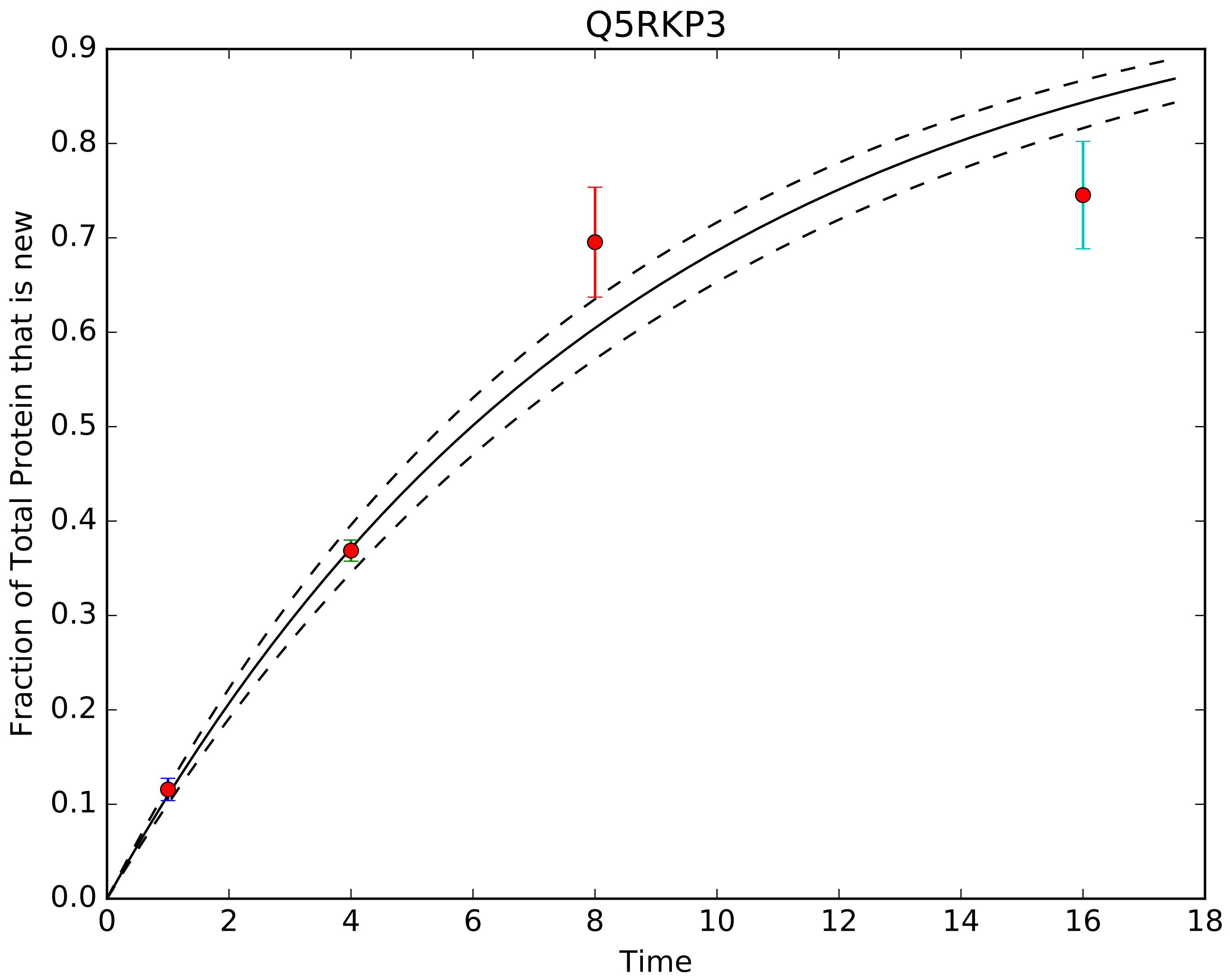

Q5RKP3
Fraction of Total Protein that is new
Time
0.0
0.1
0.2
0.3
0.4
0.5
0.6
0.7
0.8
0.9
0
2
4
6
8
10
12
14
16
18

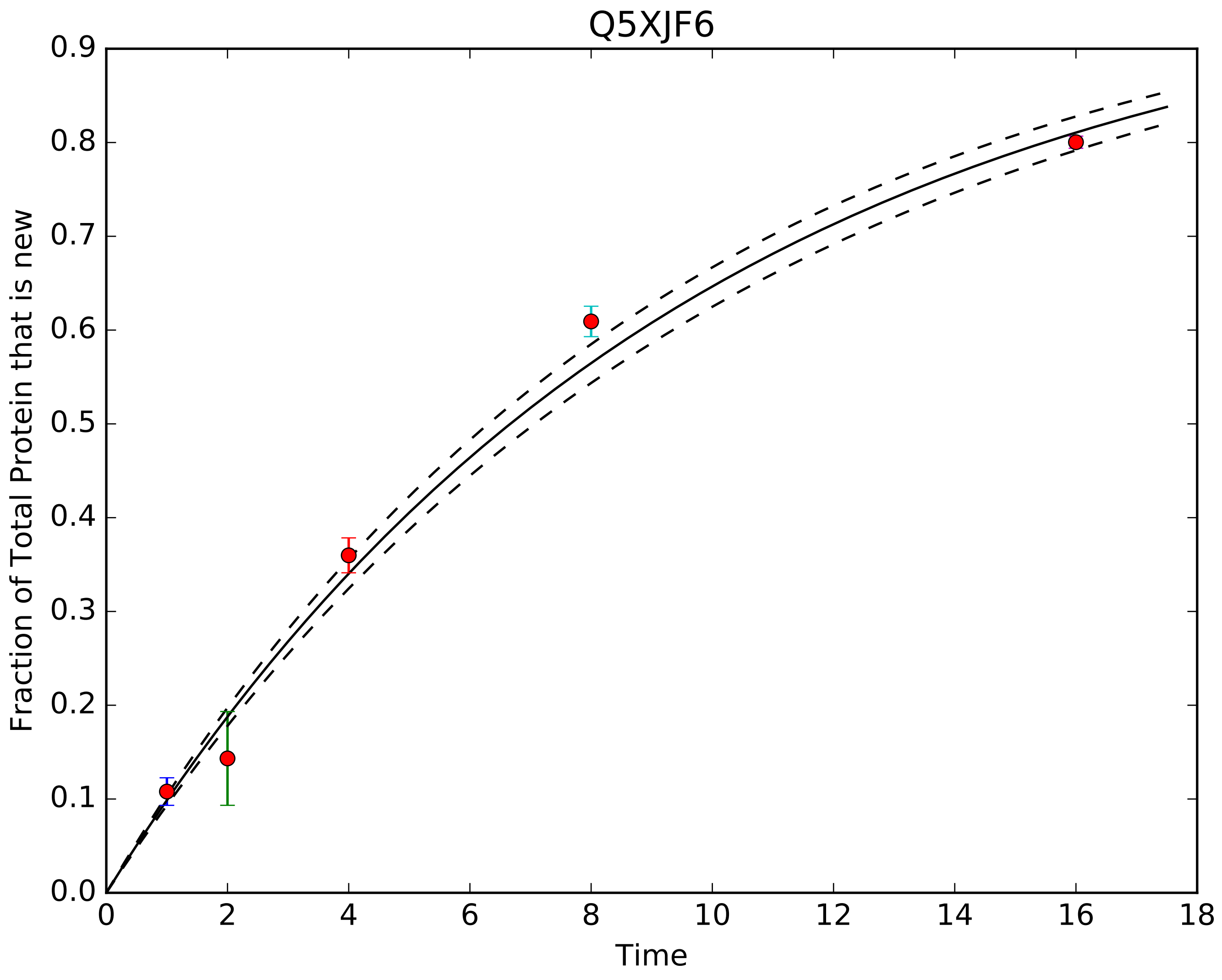
Q5XJF6
Fraction of Total Protein that is new
Time
0.0
0.1
0.2
0.3
0.4
0.5
0.6
0.7
0.8
0.9
0
2
4
6
8
10
12
14
16
18

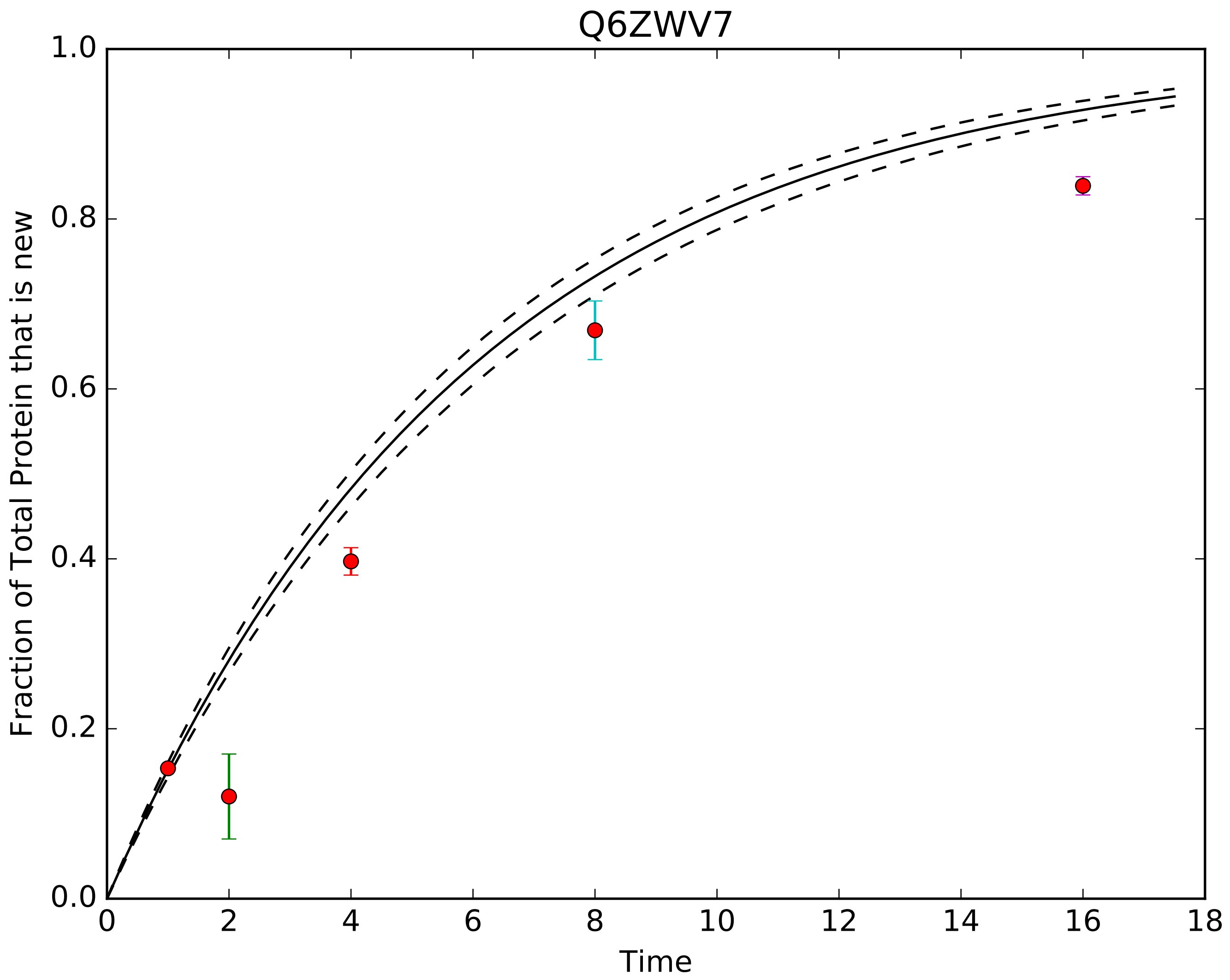
Q6ZWV7
Fraction of Total Protein that is new
Time
0.0
0.2
0.4
0.6
0.8
1.0
0
2
4
6
8
10
12
14
16
18

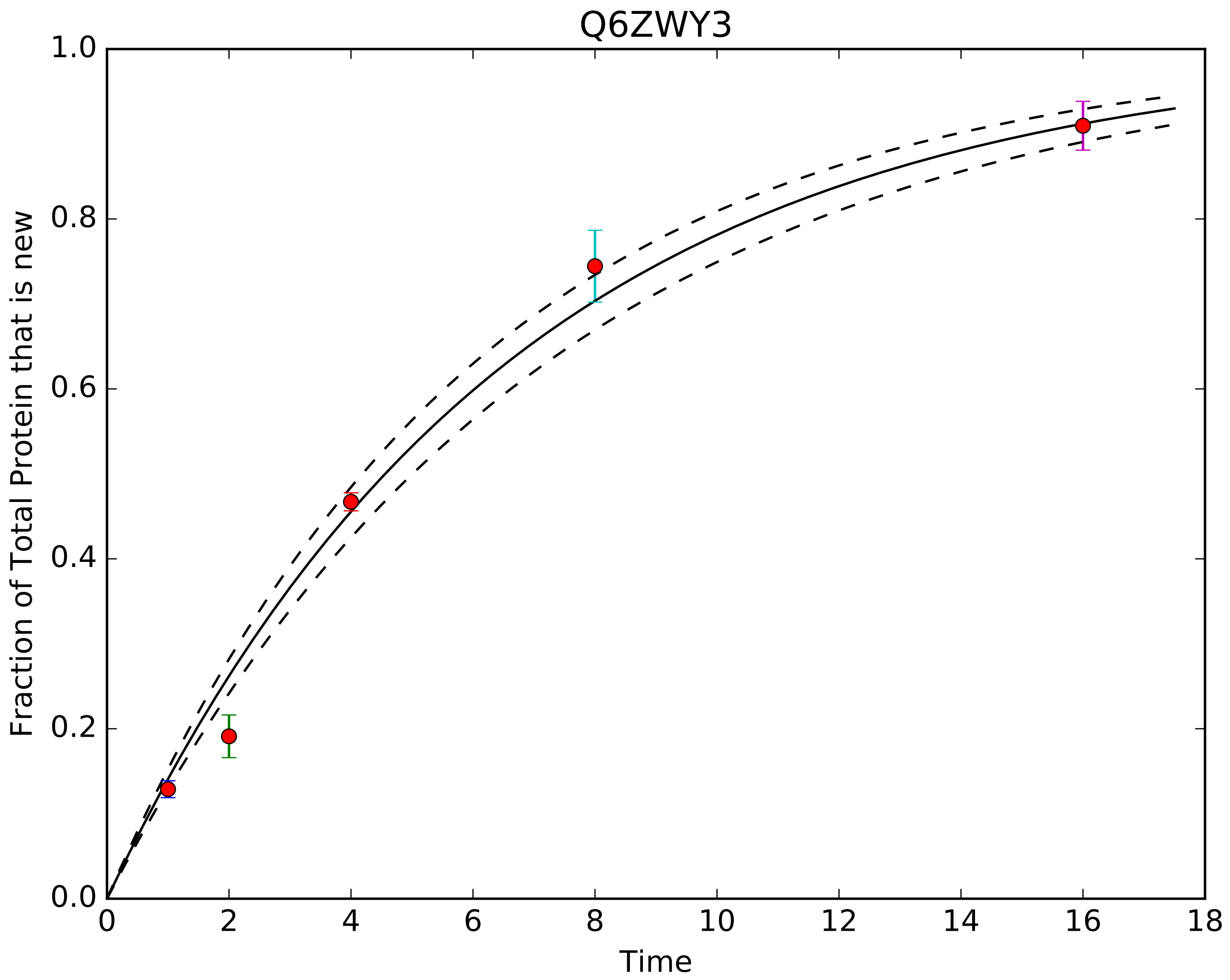
Q6ZWY3
Fraction of Total Protein that is new
Time
0.0
0.2
0.4
0.6
0.8
1.0
0
2
4
6
8
10
12
14
16
18

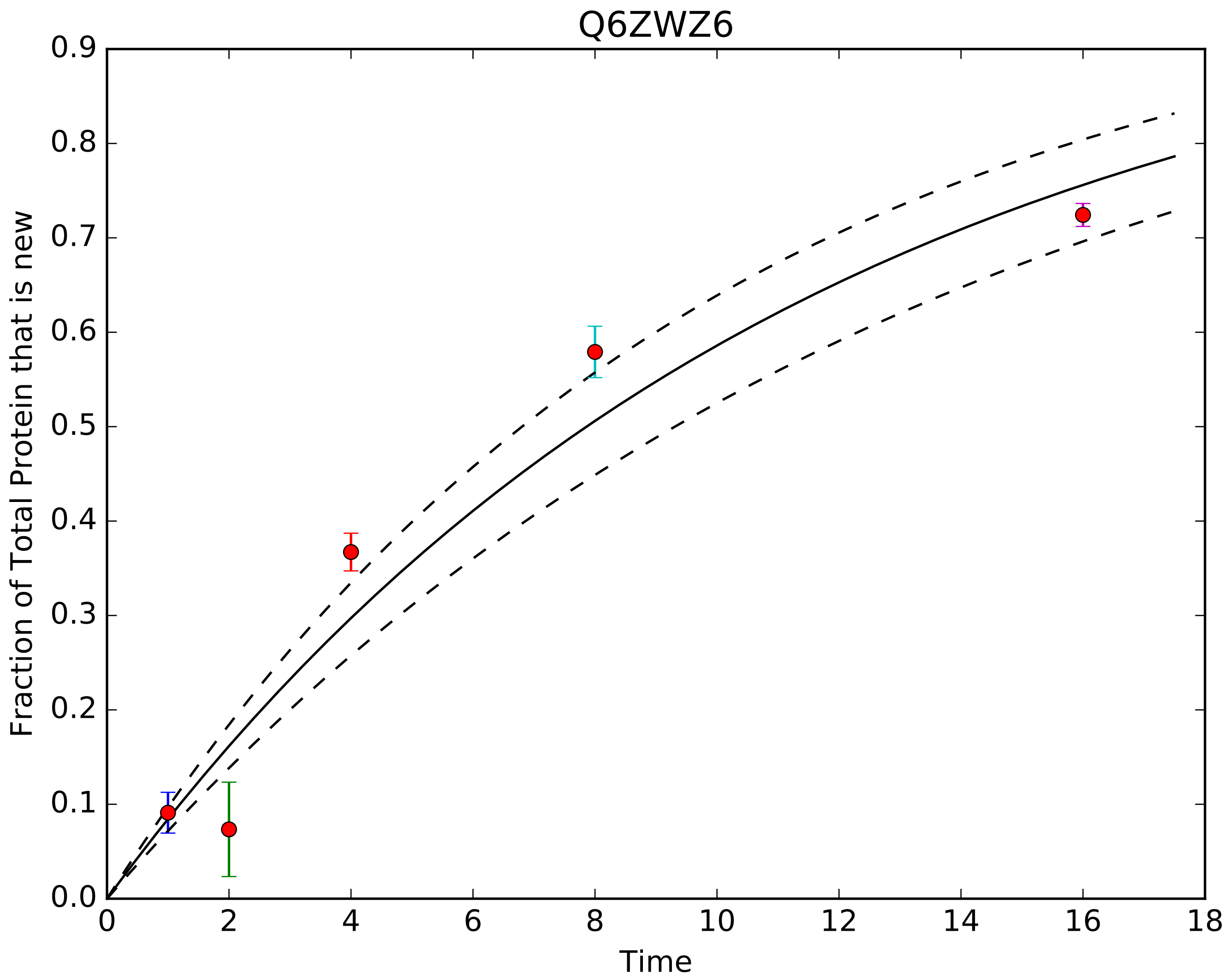
Q6ZWZ6
Fraction of Total Protein that is new
Time
0.0
0.1
0.2
0.3
0.4
0.5
0.6
0.7
0.8
0.9
0
2
4
6
8
10
12
14
16
18

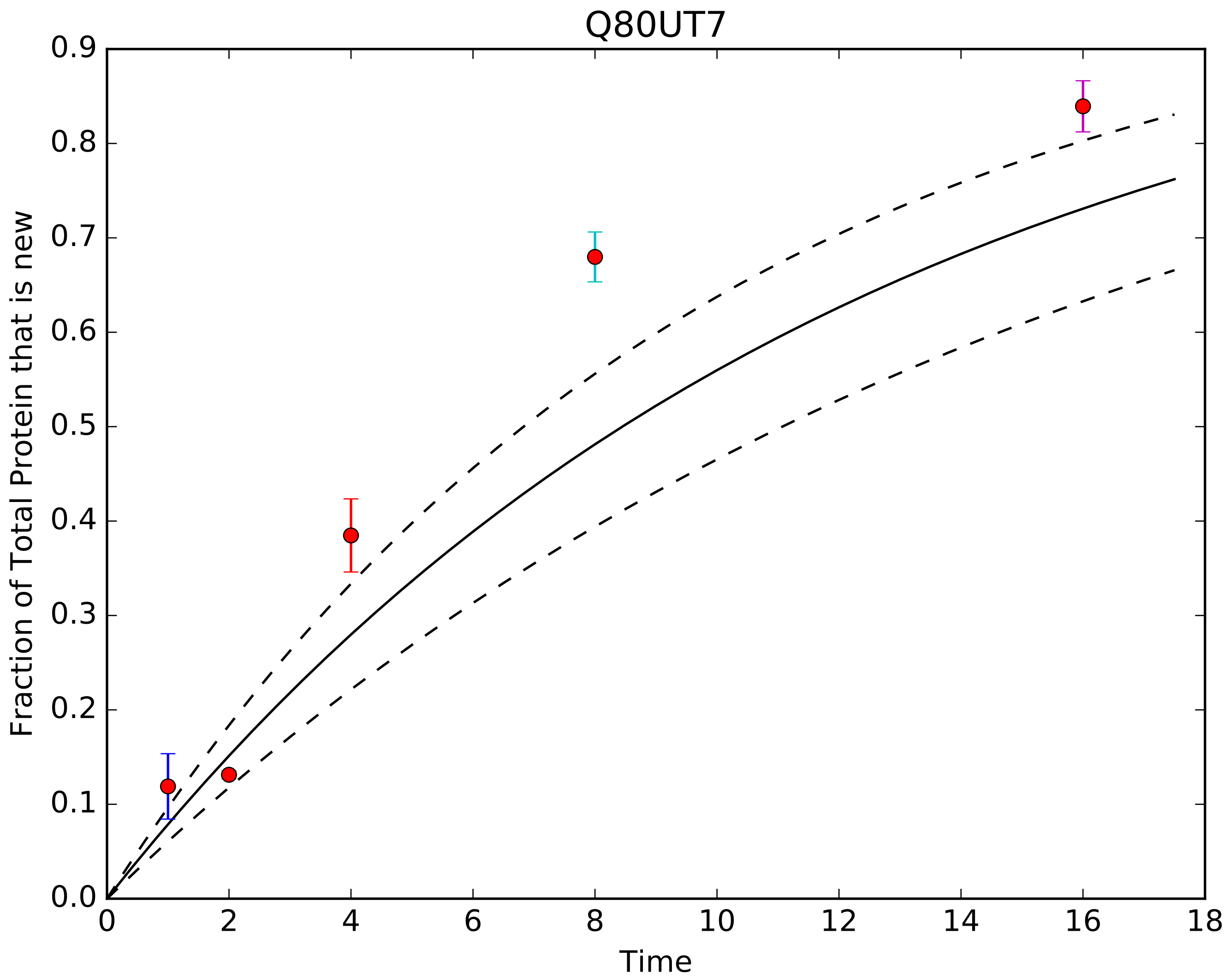
Q80UT7
Fraction of Total Protein that is new
Time
0.0
0.1
0.2
0.3
0.4
0.5
0.6
0.7
0.8
0.9
0
2
4
6
8
10
12
14
16
18

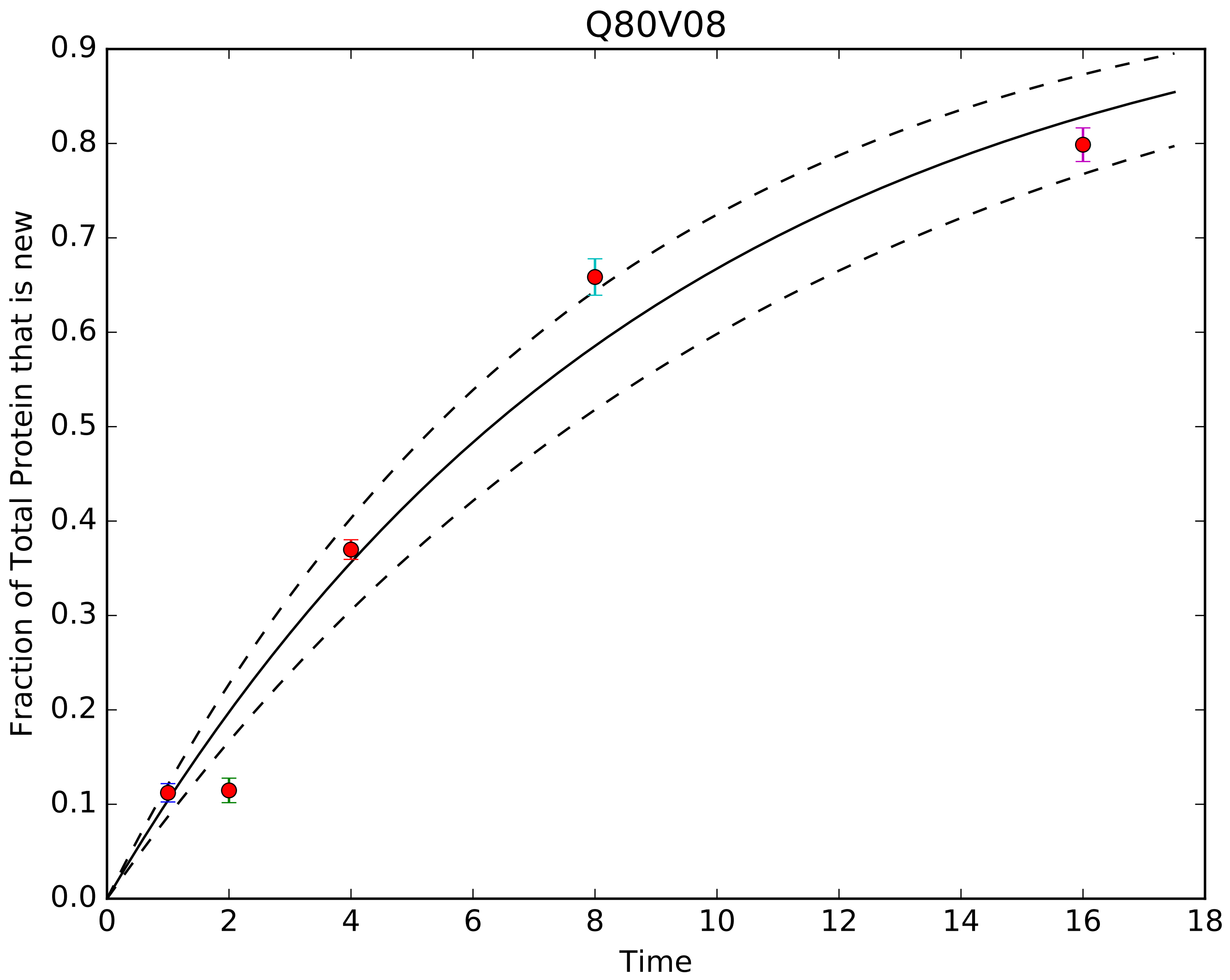
Q80V08
Fraction of Total Protein that is new
Time
0.0
0.1
0.2
0.3
0.4
0.5
0.6
0.7
0.8
0.9
0
2
4
6
8
10
12
14
16
18

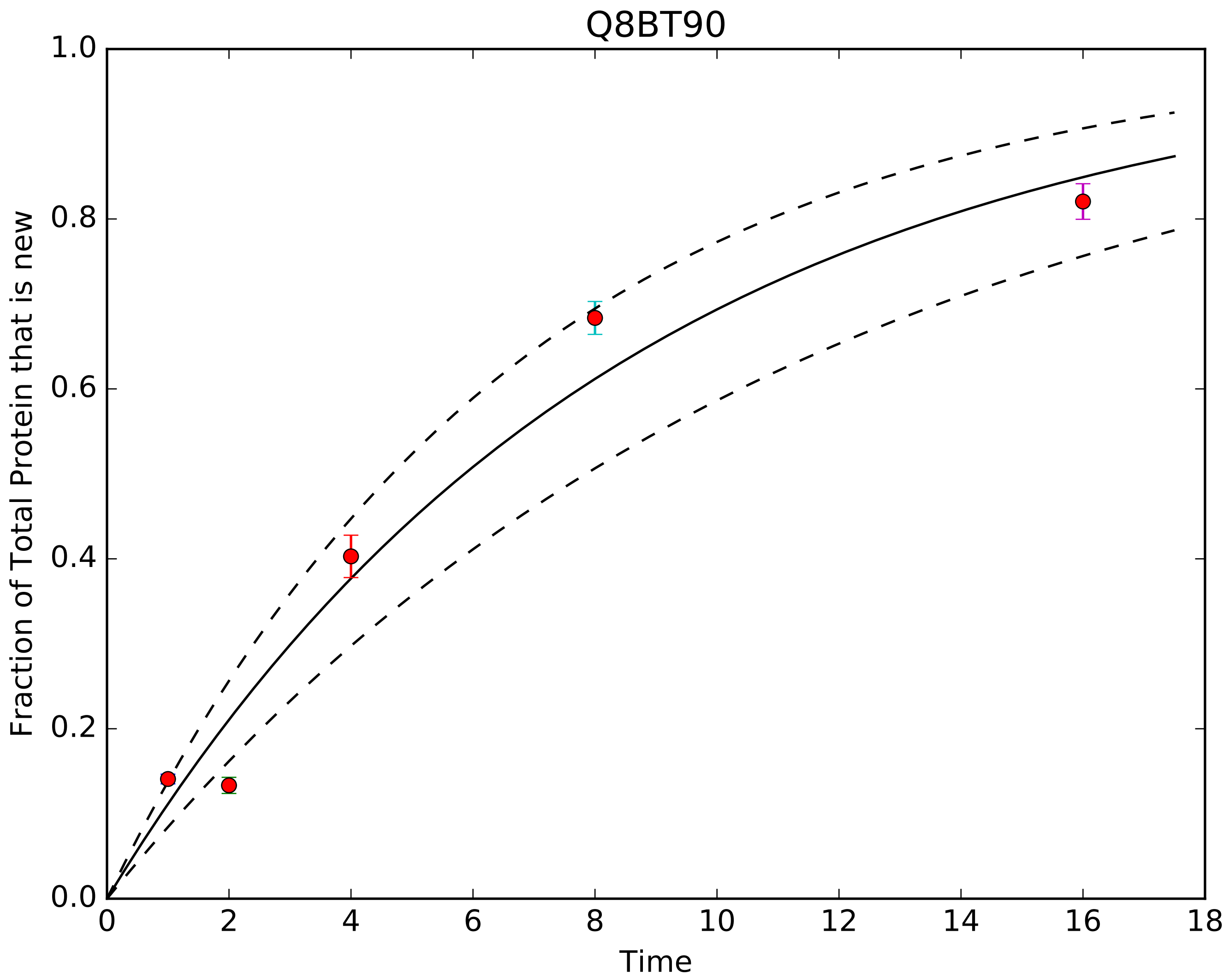
Q8BT90
Fraction of Total Protein that is new
Time
0.0
0.2
0.4
0.6
0.8
1.0
0
2
4
6
8
10
12
14
16
18

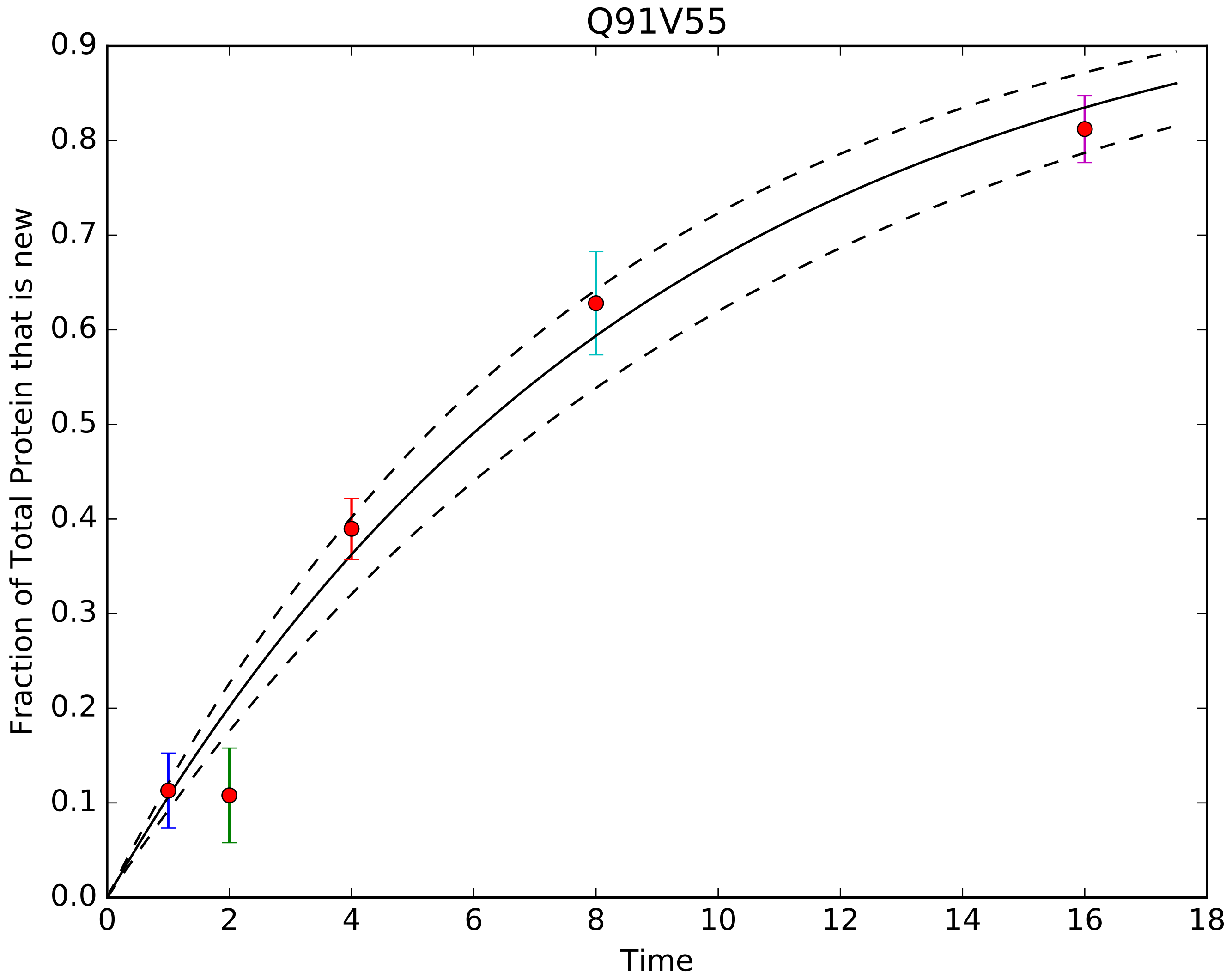
Q91V55
Fraction of Total Protein that is new
Time
0.0
0.1
0.2
0.3
0.4
0.5
0.6
0.7
0.8
0.9
0
2
4
6
8
10
12
14
16
18

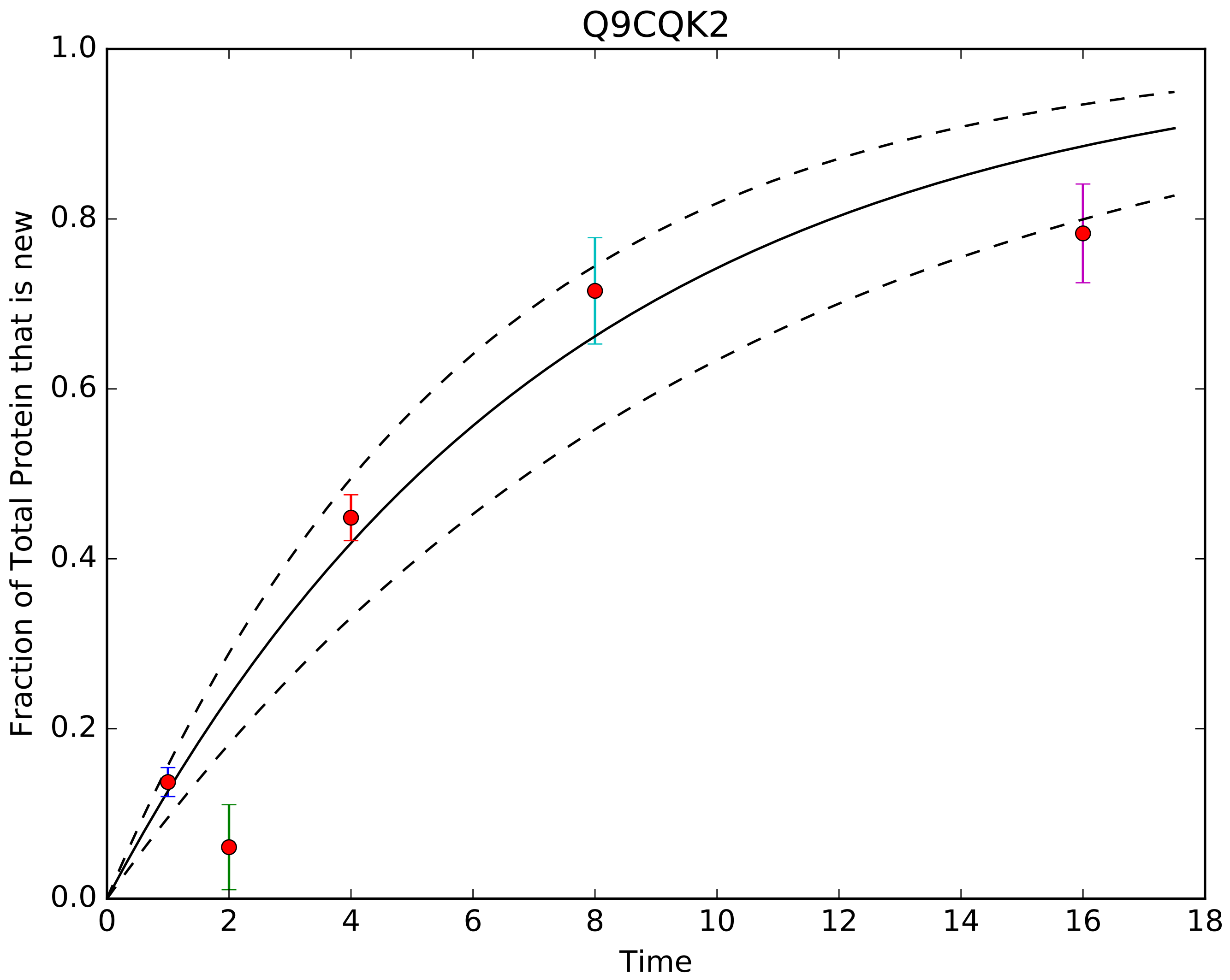
Q9CQK2
Fraction of Total Protein that is new
Time
0.0
0.2
0.4
0.6
0.8
1.0
0
2
4
6
8
10
12
14
16
18

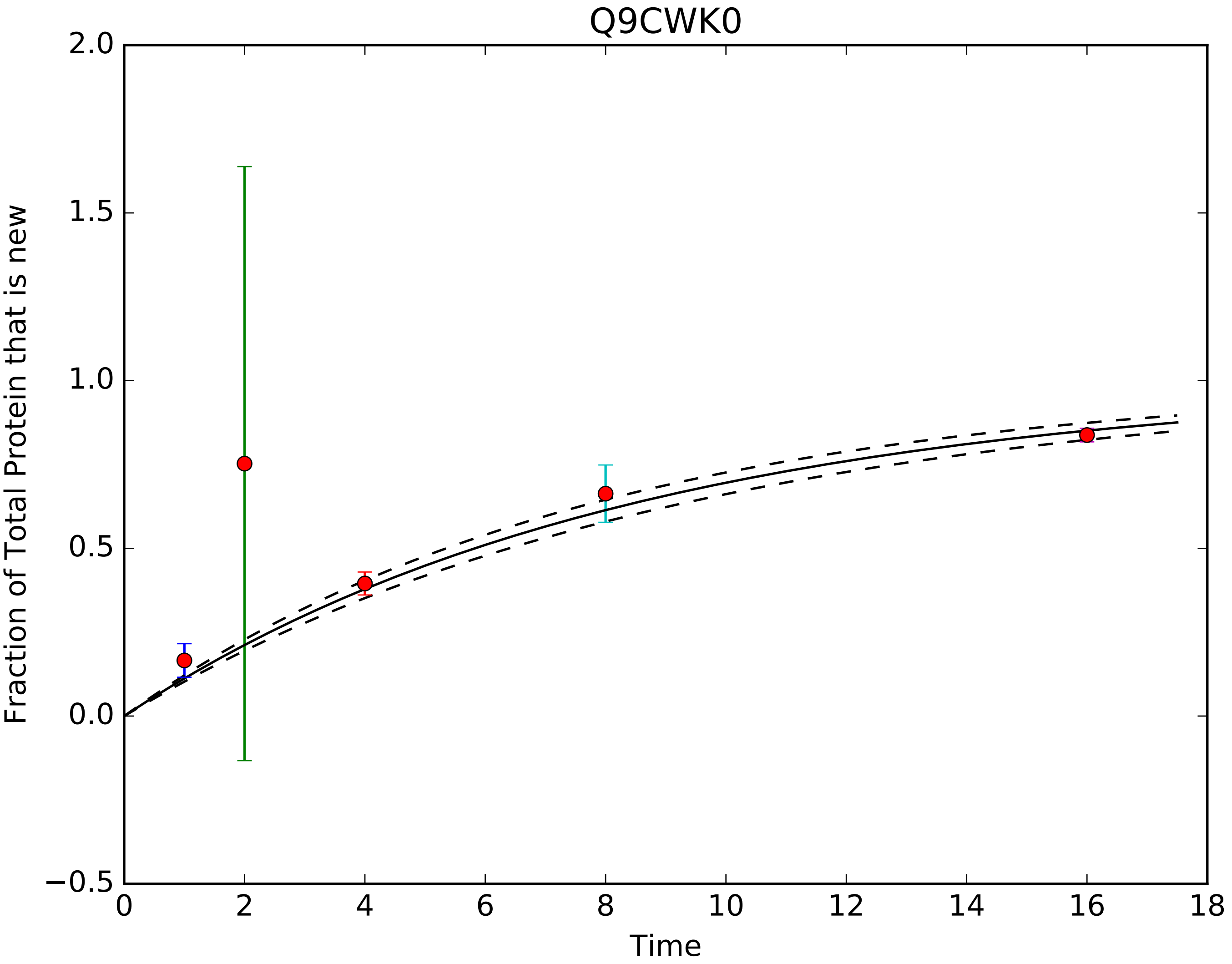

Q9CWK0
Fraction of Total Protein that is new
Time
2.0
1.5
1.0
0.5
0.0
−0.5
0
2
4
6
8
10
12
14
16
18

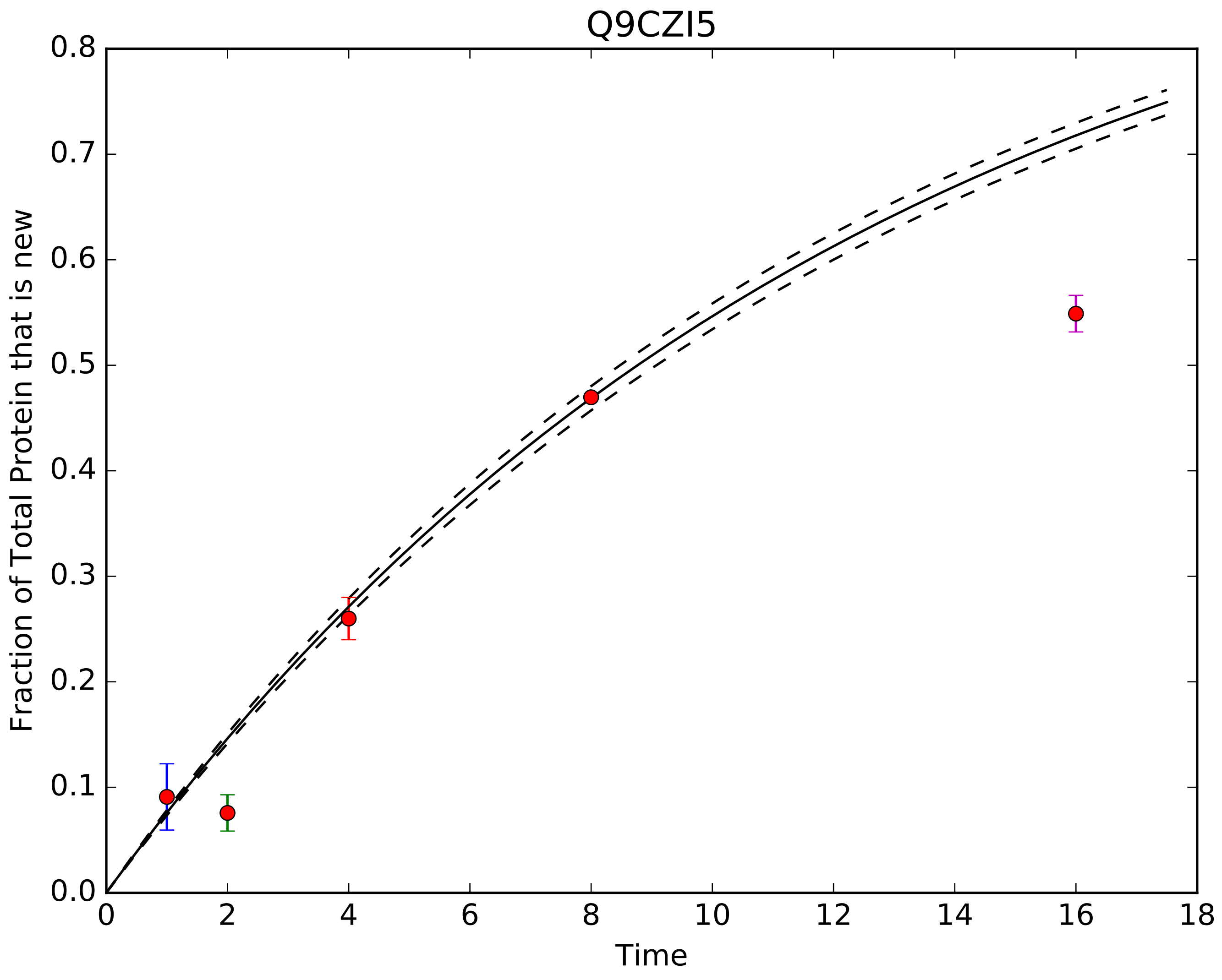
Q9CZI5
Fraction of Total Protein that is new
Time
0.0
0.1
0.2
0.3
0.4
0.5
0.6
0.7
0.8
0
2
4
6
8
10
12
14
16
18

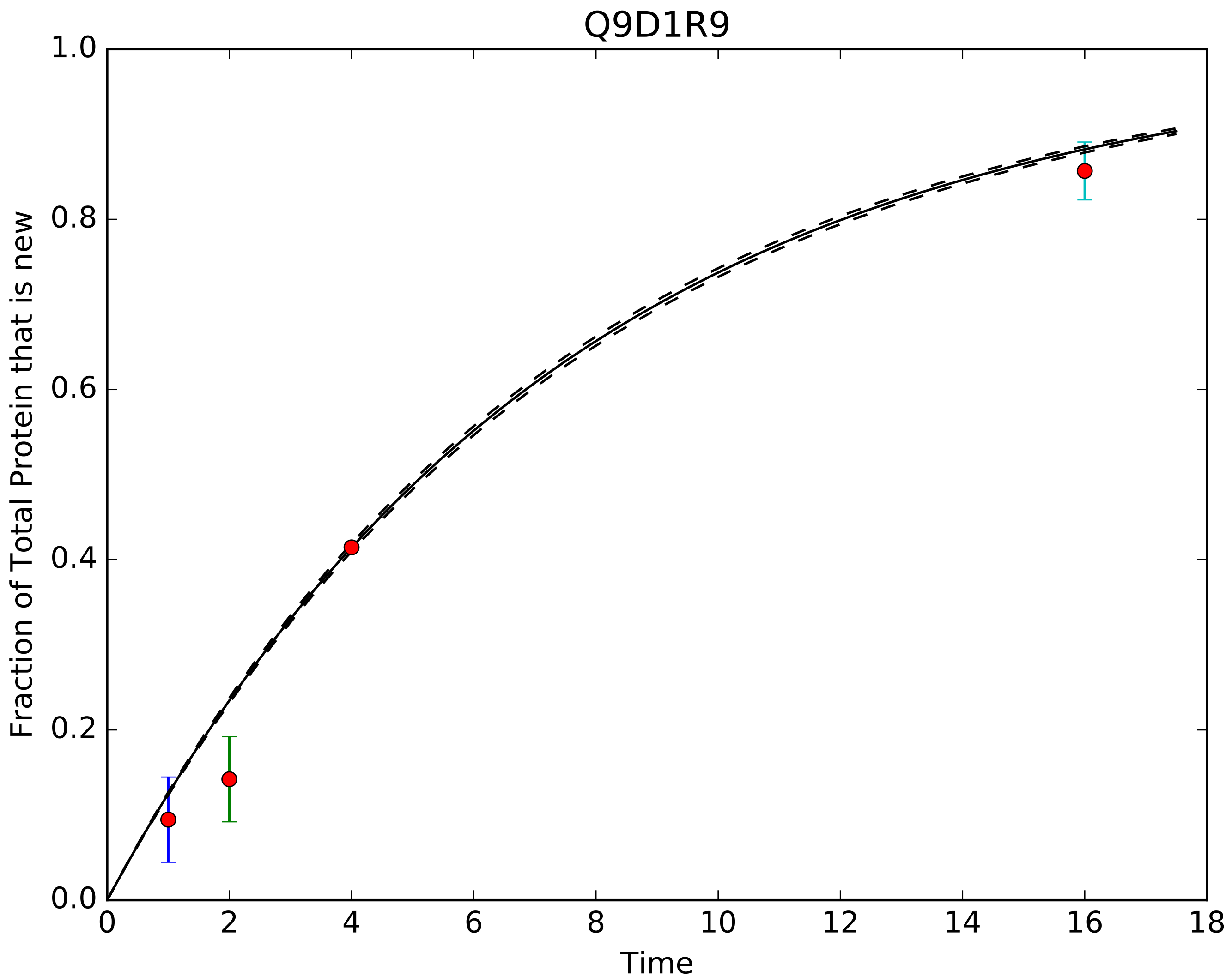

Q9D1R9
Fraction of Total Protein that is new
Time
0.0
0.2
0.4
0.6
0.8
1.0
0
2
4
6
8
10
12
14
16
18

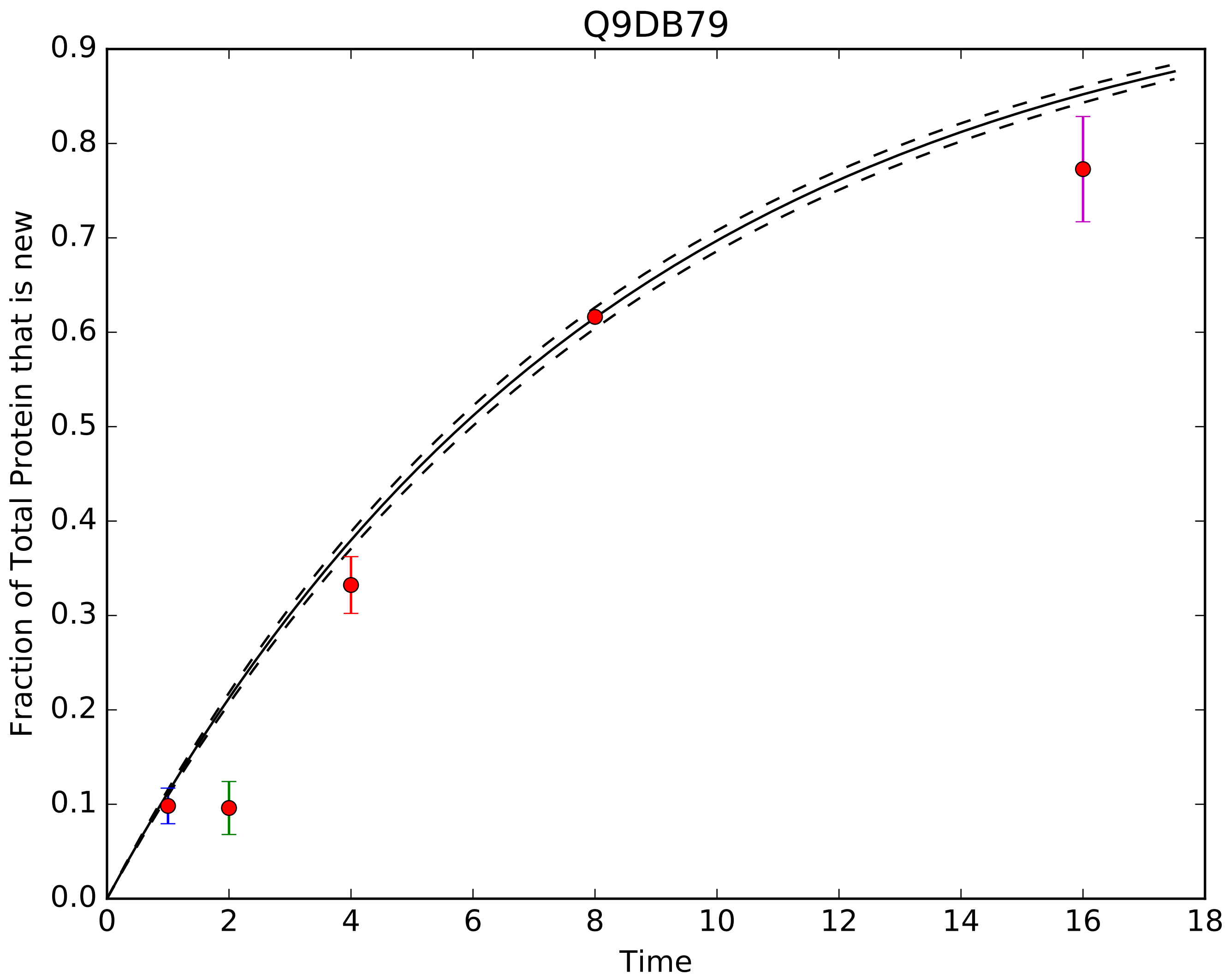

Q9DB79
Fraction of Total Protein that is new
Time
0.0
0.1
0.2
0.3
0.4
0.5
0.6
0.7
0.8
0.9
0
2
4
6
8
10
12
14
16
18

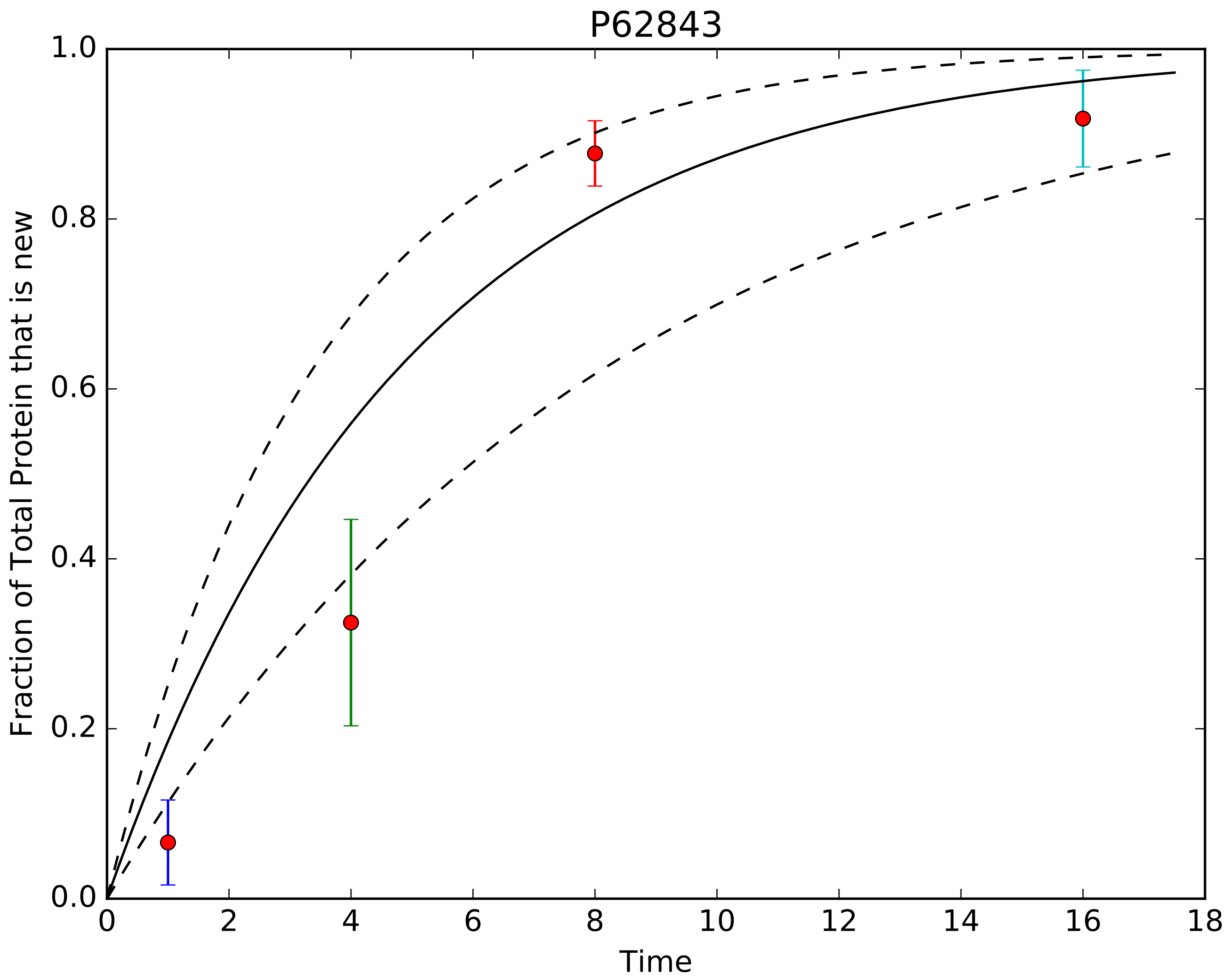
P62843
1.0
0.8
0.6
0.4
0.2
0.0
0
2
4
6
8
10
12
14
16
18
Time
Fraction of Total Protein that is new
