# Supplementary material for: Mechanisms of In Vivo Ribosome Maintenance Change in Response to Nutrient Signals
Source: Mol Cell Proteomics. 2016 Dec 8;16(2):243–54. doi: 10.1074/mcp.M116.063255 (PMC5294211; doi:10.1074/mcp.M116.063255)
Supplement: Supplemental Data [file 10.1074_M116.063255_mcp.M116.063255-2.pdf]

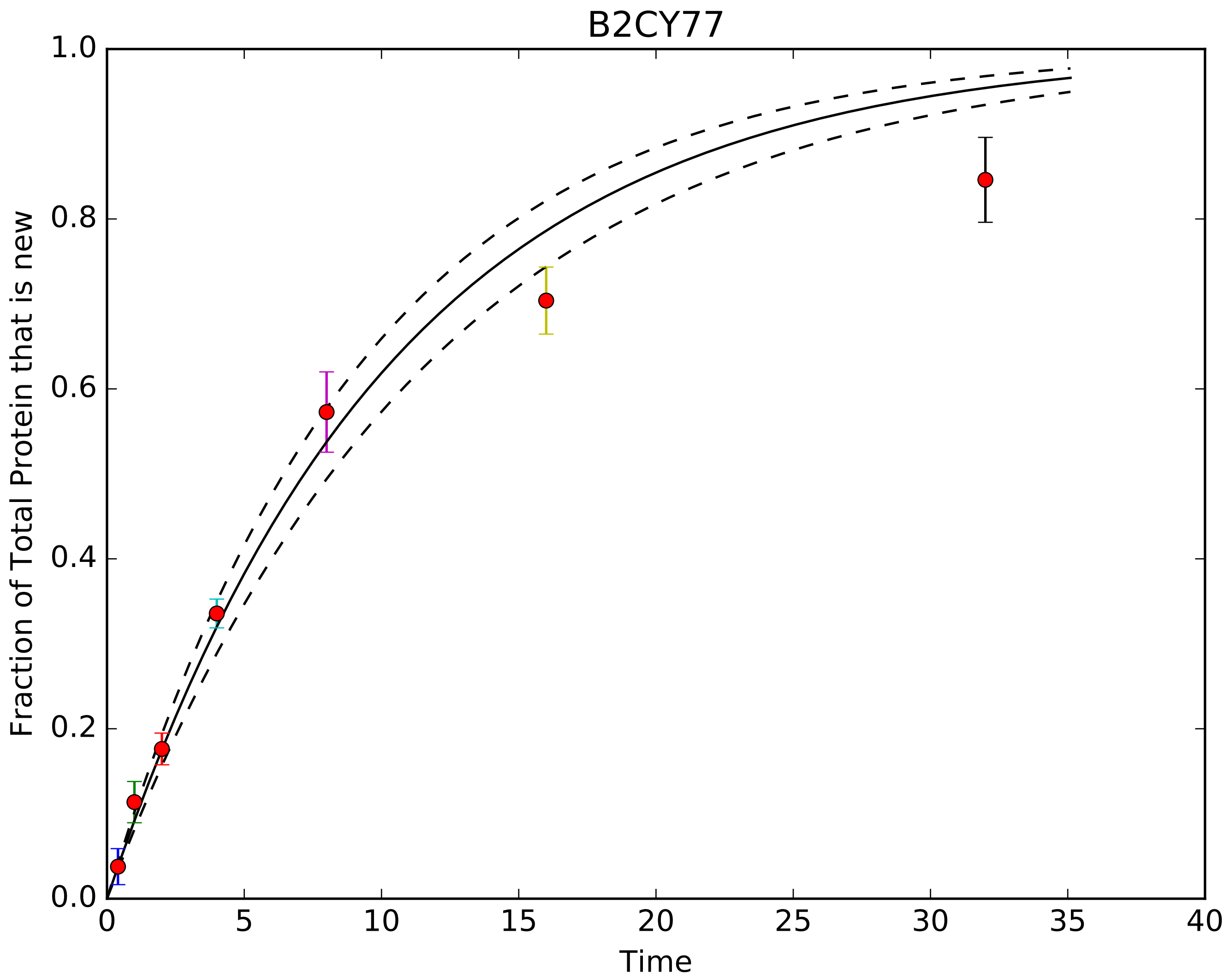

B2CY77
Fraction of Total Protein that is new
Time
0.0
0.2
0.4
0.6
0.8
1.0
0
5
10
15
20
25
30
35
40

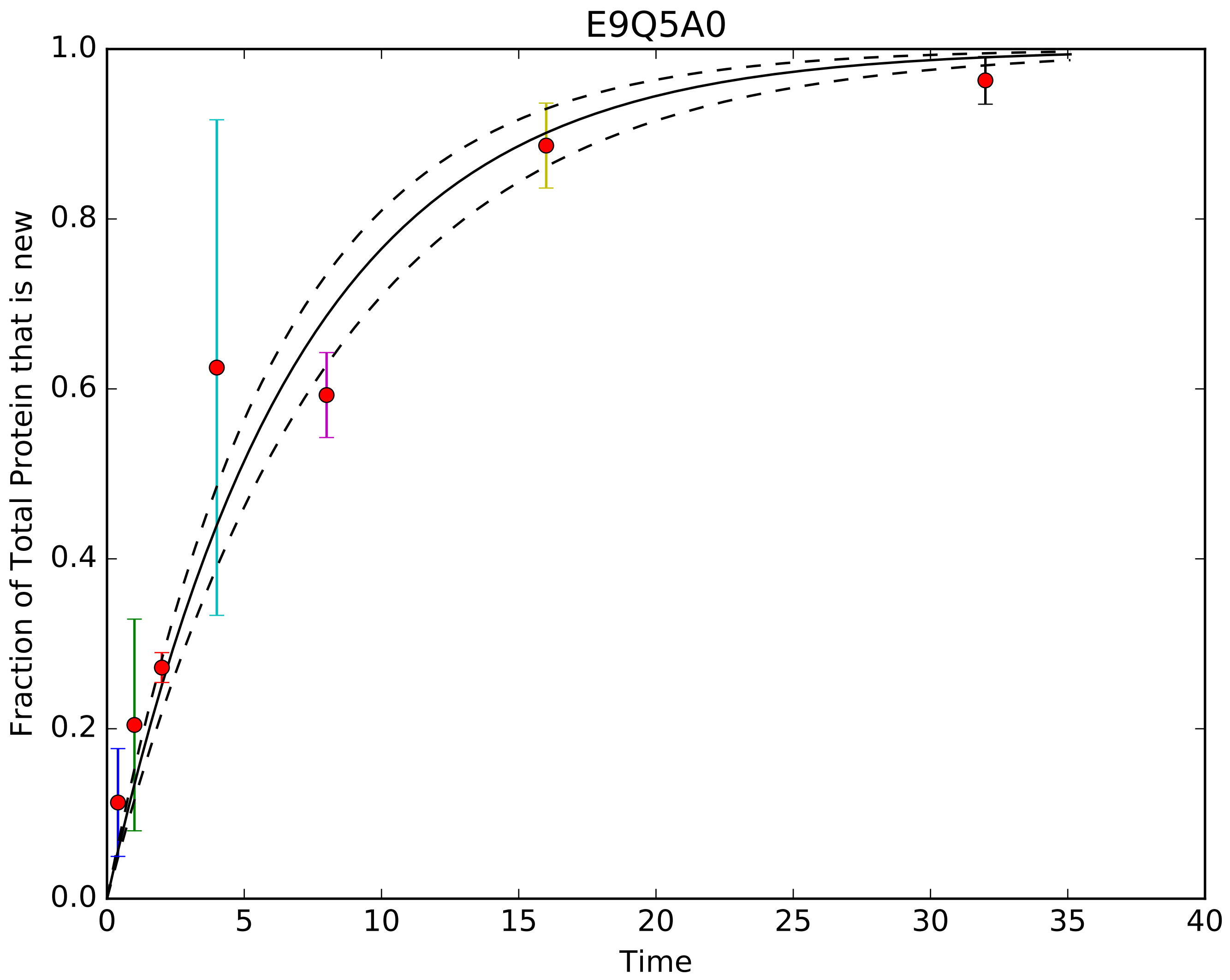
E9Q5A0
Fraction of Total Protein that is new
Time
0.0
0.2
0.4
0.6
0.8
1.0
0
5
10
15
20
25
30
35
40

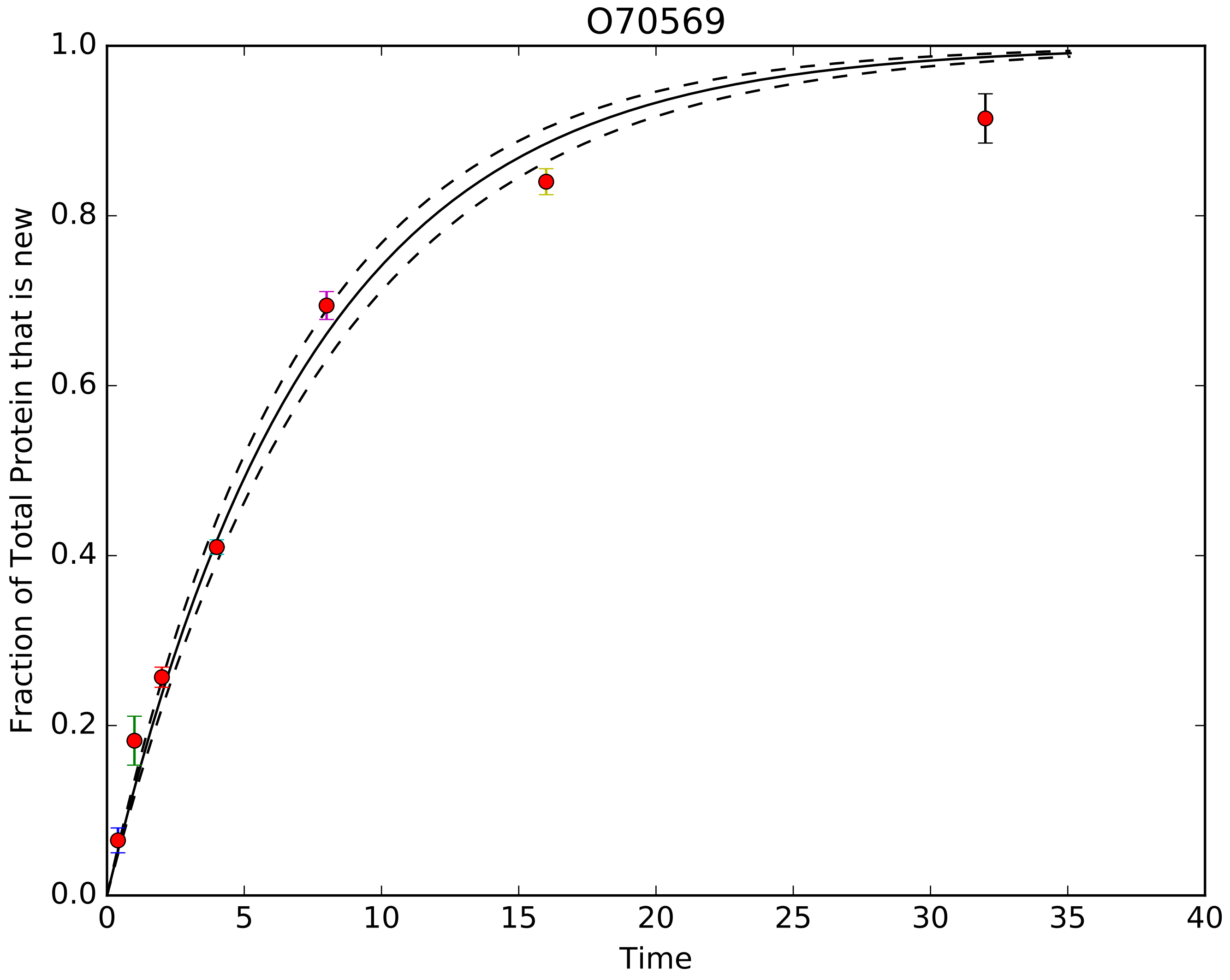

O70569
Fraction of Total Protein that is new
Time
0.0
0.2
0.4
0.6
0.8
1.0
0
5
10
15
20
25
30
35
40

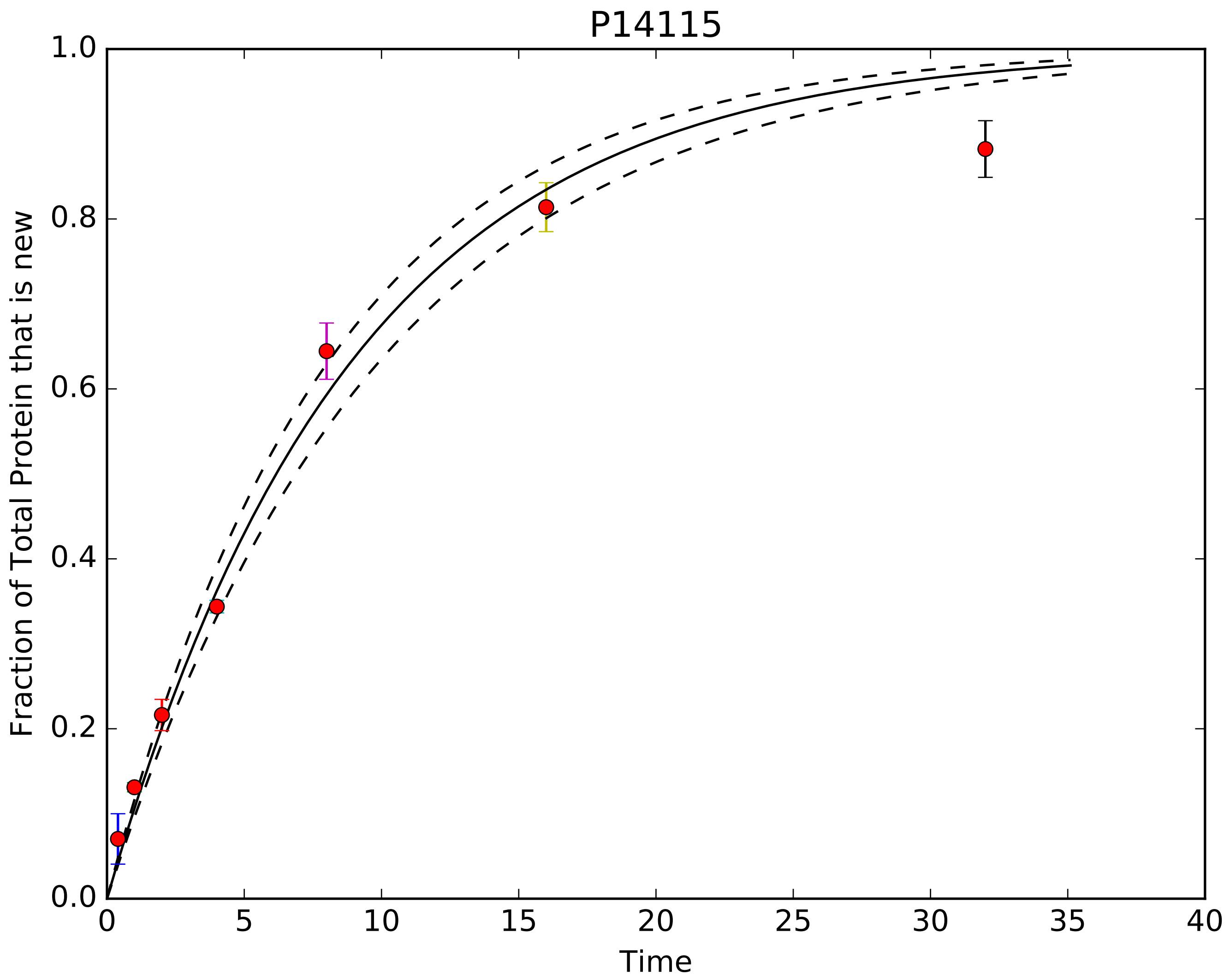

P14115
Fraction of Total Protein that is new
Time
0.0
0.2
0.4
0.6
0.8
1.0
0
5
10
15
20
25
30
35
40

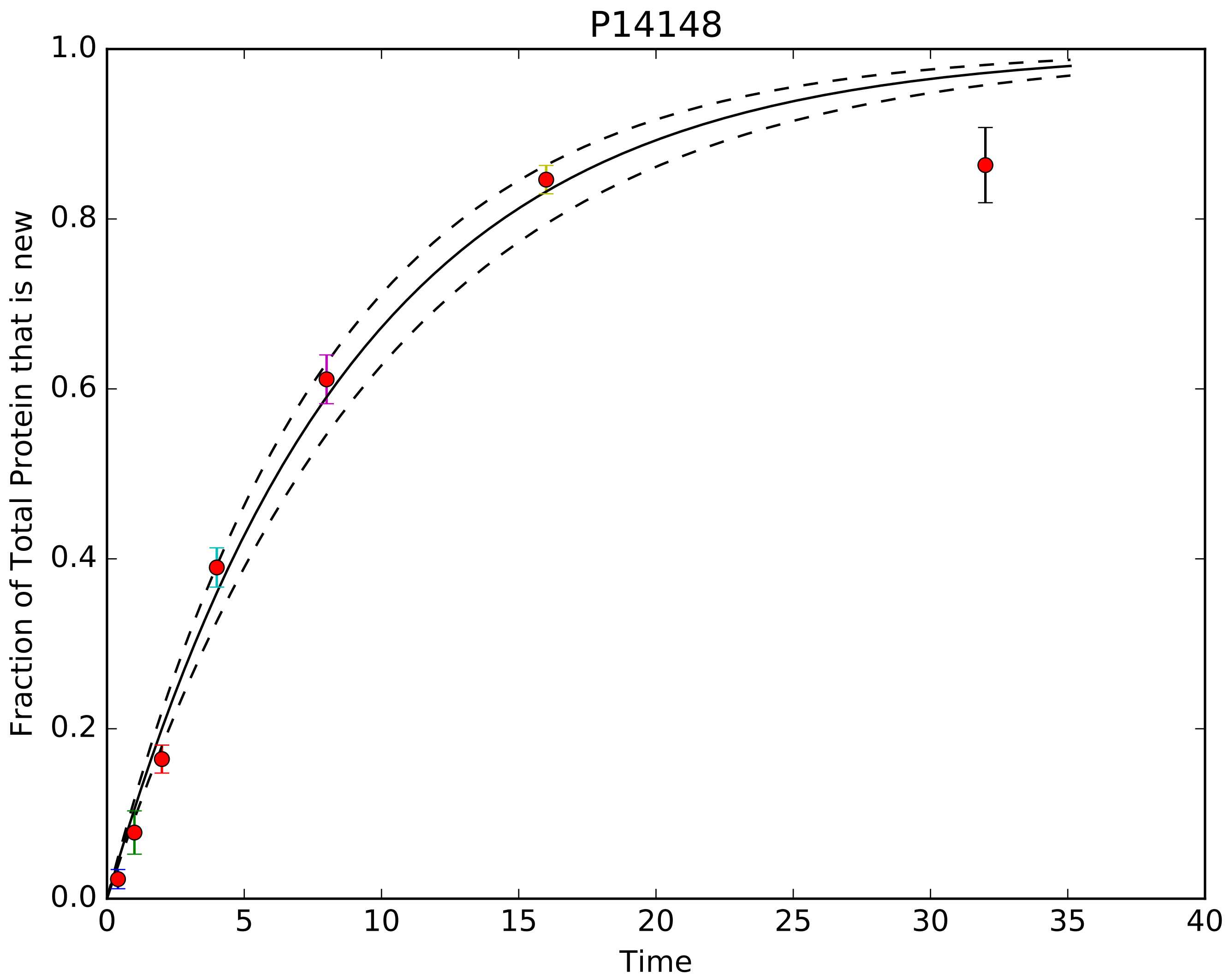
P14148
Fraction of Total Protein that is new
Time
0.0
0.2
0.4
0.6
0.8
1.0
0
5
10
15
20
25
30
35
40

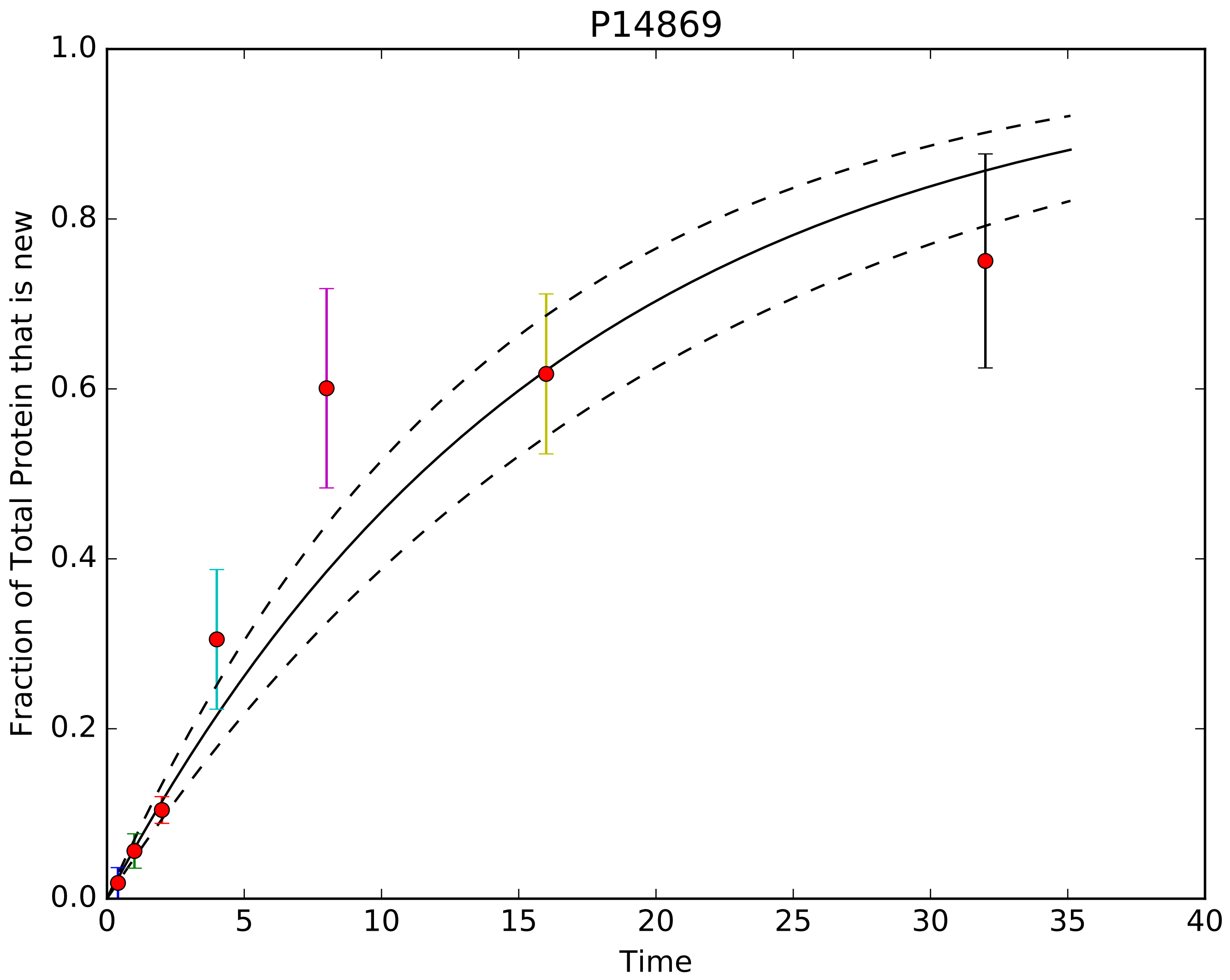

P14869
Fraction of Total Protein that is new
Time
0.0
0.2
0.4
0.6
0.8
1.0
0
5
10
15
20
25
30
35
40

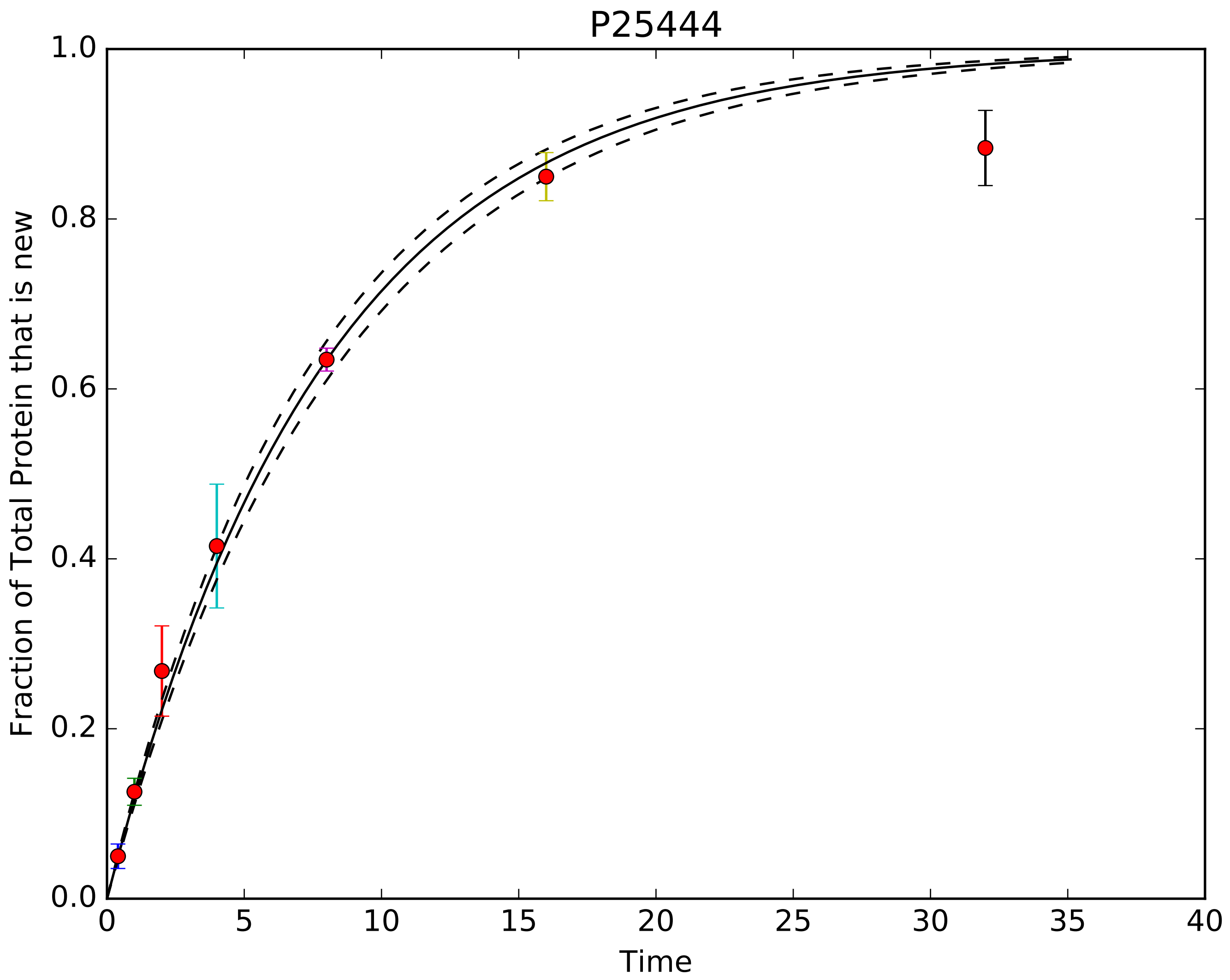

P25444
Fraction of Total Protein that is new
Time
0.0
0.2
0.4
0.6
0.8
1.0
0
5
10
15
20
25
30
35
40

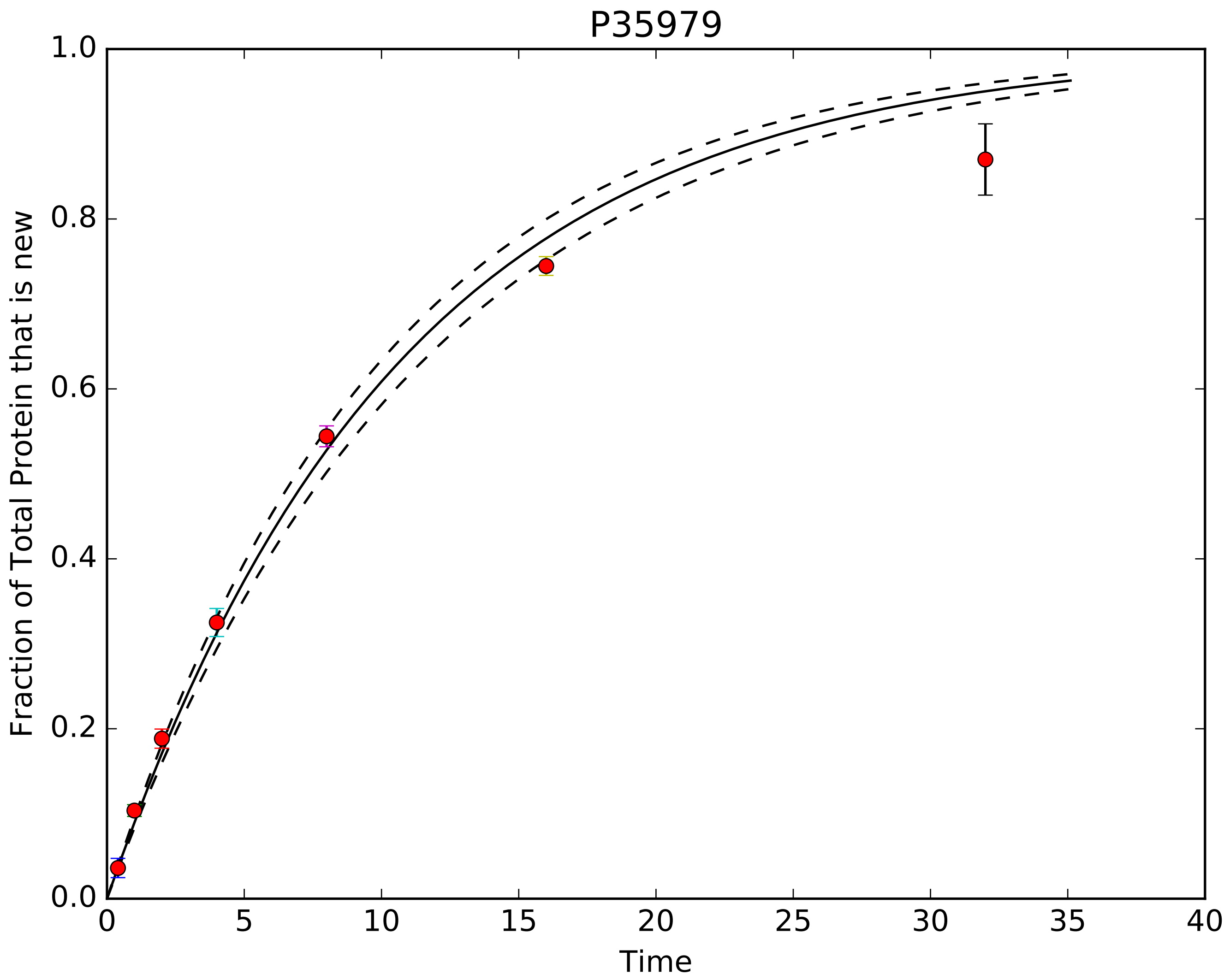
P35979
Fraction of Total Protein that is new
Time
1.0
0.8
0.6
0.4
0.2
0.0
0
5
10
15
20
25
30
35
40

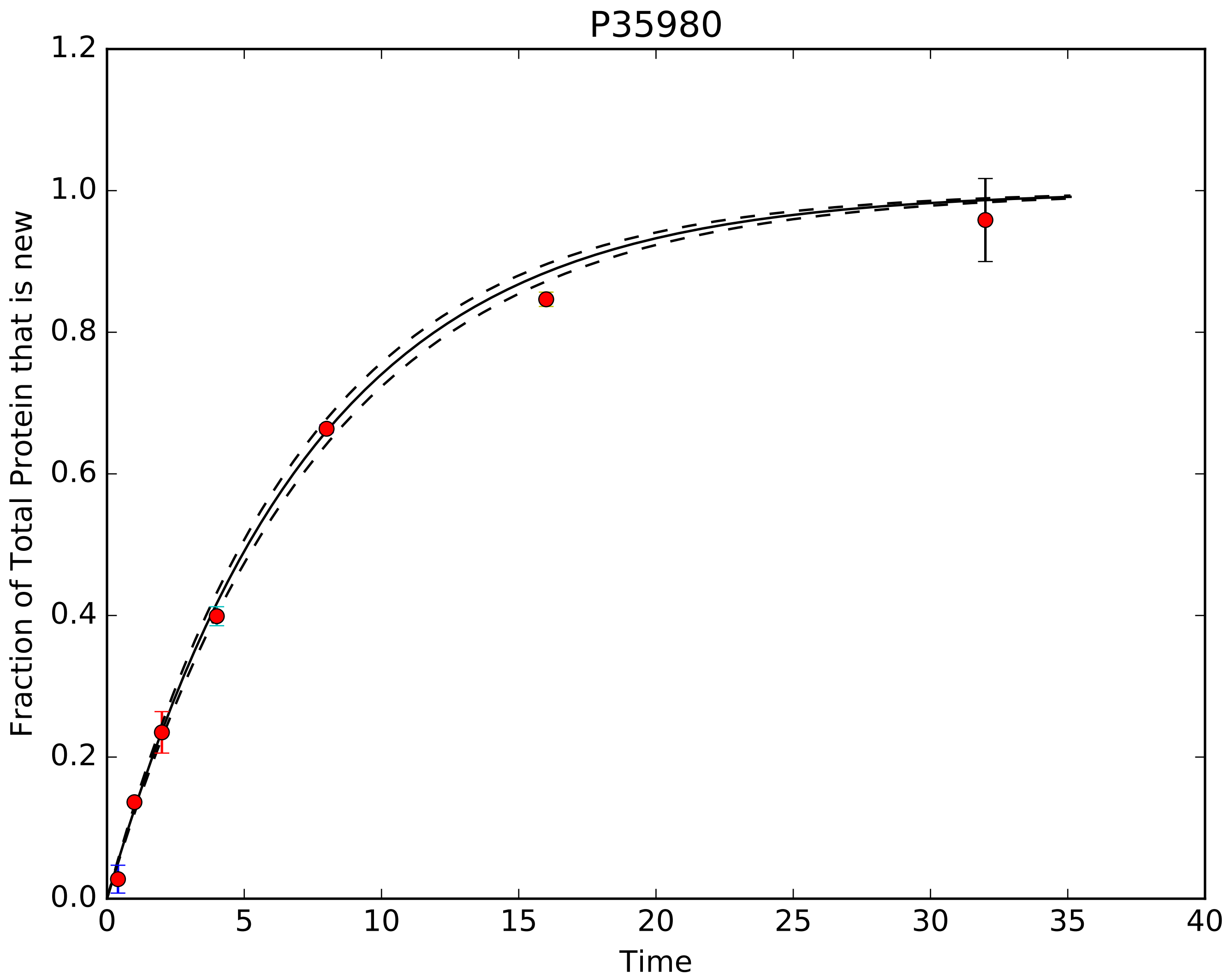

P35980
Fraction of Total Protein that is new
Time
0.0
0.2
0.4
0.6
0.8
1.0
1.2
0
5
10
15
20
25
30
35
40

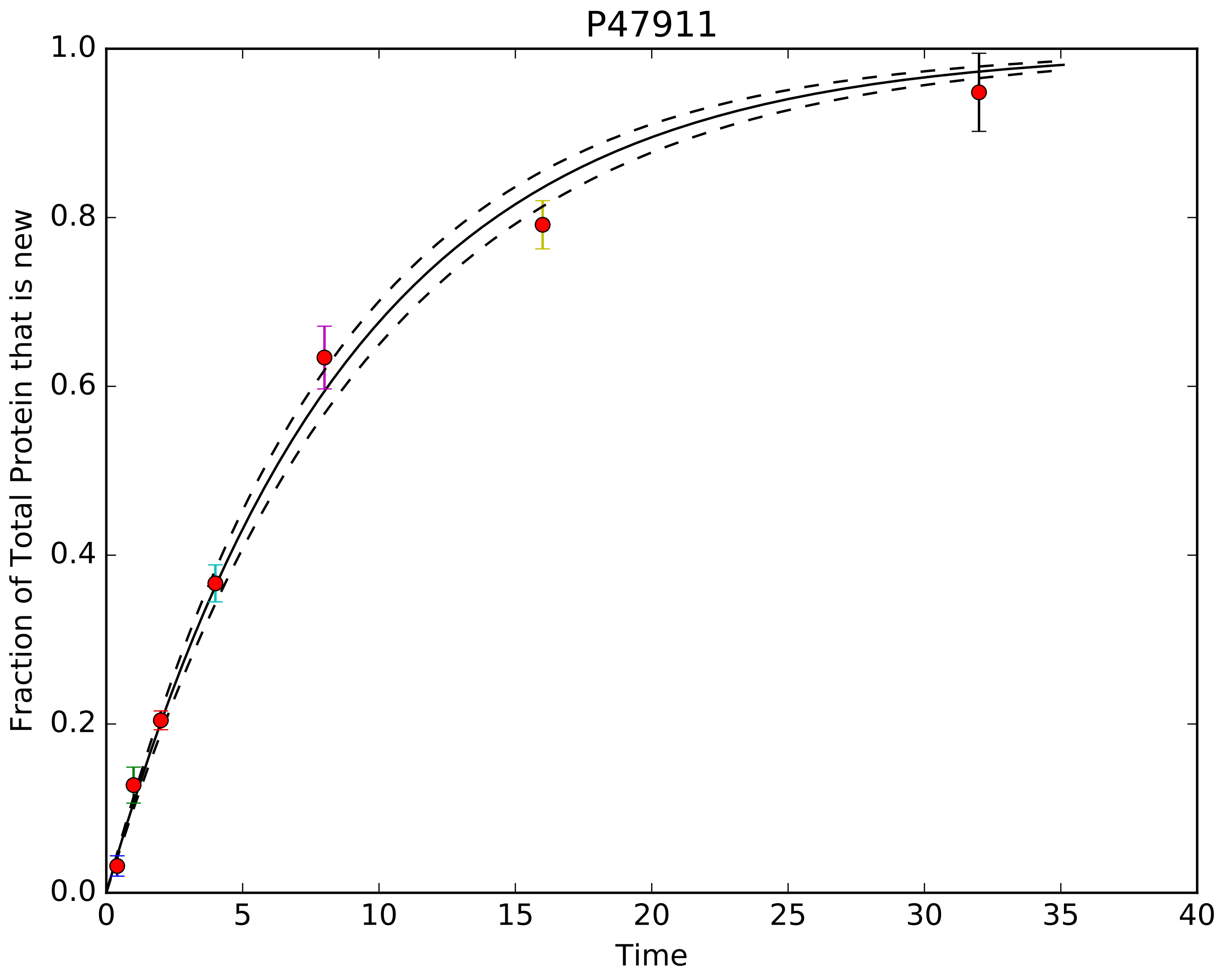

P47911
Fraction of Total Protein that is new
Time
0.0
0.2
0.4
0.6
0.8
1.0
0
5
10
15
20
25
30
35
40

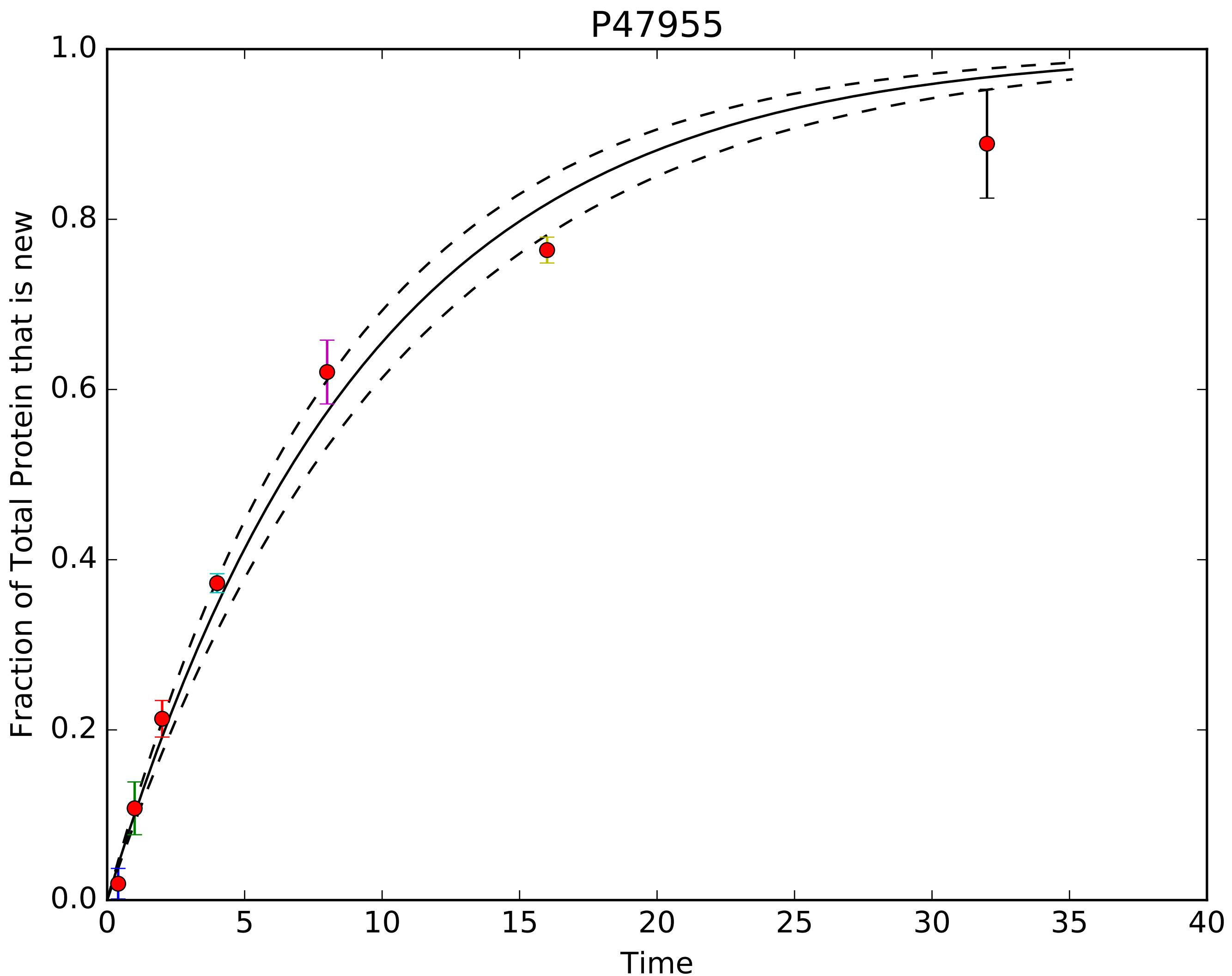

P47955
Fraction of Total Protein that is new
Time
0.0
0.2
0.4
0.6
0.8
1.0
0
5
10
15
20
25
30
35
40

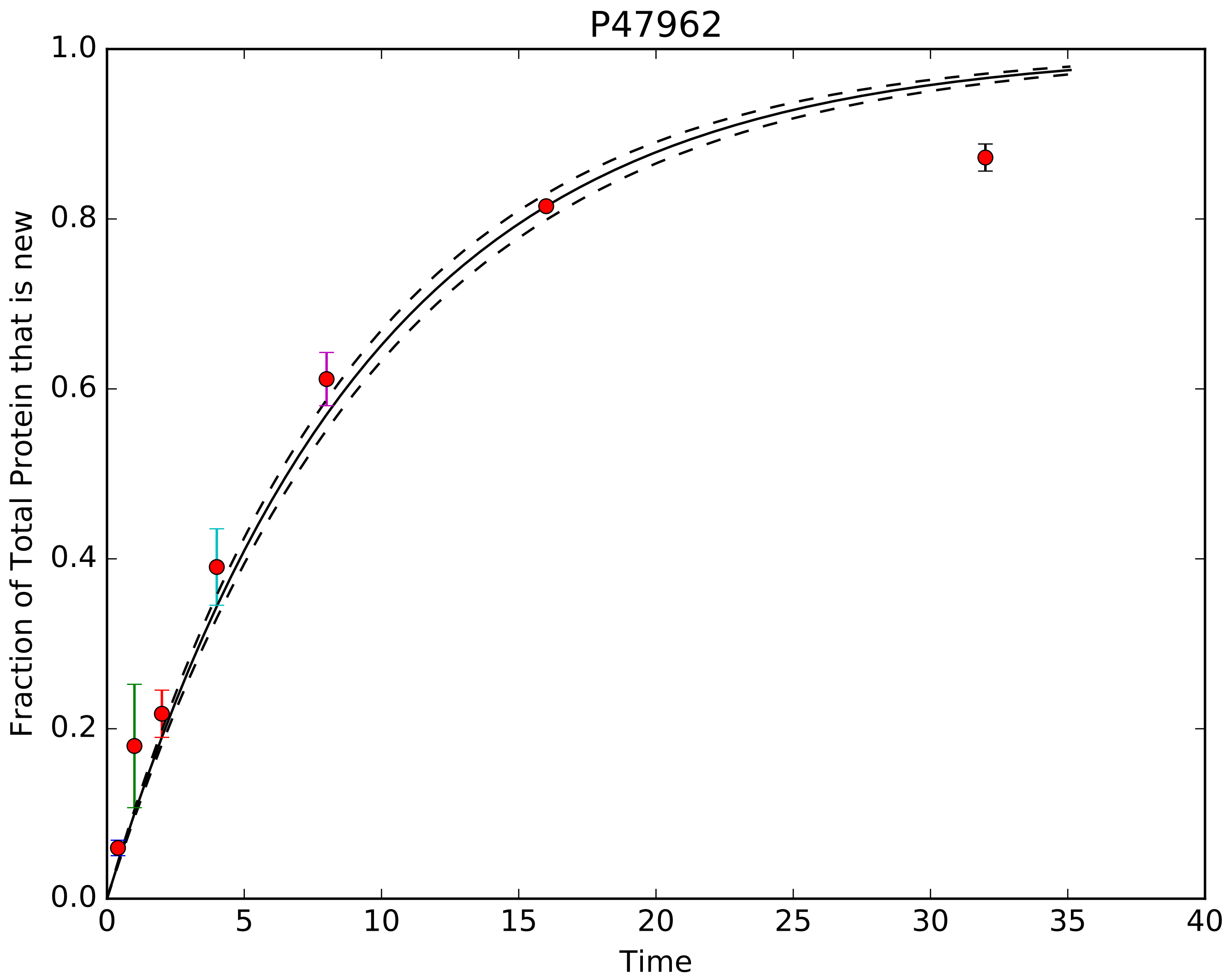

P47962
Fraction of Total Protein that is new
Time
0.0
0.2
0.4
0.6
0.8
1.0
0
5
10
15
20
25
30
35
40

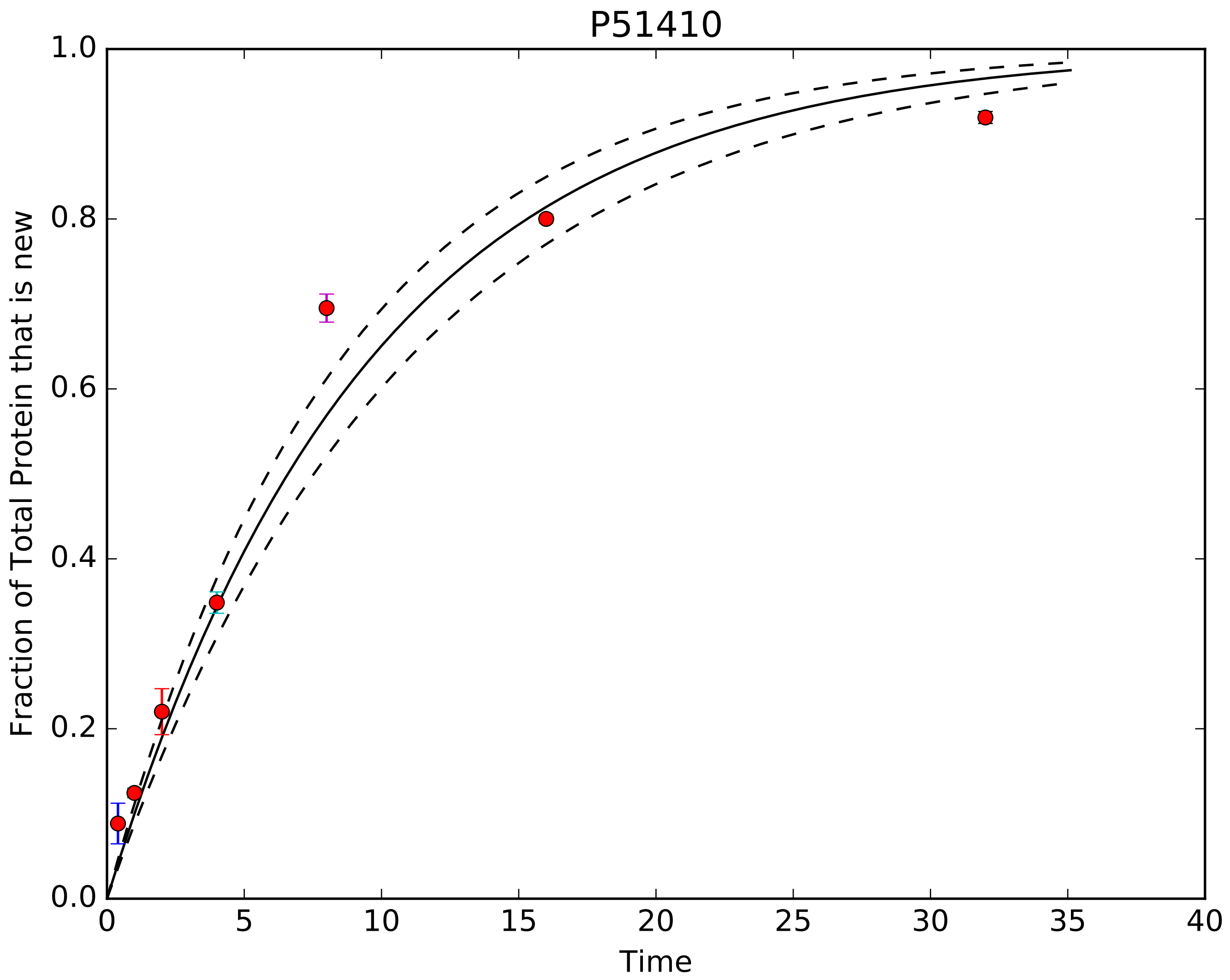

P51410
Fraction of Total Protein that is new
Time
1.0
0.8
0.6
0.4
0.2
0.0
0
5
10
15
20
25
30
35
40

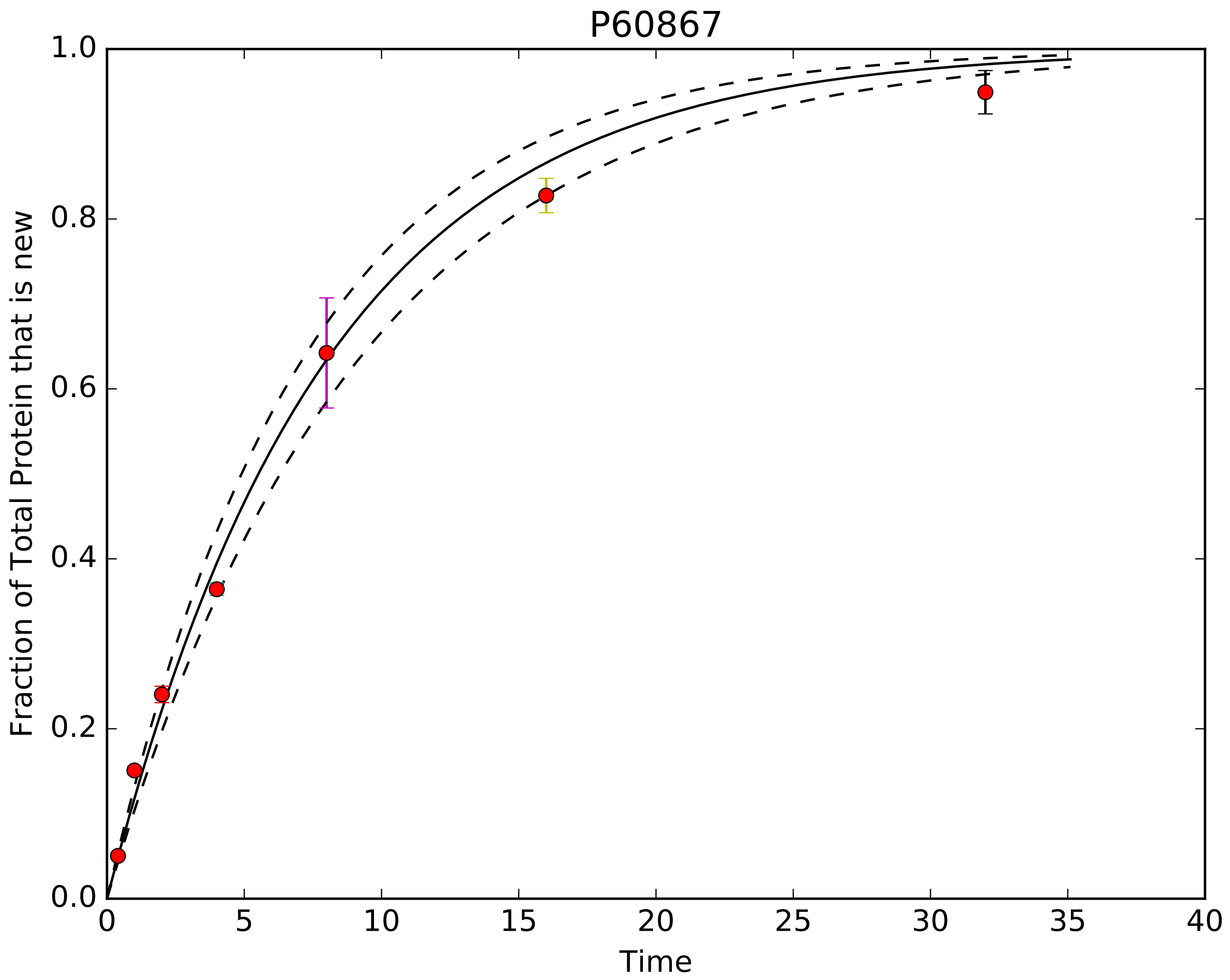
P60867
Fraction of Total Protein that is new
Time
0.0
0.2
0.4
0.6
0.8
1.0
0
5
10
15
20
25
30
35
40

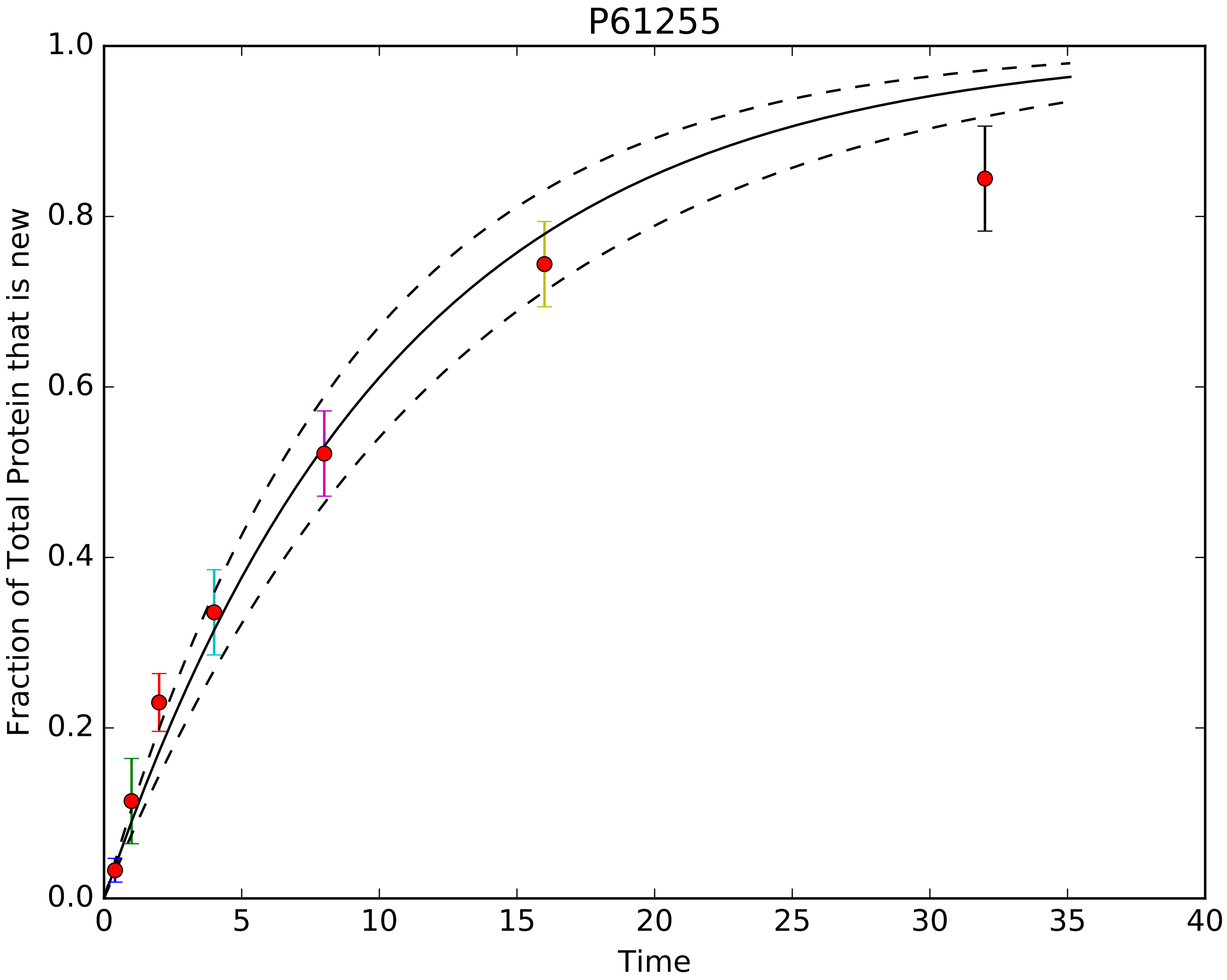

P61255
Fraction of Total Protein that is new
Time
0.0
0.2
0.4
0.6
0.8
1.0
0
5
10
15
20
25
30
35
40

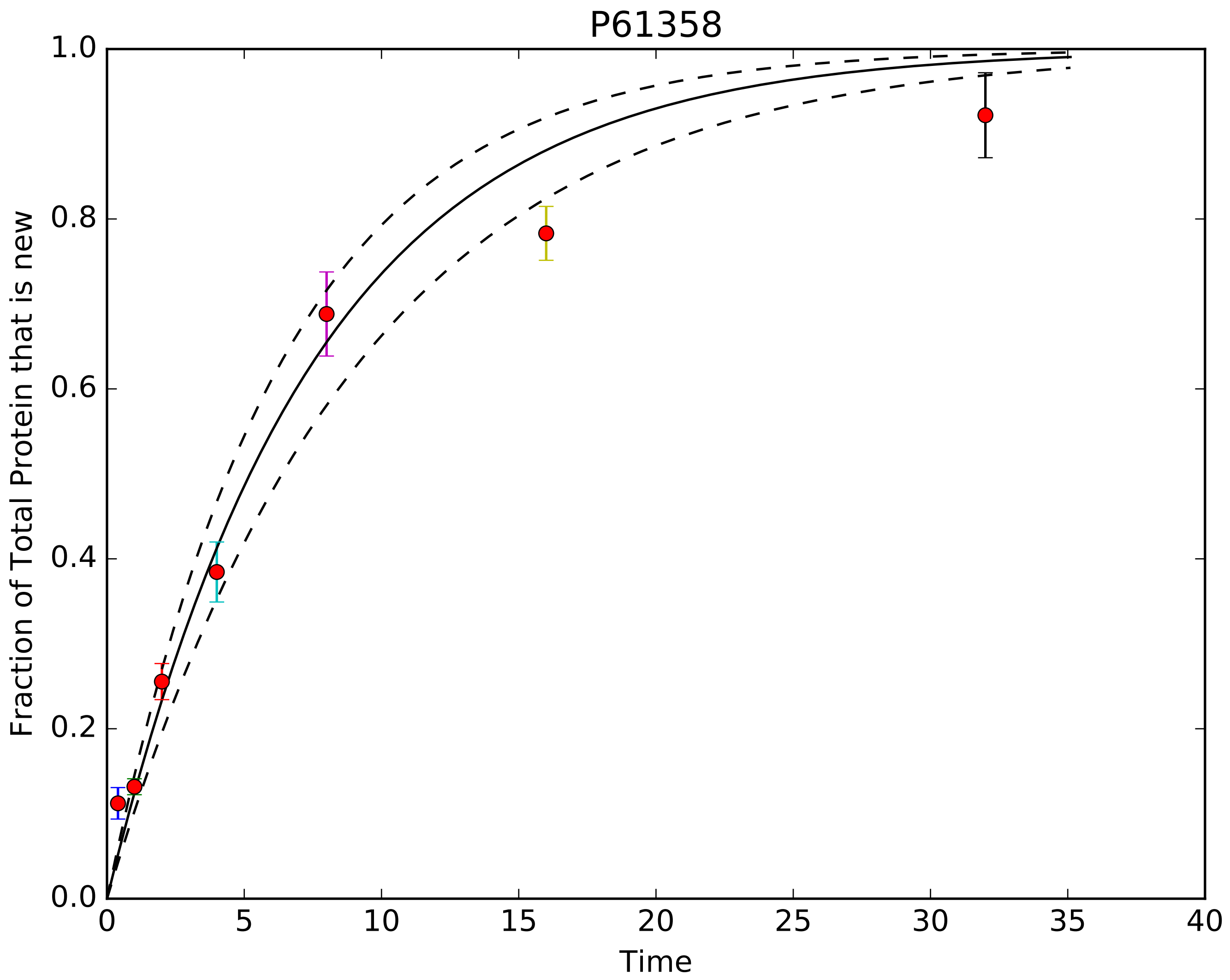

P61358
Fraction of Total Protein that is new
Time
0.0
0.2
0.4
0.6
0.8
1.0
0
5
10
15
20
25
30
35
40

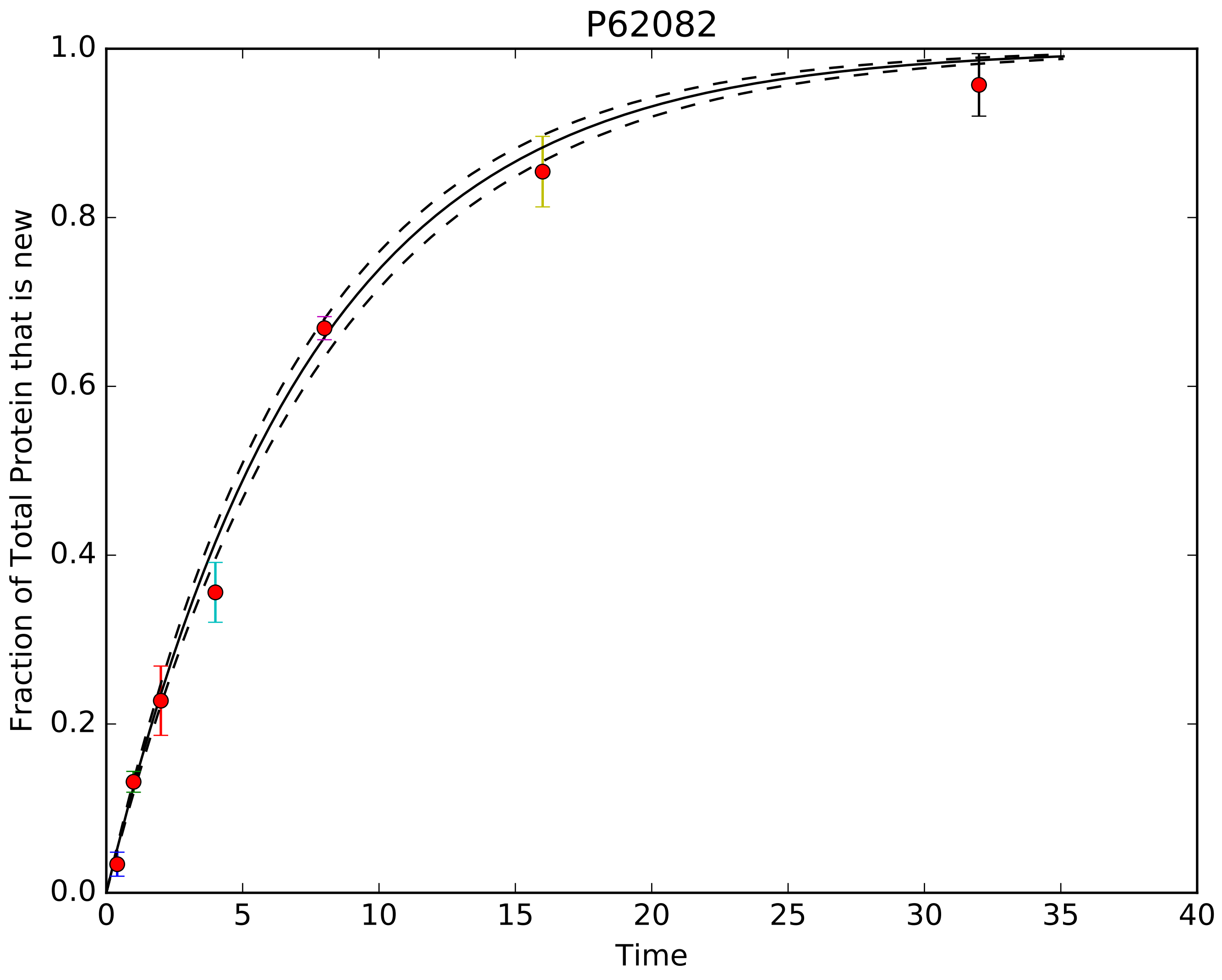
P62082
Fraction of Total Protein that is new
Time
0.0
0.2
0.4
0.6
0.8
1.0
0
5
10
15
20
25
30
35
40

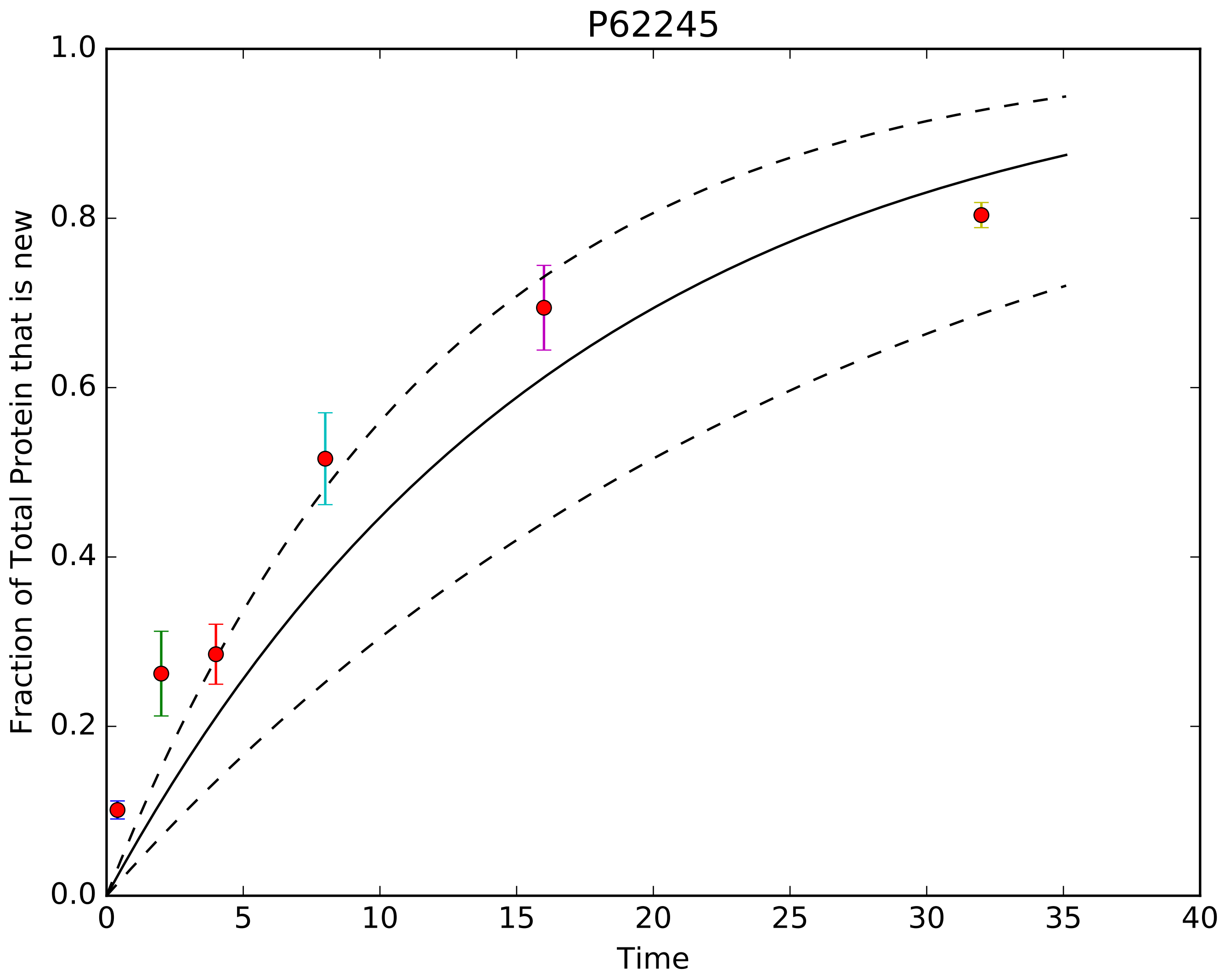
P62245
Fraction of Total Protein that is new
Time
0.0
0.2
0.4
0.6
0.8
1.0
0
5
10
15
20
25
30
35
40

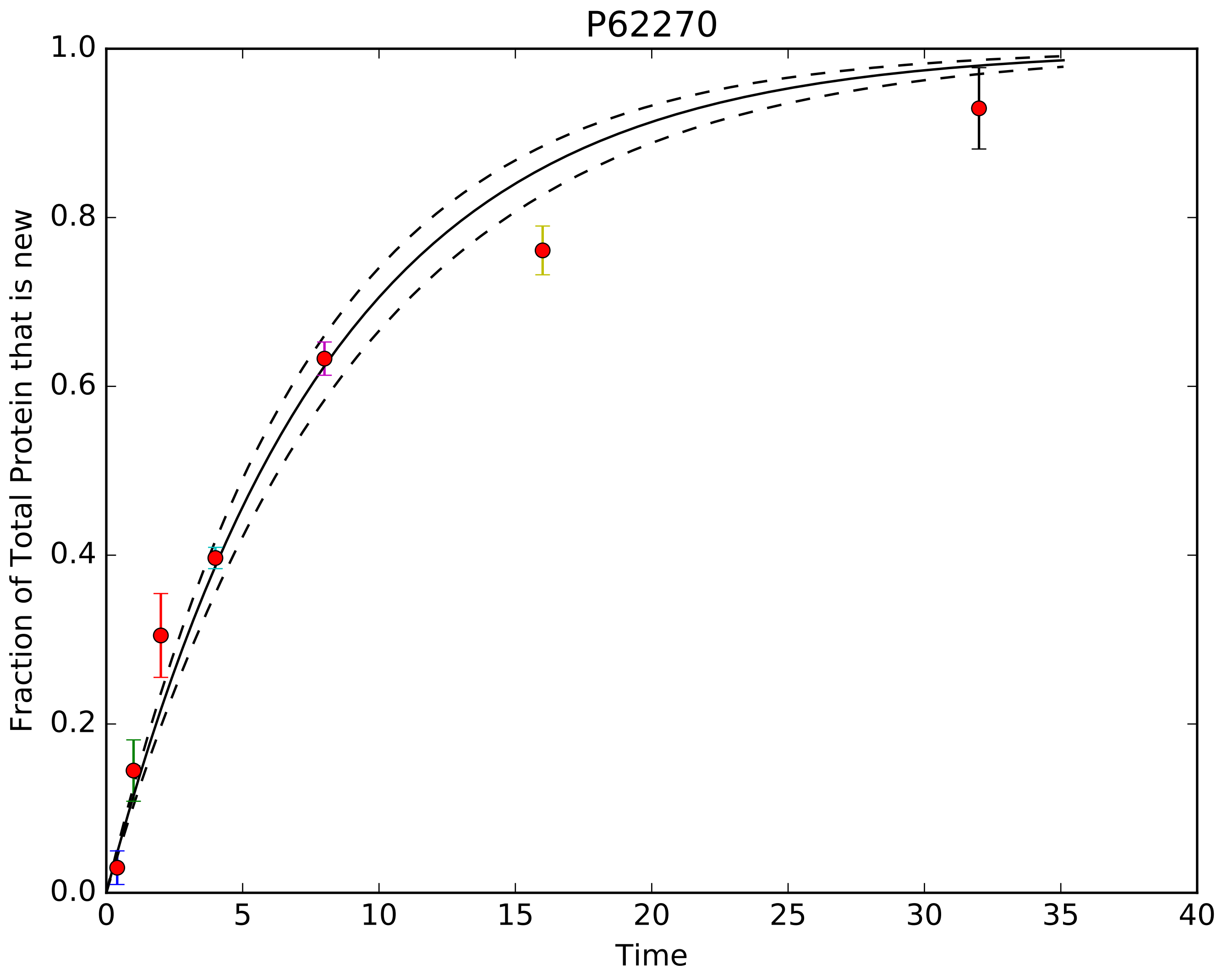
P62270
Fraction of Total Protein that is new
Time
0.0
0.2
0.4
0.6
0.8
1.0
0
5
10
15
20
25
30
35
40

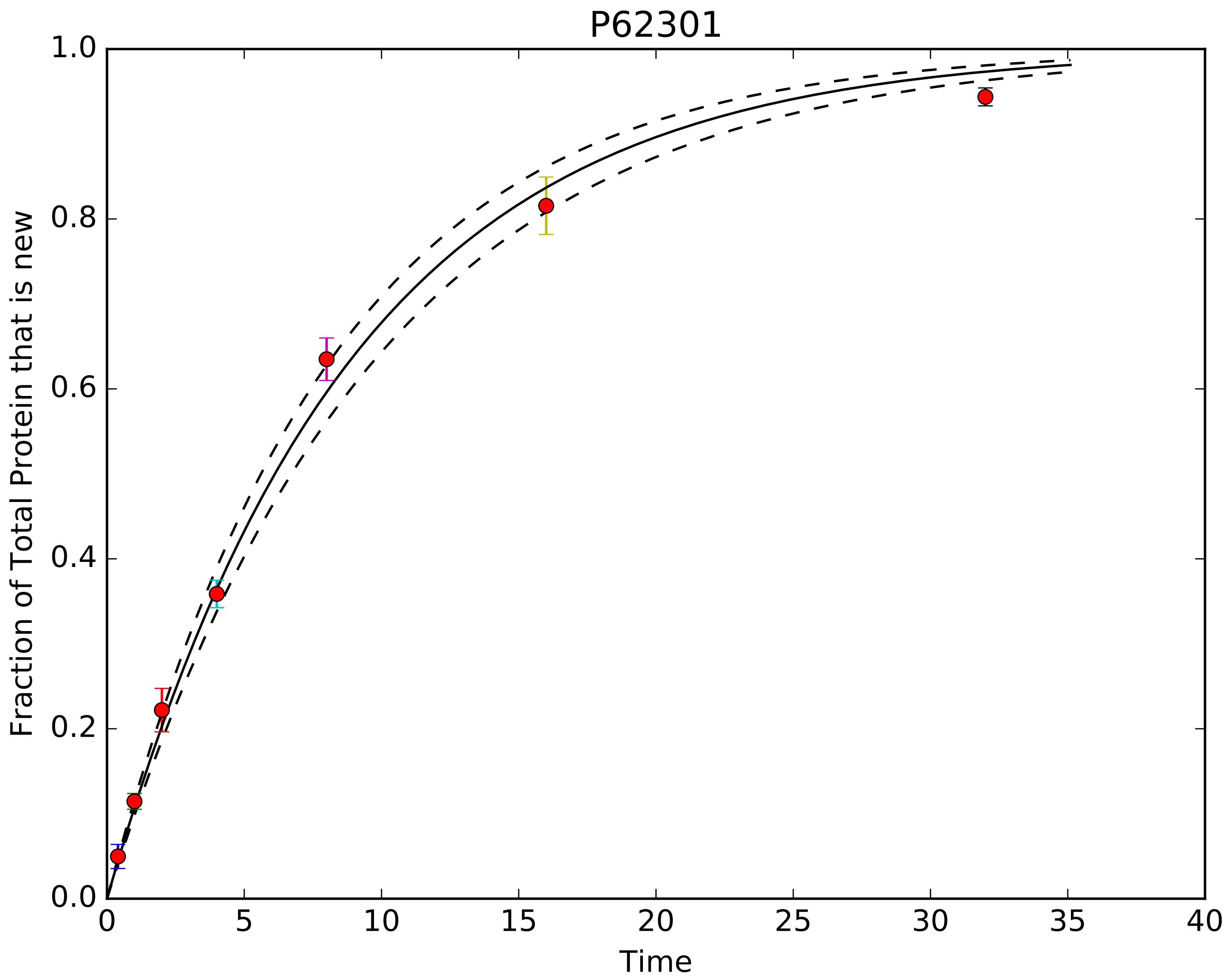

P62301
Fraction of Total Protein that is new
Time
0.0
0.2
0.4
0.6
0.8
1.0
0
5
10
15
20
25
30
35
40

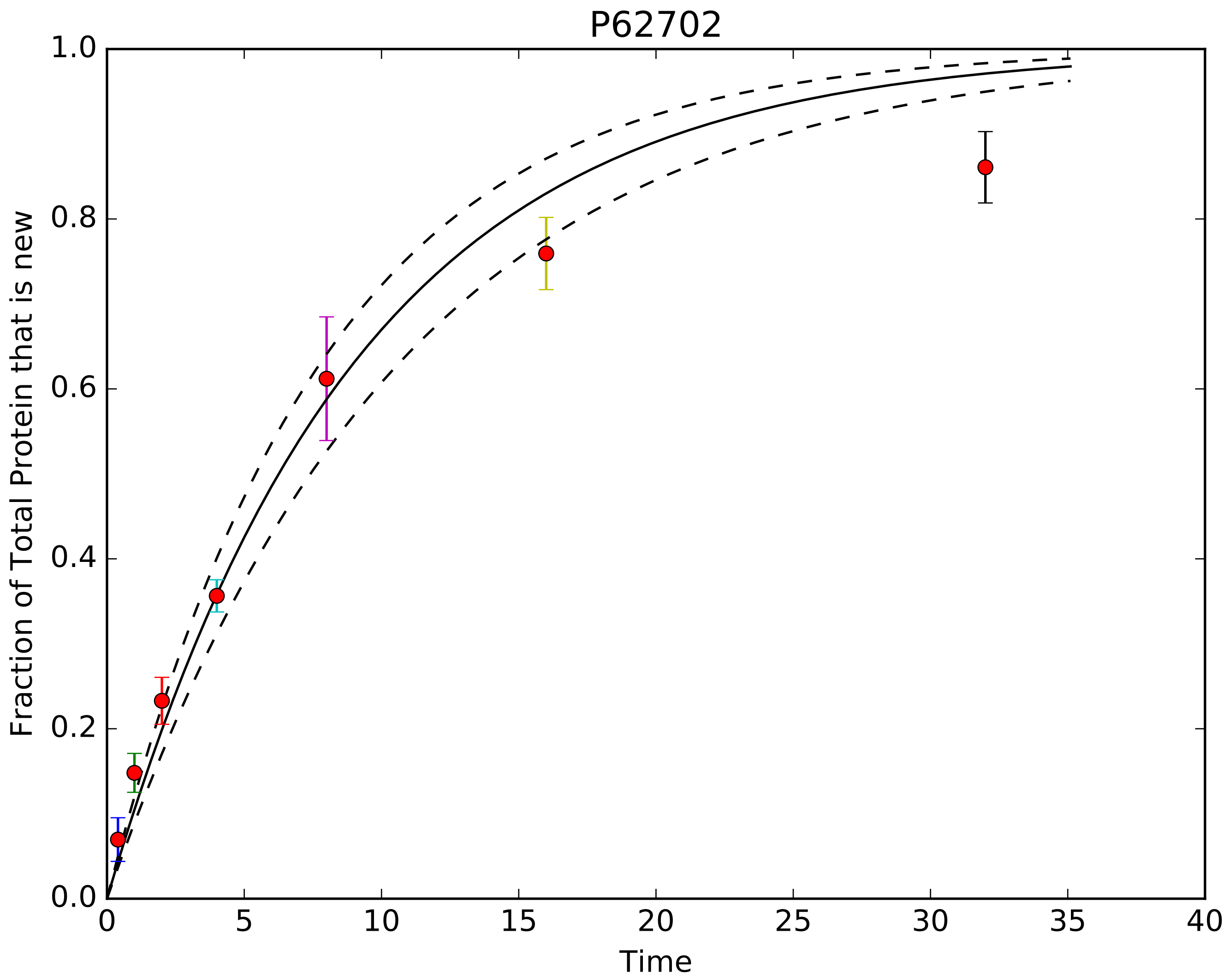
P62702
Fraction of Total Protein that is new
Time
0.0
0.2
0.4
0.6
0.8
1.0
0
5
10
15
20
25
30
35
40

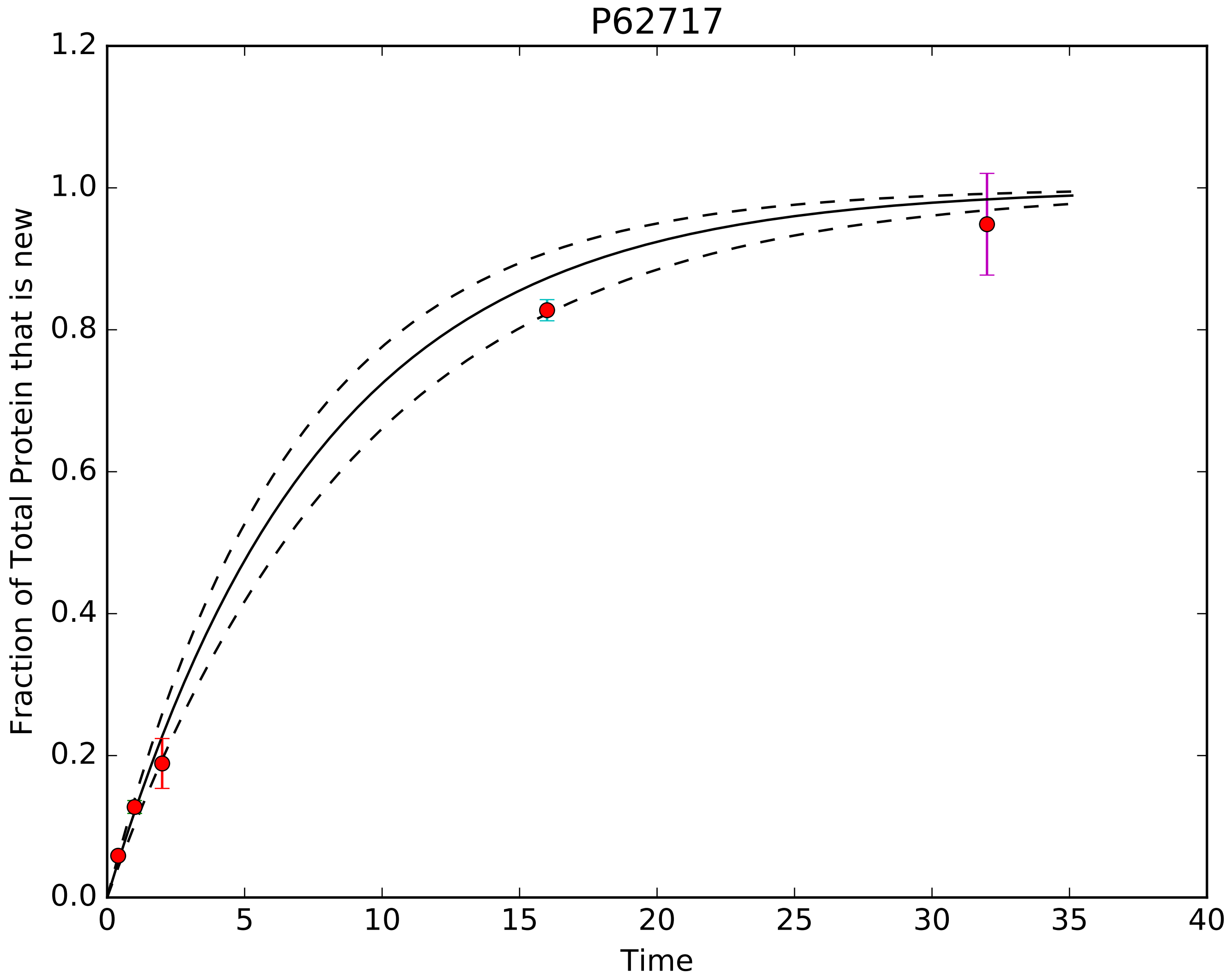

P62717
Fraction of Total Protein that is new
Time
0.0
0.2
0.4
0.6
0.8
1.0
1.2
0
5
10
15
20
25
30
35
40

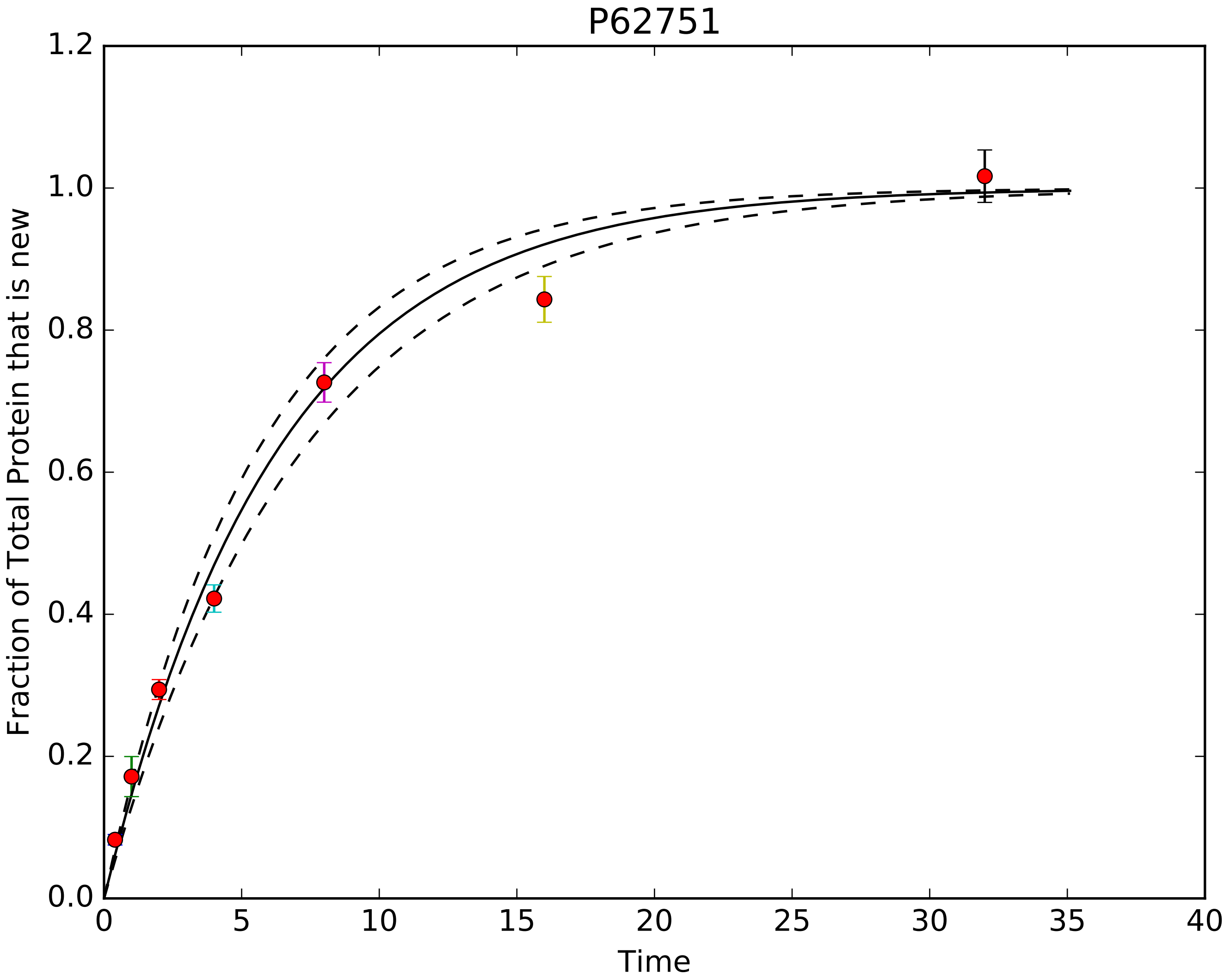
P62751
Fraction of Total Protein that is new
Time
0.0
0.2
0.4
0.6
0.8
1.0
1.2
0
5
10
15
20
25
30
35
40

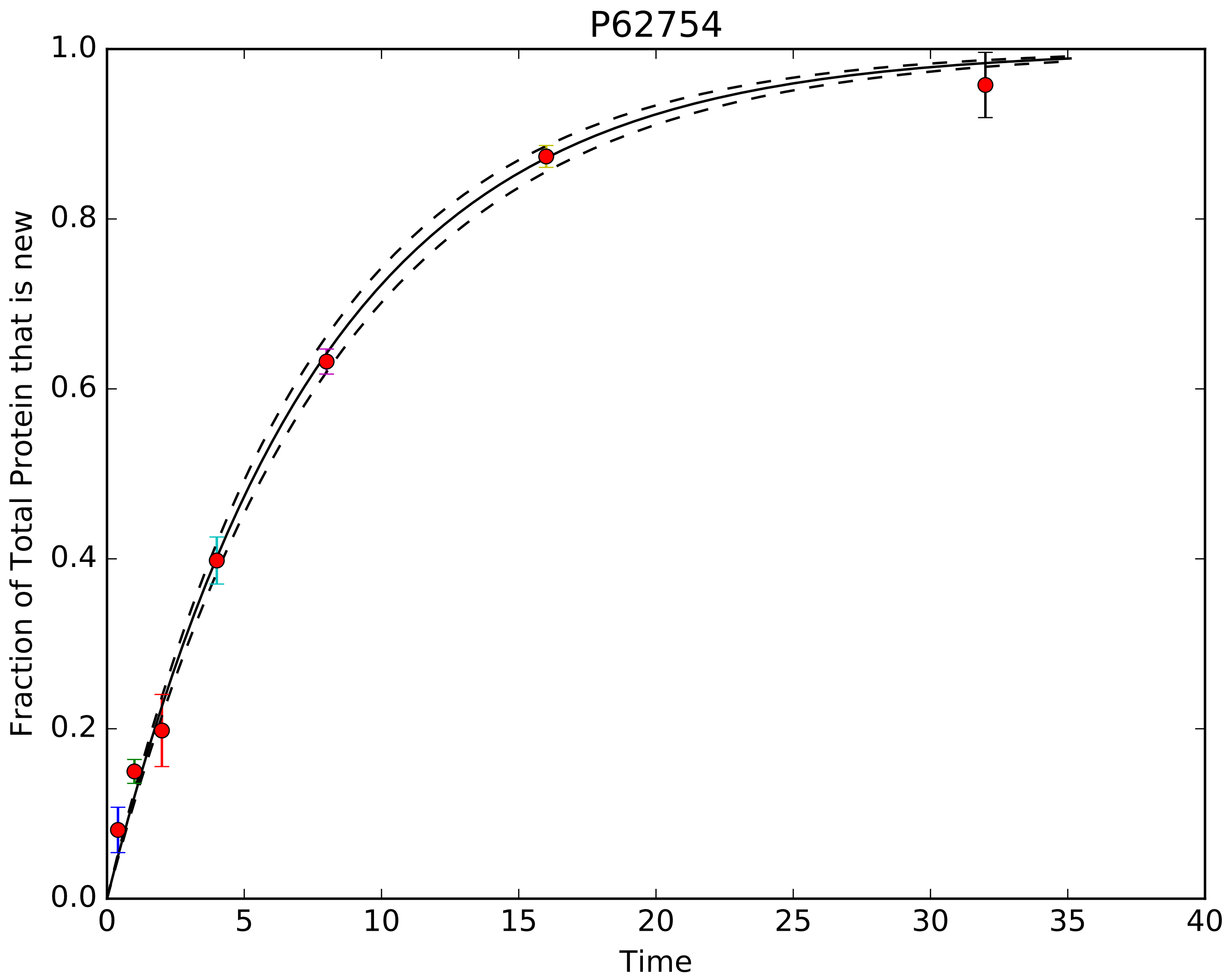
P62754
Fraction of Total Protein that is new
Time
1.0
0.8
0.6
0.4
0.2
0.0
0
5
10
15
20
25
30
35
40

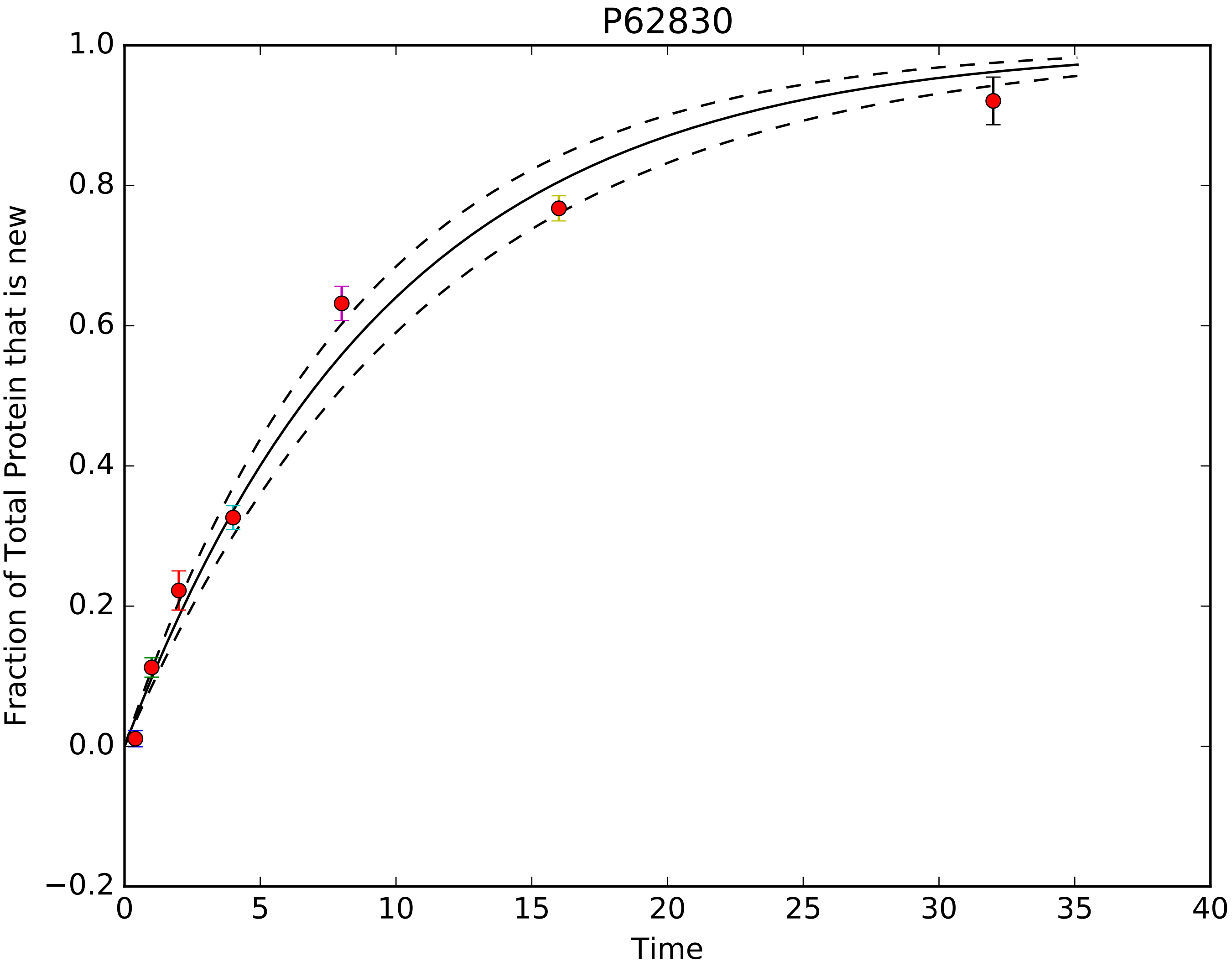
P62830
Fraction of Total Protein that is new
Time
1.0
0.8
0.6
0.4
0.2
0.0
−0.2
0
5
10
15
20
25
30
35
40

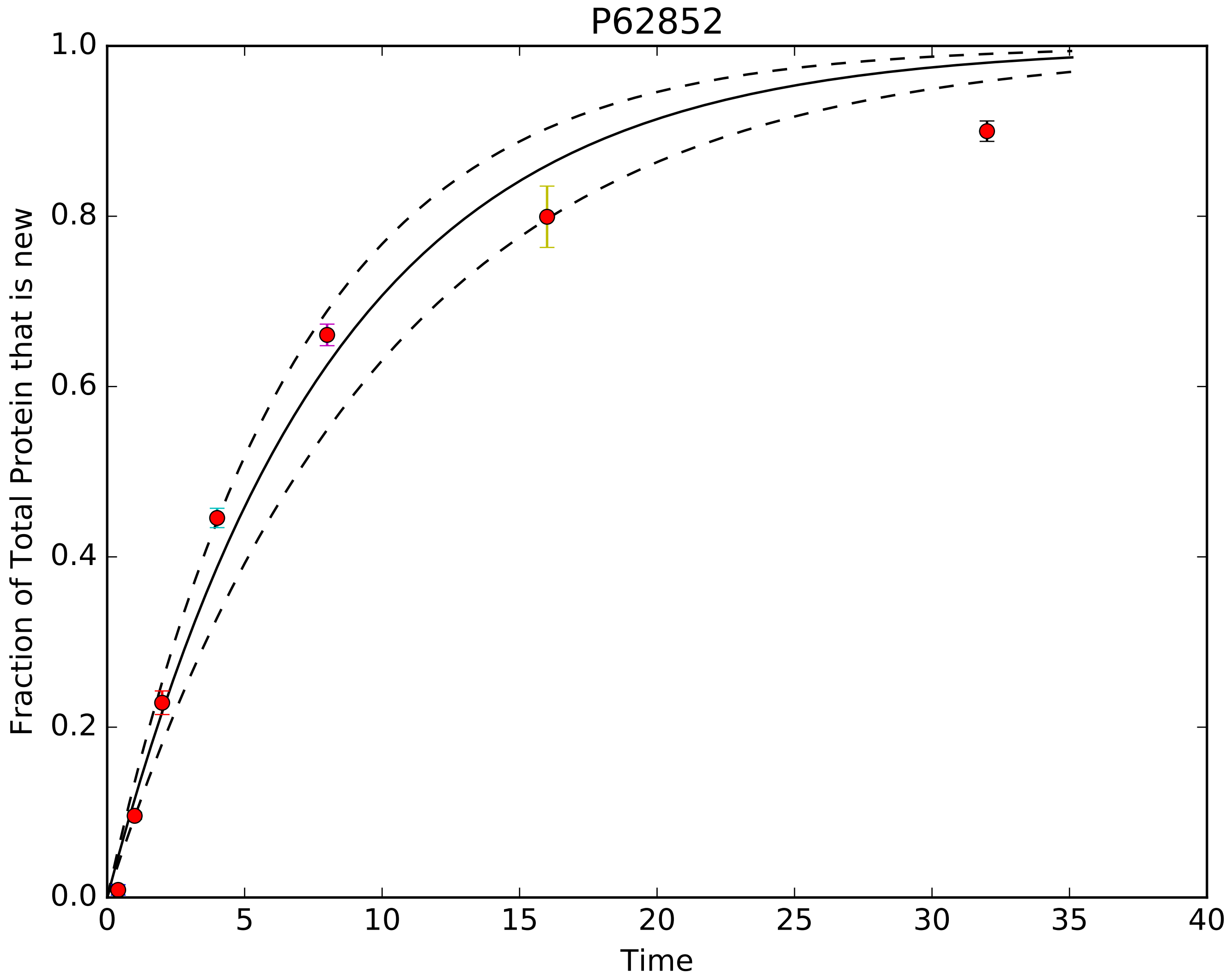
P62852
Fraction of Total Protein that is new
Time
0.0
0.2
0.4
0.6
0.8
1.0
0
5
10
15
20
25
30
35
40

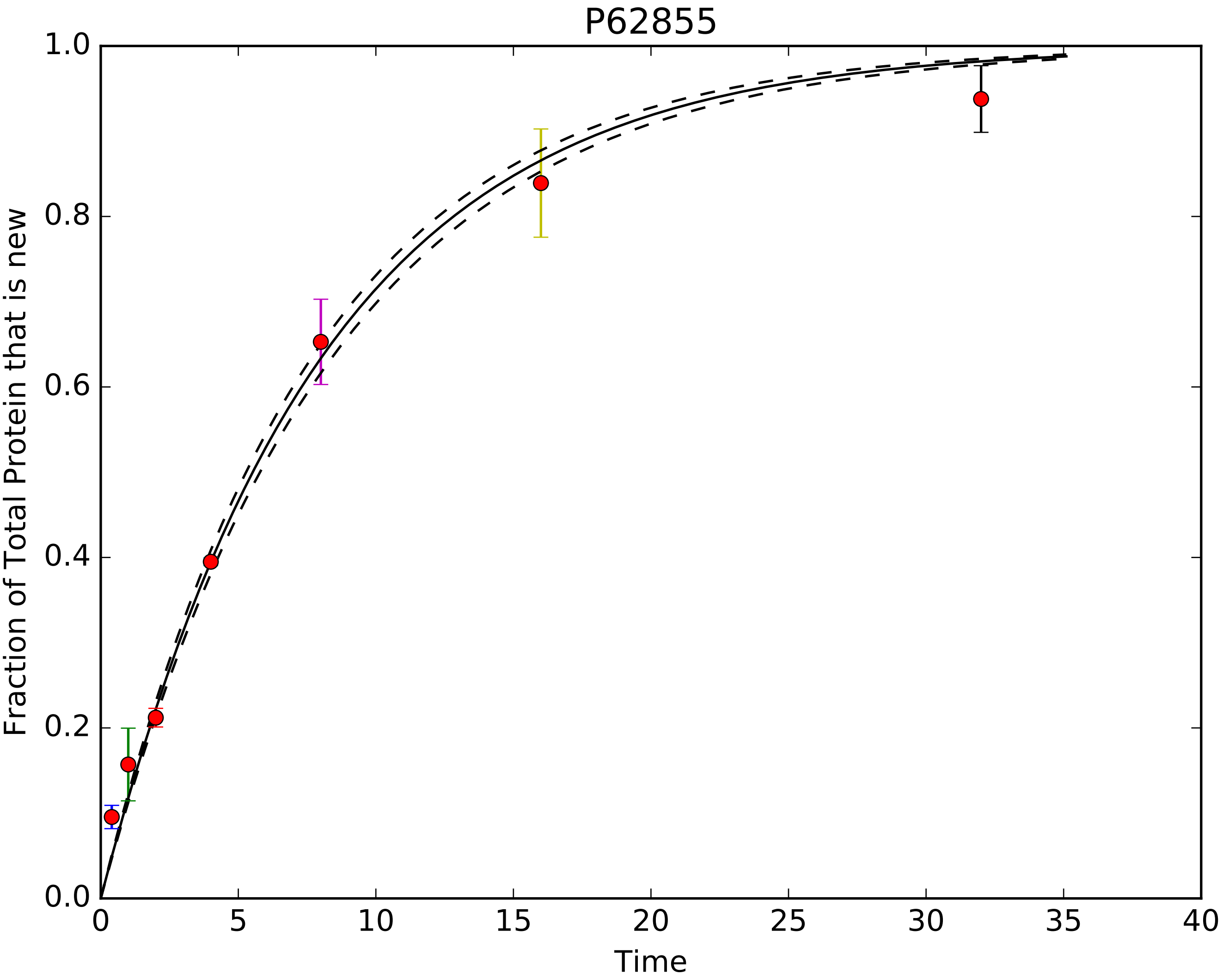
P62855
Fraction of Total Protein that is new
Time
0.0
0.2
0.4
0.6
0.8
1.0
0
5
10
15
20
25
30
35
40

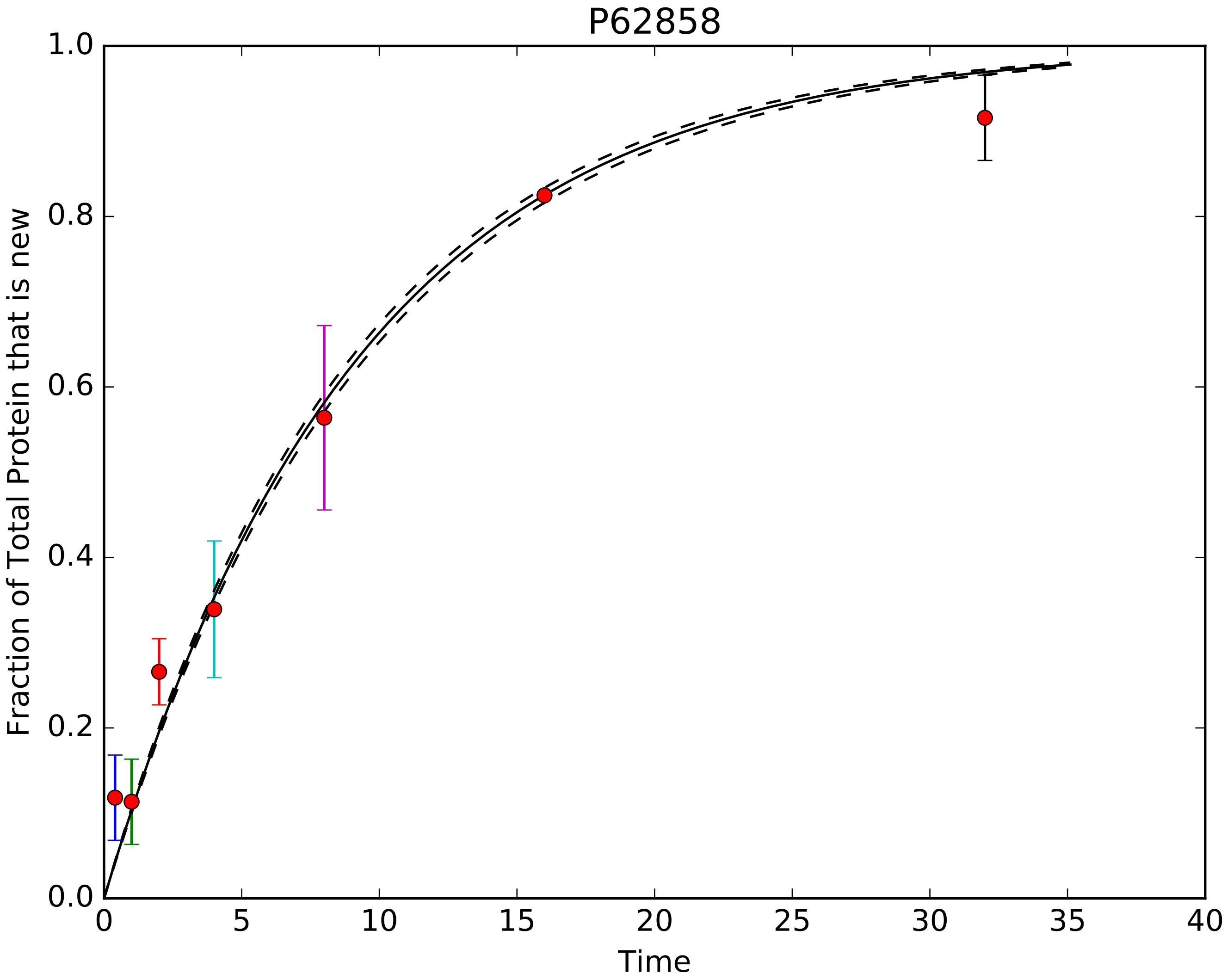
P62858
Fraction of Total Protein that is new
Time
0.0
0.2
0.4
0.6
0.8
1.0
0
5
10
15
20
25
30
35
40

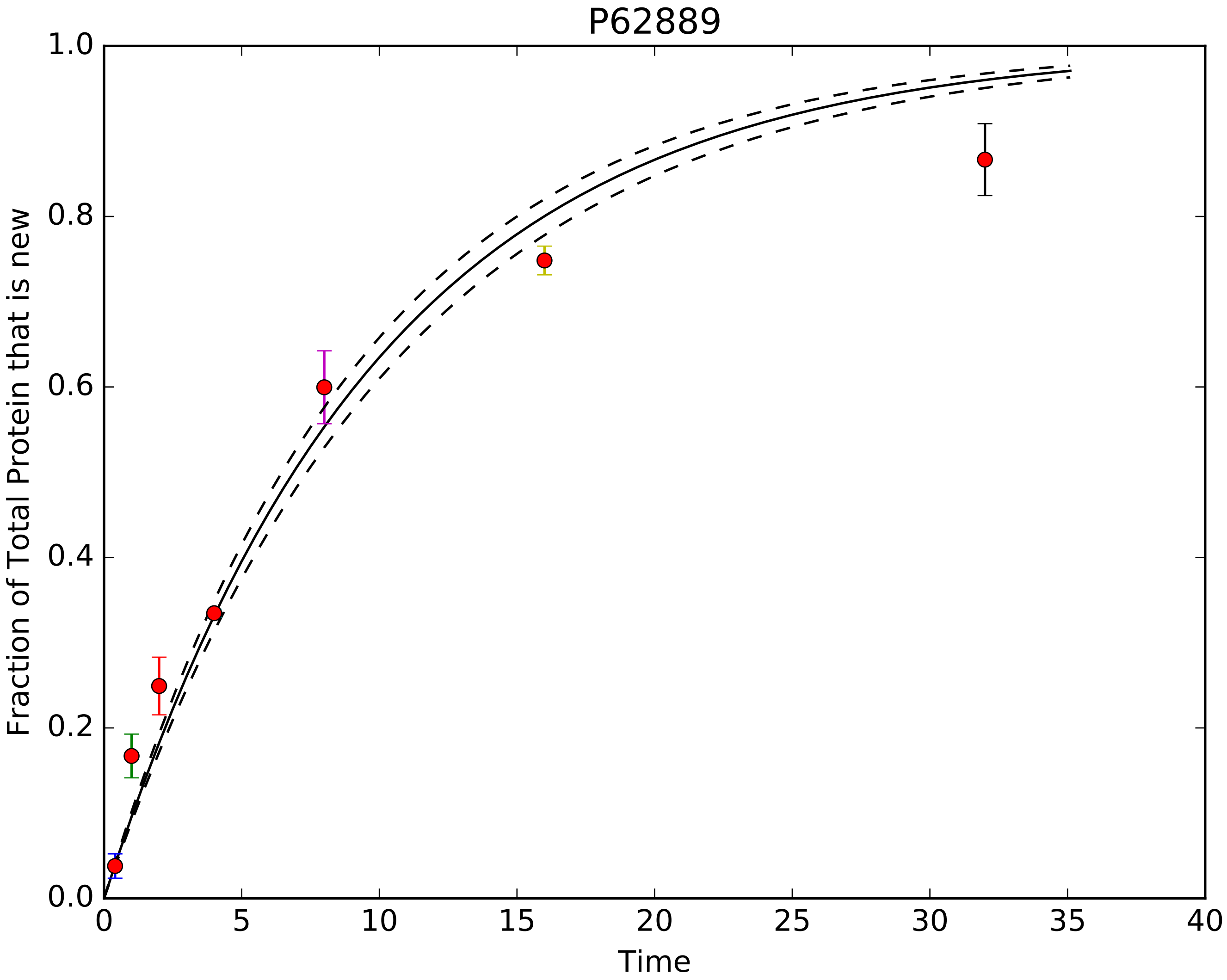
P62889
Fraction of Total Protein that is new
Time
0.0
0.2
0.4
0.6
0.8
1.0
0
5
10
15
20
25
30
35
40

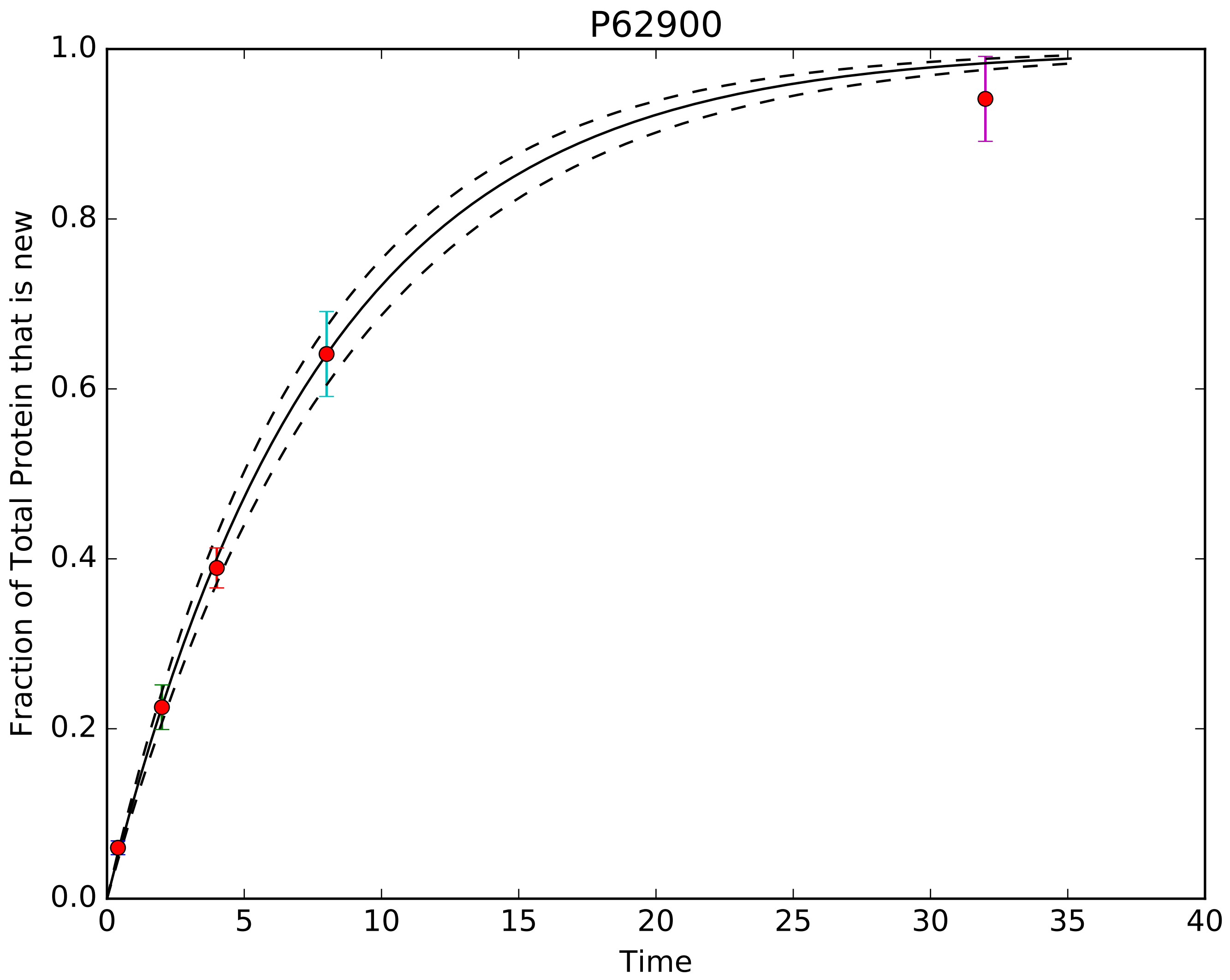
P62900
Fraction of Total Protein that is new
Time
1.0
0.8
0.6
0.4
0.2
0.0
0
5
10
15
20
25
30
35
40

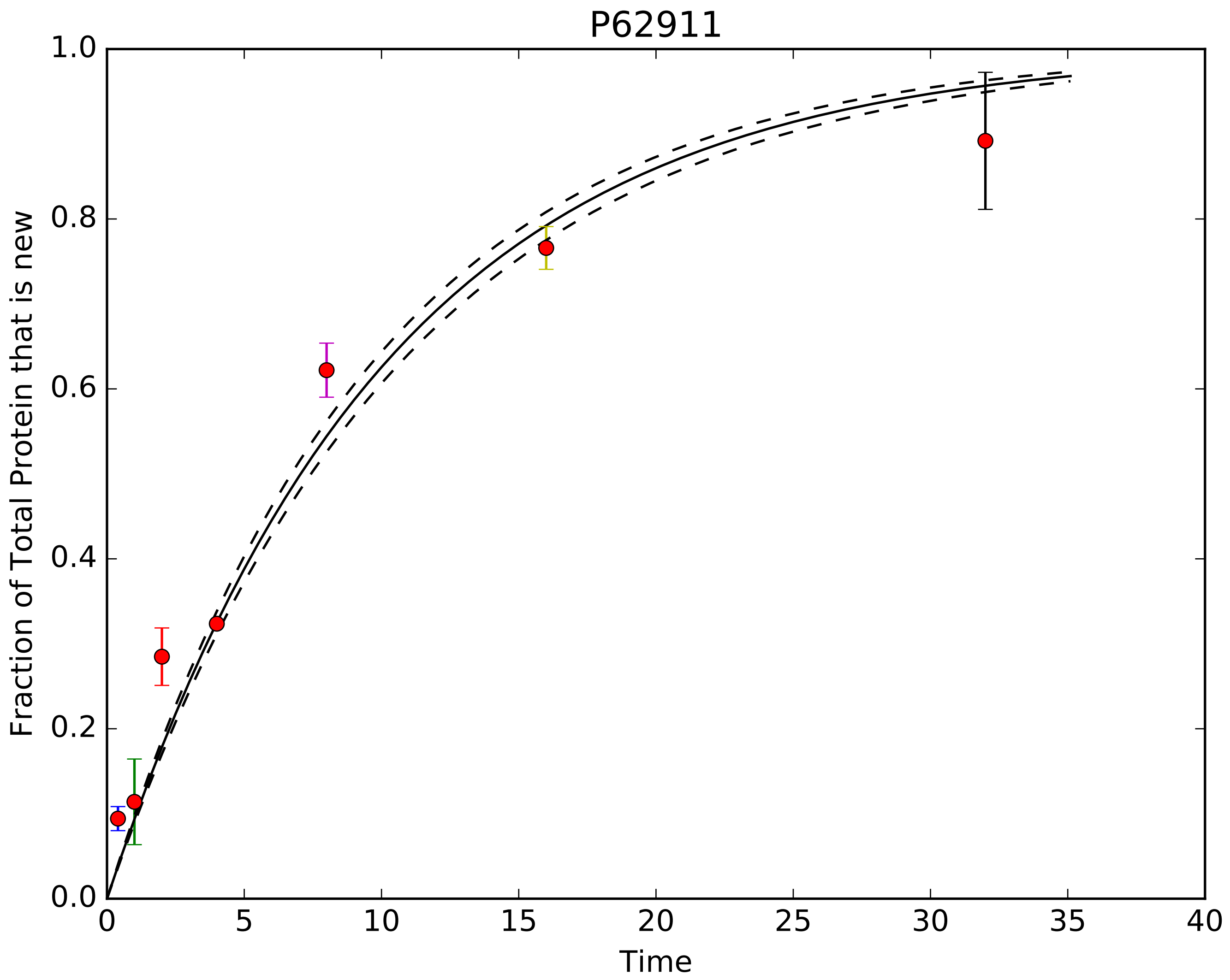
P62911
Fraction of Total Protein that is new
Time
0.0
0.2
0.4
0.6
0.8
1.0
0
5
10
15
20
25
30
35
40

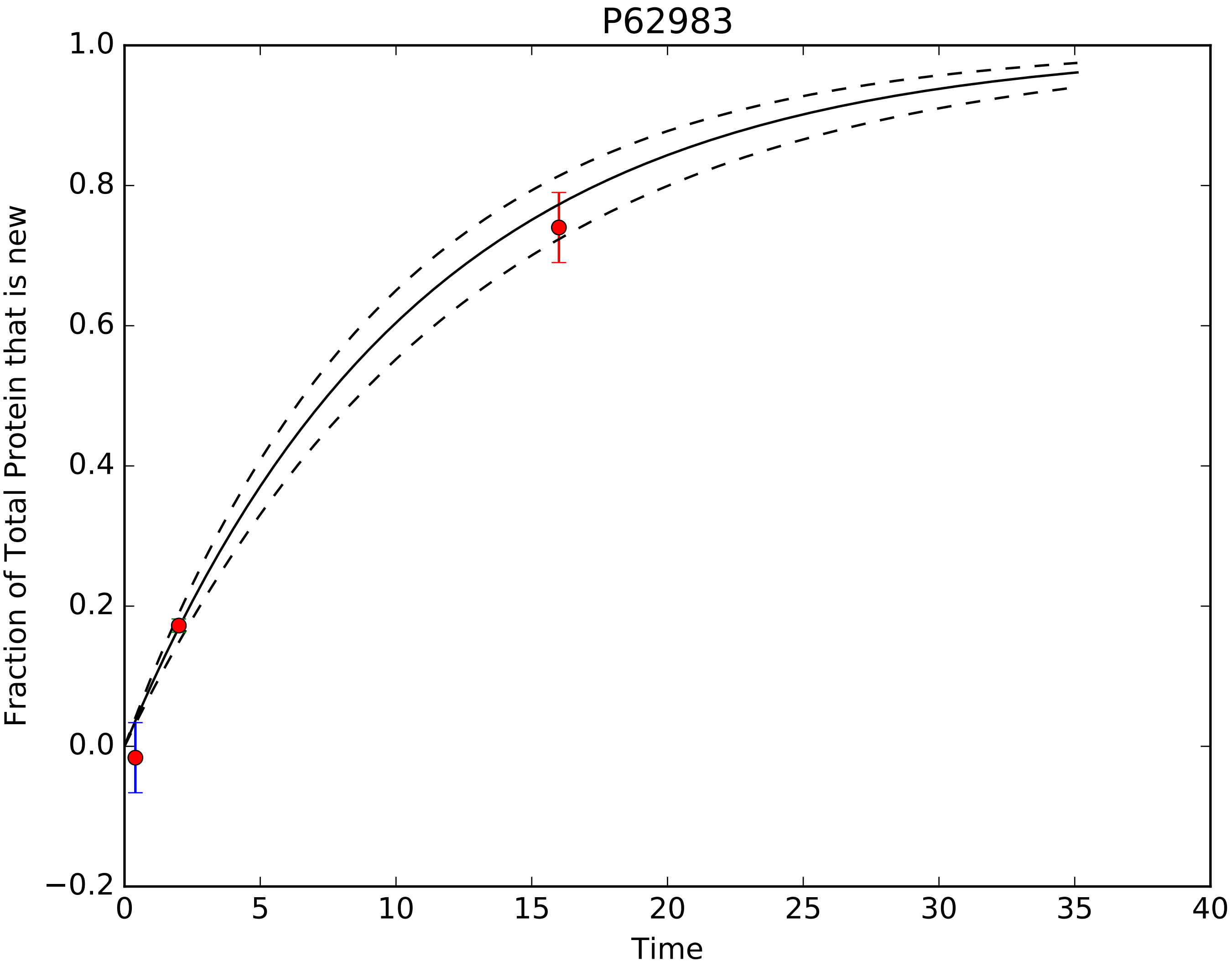

P62983
Fraction of Total Protein that is new
Time
1.0
0.8
0.6
0.4
0.2
0.0
−0.2
0
5
10
15
20
25
30
35
40

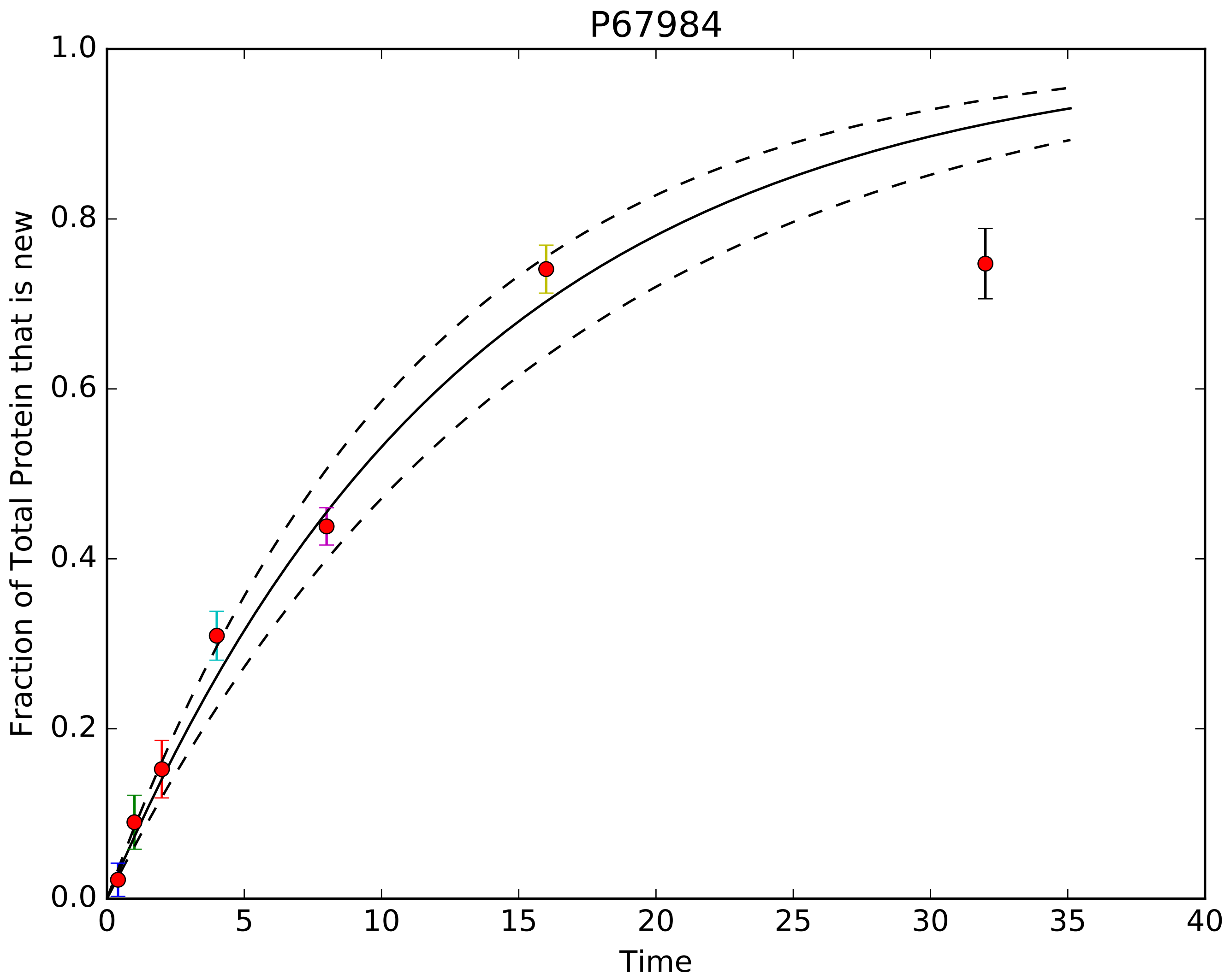

P67984
Fraction of Total Protein that is new
Time
1.0
0.8
0.6
0.4
0.2
0.0
0
5
10
15
20
25
30
35
40

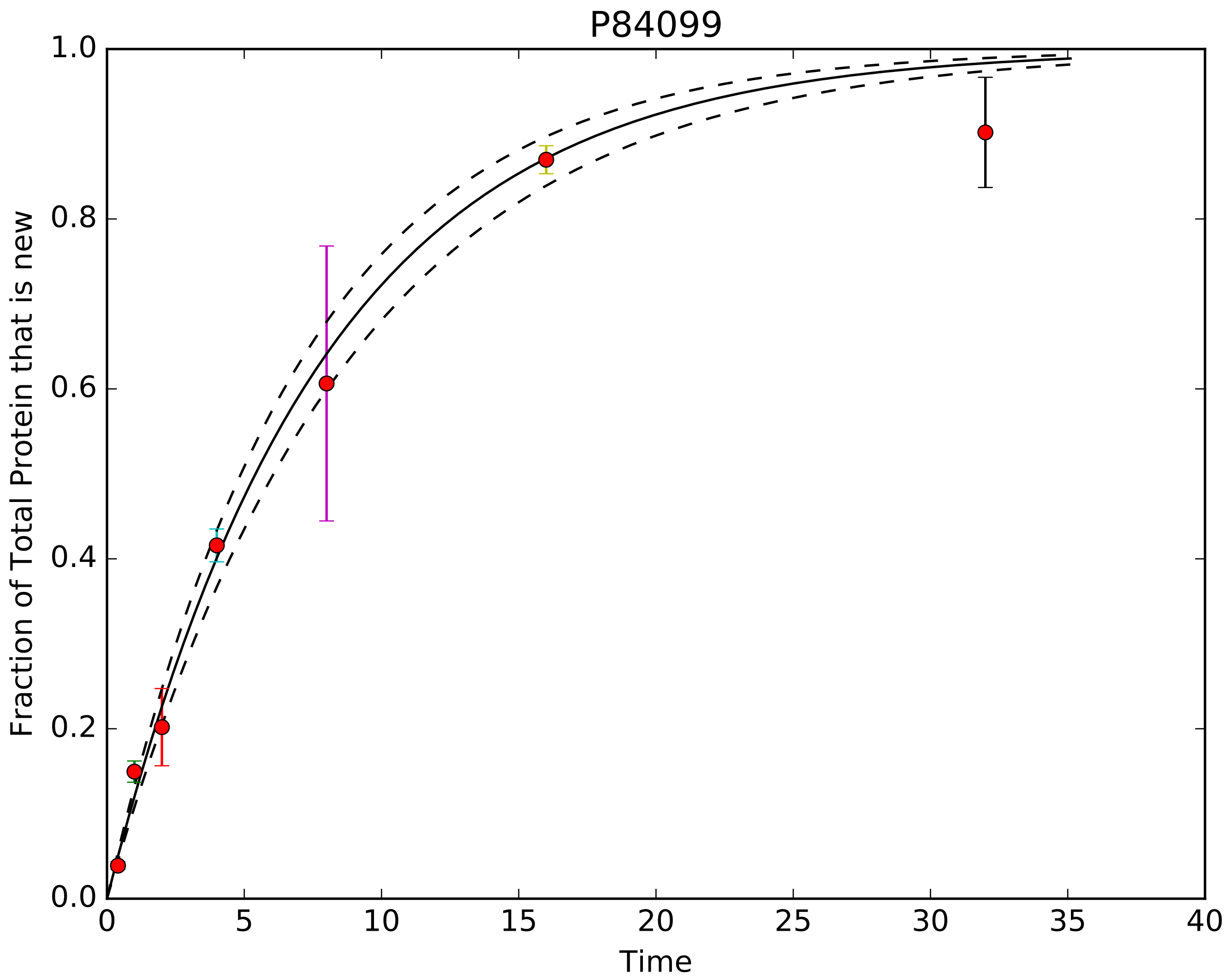
P84099
Fraction of Total Protein that is new
Time
0.0
0.2
0.4
0.6
0.8
1.0
0
5
10
15
20
25
30
35
40

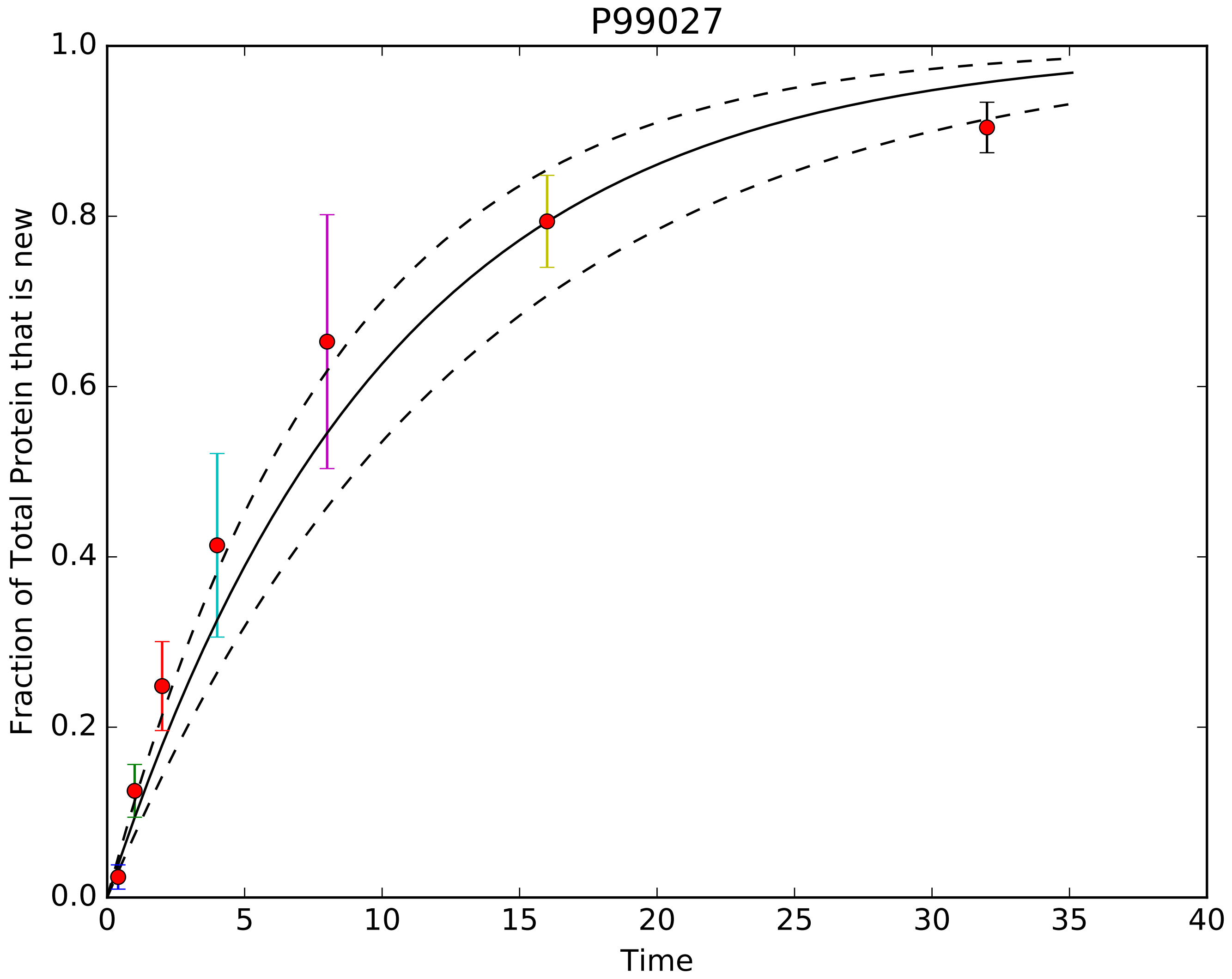
P99027
Fraction of Total Protein that is new
Time
0.0
0.2
0.4
0.6
0.8
1.0
0
5
10
15
20
25
30
35
40

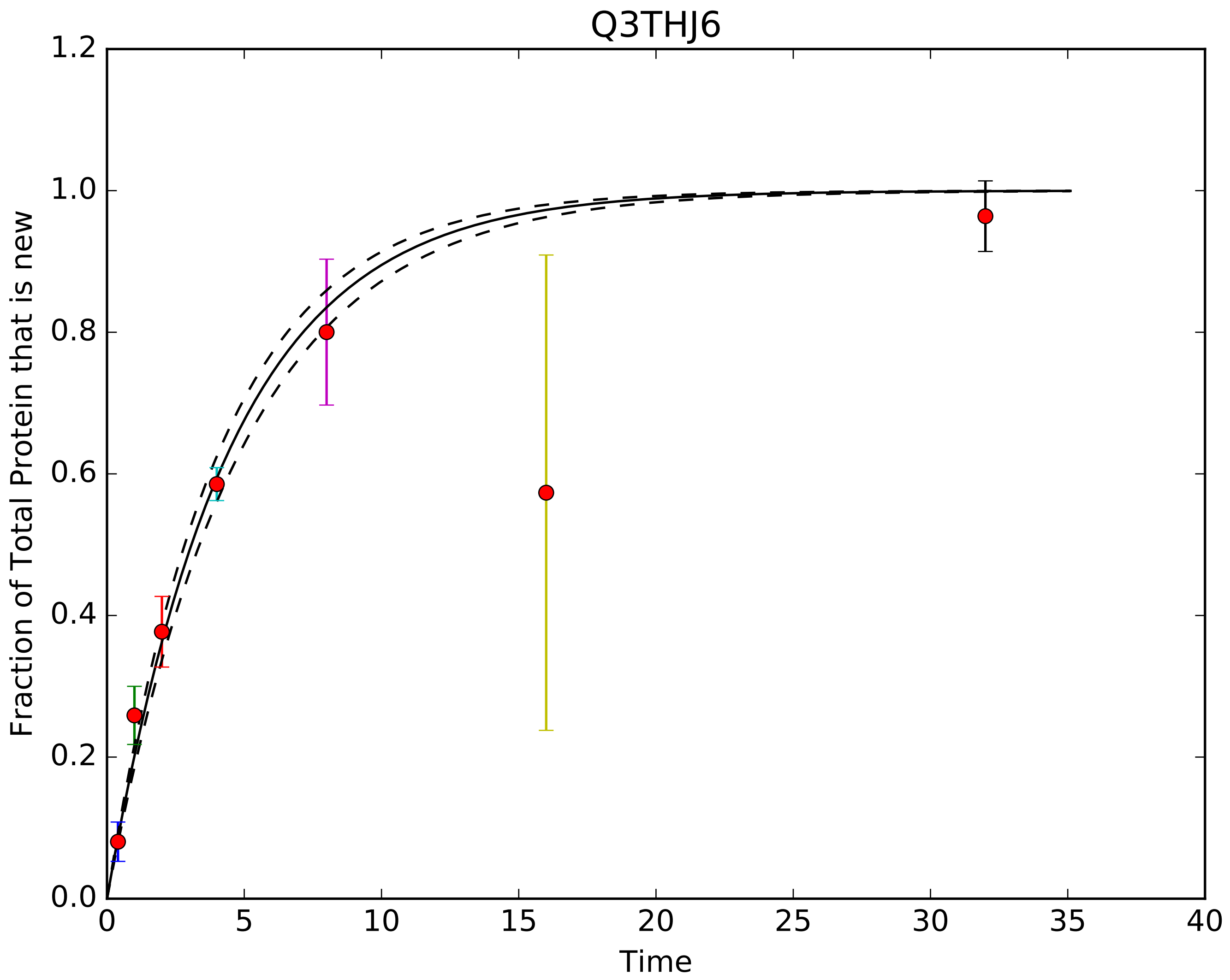

Q3THJ6
Fraction of Total Protein that is new
Time
0.0
0.2
0.4
0.6
0.8
1.0
1.2
0
5
10
15
20
25
30
35
40

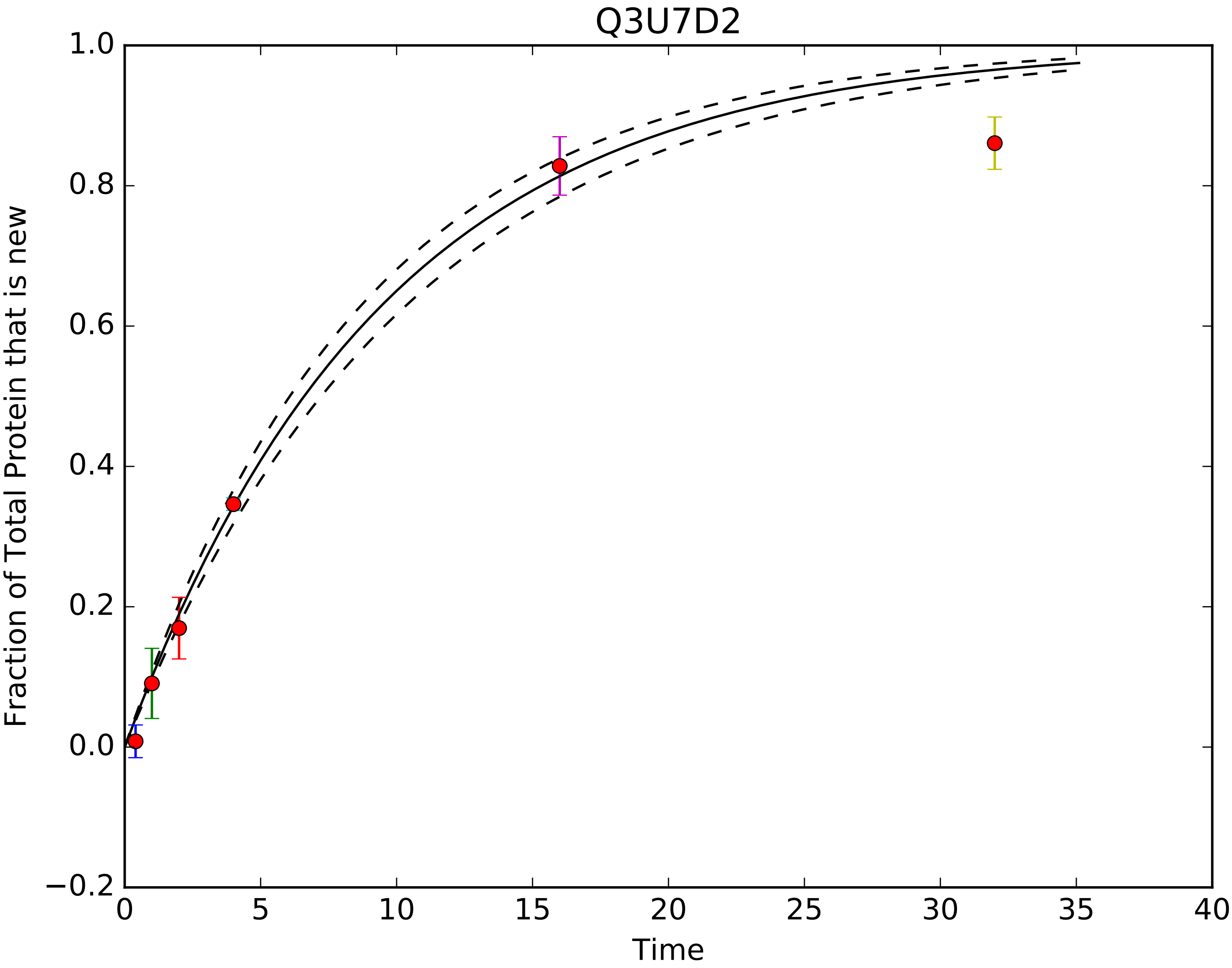
Q3U7D2
Fraction of Total Protein that is new
Time
1.0
0.8
0.6
0.4
0.2
0.0
−0.2
0
5
10
15
20
25
30
35
40

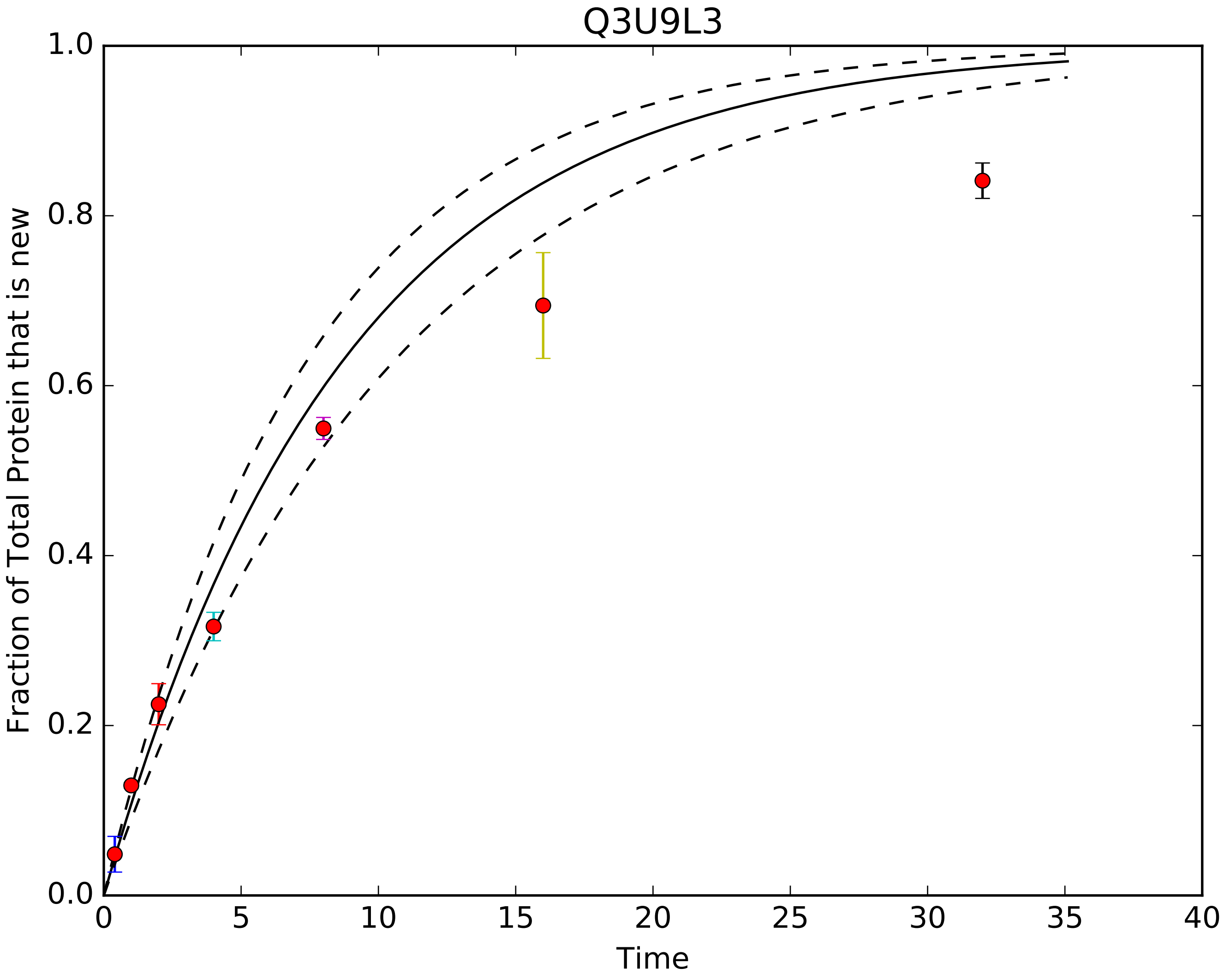
Q3U9L3
Fraction of Total Protein that is new
Time
0.0
0.2
0.4
0.6
0.8
1.0
0
5
10
15
20
25
30
35
40

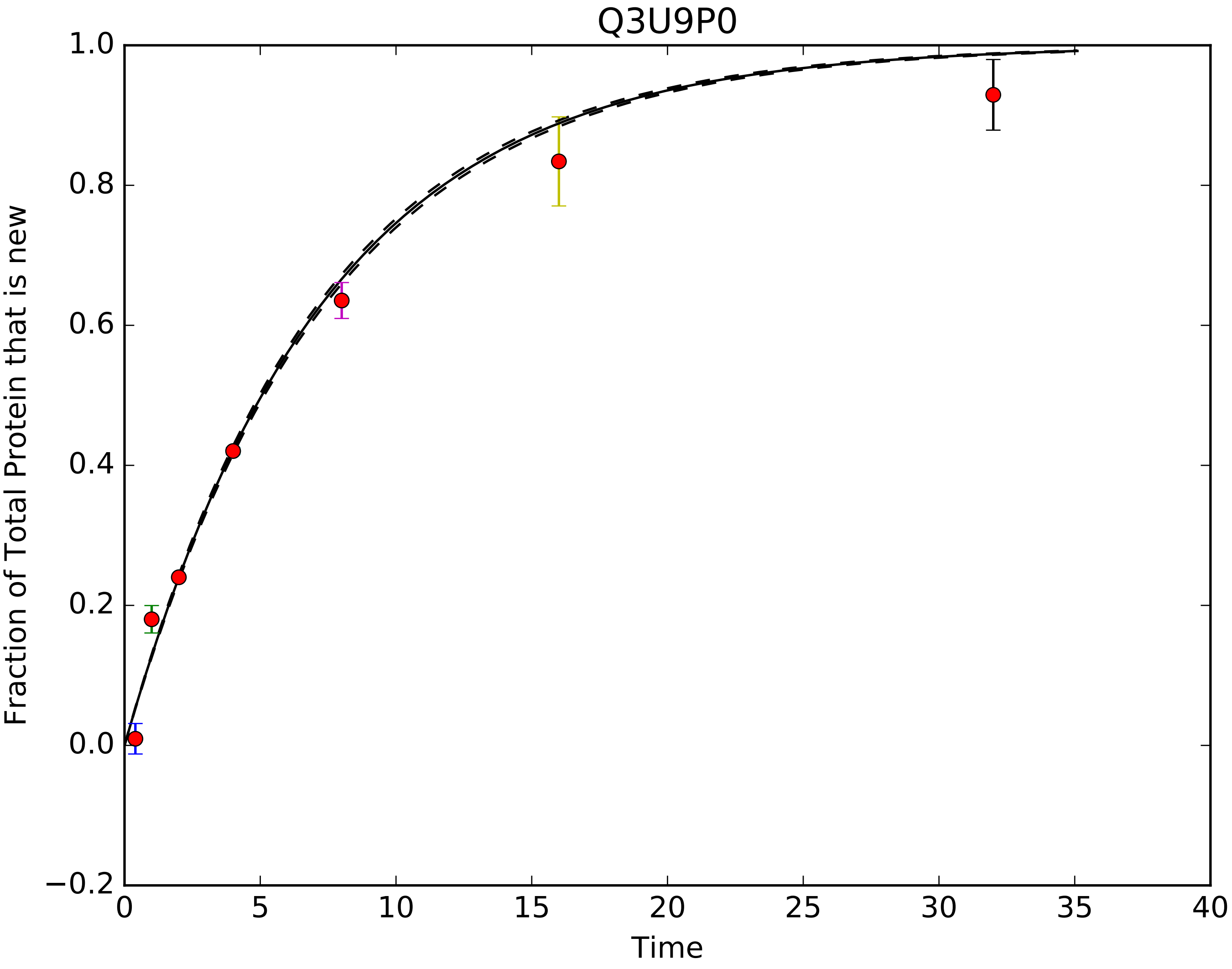
Q3U9P0
Fraction of Total Protein that is new
Time
1.0
0.8
0.6
0.4
0.2
0.0
−0.2
0
5
10
15
20
25
30
35
40

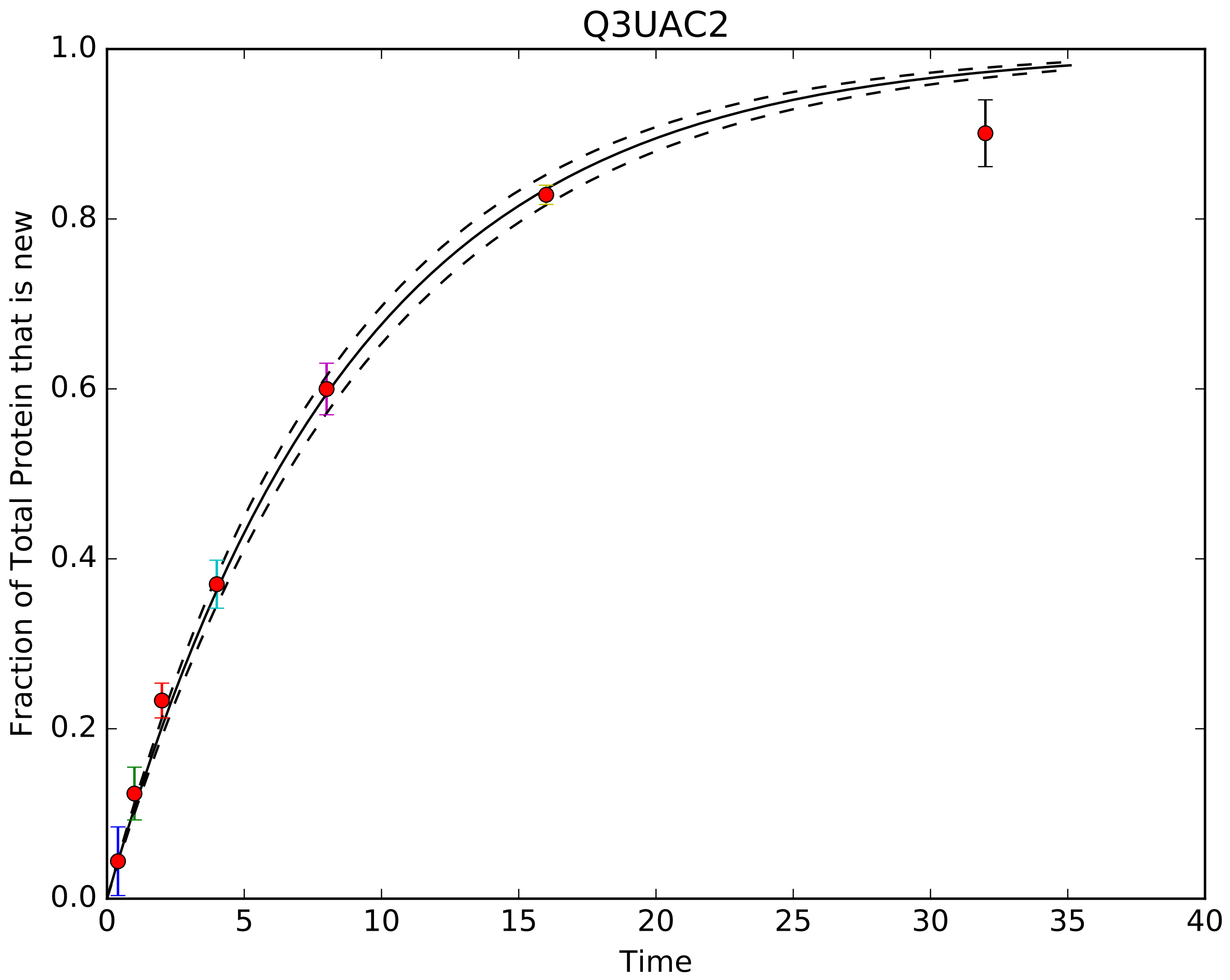
Q3UAC2
Fraction of Total Protein that is new
Time
0.0
0.2
0.4
0.6
0.8
1.0
0
5
10
15
20
25
30
35
40

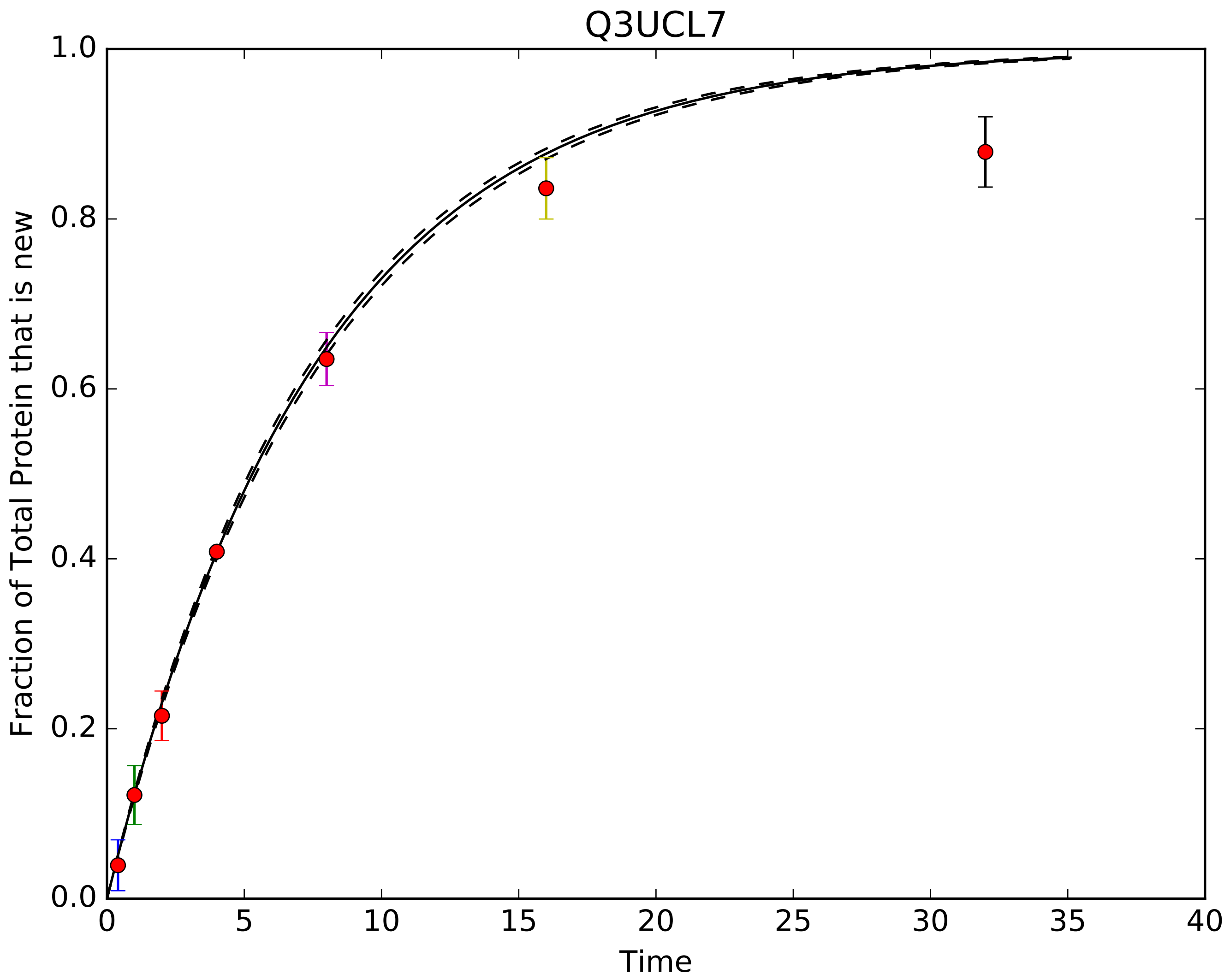

Q3UCL7
Fraction of Total Protein that is new
Time
0.0
0.2
0.4
0.6
0.8
1.0
0
5
10
15
20
25
30
35
40

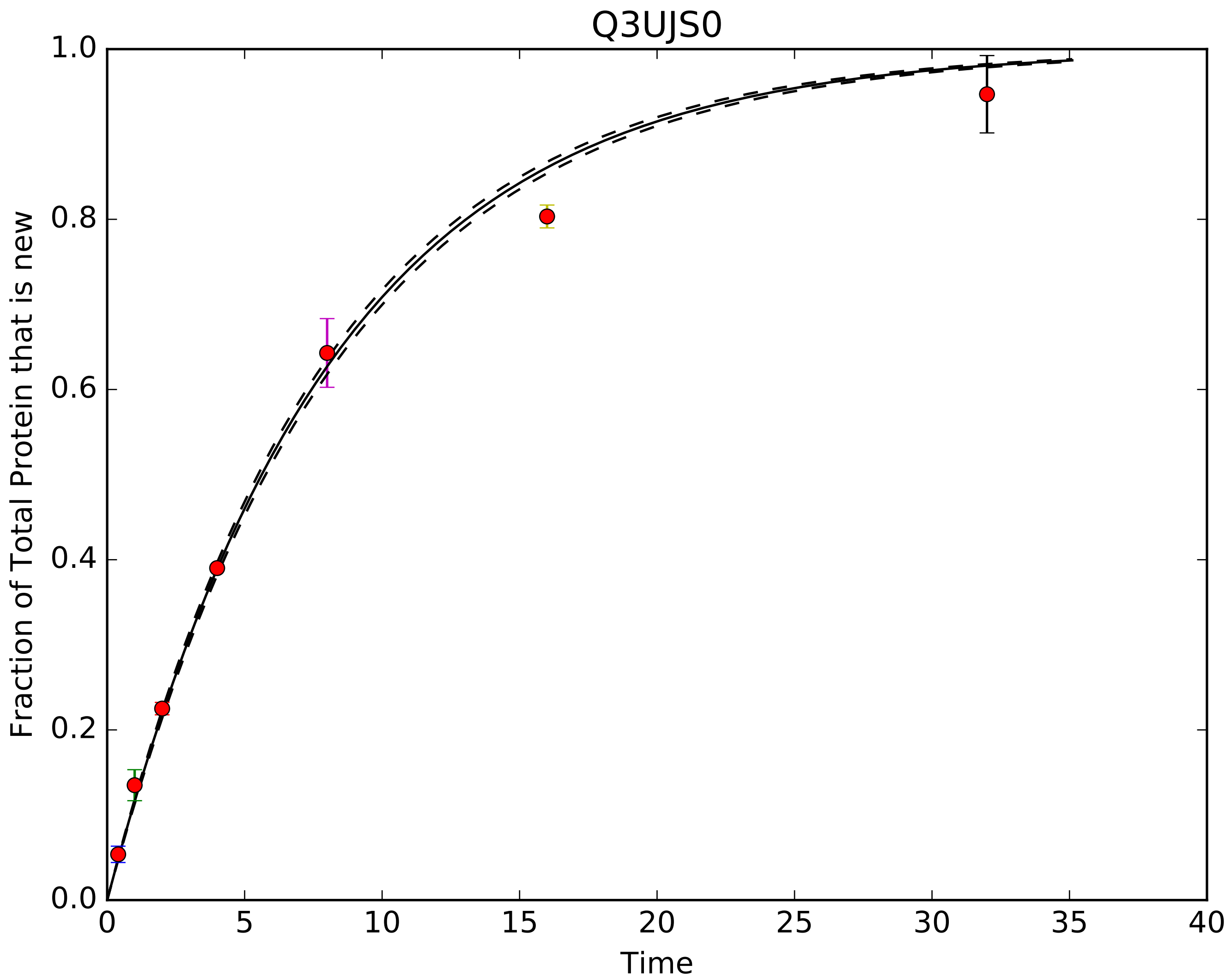

Q3UJS0
Fraction of Total Protein that is new
Time
0.0
0.2
0.4
0.6
0.8
1.0
0
5
10
15
20
25
30
35
40

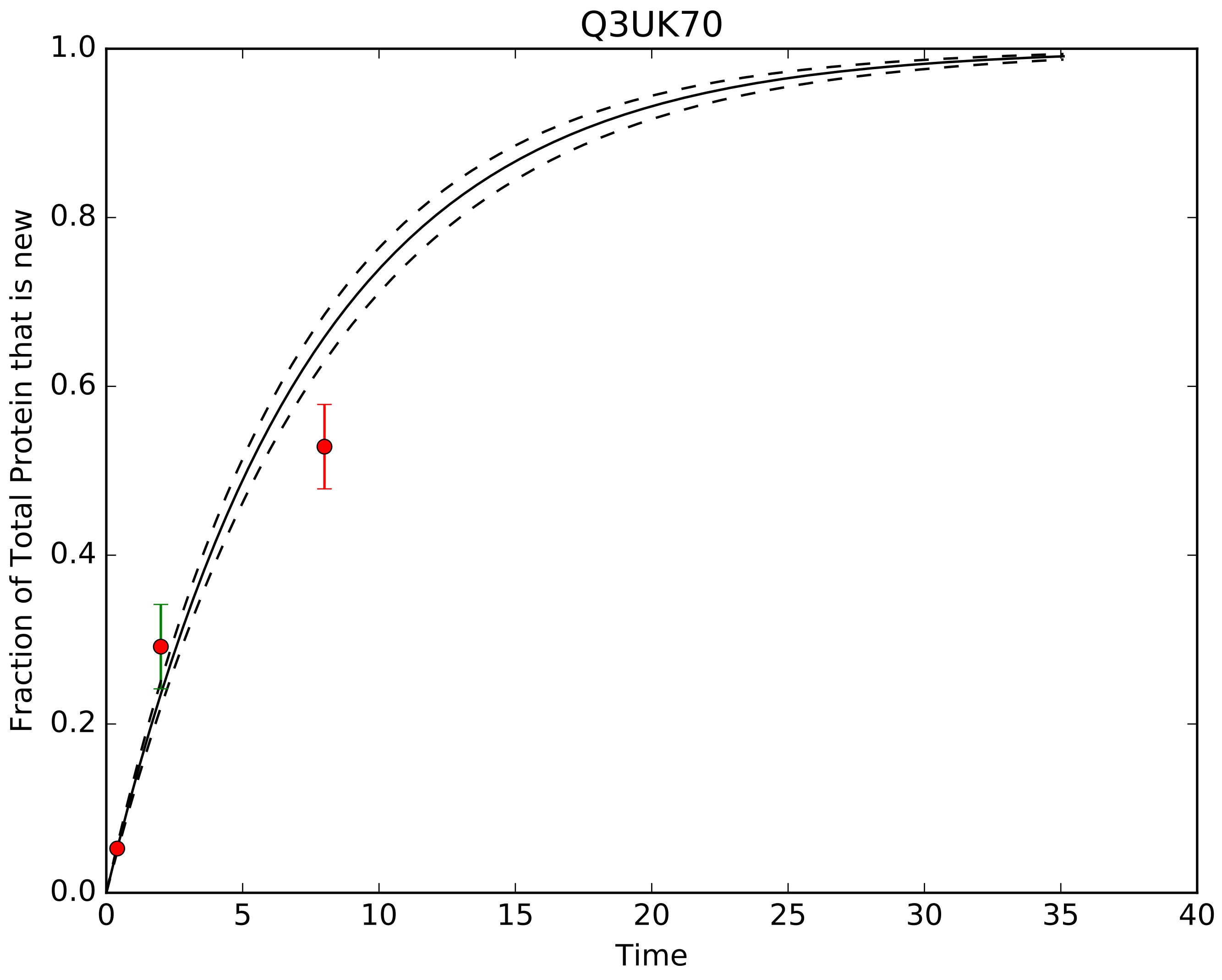

Q3UK70
Fraction of Total Protein that is new
Time
0.0
0.2
0.4
0.6
0.8
1.0
0
5
10
15
20
25
30
35
40

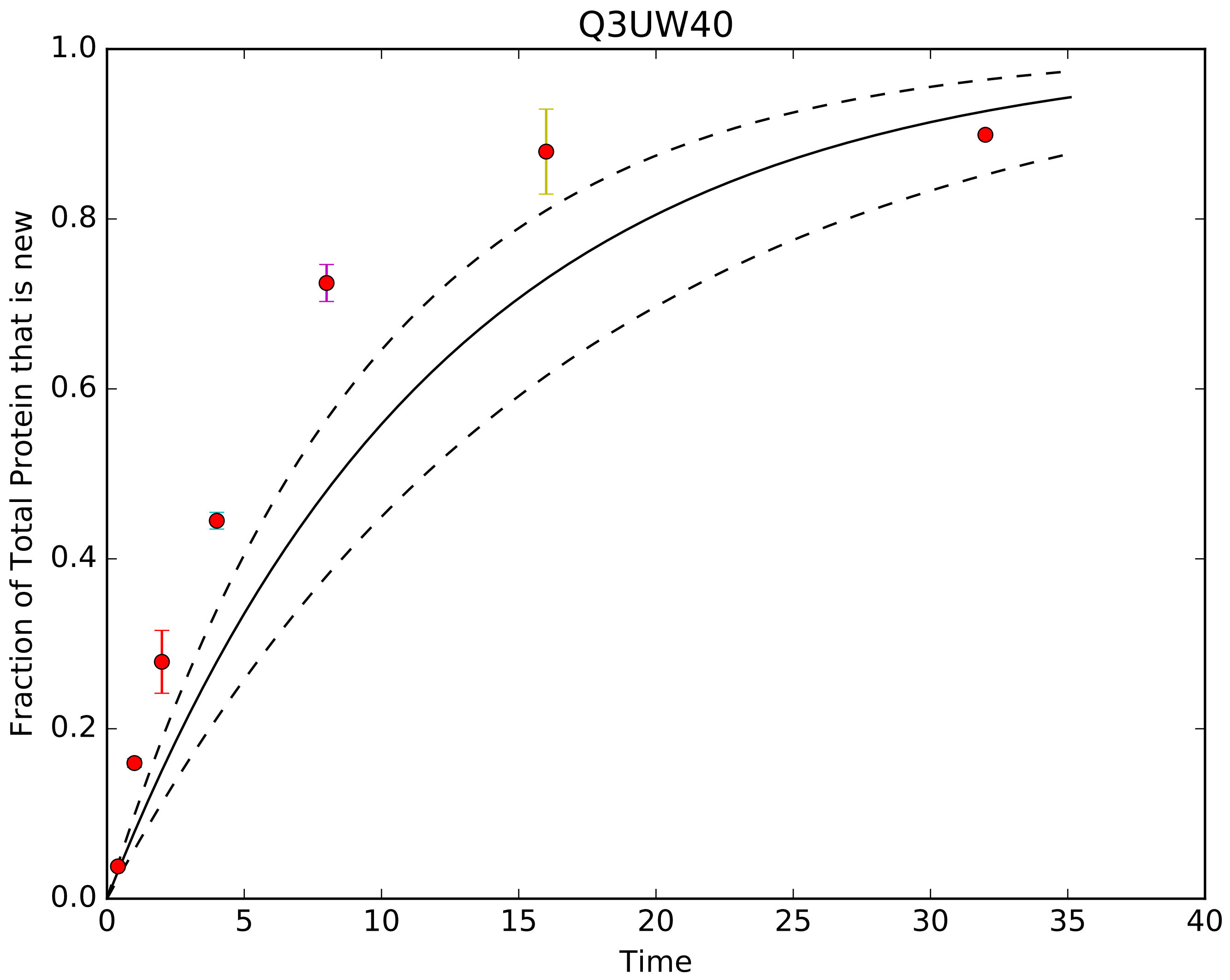
Q3UW40
Fraction of Total Protein that is new
Time
0.0
0.2
0.4
0.6
0.8
1.0
0
5
10
15
20
25
30
35
40

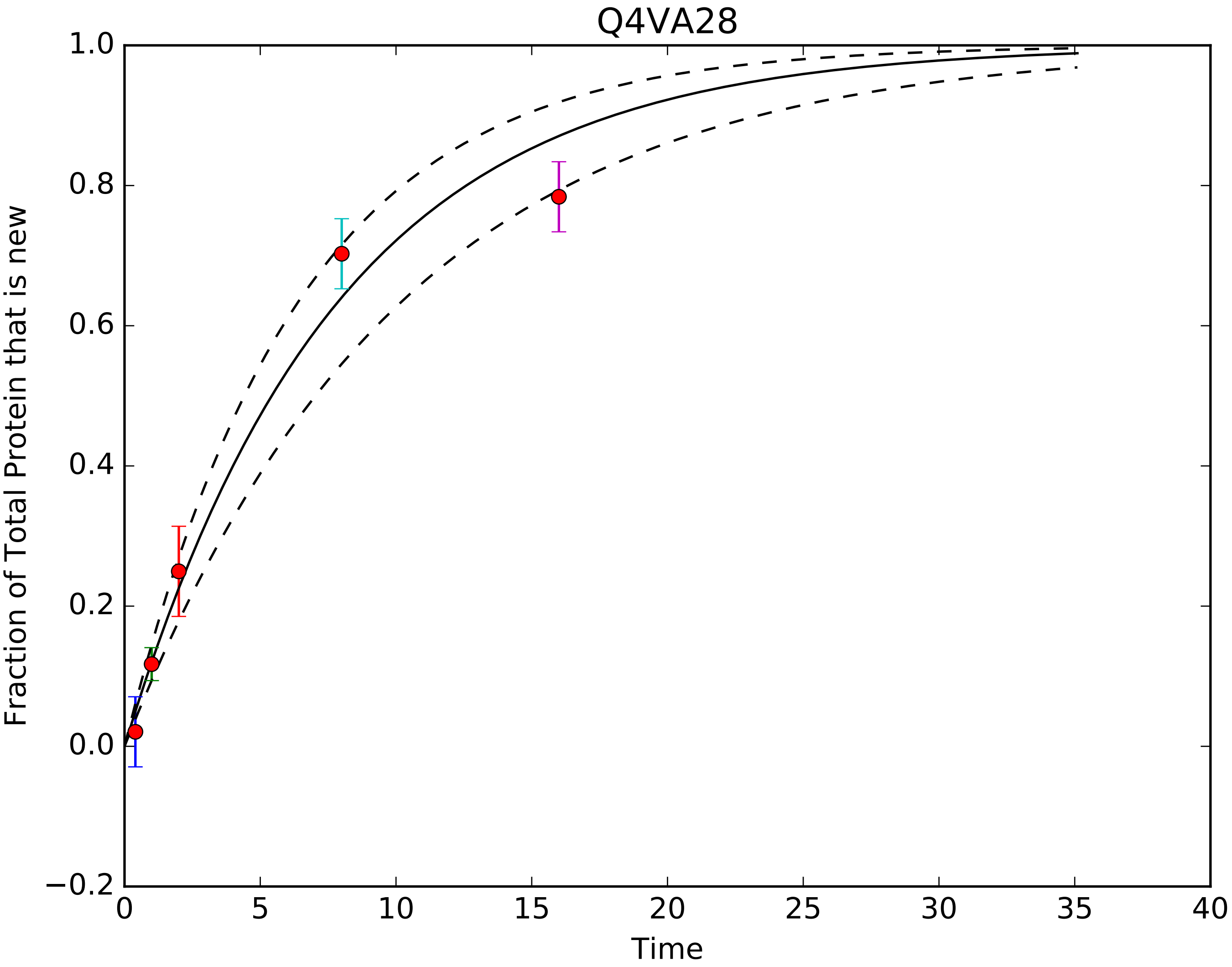
Q4VA28
Fraction of Total Protein that is new
Time
1.0
0.8
0.6
0.4
0.2
0.0
−0.2
0
5
10
15
20
25
30
35
40

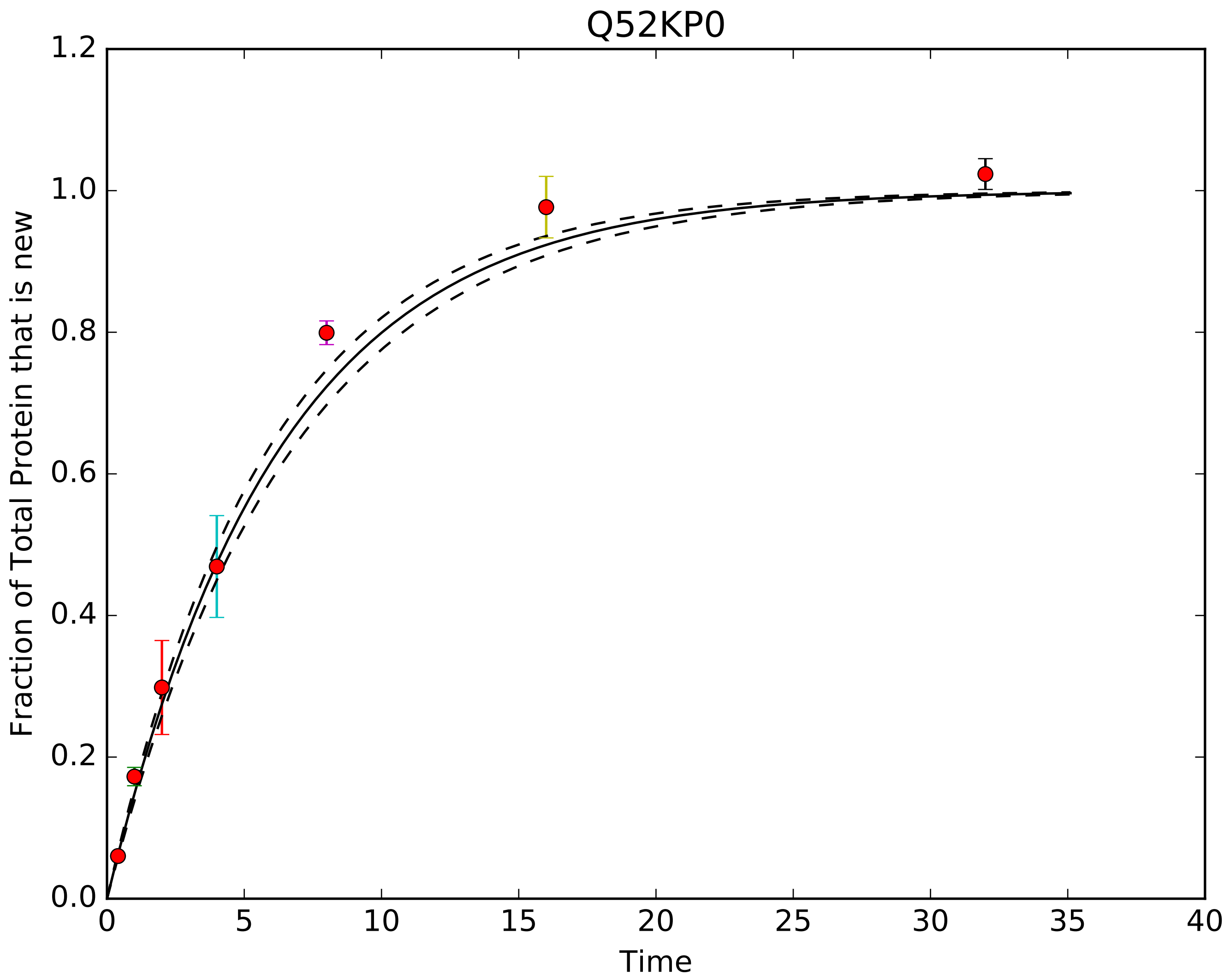

Q52KP0
Fraction of Total Protein that is new
Time
0.0
0.2
0.4
0.6
0.8
1.0
1.2
0
5
10
15
20
25
30
35
40

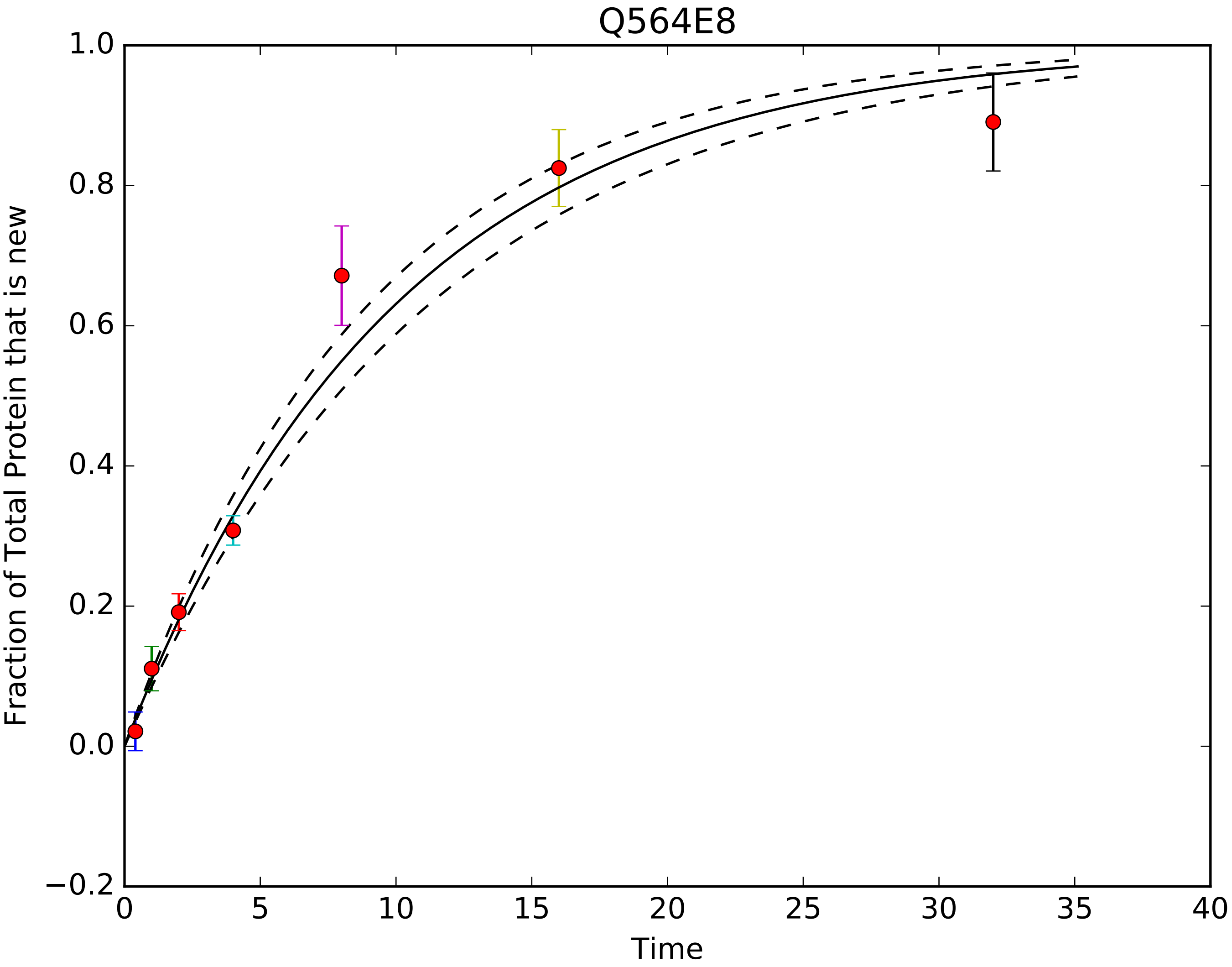

Q564E8
Fraction of Total Protein that is new
Time
1.0
0.8
0.6
0.4
0.2
0.0
−0.2
0
5
10
15
20
25
30
35
40

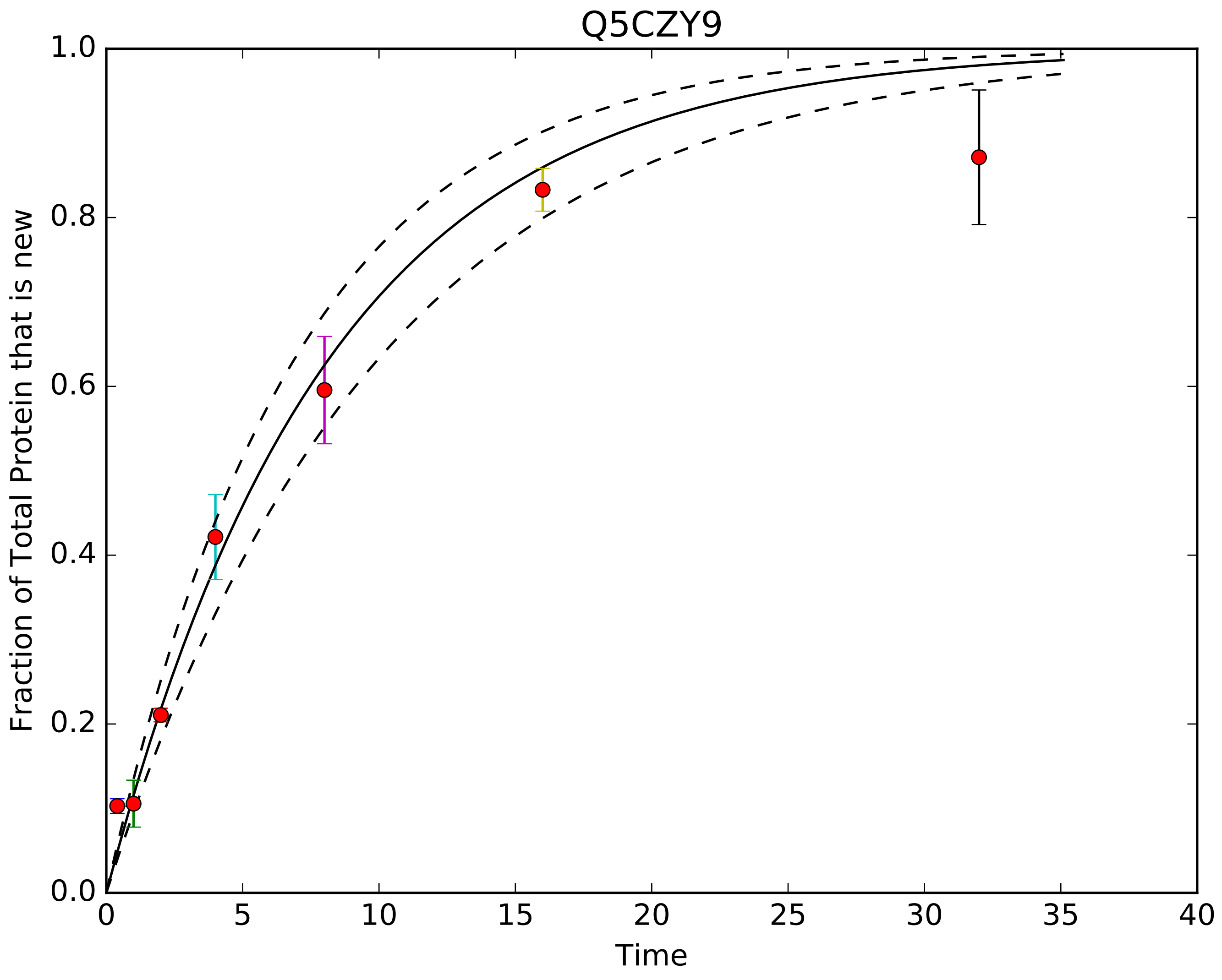

Q5CZY9
Fraction of Total Protein that is new
Time
0.0
0.2
0.4
0.6
0.8
1.0
0
5
10
15
20
25
30
35
40

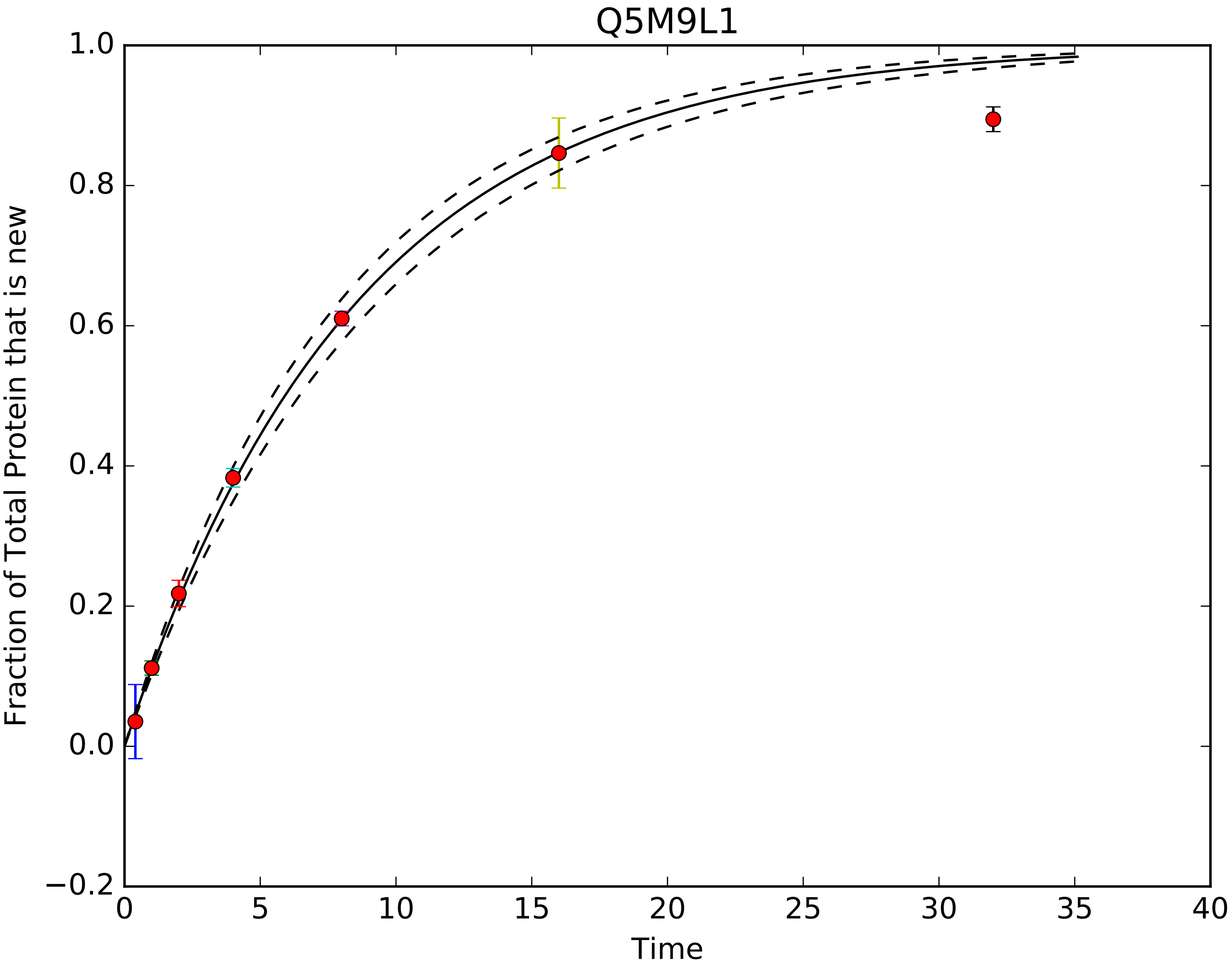
Q5M9L1
Fraction of Total Protein that is new
Time
1.0
0.8
0.6
0.4
0.2
0.0
−0.2
0
5
10
15
20
25
30
35
40

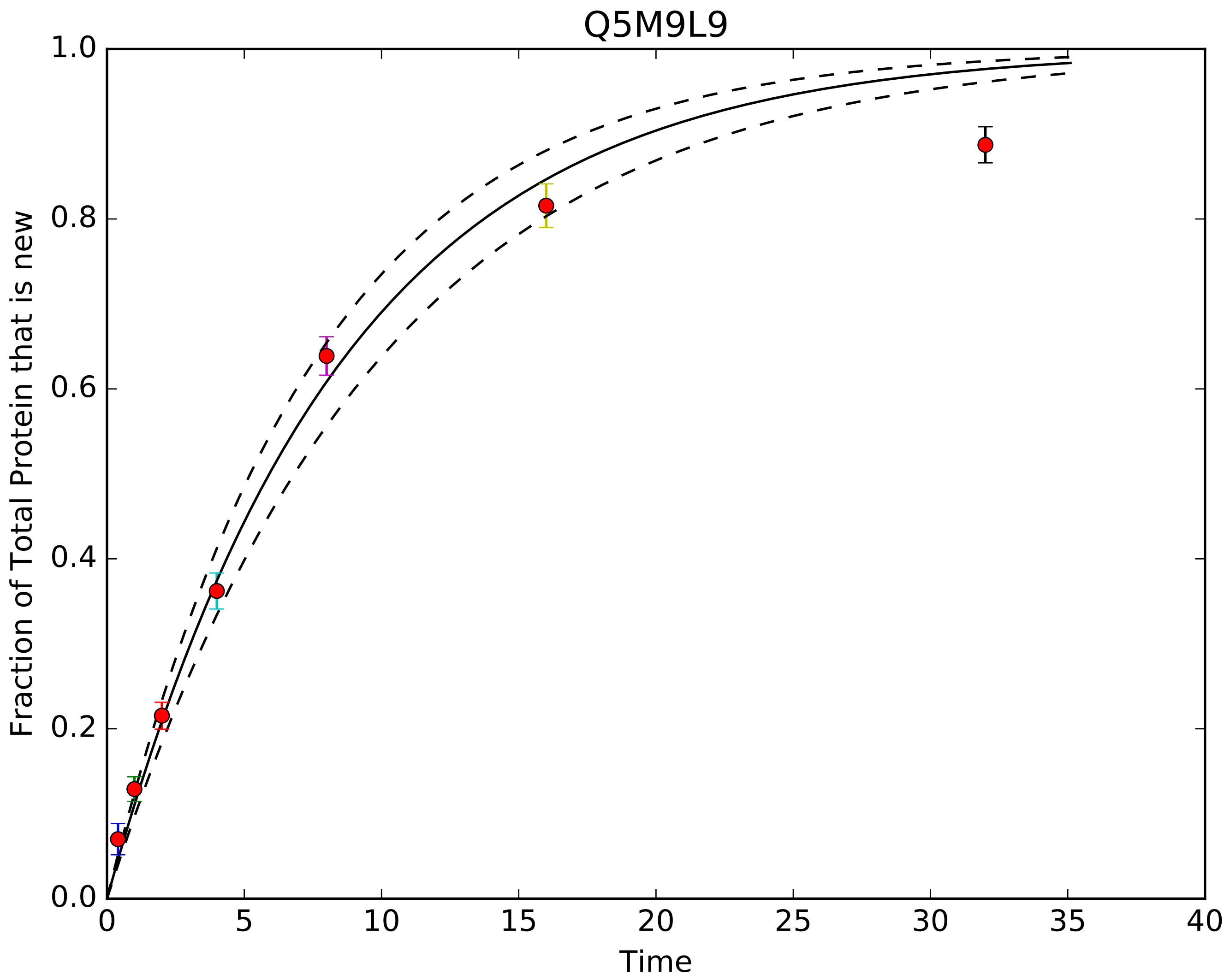
Q5M9L9
Fraction of Total Protein that is new
Time
0.0
0.2
0.4
0.6
0.8
1.0
0
5
10
15
20
25
30
35
40

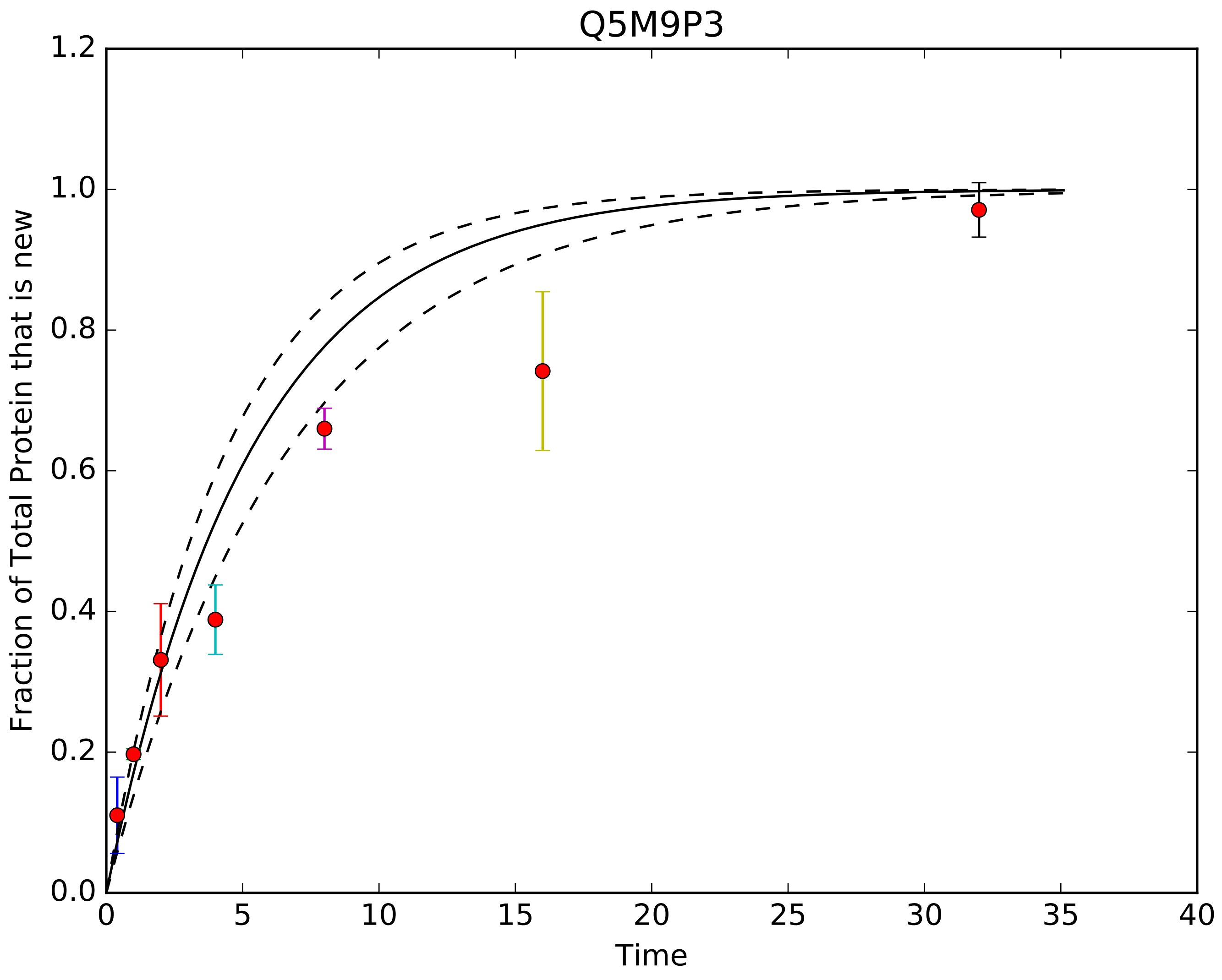

Q5M9P3
Fraction of Total Protein that is new
Time
0.0
0.2
0.4
0.6
0.8
1.0
1.2
0
5
10
15
20
25
30
35
40

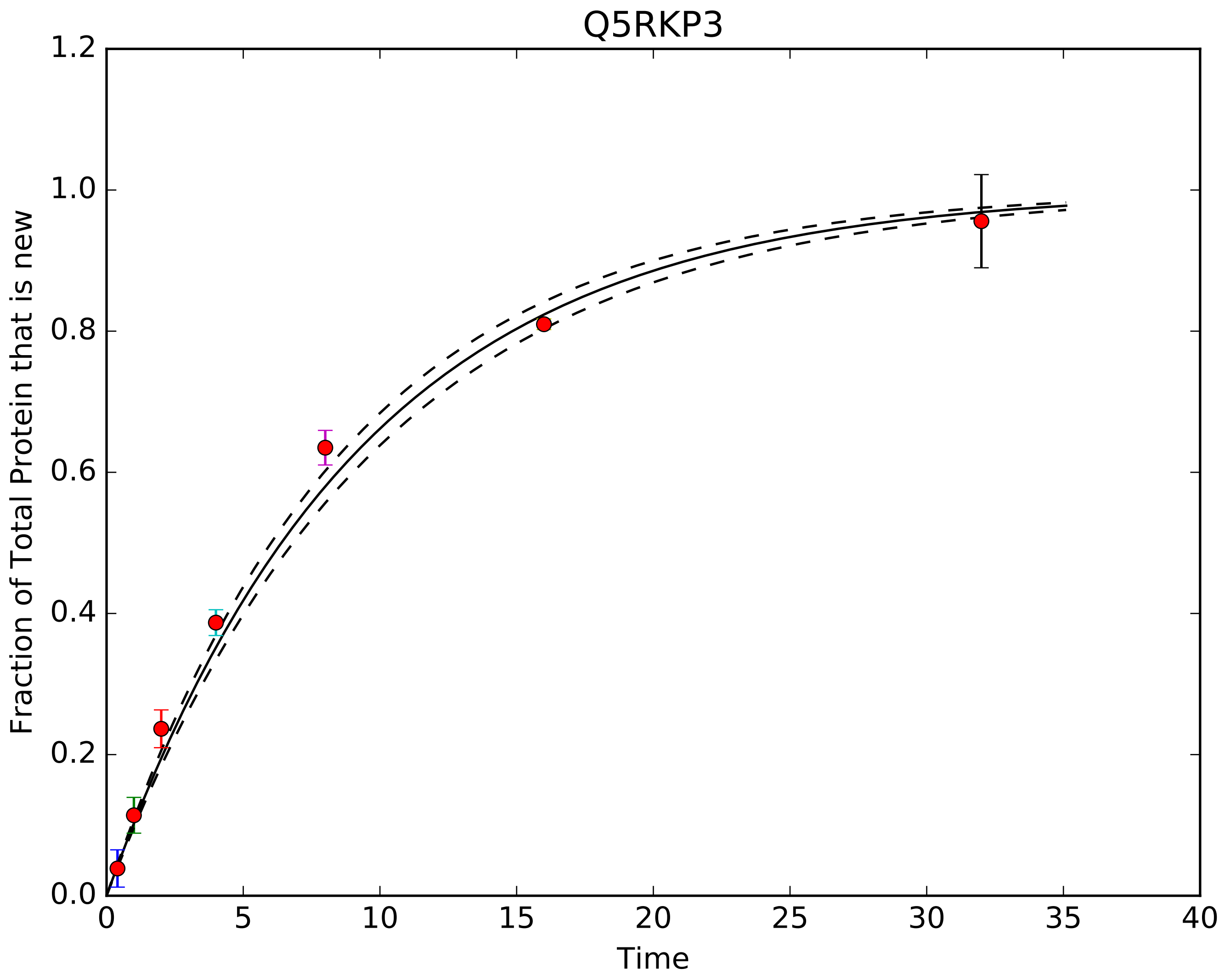
Q5RKP3
Fraction of Total Protein that is new
Time
0.0
0.2
0.4
0.6
0.8
1.0
1.2
0
5
10
15
20
25
30
35
40

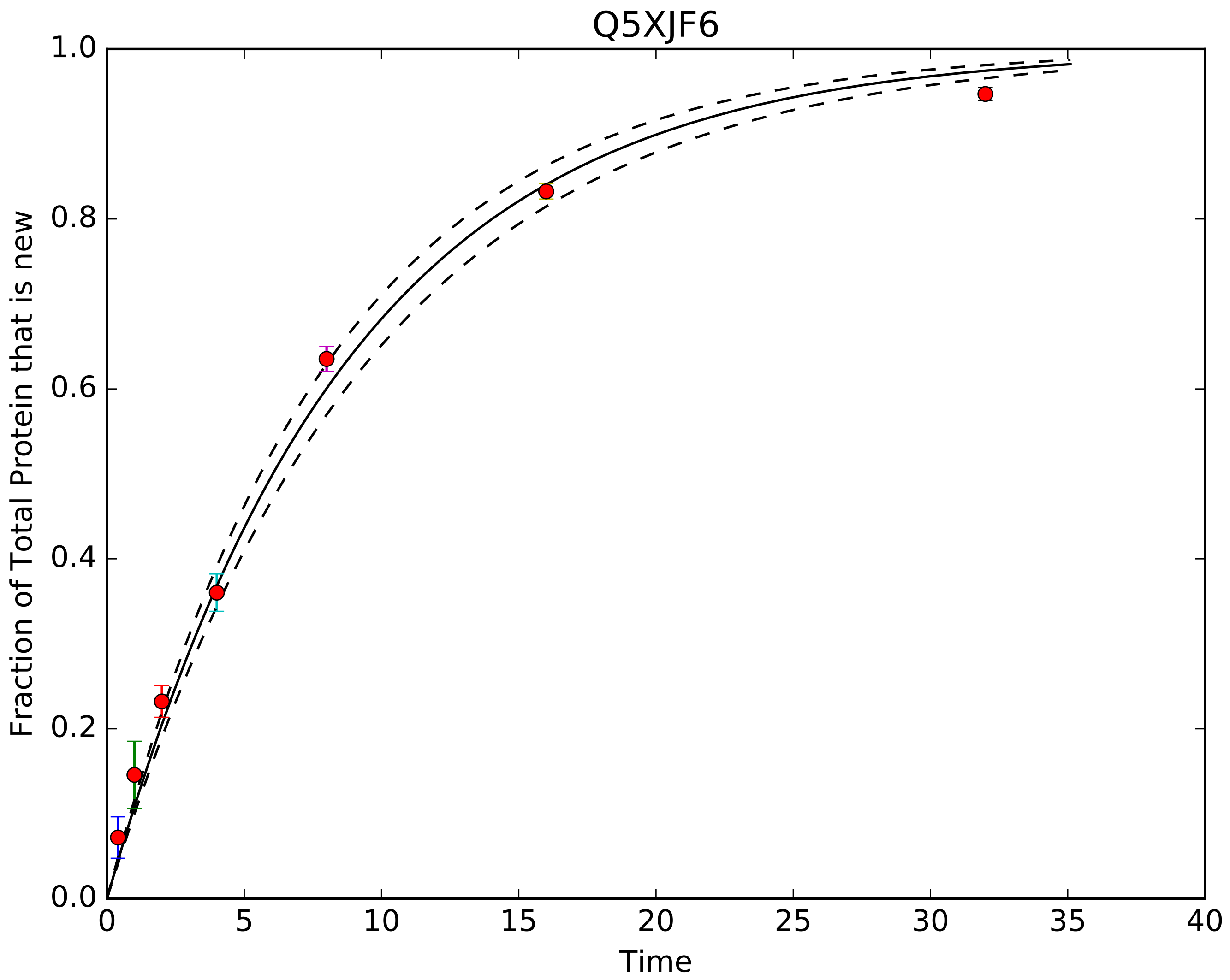

Q5XJF6
Fraction of Total Protein that is new
Time
0.0
0.2
0.4
0.6
0.8
1.0
0
5
10
15
20
25
30
35
40

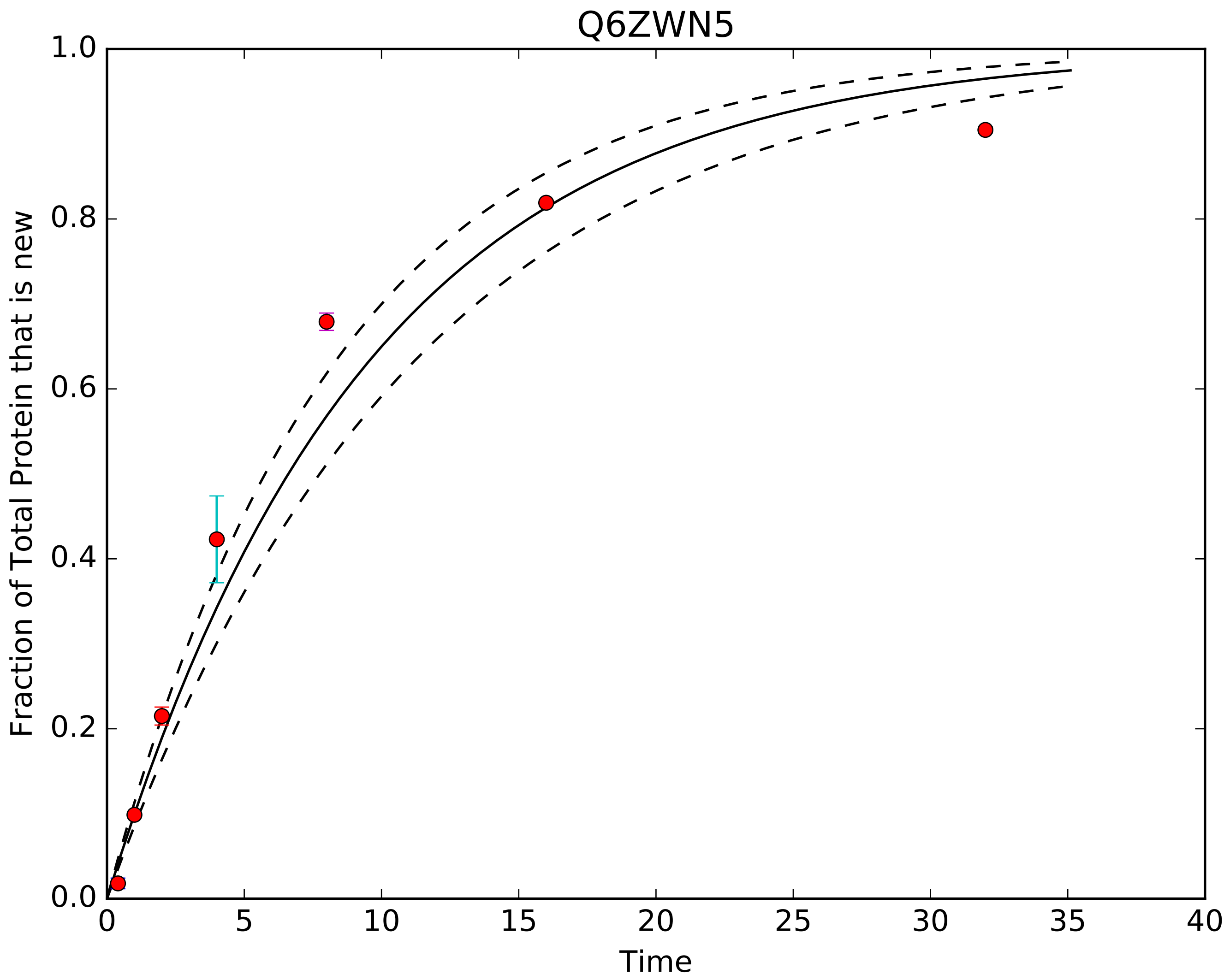

Q6ZWN5
Fraction of Total Protein that is new
Time
1.0
0.8
0.6
0.4
0.2
0.0
0
5
10
15
20
25
30
35
40

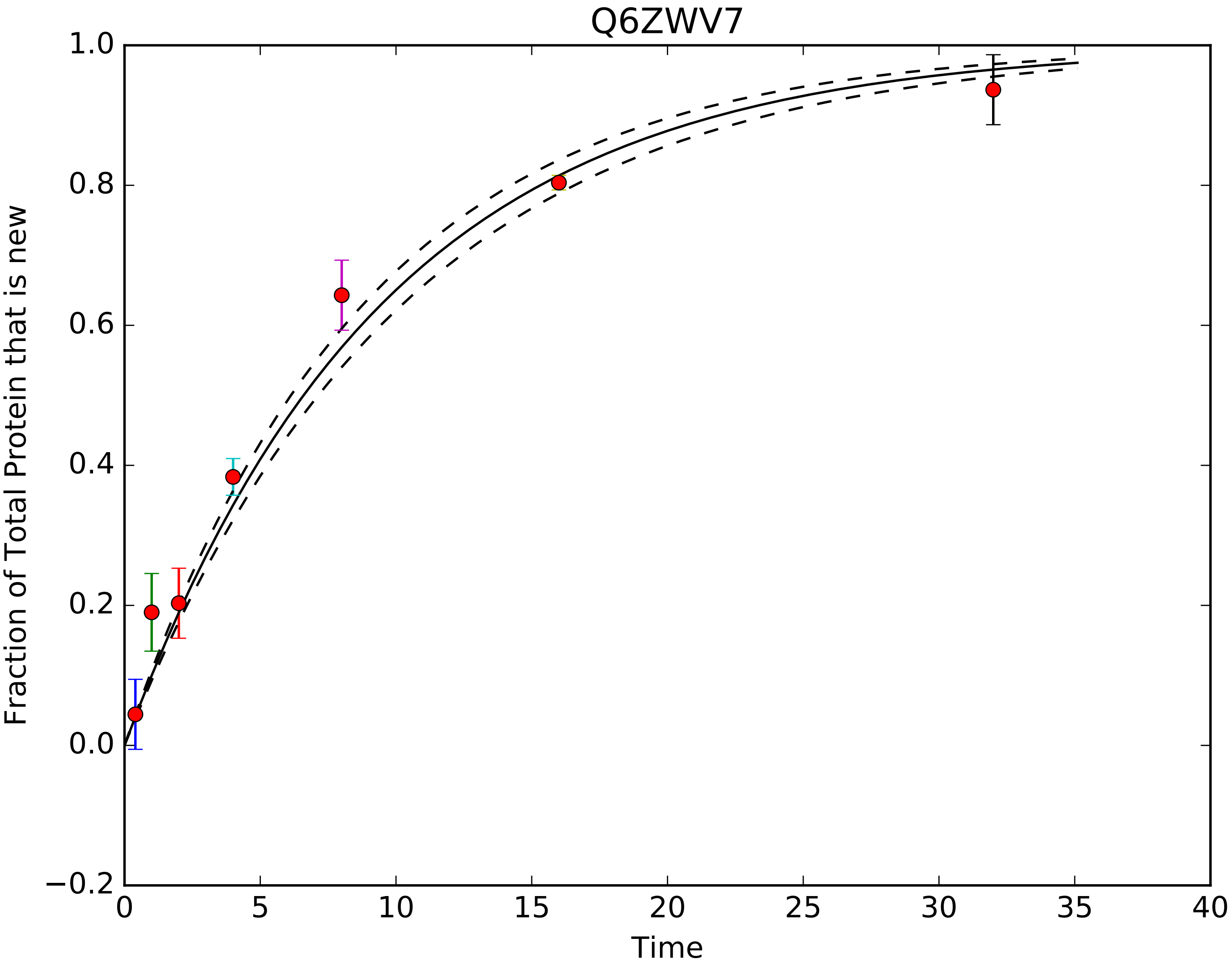
Q6ZWV7
Fraction of Total Protein that is new
Time
1.0
0.8
0.6
0.4
0.2
0.0
−0.2
0
5
10
15
20
25
30
35
40

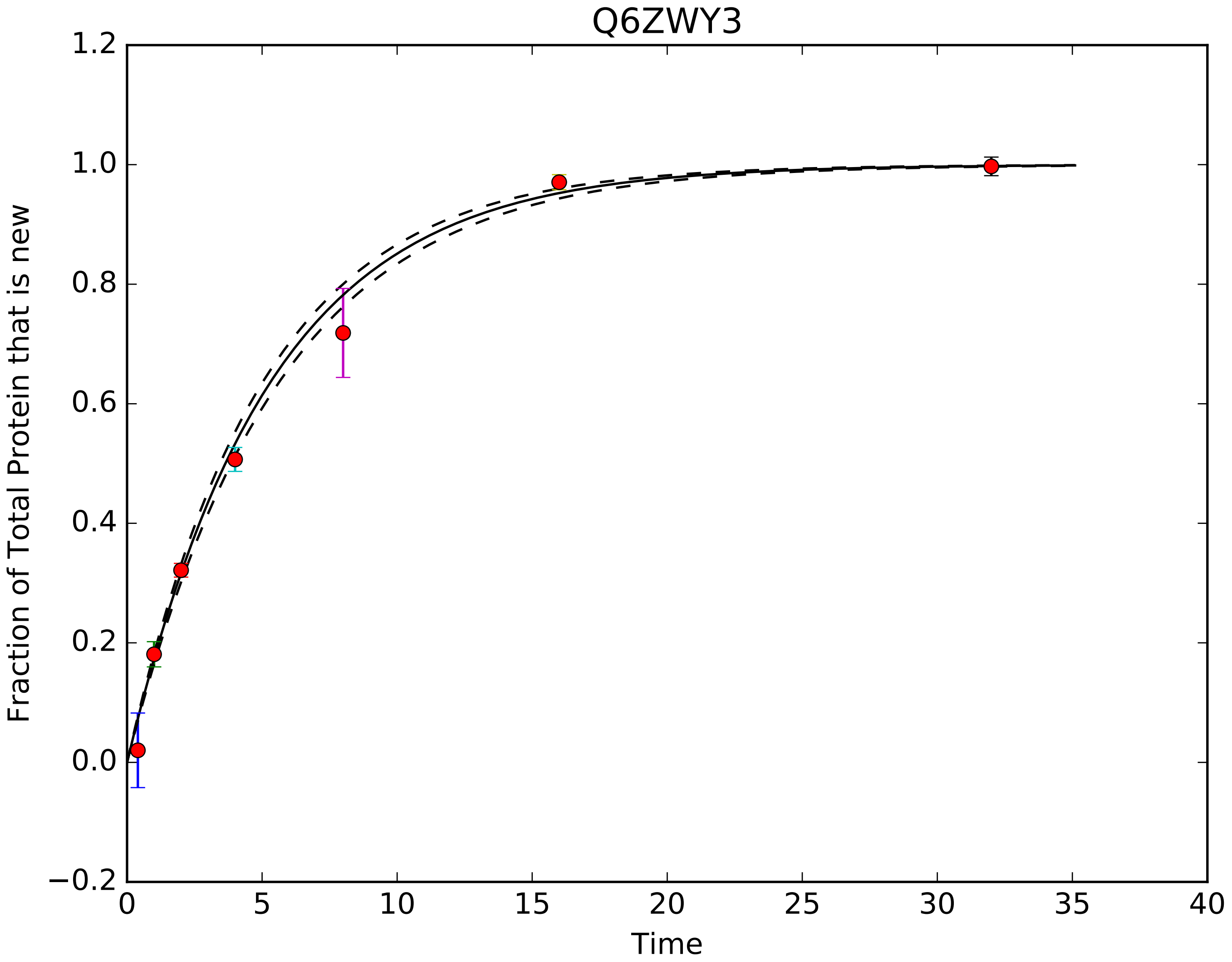

Q6ZWY3
Fraction of Total Protein that is new
Time
1.2
1.0
0.8
0.6
0.4
0.2
0.0
−0.2
0
5
10
15
20
25
30
35
40

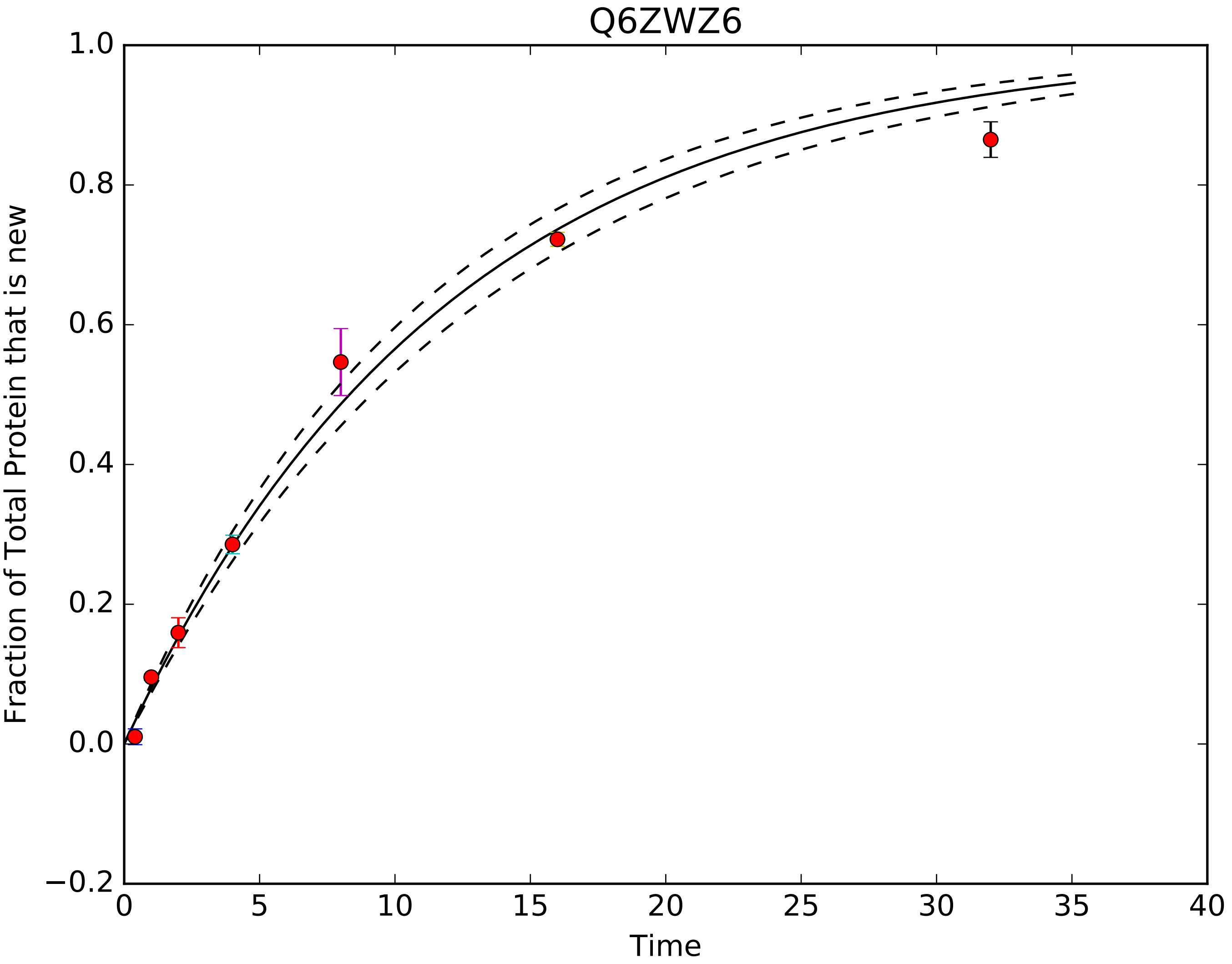
Q6ZWZ6
Fraction of Total Protein that is new
Time
1.0
0.8
0.6
0.4
0.2
0.0
−0.2
0
5
10
15
20
25
30
35
40

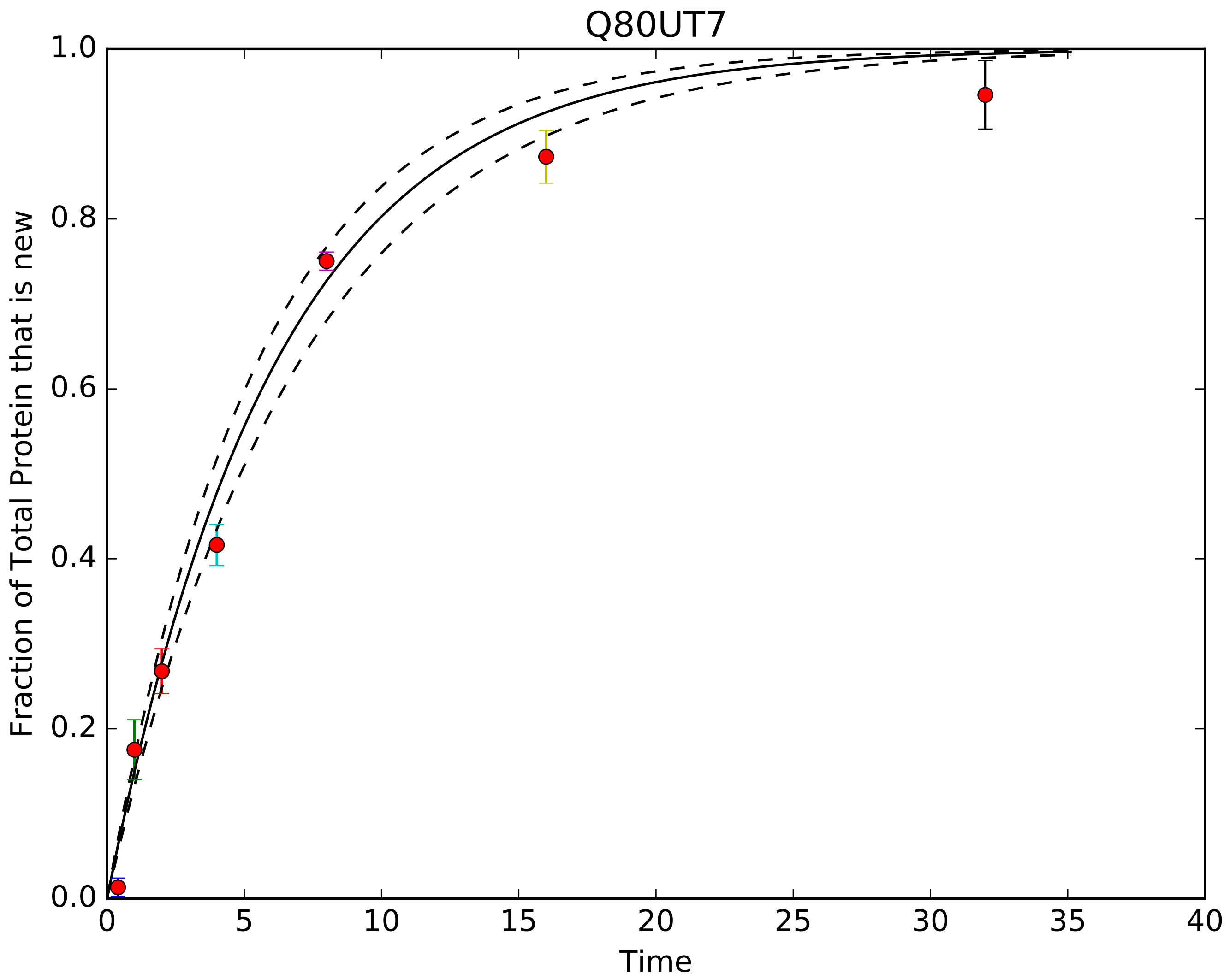
Q80UT7
Fraction of Total Protein that is new
Time
0.0
0.2
0.4
0.6
0.8
1.0
0
5
10
15
20
25
30
35
40

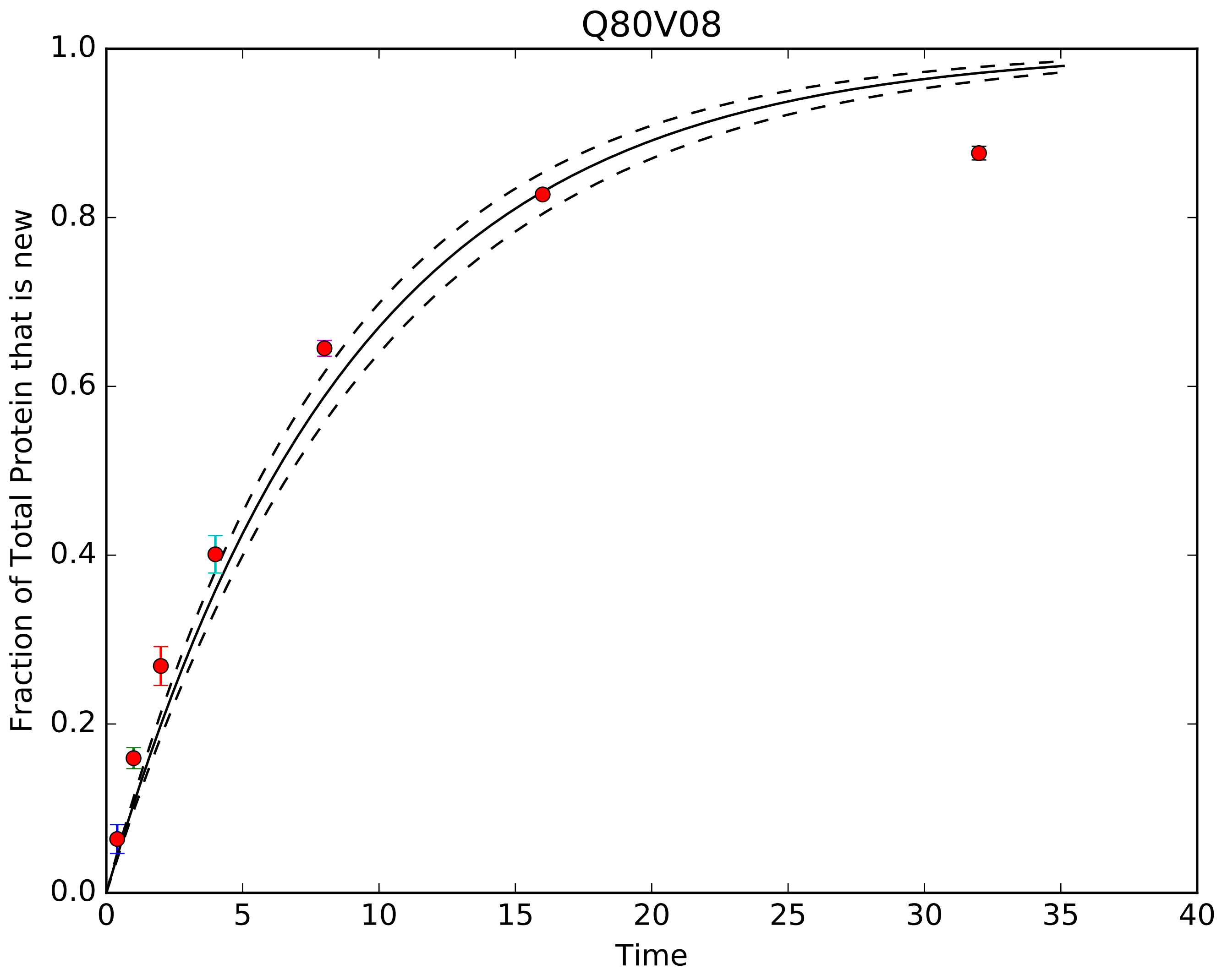

Q80V08
Fraction of Total Protein that is new
Time
0.0
0.2
0.4
0.6
0.8
1.0
0
5
10
15
20
25
30
35
40

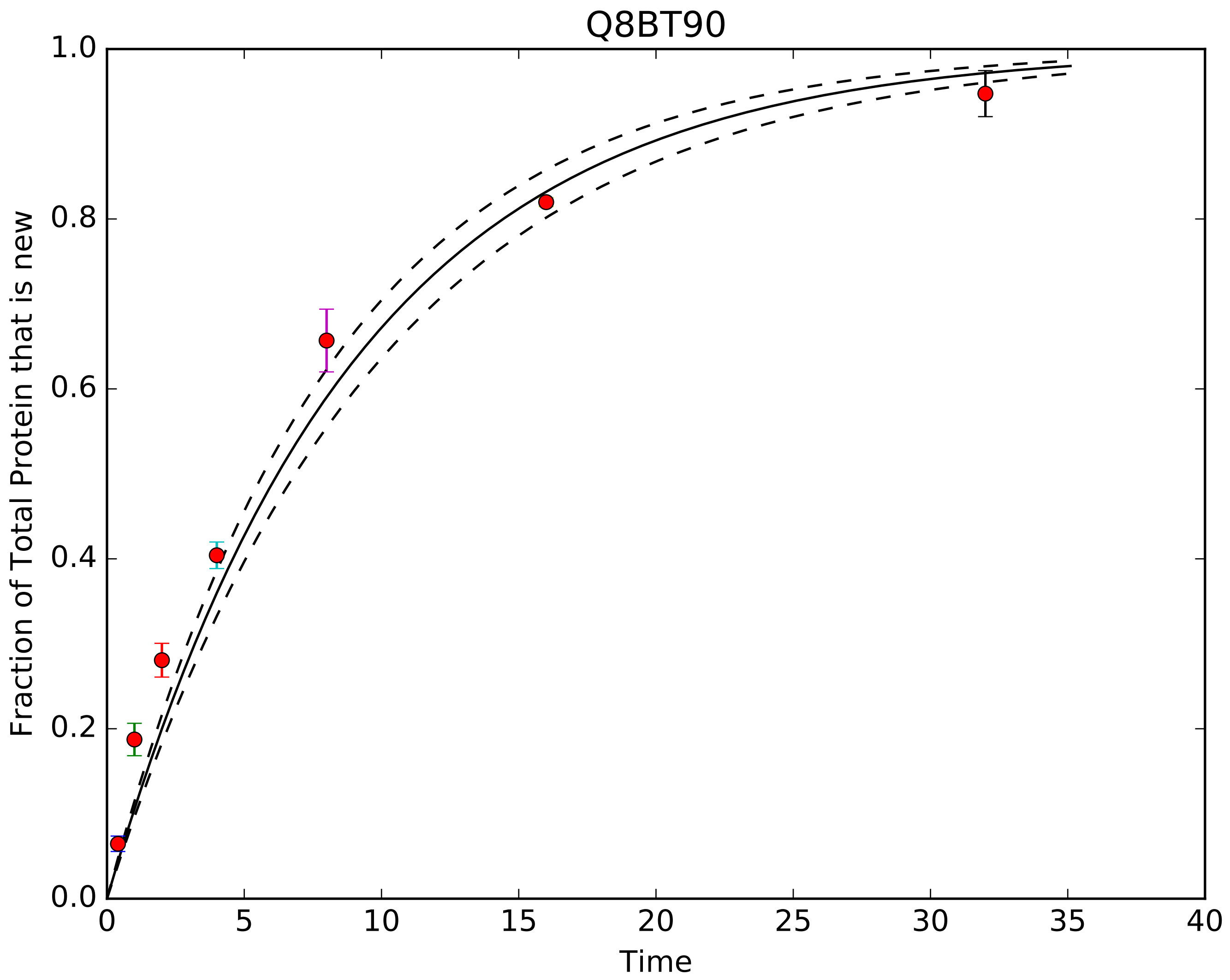
Q8BT90
Fraction of Total Protein that is new
Time
1.0
0.8
0.6
0.4
0.2
0.0
0
5
10
15
20
25
30
35
40

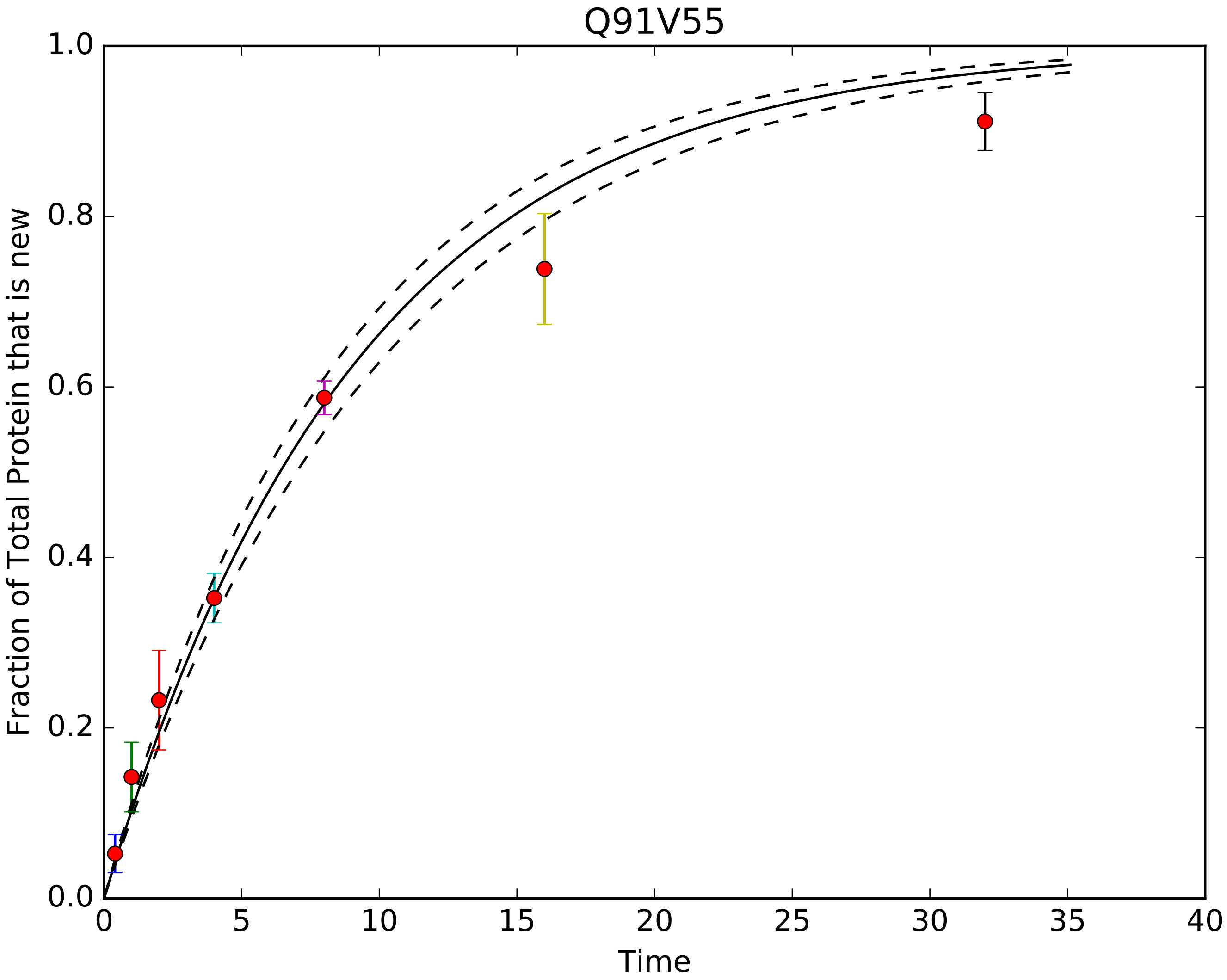
Q91V55
Fraction of Total Protein that is new
Time
0.0
0.2
0.4
0.6
0.8
1.0
0
5
10
15
20
25
30
35
40

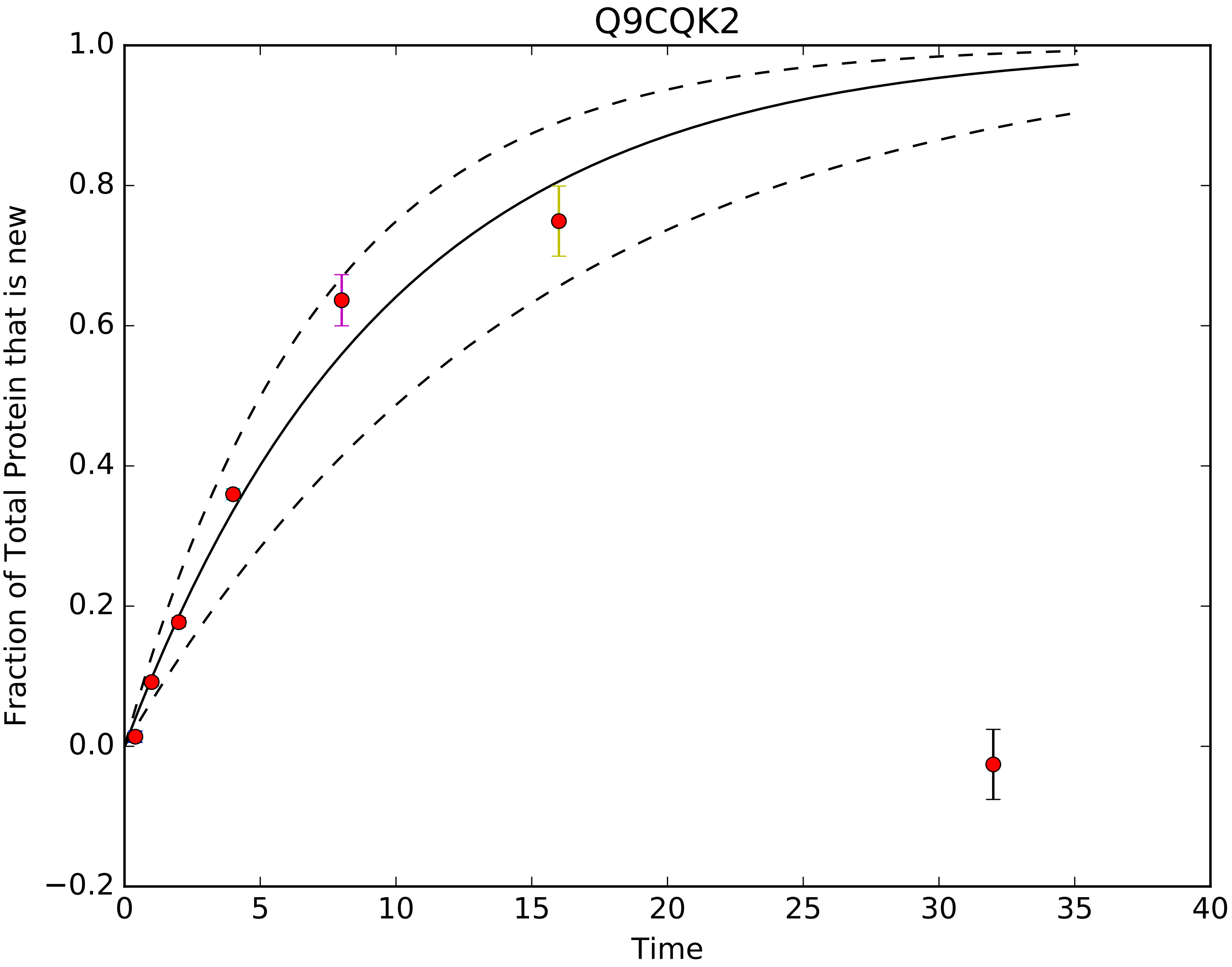
Q9CQK2
Fraction of Total Protein that is new
Time
1.0
0.8
0.6
0.4
0.2
0.0
−0.2
0
5
10
15
20
25
30
35
40

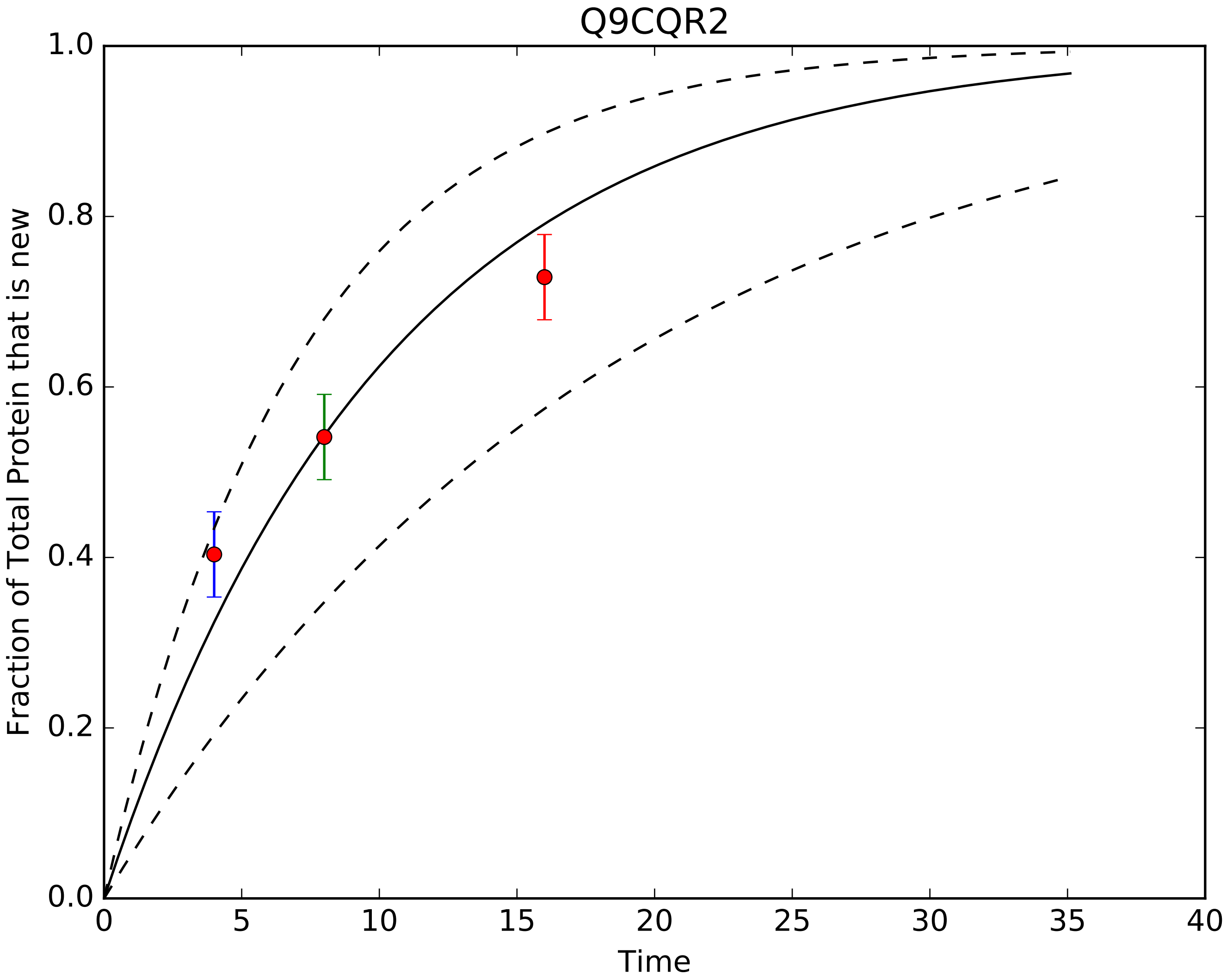

Q9CQR2
Fraction of Total Protein that is new
Time
0.0
0.2
0.4
0.6
0.8
1.0
0
5
10
15
20
25
30
35
40

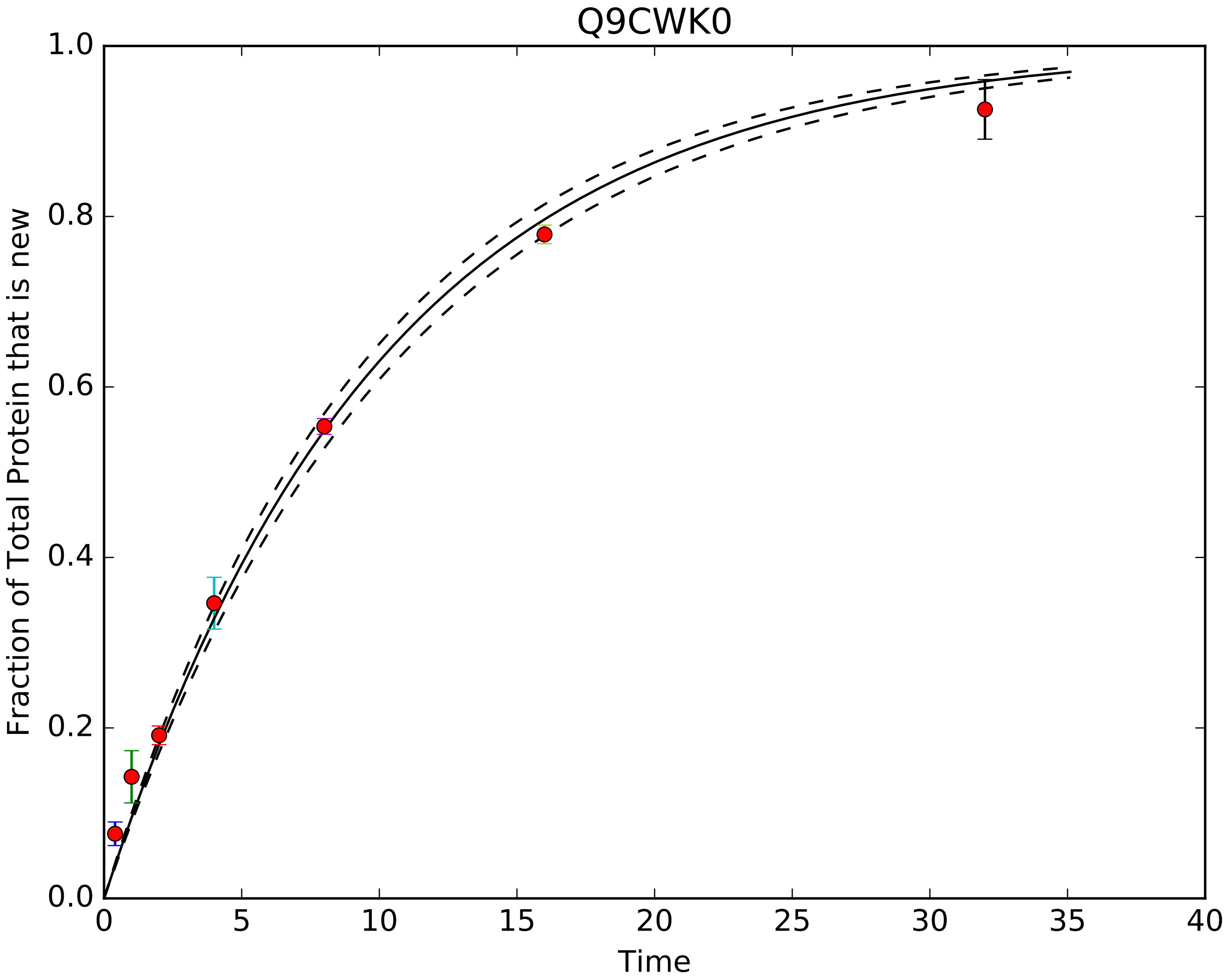
Q9CWK0
Fraction of Total Protein that is new
Time
0.0
0.2
0.4
0.6
0.8
1.0
0
5
10
15
20
25
30
35
40

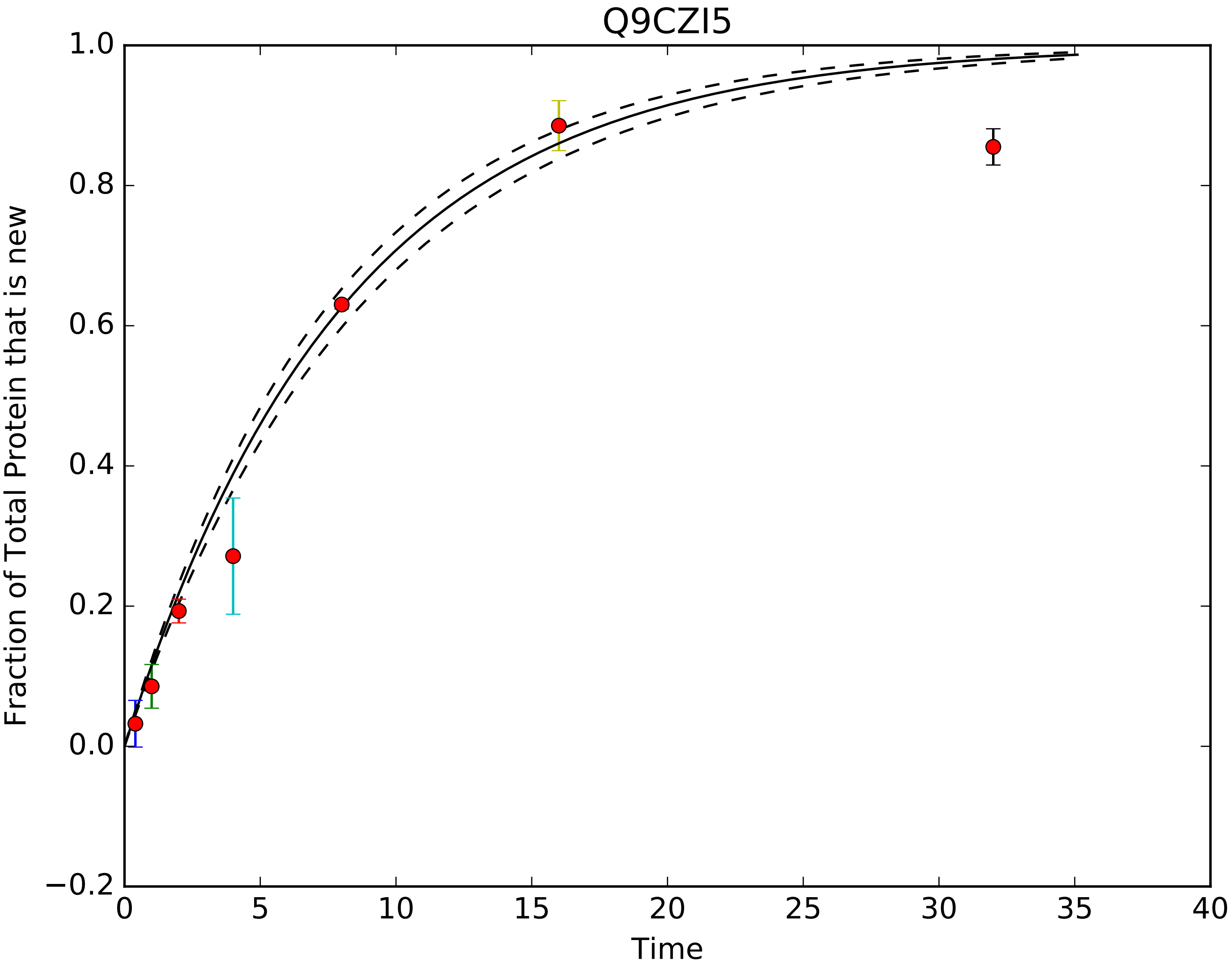

Q9CZI5
Fraction of Total Protein that is new
Time
1.0
0.8
0.6
0.4
0.2
0.0
−0.2
0
5
10
15
20
25
30
35
40

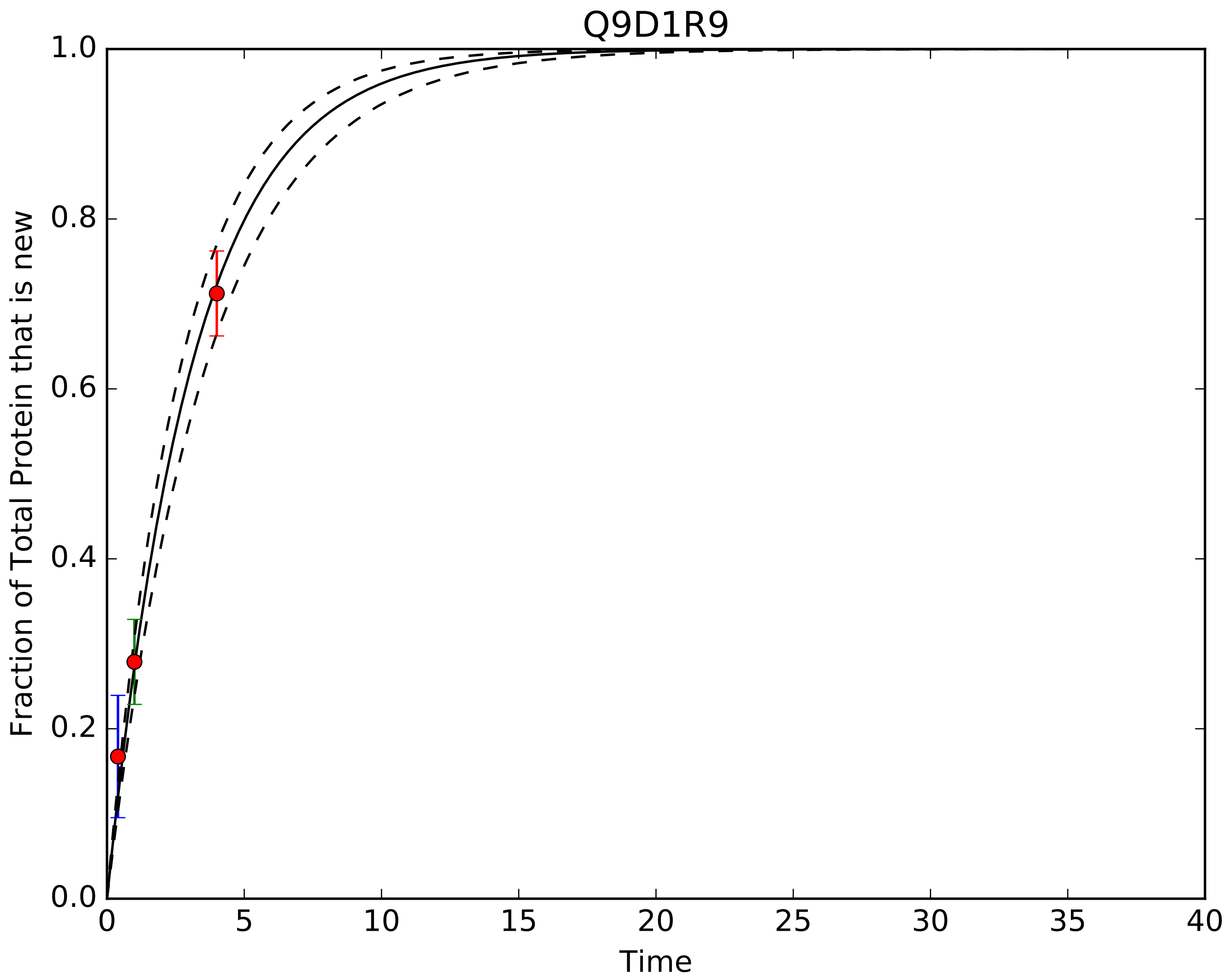

Q9D1R9
Fraction of Total Protein that is new
Time
0.0
0.2
0.4
0.6
0.8
1.0
0
5
10
15
20
25
30
35
40

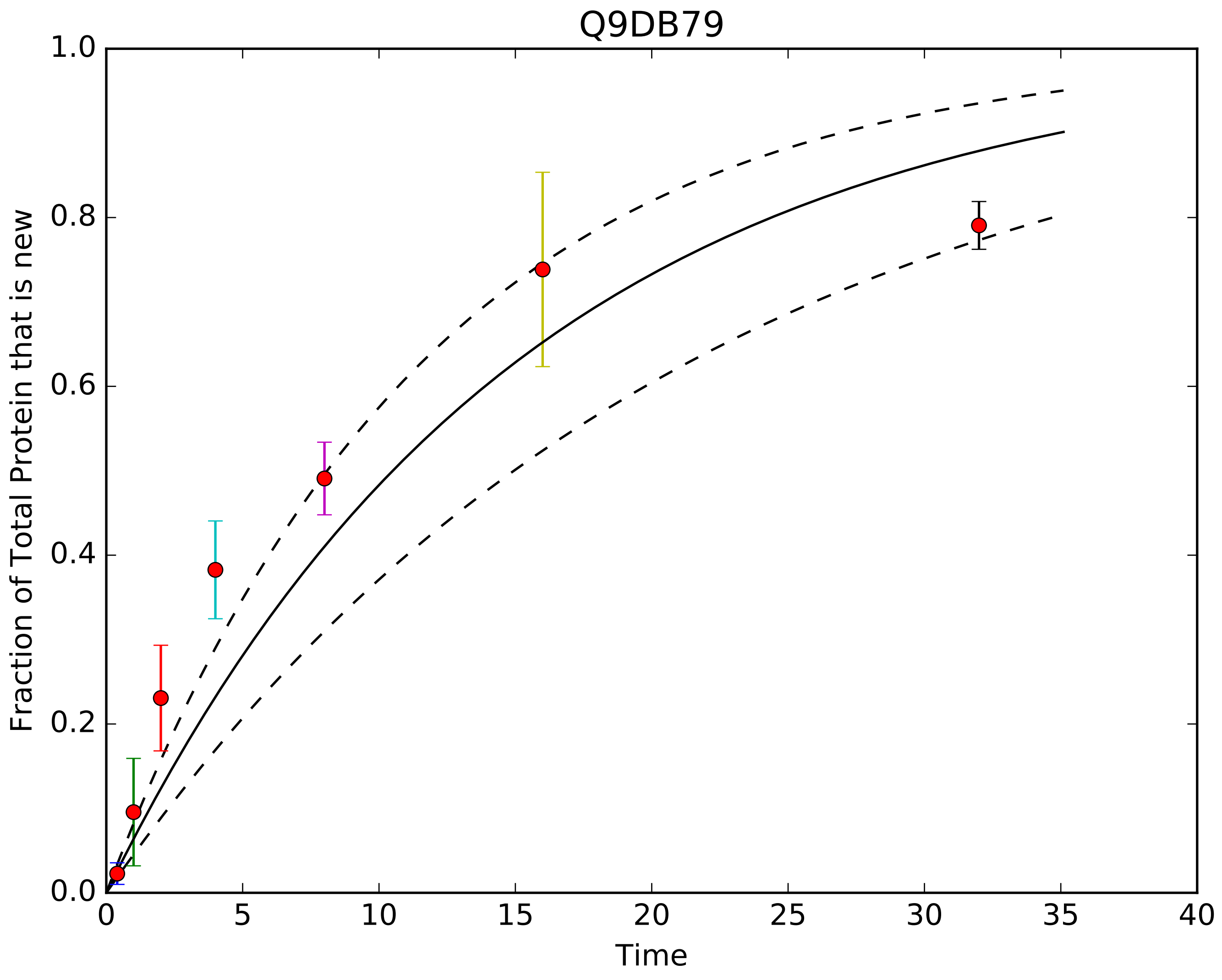
Q9DB79
Fraction of Total Protein that is new
Time
0.0
0.2
0.4
0.6
0.8
1.0
0
5
10
15
20
25
30
35
40

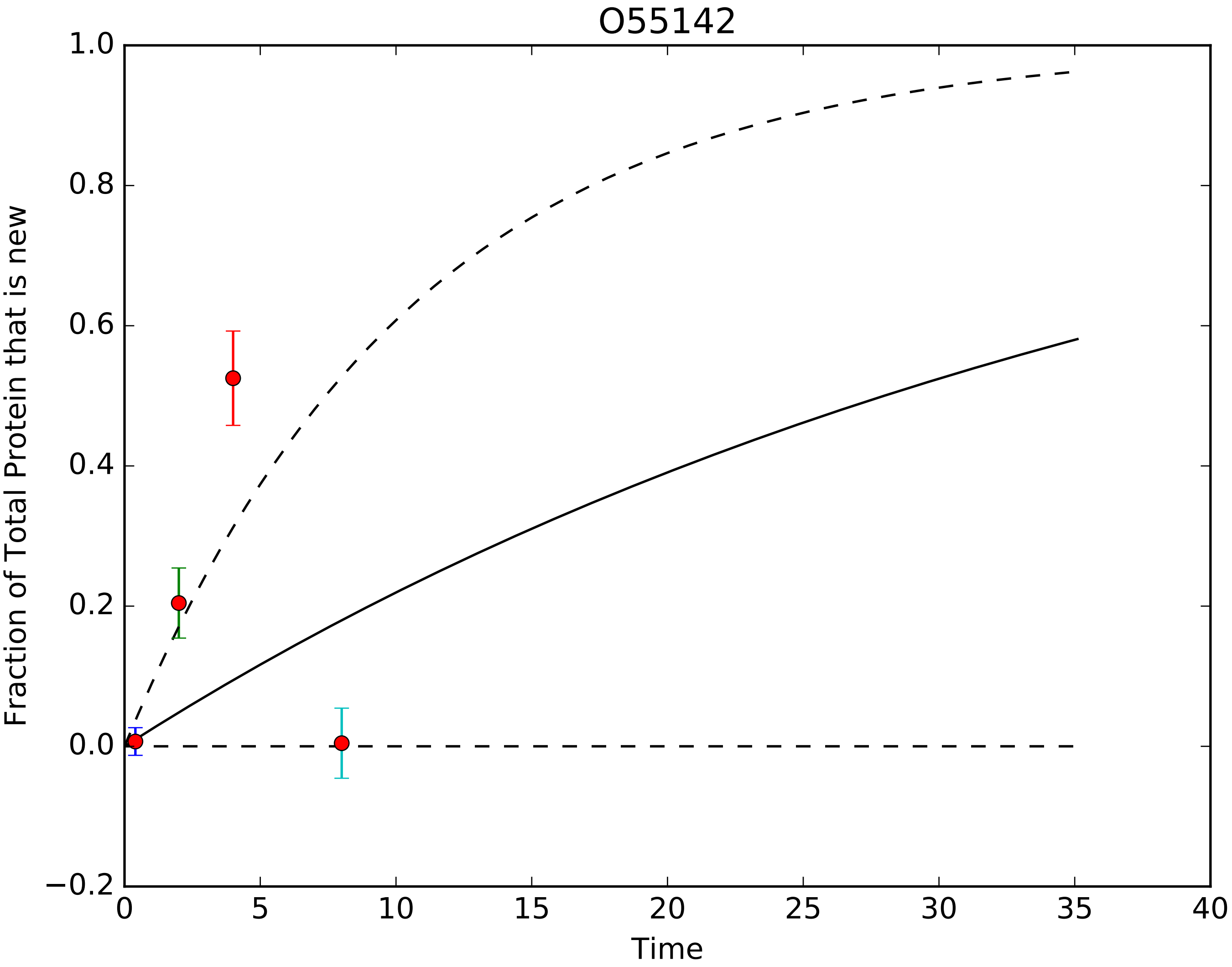

O55142
Fraction of Total Protein that is new
Time
1.0
0.8
0.6
0.4
0.2
0.0
−0.2
0
5
10
15
20
25
30
35
40
