# Supplementary material for: Mechanisms of In Vivo Ribosome Maintenance Change in Response to Nutrient Signals
Source: Mol Cell Proteomics. 2016 Dec 8;16(2):243–54. doi: 10.1074/mcp.M116.063255 (PMC5294211; doi:10.1074/mcp.M116.063255)
Supplement: Supplemental Data [file 10.1074_M116.063255_mcp.M116.063255-11.pdf]

Mechanisms of *in vivo* ribosome maintenance change in response to nutrient signals:
Supplemental information

Andrew D. Mathis[1±], Bradley C. Naylor[1±], Richard H. Carson[1], Eric Evans[1], Justin Harwell[1], Jared Knecht[1], Eric Hexem[1], Fredrick F. Peelor III[2], Benjamin F. Miller[2], Karyn L. Hamilton[2], Mark K. Transtrum[3], Benjamin T. Bikman[4], John C. Price[1*]

1- Department of Chemistry and Biochemistry, Brigham Young University, Provo, Utah 84602

2- Department of Health and Exercise Science, Colorado State University, Fort Collins, Colorado 80523

3- Department of Physics and Astronomy, Brigham Young University, Provo, Utah 84602

4- Department of Physiology and Developmental Biology, Brigham Young University, Provo, Utah 84602

± these authors contributed equally to this work

* to whom correspondence should be addressed:

John C. Price, Ph.D.

jcprice@chem.byu.edu

Brigham Young University

E113 Benson Building

Provo, Utah 84602

801-422-6040

Supplemental Table 1: Turnover rates and standard deviations of fit (SD) for ribosomal proteins are supplied as a Microsoft Excel table

Tab: *Protein Kinetic Summary* contains the summary information for each of the ribosomal proteins, including the turnover rate, the number of peptide sequences, the number of measurements used to calculate the turnover rate, the confidence interval of the rate (95% CI), and the percent coverage of the peptides that provided kinetic information in each of the four kinetic pools.

Tab: *Peptide MSMS scores* contains the sequences and Spectrum Mill MSMS scores for each sequence in the respective samples that were used for the kinetic calculations for the proteins. In all cases more peptides were identified than met the quality filters to be included in the kinetic fit. The calculated values for each peptide (including those which did not make it through the filters) used for the filtering are included in the supplemental Excel file for each of the four kinetic pools.

Graphs 1, 2, 3, 4 are the compiled fitting curves of each protein in each kinetic pool

Figure S1

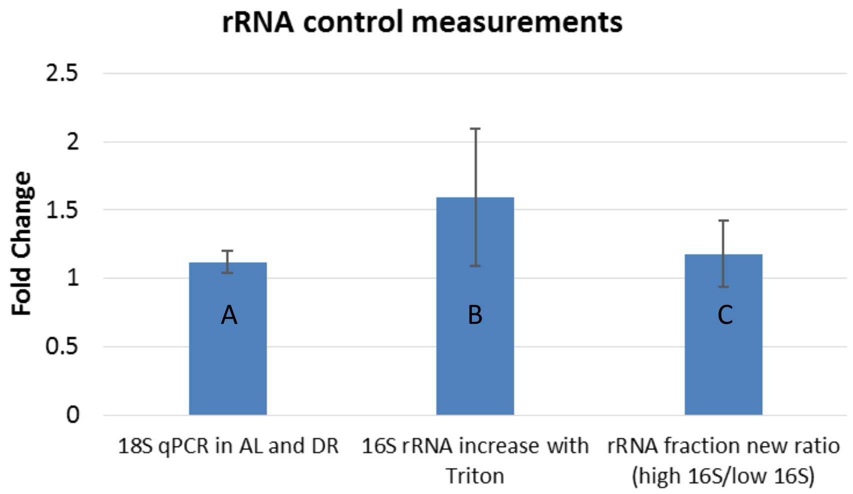

Figure S1: In order to test for biases in the rRNA turnover measurements we used qPCR to measure the total concentration of 18s rRNA in the tissue of multiple AL and DR mice (n=3 of each group). We observed that the relative concentration was not changed in AL and DR tissue (A). The total number of ribosomes as measured by 18S qPCR was not changed between DR and AL mice (n=4). In order to understand the contribution made by mitochondrial ribosomes to the rRNA turnover measurements, we modified the ribosome isolation method to include Triton X-100. Including Triton X-100 increased the 16S rRNA content ~50% in each of the samples (B, n=4). When we compared the fraction new rRNA measured in the same samples (n=4) with modified 16S content (high 16S/low 16S, column C), we saw that there was not a significant change. This suggests that the number of eukaryotic ribosomes is not changing significantly between AL and DR tissue and that the turnover measurement is not biased by any mitochondrial rRNA contamination.

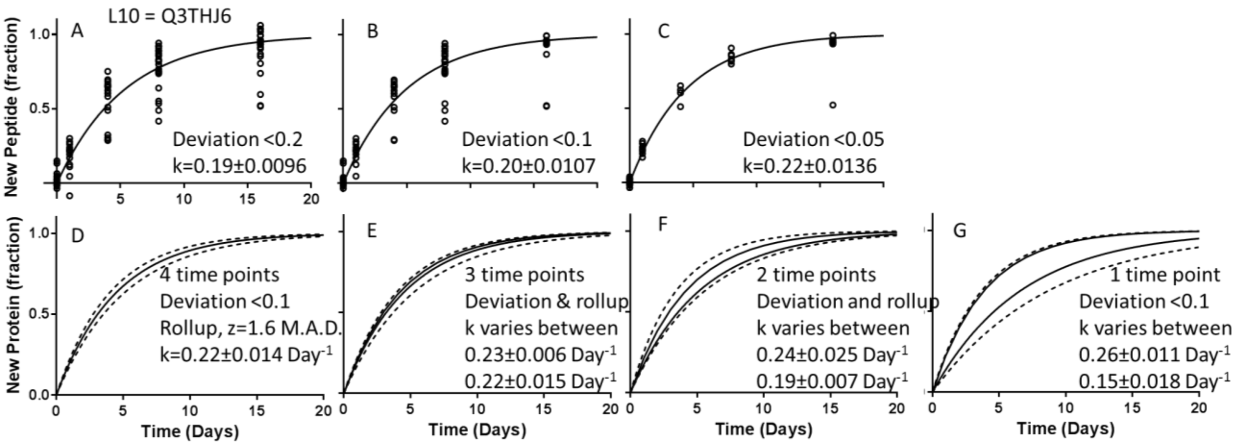

Figure S2: Data for ribosomal protein L10 (Q3THJ6) in assembled AL ribosomes is shown as an example: solid line is the fit; dotted line is the confidence interval. Extracted peptide isotope patterns (137 total) are subjected to a precision filter prior to calculating the turnover rate. Each peptide measurement (**circles in A-C**) for each protein at each time point must meet a minimum isotope precision requirement in order to be included in the rate constant calculation. Increasing the precision by reducing the allowed deviation for individual peptides is an unbiased way to remove outliers, but reduces total data density; therefore we set the global criteria for Deviation<0.1 (**panel B**). To fit the data we calculate the median value for the time point to roll up the peptides into a protein level data point. Outliers are excluded using a median absolute deviation test with z=1.4 (resulting in 47 measurements).

Removing time points allows more variability in rate constant (max and min rate values and curves shown, **panel D-G**) and lower confidence (larger 95% confidence interval, dotted lines, **D-G**). Requiring at least 3 time points minimizes the variability in the rate constant (coefficient of variance<0.1). For L10, the rate constant from any combination of three time points is still within 1 standard deviation of the rate calculated from all available time points.
